# Correction: The *Kras^G12D^;Trp53^fl/fl^* murine model of undifferentiated pleomorphic sarcoma is macrophage dense, lymphocyte poor, and resistant to immune checkpoint blockade

**DOI:** 10.1371/journal.pone.0352130

**Published:** 2026-06-22

**Authors:** Karys M. Hildebrand, Arvind K. Singla, Reid McNeil, Kayla L. Marritt, Kurt N. Hildebrand, Franz Zemp, Jahanara Rajwani, Doha Itani, Pinaki Bose, Douglas J. Mahoney, Frank R. Jirik, Michael J. Monument

After this article [1] was published, concerns were raised with Figs 1 and 3. Specifically, Figs 1F and 3I appear similar when rotated 180°.

The corresponding author stated that, following a review of the archived source image files and figure preparation materials, they identified that Fig 3I is incorrect. To address this issue, they provided an updated version of Fig 3 with a replacement Fig 3I obtained from archived experimental material generated at the time of the original experiments.

The original source image files associated with Figs 1 and 3 are provided at 10.6084/m9.figshare.32415408.

**Fig 3:**
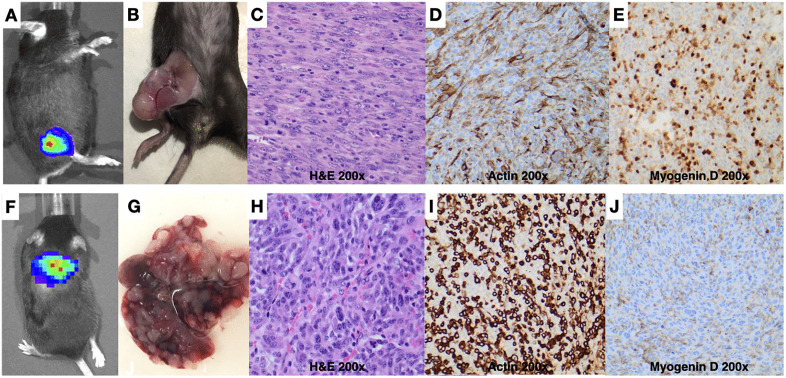
Cell lines derived from spontaneous UPS tumours are easily engrafted in the hindlimb and lung of naïve, immunologically competent C57Bl/6 mice. **(A-B)** Following injection of 100,000 UPS cells into the hindlimb of C57Bl/6 mice, tumours are detectable via bioluminescence imaging and result in large palpable tumours by three weeks. **(C-E)** Routine histology and immunohistochemistry of cell line derived tumours is consistent with UPS and similar to spontaneous UPS tumours. **(F-G)** Tail vein injections result in bioluminescence detectable tumours in the lung with multiple tumour lung nodules on gross examination of lung tissue. **(H-J)** Routine histology and immunohistochemistry of cell line derived lung tumours is consistent with UPS and similar to spontaneous UPS tumours.

## References

[1] Hildebrand KM, Singla AK, McNeil R, Marritt KL, Hildebrand KN, Zemp F, et al. The *Kras^G12D^*;*Trp53^fl/fl^* murine model of undifferentiated pleomorphic sarcoma is macrophage dense, lymphocyte poor, and resistant to immune checkpoint blockade. PLoS One. 2021;16(7):e0253864. doi: 10.1371/journal.pone.0253864 34242269 PMC8270133

