# Supplementary material for: The KrasG12D;Trp53fl/fl murine model of undifferentiated pleomorphic sarcoma is macrophage dense, lymphocyte poor, and resistant to immune checkpoint blockade
Source: PLoS One. 2021 Jul 9;16(7):e0253864. doi: 10.1371/journal.pone.0253864 (PMC8270133; doi:10.1371/journal.pone.0253864)
Supplement: S1 File — Complete list of differentially downregulated murine genes relative to human UPS. (PDF) [file pone.0253864.s001.pdf]

	baseMean	log2FoldCh	lfcSE	stat	pvalue	padj	threshold	diffexpresse
USP9Y	504.4954	-23.7179	2.484076	-9.54797	1.32E-21	2.51E-19	FALSE	DOWN
NKX2.5	220.2177	-22.5392	2.416654	-9.3266	1.09E-20	1.91E-18	FALSE	DOWN
SPRR2F	42.58371	-20.8273	3.900616	-5.33948	9.32E-08	1.84E-06	FALSE	DOWN
CLEC3A	39.03892	-20.7213	2.873231	-7.21184	5.52E-13	3.41E-11	FALSE	DOWN
MAGEB2	18.82577	-19.6491	3.755501	-5.23208	1.68E-07	3.09E-06	FALSE	DOWN
MAGEA3	20.86991	-19.6415	3.976792	-4.93902	7.85E-07	1.19E-05	FALSE	DOWN
MAGEA6	30.01398	-19.3819	3.708917	-5.22575	1.73E-07	3.18E-06	FALSE	DOWN
CYP2W1	93.93111	-19.2893	2.631146	-7.33115	2.28E-13	1.49E-11	FALSE	DOWN
RNASE1	33078.16	-16.0243	1.592852	-10.0602	8.29E-24	2.08E-21	FALSE	DOWN
CALCOCO2	5270.384	-13.3745	1.221854	-10.9461	6.94E-28	2.40E-25	FALSE	DOWN
PLA2G2A	9152.813	-12.302	2.289574	-5.37304	7.74E-08	1.56E-06	FALSE	DOWN
A2M	118047.7	-12.2391	0.824321	-14.8474	7.23E-50	1.43E-46	FALSE	DOWN
MYOM1	2002.6	-11.9784	1.573106	-7.6145	2.65E-14	1.98E-12	FALSE	DOWN
CPVL	2431.829	-11.2674	1.402872	-8.03169	9.61E-16	9.43E-14	FALSE	DOWN
NIPSNAP3A	1093.27	-11.1054	1.222184	-9.08653	1.02E-19	1.54E-17	FALSE	DOWN
FPR3	1049.565	-11.0463	1.410623	-7.83082	4.85E-15	4.06E-13	FALSE	DOWN
H3F3C	1521.359	-10.6406	1.191171	-8.93291	4.15E-19	5.86E-17	FALSE	DOWN
SCUBE3	1275.425	-10.4237	2.020905	-5.15793	2.50E-07	4.37E-06	FALSE	DOWN
WFDC2	1920.018	-10.3554	1.993965	-5.19336	2.07E-07	3.71E-06	FALSE	DOWN
UBE2D4	648.3527	-10.351	1.229103	-8.42162	3.71E-17	4.11E-15	FALSE	DOWN
MYH11	256206.8	-10.3003	1.799302	-5.72462	1.04E-08	2.73E-07	FALSE	DOWN
SCRG1	1070.316	-10.1689	1.921629	-5.29179	1.21E-07	2.33E-06	FALSE	DOWN
METTL7B	474.4428	-9.90071	1.671277	-5.92404	3.14E-09	9.29E-08	FALSE	DOWN
RGN	447.686	-9.81718	1.829332	-5.36653	8.03E-08	1.61E-06	FALSE	DOWN
IGSF21	432.811	-9.76842	1.563651	-6.24719	4.18E-10	1.51E-08	FALSE	DOWN
LNP1	392.051	-9.62564	1.305536	-7.37294	1.67E-13	1.14E-11	FALSE	DOWN
LRRC37A	661.001	-9.41663	1.1837	-7.95525	1.79E-15	1.68E-13	FALSE	DOWN
AMY2B	335.1457	-9.39939	1.353998	-6.94195	3.87E-12	2.11E-10	FALSE	DOWN
GSTM3	2330.408	-9.3272	0.960488	-9.7109	2.71E-22	5.68E-20	FALSE	DOWN
HOXB13	256.6102	-9.01421	2.284726	-3.94542	7.97E-05	0.000637	FALSE	DOWN
C7	12159.6	-8.92452	1.438853	-6.20252	5.56E-10	1.98E-08	FALSE	DOWN
CCDC113	235.1384	-8.88818	1.405889	-6.32211	2.58E-10	9.81E-09	FALSE	DOWN
SPESP1	234.6419	-8.88476	1.795629	-4.948	7.50E-07	1.15E-05	FALSE	DOWN
PCDHA6	211.6731	-8.73649	2.314331	-3.77495	0.00016	0.001148	FALSE	DOWN
LDLRAD2	1115.234	-8.64664	0.946494	-9.13544	6.51E-20	1.00E-17	FALSE	DOWN
SCG5	1272.289	-8.60518	1.545535	-5.56777	2.58E-08	6.00E-07	FALSE	DOWN
CCDC121	191.1777	-8.58984	1.265196	-6.78934	1.13E-11	5.45E-10	FALSE	DOWN
LBH	4876.805	-8.5524	0.696911	-12.2719	1.28E-34	6.82E-32	FALSE	DOWN
LRRC25	983.611	-8.51358	1.218353	-6.98778	2.79E-12	1.58E-10	FALSE	DOWN
NMUR1	332.4095	-8.47407	1.644473	-5.15306	2.56E-07	4.47E-06	FALSE	DOWN
RPE65	614.7921	-8.4429	2.192589	-3.85065	0.000118	0.000883	FALSE	DOWN
FMO3	1807.401	-8.41512	1.765362	-4.76679	1.87E-06	2.57E-05	FALSE	DOWN
GPBAR1	168.5633	-8.40716	1.567509	-5.36339	8.17E-08	1.64E-06	FALSE	DOWN
ADRA2C	659.1574	-8.35523	1.537755	-5.43339	5.53E-08	1.17E-06	FALSE	DOWN
CYTL1	286.0762	-8.25521	1.591856	-5.1859	2.15E-07	3.85E-06	FALSE	DOWN
LMOD1	27036.34	-8.12146	1.479925	-5.48775	4.07E-08	8.92E-07	FALSE	DOWN
ENAM	136.2517	-8.10094	2.110717	-3.838	0.000124	0.000921	FALSE	DOWN

PNMAL1	1440.429	-8.08283	1.071779	-7.54151	4.65E-14	3.36E-12	FALSE	DOWN
SLC2A5	1010.525	-8.05926	1.313469	-6.13586	8.47E-10	2.87E-08	FALSE	DOWN
CPZ	5657.311	-7.99499	1.627352	-4.91288	8.97E-07	1.35E-05	FALSE	DOWN
ALDOB	124.5065	-7.96932	2.033027	-3.91993	8.86E-05	0.000694	FALSE	DOWN
RAB17	113.5389	-7.83685	1.578276	-4.96545	6.85E-07	1.06E-05	FALSE	DOWN
TMSB15A	359.4918	-7.77787	1.3888	-5.60042	2.14E-08	5.07E-07	FALSE	DOWN
CTSG	462.5315	-7.74272	1.833799	-4.22223	2.42E-05	0.000236	FALSE	DOWN
IL22RA1	104.8497	-7.72331	1.656999	-4.66102	3.15E-06	4.04E-05	FALSE	DOWN
DUOXA2	102.7727	-7.69414	2.135898	-3.6023	0.000315	0.002019	FALSE	DOWN
NRSN2	2444.965	-7.63196	0.560005	-13.6284	2.72E-42	2.35E-39	FALSE	DOWN
CDRT4	424.0609	-7.55902	0.979097	-7.7204	1.16E-14	9.17E-13	FALSE	DOWN
SPTLC3	575.7603	-7.54363	1.024804	-7.36105	1.82E-13	1.22E-11	FALSE	DOWN
IFI44L	2059.447	-7.53838	0.946412	-7.96522	1.65E-15	1.56E-13	FALSE	DOWN
BEX4	1317.236	-7.50764	0.805184	-9.32413	1.12E-20	1.94E-18	FALSE	DOWN
SERPINA5	89.10454	-7.48789	1.811111	-4.13442	3.56E-05	0.000322	FALSE	DOWN
PCDHA3	88.16688	-7.47314	1.815889	-4.11542	3.86E-05	0.000346	FALSE	DOWN
KCNK1	374.5037	-7.43192	1.168299	-6.36132	2.00E-10	7.73E-09	FALSE	DOWN
TRIM38	1245.54	-7.39163	0.562827	-13.133	2.13E-39	1.40E-36	FALSE	DOWN
TMEM59L	336.8385	-7.39152	1.589294	-4.65082	3.31E-06	4.22E-05	FALSE	DOWN
PTGDS	2719.936	-7.35672	1.528735	-4.8123	1.49E-06	2.11E-05	FALSE	DOWN
STMN3	3590.846	-7.35382	0.714895	-10.2866	8.10E-25	2.20E-22	FALSE	DOWN
ATM	2027.313	-7.33354	0.521285	-14.0682	5.96E-45	6.87E-42	FALSE	DOWN
DEFB1	79.24961	-7.31861	2.368773	-3.08962	0.002004	0.009449	FALSE	DOWN
NAT1	154.6658	-7.30922	1.268099	-5.76392	8.22E-09	2.19E-07	FALSE	DOWN
CD70	231.6285	-7.28677	1.600325	-4.5533	5.28E-06	6.32E-05	FALSE	DOWN
SPINK5	75.42133	-7.24786	1.681986	-4.30911	1.64E-05	0.000167	FALSE	DOWN
SCG2	2635.96	-7.24208	1.442556	-5.02031	5.16E-07	8.30E-06	FALSE	DOWN
CLDN4	154.0257	-7.24019	1.501748	-4.82117	1.43E-06	2.03E-05	FALSE	DOWN
LYPD3	73.87983	-7.21748	1.4098	-5.11951	3.06E-07	5.24E-06	FALSE	DOWN
TFF3	137.4875	-7.20775	1.904245	-3.7851	0.000154	0.001109	FALSE	DOWN
GPAT2	499.871	-7.20205	1.397069	-5.15511	2.53E-07	4.42E-06	FALSE	DOWN
LDOC1	3964.039	-7.17787	0.993749	-7.22302	5.08E-13	3.20E-11	FALSE	DOWN
C2CD4B	70.64201	-7.15301	1.606642	-4.45215	8.50E-06	9.46E-05	FALSE	DOWN
CCL26	69.51771	-7.12928	1.731881	-4.1165	3.85E-05	0.000345	FALSE	DOWN
PM20D2	995.3443	-7.0989	0.771312	-9.20366	3.46E-20	5.50E-18	FALSE	DOWN
NOXA1	127.9293	-7.08264	1.375711	-5.14835	2.63E-07	4.56E-06	FALSE	DOWN
PCDHA1	65.72797	-7.04947	2.022668	-3.48523	0.000492	0.002932	FALSE	DOWN
PTP4A1	7078.61	-7.04112	0.528409	-13.3251	1.65E-40	1.27E-37	FALSE	DOWN
FAM162B	122.3746	-7.0275	1.530968	-4.59023	4.43E-06	5.43E-05	FALSE	DOWN
MYL12B	10679.14	-7.01093	0.413851	-16.9407	2.25E-64	1.56E-60	FALSE	DOWN
FFAR3	60.03846	-6.91785	1.843651	-3.75225	0.000175	0.001236	FALSE	DOWN
CFD	7243.944	-6.91421	1.455779	-4.74949	2.04E-06	2.75E-05	FALSE	DOWN
PABPC5	184.7387	-6.82623	1.21137	-5.63513	1.75E-08	4.28E-07	FALSE	DOWN
PERP	1228.955	-6.81799	1.40422	-4.85535	1.20E-06	1.75E-05	FALSE	DOWN
IQCG	381.2691	-6.78886	0.860829	-7.88642	3.11E-15	2.67E-13	FALSE	DOWN
NUPL1	1589.973	-6.78673	0.494792	-13.7163	8.11E-43	7.48E-40	FALSE	DOWN
TMEM217	108.519	-6.7591	1.392048	-4.85551	1.20E-06	1.75E-05	FALSE	DOWN
MYLK	77837.25	-6.74192	1.376272	-4.89868	9.65E-07	1.44E-05	FALSE	DOWN

PRIMA1	370.7971	-6.65374	1.495068	-4.45046	8.57E-06	9.53E-05	FALSE	DOWN
IDO1	1063.647	-6.56814	1.16781	-5.62432	1.86E-08	4.50E-07	FALSE	DOWN
ST6GAL2	555.0445	-6.56436	1.235798	-5.31184	1.09E-07	2.11E-06	FALSE	DOWN
ALDH1B1	11494.2	-6.56093	1.078625	-6.08268	1.18E-09	3.82E-08	FALSE	DOWN
KCNG1	858.801	-6.5604	1.380425	-4.75245	2.01E-06	2.73E-05	FALSE	DOWN
HAPLN3	1509.494	-6.54158	0.887703	-7.36911	1.72E-13	1.16E-11	FALSE	DOWN
CCR6	45.89019	-6.53231	1.410483	-4.63126	3.63E-06	4.58E-05	FALSE	DOWN
SPATA20	2727.915	-6.52482	0.589646	-11.0657	1.84E-28	6.70E-26	FALSE	DOWN
ANKRD22	206.9563	-6.51353	1.565981	-4.15939	3.19E-05	0.000295	FALSE	DOWN
KLRB1	165.8177	-6.50879	1.364599	-4.76975	1.84E-06	2.54E-05	FALSE	DOWN
09-Mar	5863.777	-6.48713	1.13491	-5.71598	1.09E-08	2.84E-07	FALSE	DOWN
CPM	3301.686	-6.44193	0.915888	-7.03353	2.01E-12	1.16E-10	FALSE	DOWN
CYBRD1	12714.8	-6.43335	0.553729	-11.6182	3.33E-31	1.49E-28	FALSE	DOWN
MUC20	186.4916	-6.43324	1.15577	-5.5662	2.60E-08	6.03E-07	FALSE	DOWN
NAP1L3	618.0885	-6.43162	1.093235	-5.88311	4.03E-09	1.15E-07	FALSE	DOWN
CLDN14	42.71488	-6.42699	1.782428	-3.60575	0.000311	0.002	FALSE	DOWN
CRTAC1	1169.376	-6.41631	1.399295	-4.58539	4.53E-06	5.53E-05	FALSE	DOWN
IGF2	170499.6	-6.39223	1.7523	-3.64791	0.000264	0.001754	FALSE	DOWN
ACBD7	125.6078	-6.37682	1.280747	-4.97898	6.39E-07	1.00E-05	FALSE	DOWN
FBP1	355.0838	-6.36791	1.213552	-5.24733	1.54E-07	2.90E-06	FALSE	DOWN
RERGL	40.52951	-6.35184	1.814911	-3.49981	0.000466	0.002802	FALSE	DOWN
ALPL	5271.303	-6.31732	1.304102	-4.84419	1.27E-06	1.84E-05	FALSE	DOWN
FATE1	39.5208	-6.31335	1.618674	-3.90032	9.61E-05	0.000744	FALSE	DOWN
CFI	2017.505	-6.30936	1.037699	-6.08015	1.20E-09	3.87E-08	FALSE	DOWN
SPOCK1	5124.734	-6.28982	0.986253	-6.37749	1.80E-10	7.06E-09	FALSE	DOWN
FCGBP	5331.335	-6.26797	1.353791	-4.62994	3.66E-06	4.60E-05	FALSE	DOWN
XRRA1	1002.188	-6.26623	0.501866	-12.4859	8.92E-36	5.14E-33	FALSE	DOWN
MFRP	3131.086	-6.22285	0.80777	-7.70374	1.32E-14	1.03E-12	FALSE	DOWN
LYG1	37.00644	-6.21782	1.293382	-4.80741	1.53E-06	2.14E-05	FALSE	DOWN
PNMA1	4231.205	-6.21595	0.475759	-13.0653	5.20E-39	3.13E-36	FALSE	DOWN
TCF21	3256.039	-6.19033	1.614127	-3.83509	0.000126	0.00093	FALSE	DOWN
APOC1	1595.508	-6.16708	1.249036	-4.93747	7.91E-07	1.20E-05	FALSE	DOWN
CRISPLD1	1783.716	-6.16018	1.220507	-5.04723	4.48E-07	7.34E-06	FALSE	DOWN
KLHL6	298.7543	-6.13677	1.011483	-6.0671	1.30E-09	4.17E-08	FALSE	DOWN
HSD11B2	478.1221	-6.13367	1.268639	-4.83484	1.33E-06	1.92E-05	FALSE	DOWN
SLC17A7	226.2385	-6.13366	1.412565	-4.34221	1.41E-05	0.000147	FALSE	DOWN
SLC27A2	65.24315	-6.12361	1.604671	-3.81612	0.000136	0.000992	FALSE	DOWN
FHL5	147.141	-6.1232	1.417213	-4.3206	1.56E-05	0.00016	FALSE	DOWN
PARM1	5180.046	-6.11981	0.955022	-6.40803	1.47E-10	5.87E-09	FALSE	DOWN
DLX5	477.3877	-6.11148	1.241833	-4.92134	8.60E-07	1.29E-05	FALSE	DOWN
ACOX2	607.1092	-6.07712	0.914865	-6.64264	3.08E-11	1.37E-09	FALSE	DOWN
TNFAIP8L3	2148.031	-6.04945	1.040826	-5.81216	6.17E-09	1.69E-07	FALSE	DOWN
TMEM54	2159.155	-6.04801	0.705839	-8.56853	1.05E-17	1.22E-15	FALSE	DOWN
AOC3	14127.77	-6.04455	1.109688	-5.44707	5.12E-08	1.10E-06	FALSE	DOWN
TMEM30B	617.4031	-6.00642	1.137886	-5.27858	1.30E-07	2.48E-06	FALSE	DOWN
BTN2A2	809.7289	-5.9837	0.566259	-10.5671	4.24E-26	1.25E-23	FALSE	DOWN
BEX2	170.1453	-5.98093	1.520913	-3.93246	8.41E-05	0.000666	FALSE	DOWN
KEL	31.30225	-5.97855	1.672709	-3.57417	0.000351	0.002208	FALSE	DOWN

S100A2	187.3415	-5.9619	1.4248	-4.18438	2.86E-05	0.000269	FALSE	DOWN
GRIN2C	113.1411	-5.94323	1.402414	-4.23786	2.26E-05	0.000221	FALSE	DOWN
TAF7L	30.44617	-5.93933	1.804402	-3.29158	0.000996	0.005329	FALSE	DOWN
FAM198A	844.5968	-5.91601	0.922745	-6.41131	1.44E-10	5.77E-09	FALSE	DOWN
PGM5	8321.112	-5.91128	1.209236	-4.88844	1.02E-06	1.51E-05	FALSE	DOWN
MSMP	56.6603	-5.90173	1.256429	-4.69722	2.64E-06	3.46E-05	FALSE	DOWN
OLFML1	2062.24	-5.90108	0.875958	-6.73671	1.62E-11	7.60E-10	FALSE	DOWN
SPOCD1	1044.087	-5.89712	1.400657	-4.21025	2.55E-05	0.000246	FALSE	DOWN
TDRD6	29.38309	-5.8876	1.448644	-4.06421	4.82E-05	0.000418	FALSE	DOWN
CD163L1	732.4987	-5.87243	1.035215	-5.67266	1.41E-08	3.53E-07	FALSE	DOWN
UBE2NL	28.98093	-5.87157	1.310134	-4.48165	7.41E-06	8.41E-05	FALSE	DOWN
PLA2G5	303.6998	-5.86378	1.416115	-4.14075	3.46E-05	0.000315	FALSE	DOWN
CNTNAP3	322.1356	-5.85697	1.411424	-4.14969	3.33E-05	0.000305	FALSE	DOWN
FAM107A	1132.503	-5.85603	0.803508	-7.28808	3.14E-13	2.02E-11	FALSE	DOWN
RARRES1	2341.301	-5.84136	1.276499	-4.57608	4.74E-06	5.76E-05	FALSE	DOWN
CCDC81	226.3109	-5.81577	1.336933	-4.35008	1.36E-05	0.000142	FALSE	DOWN
NKX3.1	152.915	-5.79705	1.35047	-4.29262	1.77E-05	0.000179	FALSE	DOWN
DNALI1	399.2048	-5.78933	1.158352	-4.99791	5.80E-07	9.21E-06	FALSE	DOWN
GPR1	539.2774	-5.77915	1.342337	-4.30529	1.67E-05	0.00017	FALSE	DOWN
KCNJ12	622.745	-5.77558	0.969995	-5.95423	2.61E-09	7.89E-08	FALSE	DOWN
FAM122C	100.0237	-5.76964	1.043586	-5.52866	3.23E-08	7.26E-07	FALSE	DOWN
ALDH1A3	8438.738	-5.76872	1.369562	-4.21209	2.53E-05	0.000244	FALSE	DOWN
CIDEB	952.6766	-5.76352	0.56255	-10.2453	1.24E-24	3.24E-22	FALSE	DOWN
SPATA18	679.6382	-5.73128	1.207349	-4.74699	2.06E-06	2.78E-05	FALSE	DOWN
LCN10	25.94292	-5.70826	1.712881	-3.33255	0.000861	0.004717	FALSE	DOWN
CYP1B1	5143.59	-5.69498	1.011622	-5.62956	1.81E-08	4.39E-07	FALSE	DOWN
NTRK1	112.4805	-5.68463	1.470878	-3.86479	0.000111	0.000845	FALSE	DOWN
SYNPO2	32128.28	-5.65659	1.300807	-4.34853	1.37E-05	0.000143	FALSE	DOWN
CLSTN3	2310.043	-5.61808	0.607849	-9.24256	2.41E-20	3.96E-18	FALSE	DOWN
EMILIN3	1371.599	-5.58289	1.392801	-4.00839	6.11E-05	0.000512	FALSE	DOWN
FST	1149.309	-5.57868	0.931561	-5.98853	2.12E-09	6.50E-08	FALSE	DOWN
KCTD20	3227.989	-5.5648	0.460065	-12.0957	1.11E-33	5.50E-31	FALSE	DOWN
VSIG4	4555.097	-5.54354	1.030716	-5.37834	7.52E-08	1.52E-06	FALSE	DOWN
SLC25A1	3028.214	-5.53362	0.384347	-14.3975	5.37E-47	7.43E-44	FALSE	DOWN
DYRK4	762.6674	-5.53345	0.635341	-8.70942	3.05E-18	3.84E-16	FALSE	DOWN
TNFSF4	926.5025	-5.53186	1.034979	-5.3449	9.05E-08	1.80E-06	FALSE	DOWN
PATL2	45.47533	-5.52587	1.407181	-3.92691	8.60E-05	0.00068	FALSE	DOWN
UHRF1BP1	857.1322	-5.51337	0.510421	-10.8016	3.38E-27	1.11E-24	FALSE	DOWN
KCNMB1	5181.761	-5.51039	1.465998	-3.7588	0.000171	0.00121	FALSE	DOWN
EYA2	1717.025	-5.50661	0.87865	-6.26713	3.68E-10	1.35E-08	FALSE	DOWN
MDH1B	42.49524	-5.49549	1.41906	-3.87262	0.000108	0.000823	FALSE	DOWN
TREX2	22.32812	-5.49398	1.44341	-3.80625	0.000141	0.001027	FALSE	DOWN
TPPP	656.4728	-5.48353	1.292146	-4.24374	2.20E-05	0.000217	FALSE	DOWN
HRH1	1591.434	-5.47908	0.736648	-7.43785	1.02E-13	7.04E-12	FALSE	DOWN
TBX19	179.1729	-5.47404	0.848637	-6.45039	1.12E-10	4.58E-09	FALSE	DOWN
FKBP5	8604.702	-5.45913	0.767542	-7.11249	1.14E-12	6.74E-11	FALSE	DOWN
PPIL6	98.41086	-5.45697	1.038611	-5.2541	1.49E-07	2.81E-06	FALSE	DOWN
HS3ST2	557.9501	-5.44989	1.540075	-3.53872	0.000402	0.002474	FALSE	DOWN

SYNM	22177.24	-5.44689	1.291774	-4.2166	2.48E-05	0.000241	FALSE	DOWN
KRT86	40.88723	-5.35738	1.568686	-3.4152	0.000637	0.003661	FALSE	DOWN
RSPO3	2324.651	-5.35291	1.275722	-4.19598	2.72E-05	0.000259	FALSE	DOWN
MST1	234.0614	-5.33839	0.985498	-5.41695	6.06E-08	1.26E-06	FALSE	DOWN
TFAP2E	75.53819	-5.33346	1.058119	-5.04051	4.64E-07	7.57E-06	FALSE	DOWN
PTPRU	1220.506	-5.33024	0.952302	-5.59721	2.18E-08	5.15E-07	FALSE	DOWN
LIMS2	5284.542	-5.32338	0.844371	-6.30454	2.89E-10	1.08E-08	FALSE	DOWN
FAM24B	62.91081	-5.32282	1.274937	-4.17497	2.98E-05	0.000278	FALSE	DOWN
OLFM2	1956.748	-5.31404	1.243108	-4.2748	1.91E-05	0.000192	FALSE	DOWN
RIBC2	126.2625	-5.30357	1.125959	-4.71027	2.47E-06	3.26E-05	FALSE	DOWN
ACTG2	78309.05	-5.30062	1.615158	-3.2818	0.001031	0.005468	FALSE	DOWN
CATSPER1	19.49316	-5.29372	1.564601	-3.38343	0.000716	0.004031	FALSE	DOWN
GPC3	4484.199	-5.29262	1.484216	-3.56594	0.000363	0.002262	FALSE	DOWN
CPS1	526.8267	-5.26597	0.925157	-5.69198	1.26E-08	3.20E-07	FALSE	DOWN
SLC24A1	525.7436	-5.2432	0.488595	-10.7312	7.27E-27	2.28E-24	FALSE	DOWN
CCDC146	544.2146	-5.22803	0.870897	-6.00304	1.94E-09	5.97E-08	FALSE	DOWN
SFRP1	6262.644	-5.22612	1.139665	-4.58566	4.53E-06	5.53E-05	FALSE	DOWN
MAOB	5192.218	-5.21211	1.411397	-3.69287	0.000222	0.001512	FALSE	DOWN
HSD17B6	975.2035	-5.2111	1.355736	-3.84374	0.000121	0.000903	FALSE	DOWN
SNURF	143.9374	-5.20948	0.9942	-5.23987	1.61E-07	2.99E-06	FALSE	DOWN
TPSAB1	1649.765	-5.20939	1.492058	-3.49141	0.00048	0.002877	FALSE	DOWN
TNXB	28614.45	-5.18556	1.361319	-3.80922	0.000139	0.001017	FALSE	DOWN
RBP7	2011.738	-5.18458	0.999874	-5.18523	2.16E-07	3.86E-06	FALSE	DOWN
CLEC4F	17.92254	-5.17687	1.571794	-3.29361	0.000989	0.005301	FALSE	DOWN
CELF6	247.1904	-5.17677	0.911385	-5.68012	1.35E-08	3.40E-07	FALSE	DOWN
LY6K	281.0076	-5.17202	1.421105	-3.63944	0.000273	0.0018	FALSE	DOWN
SYTL3	244.9642	-5.15622	0.858625	-6.0052	1.91E-09	5.90E-08	FALSE	DOWN
PTGER3	1999.903	-5.14365	1.248145	-4.12104	3.77E-05	0.000339	FALSE	DOWN
ELF3	51.89822	-5.12839	1.604382	-3.19649	0.001391	0.006976	FALSE	DOWN
MAP7D3	1131.88	-5.12476	0.568204	-9.01922	1.89E-19	2.76E-17	FALSE	DOWN
SMOC1	2800.425	-5.08705	1.568086	-3.24411	0.001178	0.006089	FALSE	DOWN
QPRT	1116.435	-5.07066	0.896137	-5.65835	1.53E-08	3.81E-07	FALSE	DOWN
PCDHB2	451.923	-5.06828	1.038034	-4.88258	1.05E-06	1.55E-05	FALSE	DOWN
ARHGEF37	949.0654	-5.05655	0.895124	-5.64899	1.61E-08	3.99E-07	FALSE	DOWN
TCEAL3	3364.486	-5.03256	0.775343	-6.49076	8.54E-11	3.58E-09	FALSE	DOWN
HSPB6	29545.79	-5.02946	0.961668	-5.22993	1.70E-07	3.12E-06	FALSE	DOWN
LMO3	2164.966	-5.01247	1.625138	-3.08434	0.00204	0.009598	FALSE	DOWN
HNMT	2743.801	-5.01194	0.562375	-8.91211	5.01E-19	7.00E-17	FALSE	DOWN
SNCG	826.8535	-5.00822	1.029519	-4.86462	1.15E-06	1.69E-05	FALSE	DOWN
HRASLS5	489.8575	-5.00529	1.262068	-3.96594	7.31E-05	0.000594	FALSE	DOWN
RASD2	2227.546	-4.97753	1.336847	-3.72334	0.000197	0.001364	FALSE	DOWN
SAMD11	544.548	-4.96632	1.371288	-3.62165	0.000293	0.001908	FALSE	DOWN
CITED4	596.0332	-4.94662	0.847995	-5.83331	5.43E-09	1.51E-07	FALSE	DOWN
CD300C	162.1742	-4.92567	1.143432	-4.30779	1.65E-05	0.000168	FALSE	DOWN
SDSL	668.4243	-4.92558	0.741477	-6.64293	3.08E-11	1.37E-09	FALSE	DOWN
PIPOX	105.7192	-4.92005	0.943444	-5.21499	1.84E-07	3.36E-06	FALSE	DOWN
CBS	1049.89	-4.91866	0.950531	-5.17465	2.28E-07	4.05E-06	FALSE	DOWN
DVL3	5640.065	-4.90929	0.329651	-14.8924	3.69E-50	8.52E-47	FALSE	DOWN

FAM50B	476.6322	-4.90439	0.753312	-6.51044	7.49E-11	3.17E-09	FALSE	DOWN
PNMA2	1189.34	-4.90271	1.009526	-4.85644	1.20E-06	1.75E-05	FALSE	DOWN
LAMA3	2881.577	-4.89131	1.01716	-4.80879	1.52E-06	2.14E-05	FALSE	DOWN
NINJ2	258.0284	-4.88673	1.054143	-4.63574	3.56E-06	4.49E-05	FALSE	DOWN
PCDHB10	571.3384	-4.88655	0.885493	-5.51845	3.42E-08	7.66E-07	FALSE	DOWN
TEKT4	14.62084	-4.87777	1.51145	-3.22721	0.00125	0.006399	FALSE	DOWN
TOX2	1628.04	-4.86712	0.933452	-5.21411	1.85E-07	3.37E-06	FALSE	DOWN
HIST1H2BK	1654.364	-4.85032	0.71483	-6.78528	1.16E-11	5.59E-10	FALSE	DOWN
SATL1	51.19422	-4.83224	1.029081	-4.69569	2.66E-06	3.48E-05	FALSE	DOWN
P2RX1	1000.18	-4.82614	1.46116	-3.30295	0.000957	0.005155	FALSE	DOWN
CLEC3B	10376.96	-4.80872	1.266598	-3.79657	0.000147	0.001064	FALSE	DOWN
ROBO3	552.7645	-4.7892	0.824984	-5.80521	6.43E-09	1.75E-07	FALSE	DOWN
PCDHB4	450.3941	-4.77646	1.063757	-4.49018	7.12E-06	8.14E-05	FALSE	DOWN
PPARD	2758.584	-4.76675	0.364362	-13.0825	4.15E-39	2.61E-36	FALSE	DOWN
ZSCAN18	3103.854	-4.75367	0.588889	-8.07227	6.90E-16	6.97E-14	FALSE	DOWN
PCDHB12	203.4855	-4.75087	0.893839	-5.31512	1.07E-07	2.09E-06	FALSE	DOWN
STYXL1	1025.1	-4.71555	0.420513	-11.2138	3.49E-29	1.34E-26	FALSE	DOWN
TMEM178	119.4512	-4.71193	1.224701	-3.84741	0.000119	0.000892	FALSE	DOWN
C1QL1	1300.866	-4.70458	1.124066	-4.18533	2.85E-05	0.000268	FALSE	DOWN
WFDC1	1563.698	-4.69484	1.259931	-3.72627	0.000194	0.00135	FALSE	DOWN
AASS	1211.683	-4.67975	0.5342	-8.76029	1.95E-18	2.52E-16	FALSE	DOWN
HRNR	68.47044	-4.67883	1.327236	-3.52524	0.000423	0.002578	FALSE	DOWN
MYL9	120169.5	-4.67221	1.143664	-4.0853	4.40E-05	0.000386	FALSE	DOWN
CD163	9408.869	-4.65319	0.97354	-4.77966	1.76E-06	2.43E-05	FALSE	DOWN
PPP1R14A	1814.979	-4.64624	1.110074	-4.18553	2.85E-05	0.000268	FALSE	DOWN
TCEAL7	586.8121	-4.64552	1.214761	-3.82422	0.000131	0.000965	FALSE	DOWN
VMO1	368.8375	-4.61373	1.055273	-4.37207	1.23E-05	0.000132	FALSE	DOWN
EFHC2	51.45274	-4.60964	1.445668	-3.18859	0.00143	0.007113	FALSE	DOWN
IDI2	141.1691	-4.59732	0.741568	-6.19946	5.67E-10	2.00E-08	FALSE	DOWN
NAGS	94.66468	-4.58	1.003129	-4.56571	4.98E-06	6.00E-05	FALSE	DOWN
MAPK13	388.3851	-4.56926	0.903817	-5.05551	4.29E-07	7.08E-06	FALSE	DOWN
PPP2R2B	542.2154	-4.55734	1.159696	-3.92977	8.50E-05	0.000673	FALSE	DOWN
CMTM1	238.075	-4.55666	0.645258	-7.06177	1.64E-12	9.60E-11	FALSE	DOWN
GPNMB	26360.35	-4.55443	0.854755	-5.32834	9.91E-08	1.95E-06	FALSE	DOWN
SUSD5	1972.817	-4.55376	1.290819	-3.52781	0.000419	0.002555	FALSE	DOWN
LRRC66	53.471	-4.54753	1.260213	-3.60854	0.000308	0.001983	FALSE	DOWN
FUT1	109.2731	-4.53638	0.948969	-4.78033	1.75E-06	2.43E-05	FALSE	DOWN
CCDC69	2777.111	-4.51943	0.870531	-5.19157	2.09E-07	3.74E-06	FALSE	DOWN
ATF7IP2	201.7661	-4.51109	0.954141	-4.72791	2.27E-06	3.01E-05	FALSE	DOWN
RIMKLB	1423.978	-4.50276	0.533818	-8.43501	3.31E-17	3.70E-15	FALSE	DOWN
RIMS4	486.045	-4.50122	1.437467	-3.13136	0.00174	0.008374	FALSE	DOWN
EPHX2	780.5782	-4.48449	0.894469	-5.01358	5.34E-07	8.57E-06	FALSE	DOWN
SLC40A1	6869.327	-4.48238	0.595273	-7.52996	5.08E-14	3.62E-12	FALSE	DOWN
NHLRC4	78.00145	-4.46927	1.121185	-3.9862	6.71E-05	0.000554	FALSE	DOWN
PLIN4	3192.664	-4.46904	1.374367	-3.25171	0.001147	0.005961	FALSE	DOWN
TAF7	5420.401	-4.46831	0.292523	-15.2751	1.12E-52	3.88E-49	FALSE	DOWN
RIMKLA	130.0119	-4.46739	1.230488	-3.63059	0.000283	0.001853	FALSE	DOWN
SLC47A1	802.9073	-4.46592	1.227489	-3.63826	0.000274	0.001806	FALSE	DOWN

FBXL7	3778.505	-4.45327	0.473263	-9.40971	4.98E-21	9.06E-19	FALSE	DOWN
HEY2	758.2984	-4.45107	1.066821	-4.17227	3.02E-05	0.000281	FALSE	DOWN
TSSK3	55.76646	-4.44762	1.104906	-4.02534	5.69E-05	0.00048	FALSE	DOWN
ADCY9	2918.708	-4.43577	0.432112	-10.2653	1.01E-24	2.69E-22	FALSE	DOWN
RPP25	1454.741	-4.42599	0.720277	-6.14485	8.00E-10	2.74E-08	FALSE	DOWN
BGLAP	150.0134	-4.42543	0.776695	-5.69777	1.21E-08	3.11E-07	FALSE	DOWN
CCDC80	34128.27	-4.42237	0.909223	-4.86391	1.15E-06	1.69E-05	FALSE	DOWN
KLHDC9	129.5046	-4.41546	1.138493	-3.87834	0.000105	0.000807	FALSE	DOWN
HFM1	54.06234	-4.41326	1.285335	-3.43355	0.000596	0.003466	FALSE	DOWN
SNRPN	4111.415	-4.4099	0.514523	-8.57085	1.03E-17	1.20E-15	FALSE	DOWN
VWF	20423.96	-4.39896	0.684971	-6.42211	1.34E-10	5.47E-09	FALSE	DOWN
HEPH	3944.364	-4.38561	1.152789	-3.80435	0.000142	0.001034	FALSE	DOWN
JAZF1	1915.186	-4.38024	0.593116	-7.38513	1.52E-13	1.04E-11	FALSE	DOWN
LONRF2	1776.883	-4.37756	1.163529	-3.76232	0.000168	0.001199	FALSE	DOWN
GNA14	586.0242	-4.35868	0.906168	-4.81002	1.51E-06	2.13E-05	FALSE	DOWN
CERCAM	12345.85	-4.34353	0.829945	-5.23351	1.66E-07	3.07E-06	FALSE	DOWN
PLB1	449.7012	-4.33128	0.770998	-5.61776	1.93E-08	4.63E-07	FALSE	DOWN
PRSS23	15217.49	-4.33118	0.63625	-6.80736	9.94E-12	4.91E-10	FALSE	DOWN
RARB	764.8779	-4.32973	0.841954	-5.14247	2.71E-07	4.69E-06	FALSE	DOWN
CCDC110	70.71822	-4.32918	1.133804	-3.81828	0.000134	0.000985	FALSE	DOWN
SORBS1	9965.329	-4.32896	1.280077	-3.38179	0.00072	0.004049	FALSE	DOWN
NTF3	375.9958	-4.32352	1.319225	-3.27732	0.001048	0.005542	FALSE	DOWN
RRAGB	953.958	-4.32217	0.489121	-8.83661	9.87E-19	1.34E-16	FALSE	DOWN
SLC35B1	1458.063	-4.31734	0.42708	-10.109	5.04E-24	1.29E-21	FALSE	DOWN
SORT1	7112.924	-4.31324	0.754025	-5.72029	1.06E-08	2.78E-07	FALSE	DOWN
HOXB7	770.9373	-4.2919	0.564459	-7.60356	2.88E-14	2.14E-12	FALSE	DOWN
CNTFR	1137.968	-4.28354	1.391202	-3.07902	0.002077	0.009737	FALSE	DOWN
PCDHB8	161.5811	-4.2827	0.977845	-4.37974	1.19E-05	0.000128	FALSE	DOWN
CD4	5158.452	-4.28261	0.854858	-5.00974	5.45E-07	8.71E-06	FALSE	DOWN
ALDH1A1	3670.825	-4.27531	1.10547	-3.86741	0.00011	0.000837	FALSE	DOWN
FCER1A	131.3695	-4.27081	1.230985	-3.46942	0.000522	0.003093	FALSE	DOWN
CYB5R2	329.3378	-4.26202	0.965292	-4.41527	1.01E-05	0.000111	FALSE	DOWN
MRVI1	7778.583	-4.25921	1.174149	-3.62749	0.000286	0.00187	FALSE	DOWN
CACNA1H	2092.274	-4.25392	1.305017	-3.25966	0.001115	0.00583	FALSE	DOWN
GIPC3	2182.971	-4.25187	1.023983	-4.15228	3.29E-05	0.000302	FALSE	DOWN
VSTM2L	309.1329	-4.25056	1.23223	-3.44948	0.000562	0.003303	FALSE	DOWN
CYFIP2	5505.592	-4.24697	1.179174	-3.60165	0.000316	0.002022	FALSE	DOWN
CHRDL1	6452.514	-4.24591	1.199985	-3.5383	0.000403	0.002476	FALSE	DOWN
USP11	7172.193	-4.2293	0.391716	-10.7969	3.56E-27	1.15E-24	FALSE	DOWN
AKAP12	10982.89	-4.21537	0.69091	-6.10118	1.05E-09	3.48E-08	FALSE	DOWN
ANKDD1A	764.6999	-4.20571	0.629897	-6.67682	2.44E-11	1.11E-09	FALSE	DOWN
CD36	8139.773	-4.20089	1.130218	-3.71689	0.000202	0.001393	FALSE	DOWN
LRRC3	211.5629	-4.17651	0.931298	-4.48461	7.30E-06	8.30E-05	FALSE	DOWN
SLC7A9	18.10326	-4.17495	1.357631	-3.07518	0.002104	0.009847	FALSE	DOWN
PPM1N	58.31124	-4.17025	1.201391	-3.47119	0.000518	0.003074	FALSE	DOWN
LRP3	3219.509	-4.15705	0.677914	-6.13212	8.67E-10	2.93E-08	FALSE	DOWN
SYNC	2891.907	-4.1555	0.750102	-5.53992	3.03E-08	6.86E-07	FALSE	DOWN
HRCT1	529.2049	-4.15538	0.998608	-4.16117	3.17E-05	0.000293	FALSE	DOWN

HIF3A	1406.648	-4.15527	1.220612	-3.40425	0.000663	0.00379	FALSE	DOWN
MMP24	521.5672	-4.15353	0.62423	-6.65384	2.86E-11	1.28E-09	FALSE	DOWN
ENPEP	2052.798	-4.15298	1.004818	-4.13307	3.58E-05	0.000323	FALSE	DOWN
SLC35F2	615.5557	-4.15291	0.805023	-5.15874	2.49E-07	4.35E-06	FALSE	DOWN
CCDC89	118.4969	-4.15181	0.702562	-5.90952	3.43E-09	9.93E-08	FALSE	DOWN
TCEAL6	297.5319	-4.14419	0.973302	-4.25786	2.06E-05	0.000205	FALSE	DOWN
ETV2	50.96277	-4.14371	1.002988	-4.13137	3.61E-05	0.000325	FALSE	DOWN
ST6GALNAC	753.8144	-4.14334	1.349292	-3.07075	0.002135	0.009974	FALSE	DOWN
A1BG	262.4401	-4.13988	0.868736	-4.76541	1.88E-06	2.58E-05	FALSE	DOWN
COLEC11	222.8021	-4.13442	1.315007	-3.14403	0.001666	0.008081	FALSE	DOWN
RNF112	1003.683	-4.1335	1.128169	-3.6639	0.000248	0.001665	FALSE	DOWN
TRIM29	115.3273	-4.12914	1.333241	-3.09707	0.001954	0.009258	FALSE	DOWN
CLU	83922.69	-4.12275	1.002044	-4.11434	3.88E-05	0.000347	FALSE	DOWN
RASL11A	795.0499	-4.11366	0.937717	-4.38689	1.15E-05	0.000124	FALSE	DOWN
CSRP1	76644.24	-4.11205	1.137082	-3.61632	0.000299	0.00194	FALSE	DOWN
PLA2G4C	1119.298	-4.10635	0.712745	-5.76132	8.35E-09	2.22E-07	FALSE	DOWN
PPARA	1198.822	-4.10133	0.489787	-8.3737	5.58E-17	5.99E-15	FALSE	DOWN
ANKRD36	361.3891	-4.09813	0.646407	-6.33986	2.30E-10	8.79E-09	FALSE	DOWN
ATP6AP1L	126.7043	-4.09454	0.685307	-5.97476	2.30E-09	7.01E-08	FALSE	DOWN
IFITM1	6635.03	-4.09347	0.749174	-5.46398	4.66E-08	1.01E-06	FALSE	DOWN
FN3K	714.2295	-4.07967	0.649501	-6.28123	3.36E-10	1.24E-08	FALSE	DOWN
SCUBE2	862.3037	-4.07954	1.007192	-4.05041	5.11E-05	0.000439	FALSE	DOWN
SENP5	1437.528	-4.07731	0.278381	-14.6465	1.42E-48	2.18E-45	FALSE	DOWN
STOX1	160.6581	-4.07311	0.997282	-4.08421	4.42E-05	0.000388	FALSE	DOWN
LRRN2	579.4595	-4.07295	1.108275	-3.67503	0.000238	0.001607	FALSE	DOWN
CYP26B1	1574.407	-4.06757	1.209129	-3.36405	0.000768	0.004275	FALSE	DOWN
NAP1L5	544.0755	-4.05986	0.605405	-6.70602	2.00E-11	9.25E-10	FALSE	DOWN
PRKDC	9119.809	-4.05923	0.4201	-9.66253	4.35E-22	8.98E-20	FALSE	DOWN
TEAD3	3783.838	-4.05909	0.663728	-6.11559	9.62E-10	3.22E-08	FALSE	DOWN
GDF5	302.5705	-4.05902	1.200791	-3.38029	0.000724	0.004069	FALSE	DOWN
TMEM102	135.1658	-4.05433	0.77215	-5.2507	1.52E-07	2.85E-06	FALSE	DOWN
EPHA4	1353.526	-4.04441	0.834475	-4.84666	1.26E-06	1.82E-05	FALSE	DOWN
CD46	5079.924	-4.04225	0.404329	-9.99741	1.56E-23	3.82E-21	FALSE	DOWN
PCDHB3	132.94	-4.0253	1.106144	-3.63904	0.000274	0.001802	FALSE	DOWN
SPON2	11426.83	-4.01299	1.100124	-3.64776	0.000265	0.001754	FALSE	DOWN
BCAM	3321.953	-4.00584	0.624439	-6.4151	1.41E-10	5.68E-09	FALSE	DOWN
TAF9B	1834.297	-3.99604	0.522367	-7.64987	2.01E-14	1.54E-12	FALSE	DOWN
FRAS1	1347.842	-3.99232	1.221294	-3.26893	0.00108	0.005686	FALSE	DOWN
EFCAB1	108.7515	-3.98899	1.249696	-3.19197	0.001413	0.007063	FALSE	DOWN
MDM2	29337.47	-3.98869	1.124711	-3.54642	0.000391	0.002411	FALSE	DOWN
RASL12	7694.329	-3.98632	1.266013	-3.14872	0.00164	0.00797	FALSE	DOWN
GIMAP5	2190.635	-3.98622	0.804957	-4.9521	7.34E-07	1.13E-05	FALSE	DOWN
EID3	238.1663	-3.97148	0.793009	-5.00812	5.50E-07	8.76E-06	FALSE	DOWN
INMT	804.1905	-3.94857	1.011556	-3.90346	9.48E-05	0.000735	FALSE	DOWN
PCDHB11	225.9506	-3.94786	1.124495	-3.51078	0.000447	0.002704	FALSE	DOWN
FRZB	932.8918	-3.94226	1.11677	-3.53006	0.000415	0.002537	FALSE	DOWN
ZP3	289.5654	-3.93675	0.723272	-5.44297	5.24E-08	1.12E-06	FALSE	DOWN
OPRL1	228.6752	-3.93391	0.724291	-5.4314	5.59E-08	1.18E-06	FALSE	DOWN

IGFBP5	73139.21	-3.92794	0.85183	-4.61118	4.00E-06	4.98E-05	FALSE	DOWN
EPAS1	14942.55	-3.92766	0.56377	-6.96678	3.24E-12	1.81E-10	FALSE	DOWN
TSPAN1	61.52573	-3.92619	1.140145	-3.44359	0.000574	0.00336	FALSE	DOWN
NXPH3	1041.602	-3.92131	0.983089	-3.98876	6.64E-05	0.00055	FALSE	DOWN
SEZ6L2	2628.989	-3.91791	1.151519	-3.40239	0.000668	0.003808	FALSE	DOWN
CNTN4	1105.828	-3.91073	1.080763	-3.61849	0.000296	0.001928	FALSE	DOWN
SUSD4	517.3618	-3.90725	1.190295	-3.28259	0.001029	0.005459	FALSE	DOWN
RSC1A1	159.8739	-3.89603	0.634271	-6.14254	8.12E-10	2.77E-08	FALSE	DOWN
RASD1	1071.706	-3.88778	1.04704	-3.71311	0.000205	0.001411	FALSE	DOWN
HSBP1L1	153.5691	-3.8874	0.846999	-4.58962	4.44E-06	5.44E-05	FALSE	DOWN
TMTC1	3055.142	-3.88359	0.712568	-5.45014	5.03E-08	1.08E-06	FALSE	DOWN
CHRNE	148.9755	-3.88017	1.065026	-3.64327	0.000269	0.00178	FALSE	DOWN
FAM13A	1758.777	-3.8768	0.460676	-8.41544	3.91E-17	4.30E-15	FALSE	DOWN
OS9	101924.4	-3.8711	1.064194	-3.63759	0.000275	0.00181	FALSE	DOWN
GPC6	4614.129	-3.87056	0.863266	-4.48362	7.34E-06	8.34E-05	FALSE	DOWN
MYOZ3	191.9975	-3.86429	1.126316	-3.43091	0.000602	0.00349	FALSE	DOWN
ELN	33651.04	-3.85813	1.121307	-3.44075	0.00058	0.00339	FALSE	DOWN
NRIP3	336.6058	-3.84413	0.806729	-4.76509	1.89E-06	2.58E-05	FALSE	DOWN
SLC19A3	138.7594	-3.83837	1.225694	-3.13159	0.001739	0.008373	FALSE	DOWN
TYMP	3715.11	-3.83824	0.760111	-5.04958	4.43E-07	7.27E-06	FALSE	DOWN
ACSM3	146.9915	-3.83671	1.01991	-3.76181	0.000169	0.001201	FALSE	DOWN
PHYHIP	819.2213	-3.83289	1.103306	-3.474	0.000513	0.003046	FALSE	DOWN
CEL	57.99677	-3.82654	1.037447	-3.68842	0.000226	0.001534	FALSE	DOWN
GLT8D2	1852.428	-3.82432	0.898409	-4.25677	2.07E-05	0.000206	FALSE	DOWN
CCDC13	56.27	-3.81058	1.068247	-3.56713	0.000361	0.002255	FALSE	DOWN
NGFR	1667.29	-3.80949	1.205925	-3.15898	0.001583	0.007734	FALSE	DOWN
CD55	3855.656	-3.80547	0.958848	-3.9688	7.22E-05	0.000589	FALSE	DOWN
GJD3	402.2856	-3.79944	1.0557	-3.59898	0.000319	0.002041	FALSE	DOWN
TNFRSF19	1871.816	-3.78898	0.997084	-3.80006	0.000145	0.001051	FALSE	DOWN
SETMAR	1413.654	-3.78252	0.689378	-5.48686	4.09E-08	8.94E-07	FALSE	DOWN
EPOR	707.1205	-3.77888	0.517199	-7.30643	2.74E-13	1.77E-11	FALSE	DOWN
LNX1	258.1735	-3.77717	0.767952	-4.91849	8.72E-07	1.31E-05	FALSE	DOWN
LDHB	13990.97	-3.77294	0.489993	-7.69999	1.36E-14	1.06E-12	FALSE	DOWN
SLC8A1	2877.449	-3.7712	0.8798	-4.28643	1.82E-05	0.000183	FALSE	DOWN
ITGB5	11524.09	-3.77053	0.54918	-6.86574	6.61E-12	3.38E-10	FALSE	DOWN
OPTN	5687.782	-3.75468	0.466986	-8.04023	8.97E-16	8.86E-14	FALSE	DOWN
SDS	184.4649	-3.75392	1.194949	-3.14149	0.001681	0.008137	FALSE	DOWN
FOXD1	887.9488	-3.75001	0.834231	-4.49517	6.95E-06	8.00E-05	FALSE	DOWN
CCIN	61.86195	-3.74638	1.112954	-3.36616	0.000762	0.004249	FALSE	DOWN
SVEP1	4058.525	-3.74608	0.997028	-3.75725	0.000172	0.001215	FALSE	DOWN
FABP3	4279.065	-3.73585	1.022878	-3.65229	0.00026	0.00173	FALSE	DOWN
ARNT2	1775.372	-3.72926	1.008107	-3.69928	0.000216	0.001481	FALSE	DOWN
LRRC24	53.19247	-3.72894	1.039792	-3.58624	0.000335	0.002125	FALSE	DOWN
TRPV1	352.6946	-3.72452	0.58689	-6.3462	2.21E-10	8.48E-09	FALSE	DOWN
LIMCH1	2876.415	-3.71551	0.67416	-5.51133	3.56E-08	7.95E-07	FALSE	DOWN
ANKRD53	111.5422	-3.69723	0.875437	-4.2233	2.41E-05	0.000235	FALSE	DOWN
EIF2B5	1828.751	-3.68526	0.265044	-13.9044	5.96E-44	6.34E-41	FALSE	DOWN
ACOT1	1469.25	-3.67675	0.691639	-5.316	1.06E-07	2.08E-06	FALSE	DOWN

BAALC	757.5074	-3.67583	1.055777	-3.48164	0.000498	0.002969	FALSE	DOWN
CADPS2	1611.85	-3.67427	0.643727	-5.70781	1.14E-08	2.96E-07	FALSE	DOWN
SPARCL1	39615.53	-3.6696	0.719136	-5.1028	3.35E-07	5.65E-06	FALSE	DOWN
SLC2A12	431.4767	-3.6682	0.868952	-4.22141	2.43E-05	0.000236	FALSE	DOWN
GGT1	349.5696	-3.66423	0.839954	-4.36241	1.29E-05	0.000136	FALSE	DOWN
PCDHB6	176.9963	-3.66413	1.13511	-3.228	0.001247	0.006386	FALSE	DOWN
NOTCH3	11364.31	-3.65359	0.607345	-6.01567	1.79E-09	5.59E-08	FALSE	DOWN
PCDH12	2622.503	-3.63068	0.80098	-4.5328	5.82E-06	6.90E-05	FALSE	DOWN
GSTK1	3722.851	-3.61055	0.391958	-9.21158	3.21E-20	5.17E-18	FALSE	DOWN
TFPI	3348.322	-3.61043	0.973601	-3.70833	0.000209	0.001435	FALSE	DOWN
RTN4R	274.1268	-3.60162	1.138719	-3.16287	0.001562	0.007646	FALSE	DOWN
SLC22A18	412.3873	-3.60058	0.755046	-4.76869	1.85E-06	2.55E-05	FALSE	DOWN
TUB	1785.189	-3.58754	0.818039	-4.38554	1.16E-05	0.000125	FALSE	DOWN
PTPRF	6030.355	-3.58707	0.781781	-4.58833	4.47E-06	5.46E-05	FALSE	DOWN
TMEM25	878.5323	-3.58442	0.886007	-4.04559	5.22E-05	0.000446	FALSE	DOWN
CDCP1	562.0379	-3.58372	1.038509	-3.45083	0.000559	0.003288	FALSE	DOWN
CLEC14A	1992.554	-3.5788	0.649618	-5.50908	3.61E-08	8.02E-07	FALSE	DOWN
CELSR3	549.4954	-3.5737	0.984693	-3.62925	0.000284	0.00186	FALSE	DOWN
QRICH2	218.059	-3.57349	0.730857	-4.88945	1.01E-06	1.50E-05	FALSE	DOWN
COL12A1	23584.98	-3.57021	0.956849	-3.73122	0.000191	0.00133	FALSE	DOWN
GPR3	207.0332	-3.56974	0.871259	-4.09722	4.18E-05	0.00037	FALSE	DOWN
MAPK10	1096.075	-3.56375	1.096992	-3.24866	0.00116	0.006011	FALSE	DOWN
ASB9	91.66768	-3.56119	0.869703	-4.09472	4.23E-05	0.000373	FALSE	DOWN
C1RL	2524.497	-3.56064	0.565117	-6.30072	2.96E-10	1.11E-08	FALSE	DOWN
ABHD3	666.8399	-3.55967	0.428943	-8.2987	1.05E-16	1.12E-14	FALSE	DOWN
CCDC92	2531.819	-3.5548	0.364418	-9.75473	1.76E-22	3.81E-20	FALSE	DOWN
MESP1	100.1131	-3.54712	0.96497	-3.67588	0.000237	0.001603	FALSE	DOWN
MEGF6	1240.303	-3.54458	0.944127	-3.75435	0.000174	0.001227	FALSE	DOWN
BTBD6	3761.808	-3.54435	0.377926	-9.37841	6.70E-21	1.20E-18	FALSE	DOWN
UPK3BL	308.5581	-3.53471	1.033032	-3.42169	0.000622	0.003588	FALSE	DOWN
PLD6	312.6609	-3.52518	0.667383	-5.2821	1.28E-07	2.44E-06	FALSE	DOWN
THSD4	3376.173	-3.50389	0.883938	-3.96396	7.37E-05	0.000597	FALSE	DOWN
SLC25A29	1364.187	-3.4932	0.503899	-6.93234	4.14E-12	2.21E-10	FALSE	DOWN
PELI3	726.1978	-3.48808	0.54004	-6.45893	1.05E-10	4.34E-09	FALSE	DOWN
GCA	741.9157	-3.48389	0.542476	-6.42221	1.34E-10	5.47E-09	FALSE	DOWN
MCAM	19114.8	-3.47091	0.797386	-4.35286	1.34E-05	0.000141	FALSE	DOWN
SNPH	685.1865	-3.4661	0.757679	-4.57464	4.77E-06	5.78E-05	FALSE	DOWN
FAM89A	499.1524	-3.46579	0.8995	-3.85301	0.000117	0.000876	FALSE	DOWN
PTAR1	1919.308	-3.45804	0.359136	-9.62877	6.04E-22	1.23E-19	FALSE	DOWN
ANKRD12	1695.924	-3.45573	0.359669	-9.60809	7.39E-22	1.46E-19	FALSE	DOWN
MAMDC2	1992.397	-3.44766	1.030623	-3.34522	0.000822	0.004538	FALSE	DOWN
MX2	1334.166	-3.44658	0.794133	-4.34005	1.42E-05	0.000148	FALSE	DOWN
HDHD3	700.9465	-3.44647	0.682972	-5.04629	4.50E-07	7.37E-06	FALSE	DOWN
SOX13	1956.803	-3.43659	0.728371	-4.71818	2.38E-06	3.15E-05	FALSE	DOWN
F8	1100.677	-3.43359	0.563098	-6.09767	1.08E-09	3.53E-08	FALSE	DOWN
NPAS2	1194.236	-3.43345	0.635812	-5.4001	6.66E-08	1.38E-06	FALSE	DOWN
WASF3	654.3697	-3.42429	1.005326	-3.40615	0.000659	0.003767	FALSE	DOWN
C1QTNF1	3977.002	-3.41965	0.852473	-4.01145	6.03E-05	0.000506	FALSE	DOWN

PXMP2	633.7989	-3.41755	0.577181	-5.92111	3.20E-09	9.37E-08	FALSE	DOWN
TAF9	2630.987	-3.41286	0.29864	-11.428	3.03E-30	1.27E-27	FALSE	DOWN
RARRES2	5354.487	-3.40808	1.017458	-3.3496	0.000809	0.004475	FALSE	DOWN
FAM184A	635.7083	-3.40203	1.033609	-3.29141	0.000997	0.005329	FALSE	DOWN
BBS10	1020.332	-3.38643	0.48291	-7.01254	2.34E-12	1.33E-10	FALSE	DOWN
POPDC2	1488.62	-3.38477	1.099189	-3.07934	0.002075	0.00973	FALSE	DOWN
SLC4A5	511.3345	-3.37888	0.600042	-5.63106	1.79E-08	4.36E-07	FALSE	DOWN
RPL27	23711.68	-3.37321	0.388105	-8.69148	3.58E-18	4.46E-16	FALSE	DOWN
KRT222	154.07	-3.3683	0.875311	-3.84812	0.000119	0.000891	FALSE	DOWN
RAVER2	824.0699	-3.36687	0.65731	-5.12219	3.02E-07	5.17E-06	FALSE	DOWN
H1FX	6596.077	-3.36497	0.374924	-8.97508	2.83E-19	4.04E-17	FALSE	DOWN
STK38	3130.884	-3.36396	0.355635	-9.45903	3.11E-21	5.73E-19	FALSE	DOWN
AGMAT	46.30105	-3.36008	1.091992	-3.07702	0.002091	0.009793	FALSE	DOWN
NECAB3	1341.093	-3.35918	0.499433	-6.726	1.74E-11	8.15E-10	FALSE	DOWN
HSPA13	2450.09	-3.35883	0.420433	-7.98898	1.36E-15	1.30E-13	FALSE	DOWN
PRH1	92.54933	-3.35427	0.774193	-4.3326	1.47E-05	0.000153	FALSE	DOWN
SEMA3G	1245.506	-3.35278	0.670438	-5.00088	5.71E-07	9.09E-06	FALSE	DOWN
YWHAQ	13739.13	-3.35257	0.236056	-14.2025	8.84E-46	1.11E-42	FALSE	DOWN
LARP6	2479.531	-3.33484	0.494925	-6.73807	1.61E-11	7.55E-10	FALSE	DOWN
DPYD	1351.442	-3.3291	0.515242	-6.46124	1.04E-10	4.29E-09	FALSE	DOWN
TMEM108	1177.308	-3.32506	1.02621	-3.24014	0.001195	0.006166	FALSE	DOWN
RAI2	1004.403	-3.32483	0.969265	-3.43026	0.000603	0.003497	FALSE	DOWN
ZCCHC3	1542.127	-3.32212	0.482115	-6.89072	5.55E-12	2.86E-10	FALSE	DOWN
NGRN	7311.518	-3.32179	0.302954	-10.9647	5.65E-28	2.00E-25	FALSE	DOWN
PCDHB9	342.5511	-3.32009	0.892483	-3.72006	0.000199	0.001379	FALSE	DOWN
RBPMS	9032.254	-3.31866	0.842032	-3.94125	8.11E-05	0.000646	FALSE	DOWN
ZDHHC23	142.8413	-3.31442	0.968869	-3.42092	0.000624	0.003595	FALSE	DOWN
ITIH5	3511.393	-3.30929	0.975443	-3.39261	0.000692	0.003926	FALSE	DOWN
CNTNAP1	2678.629	-3.30819	0.588423	-5.62213	1.89E-08	4.53E-07	FALSE	DOWN
FBXO7	4611.735	-3.30074	0.250222	-13.1912	9.86E-40	7.18E-37	FALSE	DOWN
CLDN23	169.8395	-3.29292	0.810534	-4.06266	4.85E-05	0.000421	FALSE	DOWN
LZTS1	1231.85	-3.28674	0.725362	-4.53117	5.87E-06	6.92E-05	FALSE	DOWN
PTPRN2	1054.847	-3.28673	0.92475	-3.55419	0.000379	0.002352	FALSE	DOWN
SPATS2L	6939.973	-3.27492	0.439698	-7.4481	9.47E-14	6.55E-12	FALSE	DOWN
ATP8B3	262.3635	-3.27431	0.754029	-4.34243	1.41E-05	0.000147	FALSE	DOWN
SNCA	401.6218	-3.27405	0.921865	-3.55155	0.000383	0.002372	FALSE	DOWN
GJA4	1236.327	-3.27182	0.752325	-4.34895	1.37E-05	0.000143	FALSE	DOWN
SCRN1	4018.088	-3.26721	0.856752	-3.81348	0.000137	0.001	FALSE	DOWN
CMBL	1631.413	-3.26717	0.726933	-4.49446	6.97E-06	8.02E-05	FALSE	DOWN
ADAMTS13	286.3447	-3.26183	0.695867	-4.68743	2.77E-06	3.60E-05	FALSE	DOWN
TSPAN18	4809.539	-3.2551	0.593559	-5.48404	4.16E-08	9.07E-07	FALSE	DOWN
FXYD6	5087.608	-3.25306	0.723048	-4.4991	6.82E-06	7.87E-05	FALSE	DOWN
PPARG	1195.083	-3.2476	0.951654	-3.41258	0.000644	0.003691	FALSE	DOWN
BDH1	437.6551	-3.24734	0.984478	-3.29854	0.000972	0.005232	FALSE	DOWN
KCNS3	600.031	-3.24481	0.877643	-3.69718	0.000218	0.001492	FALSE	DOWN
FGF13	897.7684	-3.2445	0.739943	-4.3848	1.16E-05	0.000125	FALSE	DOWN
CDK18	1339.198	-3.24329	0.935075	-3.46848	0.000523	0.003103	FALSE	DOWN
AMPH	872.5157	-3.23752	0.986488	-3.28187	0.001031	0.005468	FALSE	DOWN

GALNT12	1514.022	-3.23637	0.903066	-3.58376	0.000339	0.002142	FALSE	DOWN
COL9A2	664.0142	-3.23496	1.045155	-3.09519	0.001967	0.009301	FALSE	DOWN
KLF15	712.328	-3.23481	1.037009	-3.11936	0.001812	0.008671	FALSE	DOWN
RPUSD3	1016.01	-3.23245	0.309269	-10.4519	1.44E-25	4.06E-23	FALSE	DOWN
HIST1H2AC	1236.408	-3.23075	0.804242	-4.01713	5.89E-05	0.000496	FALSE	DOWN
PDK4	11588.73	-3.22266	0.97275	-3.31294	0.000923	0.005001	FALSE	DOWN
NPR1	4156.709	-3.22107	0.752921	-4.27809	1.89E-05	0.000189	FALSE	DOWN
CHST3	2901.468	-3.21844	0.520954	-6.17797	6.49E-10	2.26E-08	FALSE	DOWN
PLCXD1	1303.25	-3.21373	0.671344	-4.78701	1.69E-06	2.36E-05	FALSE	DOWN
F2RL3	463.3241	-3.19555	0.982973	-3.2509	0.00115	0.005973	FALSE	DOWN
KLF12	1497.66	-3.19331	0.453424	-7.04266	1.89E-12	1.09E-10	FALSE	DOWN
MOAP1	1516.778	-3.18764	0.539294	-5.91076	3.41E-09	9.88E-08	FALSE	DOWN
EGFLAM	1212.556	-3.18667	0.858558	-3.71165	0.000206	0.001418	FALSE	DOWN
PAPLN	1199.417	-3.17942	0.895232	-3.55151	0.000383	0.002372	FALSE	DOWN
FHL1	24856.59	-3.16457	0.942329	-3.35824	0.000784	0.004353	FALSE	DOWN
MANSC1	1029.08	-3.15713	0.492333	-6.4126	1.43E-10	5.75E-09	FALSE	DOWN
TMEM8B	1229.633	-3.151	0.434015	-7.26012	3.87E-13	2.48E-11	FALSE	DOWN
ETNK2	1037.656	-3.15072	0.843307	-3.73615	0.000187	0.001308	FALSE	DOWN
FAHD2A	675.3937	-3.14993	0.371941	-8.46891	2.48E-17	2.79E-15	FALSE	DOWN
DLG4	1223.067	-3.14223	0.454104	-6.91962	4.53E-12	2.38E-10	FALSE	DOWN
FLYWCH2	1332.717	-3.14149	0.40189	-7.81678	5.42E-15	4.49E-13	FALSE	DOWN
PCBD1	1391.545	-3.13204	0.492031	-6.36552	1.95E-10	7.56E-09	FALSE	DOWN
PCDHB7	486.7195	-3.12808	0.902975	-3.4642	0.000532	0.00315	FALSE	DOWN
KBTBD6	662.9079	-3.12741	0.529081	-5.91102	3.40E-09	9.88E-08	FALSE	DOWN
TNFSF10	2318.739	-3.12654	0.765772	-4.08286	4.45E-05	0.00039	FALSE	DOWN
MALL	1071.63	-3.12309	0.670705	-4.65643	3.22E-06	4.12E-05	FALSE	DOWN
MYO5C	412.6663	-3.11905	0.768037	-4.06107	4.88E-05	0.000422	FALSE	DOWN
APLP1	1170.139	-3.11579	0.930859	-3.34722	0.000816	0.00451	FALSE	DOWN
LRRC34	150.5199	-3.10988	0.658066	-4.72578	2.29E-06	3.04E-05	FALSE	DOWN
ATP6V1E2	139.0187	-3.10751	0.601854	-5.16323	2.43E-07	4.26E-06	FALSE	DOWN
RAB33A	216.9007	-3.10004	0.957948	-3.23612	0.001212	0.00623	FALSE	DOWN
LGALS3BP	27067.08	-3.09971	0.687054	-4.5116	6.43E-06	7.50E-05	FALSE	DOWN
NAALADL1	230.7858	-3.09934	0.819027	-3.78418	0.000154	0.001112	FALSE	DOWN
APOLD1	4110.519	-3.09875	0.660557	-4.69111	2.72E-06	3.54E-05	FALSE	DOWN
CIRBP	11243.65	-3.09011	0.451958	-6.83715	8.08E-12	4.08E-10	FALSE	DOWN
COL8A2	4952.177	-3.08794	0.977527	-3.15893	0.001584	0.007734	FALSE	DOWN
NECAB1	1107.925	-3.08625	0.818546	-3.77041	0.000163	0.001165	FALSE	DOWN
RNF208	175.8742	-3.0753	0.840061	-3.6608	0.000251	0.001683	FALSE	DOWN
PRDM6	316.6832	-3.07217	0.849091	-3.61819	0.000297	0.001929	FALSE	DOWN
PDE5A	3741.308	-3.06704	0.924249	-3.31841	0.000905	0.004928	FALSE	DOWN
RPS12	24271.68	-3.06315	0.605086	-5.06234	4.14E-07	6.86E-06	FALSE	DOWN
PLXNB1	3098.24	-3.05945	0.810261	-3.77588	0.000159	0.001144	FALSE	DOWN
FILIP1	1227.566	-3.05555	0.792584	-3.85517	0.000116	0.00087	FALSE	DOWN
FGD5	3091.441	-3.05424	0.853919	-3.57673	0.000348	0.002189	FALSE	DOWN
TMEM56	804.8114	-3.05396	0.986918	-3.09445	0.001972	0.009318	FALSE	DOWN
SPOCK2	2409.172	-3.05229	0.881312	-3.46335	0.000533	0.003158	FALSE	DOWN
ABLIM3	2515.736	-3.05227	0.656999	-4.64577	3.39E-06	4.31E-05	FALSE	DOWN
MGLL	6693.402	-3.0506	0.498849	-6.11529	9.64E-10	3.22E-08	FALSE	DOWN

COL6A2	162762	-3.04811	0.636861	-4.78616	1.70E-06	2.36E-05	FALSE	DOWN
LAMA5	7153.739	-3.03708	0.789584	-3.84643	0.00012	0.000894	FALSE	DOWN
KANK1	3944.71	-3.03624	0.801877	-3.78642	0.000153	0.001104	FALSE	DOWN
SYTL2	1976.746	-3.03561	0.789841	-3.84332	0.000121	0.000904	FALSE	DOWN
NNMT	10988	-3.0323	0.905547	-3.34858	0.000812	0.00449	FALSE	DOWN
PSMF1	5806.671	-3.03078	0.249393	-12.1526	5.56E-34	2.85E-31	FALSE	DOWN
IL17RB	100.9812	-3.02945	0.759261	-3.99	6.61E-05	0.000547	FALSE	DOWN
EFHD1	1294.84	-3.02703	0.88202	-3.43193	0.000599	0.00348	FALSE	DOWN
RECK	2130.343	-3.01854	0.58589	-5.15205	2.58E-07	4.48E-06	FALSE	DOWN
CCDC103	138.4622	-3.01828	0.710303	-4.24928	2.14E-05	0.000212	FALSE	DOWN
MFAP4	49302.08	-3.01784	0.831388	-3.62988	0.000284	0.001857	FALSE	DOWN
RNF125	368.8015	-3.01304	0.675755	-4.45878	8.24E-06	9.21E-05	FALSE	DOWN
L3MBTL4	254.4395	-3.0094	0.909429	-3.30911	0.000936	0.005065	FALSE	DOWN
FADS2	4963.383	-3.00893	0.641698	-4.68901	2.75E-06	3.57E-05	FALSE	DOWN
TCEAL1	2275.032	-3.00622	0.853152	-3.52366	0.000426	0.002591	FALSE	DOWN
P4HTM	1346.724	-3.00548	0.379022	-7.92957	2.20E-15	2.01E-13	FALSE	DOWN
EFS	2858	-3.00369	0.641953	-4.67899	2.88E-06	3.74E-05	FALSE	DOWN
MYL6B	1926.08	-3.00286	0.544904	-5.5108	3.57E-08	7.96E-07	FALSE	DOWN
RPL5	34390.76	-2.99387	0.414569	-7.22165	5.14E-13	3.22E-11	FALSE	DOWN
PPP1R3B	4784.494	-2.99165	0.856153	-3.4943	0.000475	0.002851	FALSE	DOWN
LYRM7	1026.404	-2.9858	0.377869	-7.90168	2.75E-15	2.43E-13	FALSE	DOWN
NIPSNAP1	1542.723	-2.98216	0.511513	-5.83008	5.54E-09	1.54E-07	FALSE	DOWN
PRRT3	256.7402	-2.98085	0.555006	-5.37085	7.84E-08	1.58E-06	FALSE	DOWN
OPN3	493.0565	-2.978	0.608859	-4.89111	1.00E-06	1.49E-05	FALSE	DOWN
BRPF3	1591.262	-2.97648	0.437132	-6.8091	9.82E-12	4.87E-10	FALSE	DOWN
KCNE4	2129.972	-2.9717	0.879505	-3.37884	0.000728	0.004088	FALSE	DOWN
CCDC14	1446.245	-2.96987	0.507407	-5.85303	4.83E-09	1.36E-07	FALSE	DOWN
REC8	323.2691	-2.9697	0.894367	-3.32045	0.000899	0.004899	FALSE	DOWN
RBM4	3945.347	-2.96573	0.297556	-9.96696	2.13E-23	4.99E-21	FALSE	DOWN
PLEKHG4	1430.442	-2.96305	0.727439	-4.07327	4.64E-05	0.000404	FALSE	DOWN
KLHL4	771.0821	-2.96027	0.792556	-3.7351	0.000188	0.001312	FALSE	DOWN
FBLN1	56554.01	-2.9509	0.819157	-3.60237	0.000315	0.002019	FALSE	DOWN
WFS1	4124.934	-2.94932	0.551919	-5.34375	9.10E-08	1.81E-06	FALSE	DOWN
PYGL	2782.938	-2.94139	0.53497	-5.49823	3.84E-08	8.47E-07	FALSE	DOWN
ARG2	580.9736	-2.94112	0.867764	-3.38931	0.000701	0.003959	FALSE	DOWN
SH3BP5	5708.622	-2.93856	0.593942	-4.94756	7.52E-07	1.15E-05	FALSE	DOWN
LPAR5	243.0914	-2.93419	0.930143	-3.15456	0.001607	0.007845	FALSE	DOWN
SCN4B	1224.664	-2.93299	0.910199	-3.22236	0.001271	0.006479	FALSE	DOWN
DAAM2	2554.943	-2.93265	0.734097	-3.99491	6.47E-05	0.000537	FALSE	DOWN
GAL3ST4	1530.392	-2.92986	0.554692	-5.28195	1.28E-07	2.44E-06	FALSE	DOWN
LRP11	2919.67	-2.92266	0.537731	-5.43517	5.47E-08	1.16E-06	FALSE	DOWN
SMPD4	4175.789	-2.91799	0.331352	-8.8063	1.29E-18	1.72E-16	FALSE	DOWN
S1PR3	5448.269	-2.91766	0.741	-3.93747	8.23E-05	0.000654	FALSE	DOWN
NFE2L3	497.9833	-2.91665	0.734609	-3.97034	7.18E-05	0.000586	FALSE	DOWN
ARHGAP20	739.9715	-2.90852	0.898459	-3.23723	0.001207	0.006213	FALSE	DOWN
CD276	11408.41	-2.90711	0.581553	-4.99888	5.77E-07	9.17E-06	FALSE	DOWN
SLC45A1	260.777	-2.90144	0.623532	-4.65323	3.27E-06	4.18E-05	FALSE	DOWN
FAM20C	6157.736	-2.9	0.639258	-4.53651	5.72E-06	6.80E-05	FALSE	DOWN

MRGPRF	4779.458	-2.89392	0.911999	-3.17317	0.001508	0.007414	FALSE	DOWN
ALKBH7	1672.191	-2.89342	0.394197	-7.34005	2.14E-13	1.42E-11	FALSE	DOWN
STOM	17982.6	-2.88165	0.434035	-6.63921	3.15E-11	1.39E-09	FALSE	DOWN
FBXO41	260.2062	-2.87831	0.879632	-3.27217	0.001067	0.005636	FALSE	DOWN
AKAP3	77.92095	-2.87208	0.881516	-3.25812	0.001122	0.005856	FALSE	DOWN
PPCS	1754.324	-2.86609	0.297962	-9.61899	6.65E-22	1.33E-19	FALSE	DOWN
BBS1	1561.413	-2.86449	0.482356	-5.93853	2.88E-09	8.61E-08	FALSE	DOWN
PRKG1	395.7961	-2.86293	0.840262	-3.40718	0.000656	0.003759	FALSE	DOWN
THRB	884.02	-2.85952	0.728599	-3.92469	8.68E-05	0.000684	FALSE	DOWN
TST	734.9577	-2.85893	0.629599	-4.54088	5.60E-06	6.68E-05	FALSE	DOWN
KCNIP3	909.9621	-2.85862	0.799319	-3.57631	0.000348	0.002192	FALSE	DOWN
KANK2	28699.61	-2.85649	0.662089	-4.31435	1.60E-05	0.000164	FALSE	DOWN
ADAR	12479.89	-2.85503	0.322436	-8.85458	8.40E-19	1.15E-16	FALSE	DOWN
FAU	15223.26	-2.85283	0.387364	-7.36472	1.78E-13	1.19E-11	FALSE	DOWN
GSTA4	1046.716	-2.83862	0.358795	-7.91153	2.54E-15	2.28E-13	FALSE	DOWN
NCALD	1416.931	-2.82343	0.738874	-3.82127	0.000133	0.000976	FALSE	DOWN
PIK3R3	2425.987	-2.82069	0.355	-7.9456	1.93E-15	1.79E-13	FALSE	DOWN
TSPYL5	1328.953	-2.80644	0.829615	-3.38282	0.000717	0.004037	FALSE	DOWN
GRHPR	3567.729	-2.80583	0.319182	-8.79068	1.49E-18	1.94E-16	FALSE	DOWN
FHIT	86.64575	-2.80204	0.860125	-3.25771	0.001123	0.005858	FALSE	DOWN
GUK1	8614.305	-2.79335	0.36118	-7.73395	1.04E-14	8.39E-13	FALSE	DOWN
APOL6	3480.013	-2.78567	0.61781	-4.50894	6.52E-06	7.58E-05	FALSE	DOWN
NME4	4694.245	-2.78543	0.487503	-5.71366	1.11E-08	2.88E-07	FALSE	DOWN
GGT7	948.9999	-2.77941	0.548656	-5.06585	4.07E-07	6.75E-06	FALSE	DOWN
RNF144A	2377.993	-2.778	0.807615	-3.43976	0.000582	0.003399	FALSE	DOWN
TSPAN15	1862.342	-2.77596	0.635072	-4.3711	1.24E-05	0.000132	FALSE	DOWN
SPAG8	51.89972	-2.77102	0.77873	-3.55838	0.000373	0.002319	FALSE	DOWN
B4GALT4	1747.355	-2.76905	0.383164	-7.22682	4.94E-13	3.12E-11	FALSE	DOWN
OSBPL10	1554.469	-2.76856	0.737905	-3.75192	0.000175	0.001237	FALSE	DOWN
THAP8	424.46	-2.7642	0.46733	-5.91489	3.32E-09	9.69E-08	FALSE	DOWN
PEMT	712.0555	-2.76414	0.458022	-6.03495	1.59E-09	5.01E-08	FALSE	DOWN
PLLP	122.0193	-2.75828	0.789601	-3.49325	0.000477	0.002859	FALSE	DOWN
XPC	2816.719	-2.75552	0.401778	-6.85833	6.97E-12	3.53E-10	FALSE	DOWN
SVIP	443.2338	-2.7521	0.614108	-4.48146	7.41E-06	8.41E-05	FALSE	DOWN
TSPYL4	1815.091	-2.75189	0.487096	-5.64958	1.61E-08	3.99E-07	FALSE	DOWN
ASPRV1	210.3211	-2.74755	0.598383	-4.59163	4.40E-06	5.41E-05	FALSE	DOWN
LDB2	1703.966	-2.74456	0.629277	-4.36145	1.29E-05	0.000137	FALSE	DOWN
LRFN3	837.767	-2.74317	0.395931	-6.92841	4.26E-12	2.26E-10	FALSE	DOWN
SLC41A3	2411.406	-2.74213	0.30236	-9.06908	1.20E-19	1.79E-17	FALSE	DOWN
ASTN2	704.2242	-2.73864	0.827965	-3.30768	0.000941	0.005084	FALSE	DOWN
BOC	4610.506	-2.7299	0.797053	-3.42499	0.000615	0.003556	FALSE	DOWN
AADAT	182.8977	-2.72656	0.590248	-4.61935	3.85E-06	4.81E-05	FALSE	DOWN
MGST3	4919.64	-2.72576	0.486755	-5.59986	2.15E-08	5.08E-07	FALSE	DOWN
SLC16A14	463.3761	-2.72483	0.714056	-3.81599	0.000136	0.000992	FALSE	DOWN
SPRN	198.376	-2.72286	0.615047	-4.42708	9.55E-06	0.000105	FALSE	DOWN
FBXO44	752.2249	-2.72023	0.55259	-4.9227	8.54E-07	1.29E-05	FALSE	DOWN
ACSS2	1833.885	-2.72017	0.458772	-5.92924	3.04E-09	9.05E-08	FALSE	DOWN
HSD17B1	185.033	-2.71808	0.733076	-3.70777	0.000209	0.001436	FALSE	DOWN

HEY1	738.3391	-2.71598	0.77192	-3.51847	0.000434	0.002634	FALSE	DOWN
RAD23A	9149.531	-2.71583	0.351401	-7.72857	1.09E-14	8.70E-13	FALSE	DOWN
ACTR3B	246.6188	-2.71364	0.62355	-4.35192	1.35E-05	0.000141	FALSE	DOWN
CCDC96	120.7067	-2.71167	0.557167	-4.8669	1.13E-06	1.67E-05	FALSE	DOWN
HEBP2	1722.243	-2.71071	0.604445	-4.48463	7.30E-06	8.30E-05	FALSE	DOWN
ACSL1	3459.056	-2.71017	0.631323	-4.29284	1.76E-05	0.000179	FALSE	DOWN
IL4I1	586.2435	-2.70945	0.82251	-3.29413	0.000987	0.005297	FALSE	DOWN
SMARCA1	2758.618	-2.6949	0.478874	-5.62757	1.83E-08	4.43E-07	FALSE	DOWN
SLC46A1	1420.04	-2.69179	0.422489	-6.37126	1.87E-10	7.31E-09	FALSE	DOWN
SPINT2	1428.389	-2.68926	0.843368	-3.18871	0.001429	0.007113	FALSE	DOWN
FZD4	2830.435	-2.68707	0.543768	-4.94158	7.75E-07	1.18E-05	FALSE	DOWN
SPIN4	723.0771	-2.68429	0.61069	-4.39551	1.11E-05	0.00012	FALSE	DOWN
ARL4D	1460.888	-2.68366	0.652506	-4.11284	3.91E-05	0.000349	FALSE	DOWN
RCAN2	1632.483	-2.67705	0.86884	-3.08118	0.002062	0.009683	FALSE	DOWN
KLHL29	800.106	-2.66865	0.758948	-3.51624	0.000438	0.002654	FALSE	DOWN
NUDT15	694.2431	-2.66831	0.363975	-7.33101	2.28E-13	1.49E-11	FALSE	DOWN
ATP9A	3547.305	-2.6661	0.699105	-3.81359	0.000137	0.001	FALSE	DOWN
IFNGR2	4571.509	-2.66379	0.48386	-5.50529	3.69E-08	8.17E-07	FALSE	DOWN
OAS3	3199.484	-2.66293	0.632104	-4.2128	2.52E-05	0.000244	FALSE	DOWN
CLDN15	633.0267	-2.65965	0.458107	-5.80573	6.41E-09	1.75E-07	FALSE	DOWN
LEPR	926.5787	-2.65896	0.758759	-3.50435	0.000458	0.002766	FALSE	DOWN
CREG1	7323.301	-2.65728	0.553228	-4.80323	1.56E-06	2.18E-05	FALSE	DOWN
CABLES1	1340.967	-2.63652	0.653717	-4.03312	5.50E-05	0.000468	FALSE	DOWN
MX1	4584.05	-2.63426	0.771408	-3.41487	0.000638	0.003664	FALSE	DOWN
ITPK1	3980.397	-2.63086	0.5558	-4.73346	2.21E-06	2.94E-05	FALSE	DOWN
CTSF	4091.371	-2.62706	0.545749	-4.81368	1.48E-06	2.10E-05	FALSE	DOWN
FAM122B	1394.493	-2.62602	0.395532	-6.6392	3.15E-11	1.39E-09	FALSE	DOWN
KRT10	1252.644	-2.62488	0.419016	-6.26439	3.74E-10	1.37E-08	FALSE	DOWN
MSC	1489.509	-2.61517	0.789056	-3.3143	0.000919	0.004989	FALSE	DOWN
HSD17B14	599.5371	-2.6136	0.747748	-3.4953	0.000474	0.002844	FALSE	DOWN
CHST10	1128.194	-2.60752	0.546655	-4.76996	1.84E-06	2.54E-05	FALSE	DOWN
GBE1	3337.347	-2.60514	0.543886	-4.78986	1.67E-06	2.33E-05	FALSE	DOWN
RPH3AL	331.8782	-2.60307	0.695014	-3.74534	0.00018	0.001266	FALSE	DOWN
ROR1	690.1173	-2.60282	0.770279	-3.37906	0.000727	0.004086	FALSE	DOWN
TBXA2R	870.121	-2.60001	0.7137	-3.643	0.000269	0.001781	FALSE	DOWN
MAMLD1	824.8229	-2.59962	0.71791	-3.62109	0.000293	0.001911	FALSE	DOWN
PPFIBP2	830.234	-2.59911	0.614607	-4.2289	2.35E-05	0.00023	FALSE	DOWN
COX7A1	1605.722	-2.59497	0.837669	-3.09784	0.001949	0.00924	FALSE	DOWN
NUDT3	978.0133	-2.59175	0.384355	-6.7431	1.55E-11	7.32E-10	FALSE	DOWN
PGBD1	368.3099	-2.58837	0.417076	-6.20598	5.44E-10	1.94E-08	FALSE	DOWN
STIM1	4132.304	-2.5865	0.362876	-7.12778	1.02E-12	6.06E-11	FALSE	DOWN
CREB3L4	506.6795	-2.58613	0.474039	-5.45551	4.88E-08	1.06E-06	FALSE	DOWN
TRIM14	1160.511	-2.57023	0.639269	-4.02058	5.81E-05	0.000489	FALSE	DOWN
VCL	20290.43	-2.56367	0.634803	-4.03852	5.38E-05	0.000458	FALSE	DOWN
LYPLAL1	830.6703	-2.56018	0.464886	-5.50712	3.65E-08	8.10E-07	FALSE	DOWN
SARM1	1522.951	-2.55719	0.628495	-4.06874	4.73E-05	0.000411	FALSE	DOWN
ATP5G2	8619.028	-2.55551	0.378722	-6.74771	1.50E-11	7.14E-10	FALSE	DOWN
EPB41L4A	587.9866	-2.55542	0.470727	-5.42867	5.68E-08	1.20E-06	FALSE	DOWN

SLC37A1	639.8534	-2.55525	0.557586	-4.58271	4.59E-06	5.59E-05	FALSE	DOWN
SRGAP1	1050.395	-2.55315	0.607316	-4.20399	2.62E-05	0.000252	FALSE	DOWN
EFCAB7	381.6225	-2.54726	0.508579	-5.00859	5.48E-07	8.75E-06	FALSE	DOWN
TIMP3	41010.69	-2.5459	0.730431	-3.48548	0.000491	0.002932	FALSE	DOWN
LRRC23	407.9558	-2.54503	0.533136	-4.77369	1.81E-06	2.50E-05	FALSE	DOWN
UBTD2	2394.048	-2.54426	0.45112	-5.63987	1.70E-08	4.18E-07	FALSE	DOWN
VASH1	2654.303	-2.54058	0.628206	-4.04419	5.25E-05	0.000448	FALSE	DOWN
STK32C	398.0813	-2.53451	0.564487	-4.48993	7.12E-06	8.14E-05	FALSE	DOWN
SHPK	694.9267	-2.53197	0.619239	-4.08884	4.34E-05	0.000381	FALSE	DOWN
SLC10A3	2334.687	-2.52997	0.488343	-5.18072	2.21E-07	3.93E-06	FALSE	DOWN
COQ4	1572.547	-2.52983	0.489011	-5.17337	2.30E-07	4.07E-06	FALSE	DOWN
HAAO	624.1688	-2.52696	0.75613	-3.34197	0.000832	0.004584	FALSE	DOWN
XRCC5	11208.83	-2.52337	0.264384	-9.54435	1.37E-21	2.56E-19	FALSE	DOWN
CLSTN1	12833.33	-2.52287	0.317525	-7.94543	1.94E-15	1.79E-13	FALSE	DOWN
NQO1	1314.602	-2.52274	0.743961	-3.39096	0.000696	0.003939	FALSE	DOWN
ESRP2	368.6201	-2.52253	0.480407	-5.25082	1.51E-07	2.85E-06	FALSE	DOWN
ABHD15	838.84	-2.5152	0.374816	-6.71048	1.94E-11	9.01E-10	FALSE	DOWN
HSPB1	30305.21	-2.51491	0.674908	-3.7263	0.000194	0.00135	FALSE	DOWN
ST5	6081.317	-2.50426	0.559807	-4.47343	7.70E-06	8.68E-05	FALSE	DOWN
SELENBP1	3429.639	-2.49435	0.706614	-3.53	0.000416	0.002537	FALSE	DOWN
RAB40B	619.391	-2.49349	0.509924	-4.88992	1.01E-06	1.50E-05	FALSE	DOWN
MAVS	4863.481	-2.48944	0.318645	-7.81256	5.60E-15	4.62E-13	FALSE	DOWN
NFATC2IP	1556.261	-2.48804	0.47734	-5.2123	1.87E-07	3.40E-06	FALSE	DOWN
RBMX	5566.019	-2.48525	0.227985	-10.9009	1.14E-27	3.85E-25	FALSE	DOWN
HOXB2	936.2458	-2.4827	0.533797	-4.65102	3.30E-06	4.22E-05	FALSE	DOWN
NUMA1	18851.27	-2.48222	0.395365	-6.27831	3.42E-10	1.26E-08	FALSE	DOWN
WDR34	2990.889	-2.48115	0.470511	-5.27332	1.34E-07	2.55E-06	FALSE	DOWN
TERF2IP	3981.179	-2.47467	0.307475	-8.04836	8.39E-16	8.35E-14	FALSE	DOWN
PLEKHA4	3021.985	-2.4738	0.627408	-3.94288	8.05E-05	0.000642	FALSE	DOWN
IKZF4	587.4681	-2.47312	0.616143	-4.01388	5.97E-05	0.000502	FALSE	DOWN
ATPAF1	2230.639	-2.47196	0.534139	-4.62792	3.69E-06	4.63E-05	FALSE	DOWN
CCDC149	1291.221	-2.46557	0.362228	-6.80668	9.99E-12	4.92E-10	FALSE	DOWN
ANGEL1	835.7658	-2.46115	0.339558	-7.24808	4.23E-13	2.69E-11	FALSE	DOWN
ARHGAP1	11306.43	-2.45873	0.414768	-5.92795	3.07E-09	9.09E-08	FALSE	DOWN
EFNB1	5857.563	-2.4541	0.75084	-3.26847	0.001081	0.005691	FALSE	DOWN
GLUL	27889.69	-2.45356	0.619104	-3.96308	7.40E-05	0.000598	FALSE	DOWN
SLC2A10	1763.729	-2.45346	0.67801	-3.61862	0.000296	0.001928	FALSE	DOWN
GPR4	753.8718	-2.45026	0.67194	-3.64655	0.000266	0.00176	FALSE	DOWN
ARVCF	883.0094	-2.44467	0.64763	-3.77479	0.00016	0.001148	FALSE	DOWN
EPHX1	5853.747	-2.44388	0.558737	-4.37393	1.22E-05	0.000131	FALSE	DOWN
GPRIN1	462.7511	-2.41537	0.744332	-3.24501	0.001174	0.006078	FALSE	DOWN
ODF3B	349.1	-2.40836	0.784329	-3.07061	0.002136	0.009975	FALSE	DOWN
DIXDC1	2536.322	-2.40705	0.75922	-3.17042	0.001522	0.007482	FALSE	DOWN
GNAI1	1786.258	-2.40647	0.6745	-3.56778	0.00036	0.002251	FALSE	DOWN
NEURL1B	6013.681	-2.40602	0.731096	-3.29097	0.000998	0.005336	FALSE	DOWN
FAM198B	7258.384	-2.40101	0.77677	-3.09102	0.001995	0.009416	FALSE	DOWN
PRPS2	4257.807	-2.39902	0.734305	-3.26706	0.001087	0.00571	FALSE	DOWN
SUMF2	5938.131	-2.39869	0.303287	-7.90897	2.60E-15	2.30E-13	FALSE	DOWN

SPRY3	146.7367	-2.3969	0.621824	-3.85462	0.000116	0.000871	FALSE	DOWN
KIF7	1181.946	-2.39686	0.480276	-4.99059	6.02E-07	9.52E-06	FALSE	DOWN
NID2	5480.108	-2.3935	0.643259	-3.72089	0.000199	0.001375	FALSE	DOWN
ECM2	2054.187	-2.38977	0.772156	-3.09493	0.001969	0.009306	FALSE	DOWN
ZKSCAN4	289.4952	-2.38702	0.364721	-6.5448	5.96E-11	2.56E-09	FALSE	DOWN
ICAM2	1503.354	-2.38154	0.661088	-3.60245	0.000315	0.002019	FALSE	DOWN
EXOSC6	525.458	-2.37891	0.492783	-4.8275	1.38E-06	1.98E-05	FALSE	DOWN
RAB23	7420.609	-2.37551	0.722319	-3.28873	0.001006	0.005364	FALSE	DOWN
ARHGEF9	1803.518	-2.37159	0.564653	-4.20008	2.67E-05	0.000256	FALSE	DOWN
LYRM2	1548.592	-2.3714	0.273024	-8.6857	3.76E-18	4.65E-16	FALSE	DOWN
TMEM14A	822.6151	-2.37084	0.411614	-5.75986	8.42E-09	2.23E-07	FALSE	DOWN
NDUFAF3	2514.206	-2.37069	0.353845	-6.6998	2.09E-11	9.56E-10	FALSE	DOWN
ADNP	4138.868	-2.36663	0.369464	-6.40559	1.50E-10	5.92E-09	FALSE	DOWN
SCARB2	14933.94	-2.36601	0.299142	-7.90933	2.59E-15	2.30E-13	FALSE	DOWN
MAGED2	19126.18	-2.36414	0.471782	-5.01109	5.41E-07	8.66E-06	FALSE	DOWN
NAT14	798.1263	-2.36057	0.53238	-4.43399	9.25E-06	0.000102	FALSE	DOWN
CHSY3	579.3125	-2.35456	0.711974	-3.30709	0.000943	0.005091	FALSE	DOWN
HIPK2	2492.962	-2.34175	0.601326	-3.89431	9.85E-05	0.00076	FALSE	DOWN
DDX60	1550.658	-2.33859	0.502669	-4.65234	3.28E-06	4.20E-05	FALSE	DOWN
CALML4	933.2709	-2.33827	0.370612	-6.30921	2.80E-10	1.06E-08	FALSE	DOWN
TNFAIP3	2810.748	-2.33573	0.731052	-3.19503	0.001398	0.007007	FALSE	DOWN
LMCD1	3292.652	-2.3335	0.683142	-3.41583	0.000636	0.003654	FALSE	DOWN
TMEM220	306.887	-2.32868	0.505734	-4.60455	4.13E-06	5.12E-05	FALSE	DOWN
FAM131A	1243.834	-2.32689	0.488676	-4.76162	1.92E-06	2.62E-05	FALSE	DOWN
KLF9	6250.413	-2.31898	0.578913	-4.00575	6.18E-05	0.000516	FALSE	DOWN
CALHM2	2629.704	-2.3167	0.527517	-4.39169	1.12E-05	0.000122	FALSE	DOWN
PDGFRB	17779.05	-2.31665	0.587113	-3.94584	7.95E-05	0.000636	FALSE	DOWN
LMNB2	6524.169	-2.3147	0.517722	-4.47093	7.79E-06	8.76E-05	FALSE	DOWN
TUBG2	965.5526	-2.31397	0.487014	-4.75134	2.02E-06	2.74E-05	FALSE	DOWN
CABYR	257.3272	-2.30884	0.660852	-3.49373	0.000476	0.002855	FALSE	DOWN
TMEM79	363.2769	-2.30757	0.409723	-5.63203	1.78E-08	4.35E-07	FALSE	DOWN
ICA1L	468.7482	-2.30588	0.684831	-3.36709	0.00076	0.004237	FALSE	DOWN
MMRN2	2702.415	-2.30195	0.554082	-4.15454	3.26E-05	0.0003	FALSE	DOWN
SLC43A1	880.3683	-2.29958	0.493105	-4.66347	3.11E-06	4.01E-05	FALSE	DOWN
CCDC142	364.9809	-2.29554	0.410631	-5.59028	2.27E-08	5.33E-07	FALSE	DOWN
HESX1	35.07271	-2.29427	0.730799	-3.1394	0.001693	0.008184	FALSE	DOWN
LARP1B	615.8144	-2.29182	0.388319	-5.90191	3.59E-09	1.03E-07	FALSE	DOWN
ENTPD6	2750.551	-2.29065	0.392044	-5.84284	5.13E-09	1.43E-07	FALSE	DOWN
ABHD14B	2697.125	-2.29005	0.424166	-5.39896	6.70E-08	1.38E-06	FALSE	DOWN
NPHP4	682.3078	-2.29003	0.506685	-4.51965	6.19E-06	7.26E-05	FALSE	DOWN
MED29	3690.427	-2.28951	0.290854	-7.87167	3.50E-15	2.97E-13	FALSE	DOWN
STAT5B	5494.399	-2.28843	0.483369	-4.73433	2.20E-06	2.94E-05	FALSE	DOWN
TXNIP	36392.06	-2.28723	0.553869	-4.12955	3.63E-05	0.000327	FALSE	DOWN
URB1	2469.528	-2.28689	0.524759	-4.35798	1.31E-05	0.000139	FALSE	DOWN
JAM3	6138.826	-2.28621	0.549266	-4.16229	3.15E-05	0.000292	FALSE	DOWN
TIMP1	38349.63	-2.28436	0.737704	-3.09658	0.001958	0.009267	FALSE	DOWN
MID2	997.8681	-2.27882	0.593982	-3.83651	0.000125	0.000926	FALSE	DOWN
NCOA1	2929.162	-2.27635	0.399414	-5.69924	1.20E-08	3.09E-07	FALSE	DOWN

ACSS1	1366.372	-2.27328	0.501667	-4.53146	5.86E-06	6.92E-05	FALSE	DOWN
UNC13B	1650.323	-2.26739	0.447184	-5.07038	3.97E-07	6.60E-06	FALSE	DOWN
SLC25A43	739.9299	-2.25532	0.530565	-4.2508	2.13E-05	0.000211	FALSE	DOWN
KCNC4	424.6497	-2.25407	0.59757	-3.77206	0.000162	0.001159	FALSE	DOWN
SGCD	2682.682	-2.25381	0.700824	-3.21594	0.0013	0.006592	FALSE	DOWN
MSRB2	1718.81	-2.25002	0.541577	-4.15457	3.26E-05	0.0003	FALSE	DOWN
ILVBL	3647.735	-2.24475	0.361702	-6.20607	5.43E-10	1.94E-08	FALSE	DOWN
PGAP3	1407.908	-2.24366	0.366864	-6.11579	9.61E-10	3.22E-08	FALSE	DOWN
RWDD2B	905.9667	-2.2383	0.36962	-6.05566	1.40E-09	4.46E-08	FALSE	DOWN
CRY2	2402.878	-2.23808	0.391862	-5.7114	1.12E-08	2.91E-07	FALSE	DOWN
FAM102A	4000.25	-2.23448	0.432097	-5.17125	2.33E-07	4.10E-06	FALSE	DOWN
PVRIG	154.6555	-2.23292	0.714513	-3.1251	0.001777	0.008527	FALSE	DOWN
FBXW5	5958.411	-2.23115	0.335149	-6.65718	2.79E-11	1.26E-09	FALSE	DOWN
FUCA2	5062.377	-2.22537	0.683715	-3.25482	0.001135	0.005913	FALSE	DOWN
GIMAP7	757.438	-2.22437	0.680764	-3.26746	0.001085	0.005705	FALSE	DOWN
B3GALT6	1533.735	-2.22321	0.535659	-4.15041	3.32E-05	0.000304	FALSE	DOWN
HOXB3	908.3007	-2.22258	0.519317	-4.27982	1.87E-05	0.000188	FALSE	DOWN
TMUB1	1613.006	-2.21952	0.370775	-5.98617	2.15E-09	6.58E-08	FALSE	DOWN
SLC27A3	2886.976	-2.21798	0.495893	-4.4727	7.72E-06	8.70E-05	FALSE	DOWN
ADI1	3839.044	-2.21695	0.423264	-5.23775	1.63E-07	3.02E-06	FALSE	DOWN
ANKS6	1211.417	-2.21254	0.406169	-5.44733	5.11E-08	1.10E-06	FALSE	DOWN
LMBR1	1558.039	-2.20353	0.394066	-5.59179	2.25E-08	5.30E-07	FALSE	DOWN
FAS	1596.836	-2.20168	0.492833	-4.46739	7.92E-06	8.88E-05	FALSE	DOWN
COL4A2	81700.84	-2.19816	0.612911	-3.58643	0.000335	0.002125	FALSE	DOWN
LIN7B	143.895	-2.19682	0.601967	-3.6494	0.000263	0.001746	FALSE	DOWN
PHF19	3136.188	-2.19597	0.531476	-4.13184	3.60E-05	0.000325	FALSE	DOWN
BET1L	3861.939	-2.19216	0.29748	-7.36908	1.72E-13	1.16E-11	FALSE	DOWN
PLSCR4	1842.76	-2.19097	0.578695	-3.78605	0.000153	0.001105	FALSE	DOWN
USP22	12898.5	-2.18591	0.441248	-4.95393	7.27E-07	1.12E-05	FALSE	DOWN
DCUN1D4	2040.675	-2.18298	0.428298	-5.09688	3.45E-07	5.83E-06	FALSE	DOWN
PRKD1	1451.701	-2.18291	0.618325	-3.53037	0.000415	0.002537	FALSE	DOWN
GAS6	16113.49	-2.18154	0.689625	-3.16336	0.00156	0.007636	FALSE	DOWN
BTG2	4619.669	-2.18151	0.557704	-3.91159	9.17E-05	0.000715	FALSE	DOWN
TACO1	862.2629	-2.1802	0.320122	-6.81054	9.72E-12	4.84E-10	FALSE	DOWN
SNX29	1514.437	-2.17885	0.410437	-5.30861	1.10E-07	2.14E-06	FALSE	DOWN
RAB27A	1362.952	-2.17815	0.589408	-3.69549	0.000219	0.0015	FALSE	DOWN
XRCC6	12220.48	-2.17699	0.269185	-8.08734	6.10E-16	6.25E-14	FALSE	DOWN
TYW3	1135.063	-2.17689	0.34721	-6.26969	3.62E-10	1.33E-08	FALSE	DOWN
HDDC2	2034.536	-2.17581	0.415167	-5.2408	1.60E-07	2.98E-06	FALSE	DOWN
BCAP29	2772.719	-2.17477	0.270205	-8.0486	8.37E-16	8.35E-14	FALSE	DOWN
SPAG16	718.2135	-2.17422	0.55906	-3.88907	0.000101	0.000775	FALSE	DOWN
RPL10	21524.83	-2.17352	0.498992	-4.35582	1.33E-05	0.000139	FALSE	DOWN
ERBB2	3362.508	-2.1735	0.459468	-4.73048	2.24E-06	2.98E-05	FALSE	DOWN
TTC3	9806.468	-2.17219	0.443573	-4.89702	9.73E-07	1.45E-05	FALSE	DOWN
ZGLP1	98.31234	-2.17194	0.63413	-3.42507	0.000615	0.003556	FALSE	DOWN
PER1	8347.046	-2.1714	0.596388	-3.64092	0.000272	0.001793	FALSE	DOWN
GYPC	5178.267	-2.17068	0.588428	-3.68894	0.000225	0.001532	FALSE	DOWN
SAT2	1982.655	-2.16737	0.522286	-4.14977	3.33E-05	0.000305	FALSE	DOWN

PCDHB14	666.8394	-2.16165	0.693923	-3.11511	0.001839	0.008785	FALSE	DOWN
ESAM	3111.967	-2.15367	0.596703	-3.60928	0.000307	0.00198	FALSE	DOWN
SGCB	4769.282	-2.15212	0.484319	-4.4436	8.85E-06	9.80E-05	FALSE	DOWN
CCDC51	1071.573	-2.1397	0.660472	-3.23966	0.001197	0.006174	FALSE	DOWN
PDE2A	1073.937	-2.13467	0.681895	-3.13049	0.001745	0.008387	FALSE	DOWN
TMEM219	4038.718	-2.13217	0.372813	-5.71915	1.07E-08	2.79E-07	FALSE	DOWN
DENND3	1426.856	-2.13185	0.515099	-4.13873	3.49E-05	0.000317	FALSE	DOWN
ZFP36	9044.9	-2.12991	0.582953	-3.65366	0.000259	0.001725	FALSE	DOWN
CNP	5722.476	-2.12511	0.244869	-8.67856	4.01E-18	4.91E-16	FALSE	DOWN
HOXA4	255.5492	-2.11727	0.623814	-3.39407	0.000689	0.003908	FALSE	DOWN
GSPT2	617.1475	-2.11348	0.418369	-5.05171	4.38E-07	7.21E-06	FALSE	DOWN
MPZL1	8655.956	-2.11039	0.64087	-3.29301	0.000991	0.005307	FALSE	DOWN
SEC61G	2464.528	-2.10753	0.433941	-4.85672	1.19E-06	1.75E-05	FALSE	DOWN
ANTXR1	12708.67	-2.10543	0.677126	-3.10936	0.001875	0.008936	FALSE	DOWN
SAP18	4579.07	-2.10409	0.304403	-6.91218	4.77E-12	2.49E-10	FALSE	DOWN
RAB42	140.5825	-2.10404	0.652343	-3.22536	0.001258	0.00643	FALSE	DOWN
KCTD1	803.9485	-2.10139	0.485227	-4.33075	1.49E-05	0.000154	FALSE	DOWN
JAG1	5222.087	-2.10056	0.442366	-4.74846	2.05E-06	2.76E-05	FALSE	DOWN
SGSH	2274.508	-2.0995	0.387141	-5.4231	5.86E-08	1.23E-06	FALSE	DOWN
GALM	1308.218	-2.09909	0.577682	-3.63364	0.000279	0.001833	FALSE	DOWN
RCAN1	3655.749	-2.09446	0.498722	-4.19966	2.67E-05	0.000256	FALSE	DOWN
EMILIN1	27729.03	-2.09446	0.676437	-3.09632	0.001959	0.009272	FALSE	DOWN
AHR	3710.042	-2.09441	0.677894	-3.08959	0.002004	0.009449	FALSE	DOWN
CYP2R1	337.9031	-2.09389	0.427833	-4.89417	9.87E-07	1.47E-05	FALSE	DOWN
WARS	9270.813	-2.0906	0.646379	-3.23433	0.001219	0.00626	FALSE	DOWN
SLC22A17	2161.649	-2.09051	0.64986	-3.21685	0.001296	0.006578	FALSE	DOWN
RHBDD2	3254.446	-2.08903	0.519573	-4.02066	5.80E-05	0.000489	FALSE	DOWN
ILF3	12459.14	-2.08627	0.291364	-7.16035	8.05E-13	4.84E-11	FALSE	DOWN
CRTC3	4603.052	-2.08377	0.393463	-5.29597	1.18E-07	2.28E-06	FALSE	DOWN
PYGB	12594.89	-2.08184	0.633752	-3.28494	0.00102	0.005422	FALSE	DOWN
UXT	2444.158	-2.07805	0.408207	-5.09068	3.57E-07	5.99E-06	FALSE	DOWN
SYTL4	1434.759	-2.07704	0.562689	-3.69128	0.000223	0.00152	FALSE	DOWN
RETSAT	2398.563	-2.07535	0.375172	-5.53173	3.17E-08	7.16E-07	FALSE	DOWN
PPDPF	7976.071	-2.07469	0.427227	-4.85617	1.20E-06	1.75E-05	FALSE	DOWN
SCO2	1162.756	-2.07354	0.441168	-4.7001	2.60E-06	3.41E-05	FALSE	DOWN
PDPR	1593.874	-2.07214	0.613437	-3.37793	0.00073	0.004098	FALSE	DOWN
ADAMTS1	10502.36	-2.06623	0.628231	-3.28896	0.001006	0.005362	FALSE	DOWN
ELAC2	3909.887	-2.06587	0.482021	-4.28584	1.82E-05	0.000184	FALSE	DOWN
HS1BP3	2598.099	-2.06304	0.468869	-4.40003	1.08E-05	0.000118	FALSE	DOWN
LAMA4	18386.02	-2.06065	0.609903	-3.37865	0.000728	0.004089	FALSE	DOWN
WDR1	29566.67	-2.05536	0.580902	-3.53822	0.000403	0.002476	FALSE	DOWN
SUOX	1084.208	-2.05486	0.453207	-4.53404	5.79E-06	6.87E-05	FALSE	DOWN
KCNMB3	121.4823	-2.05065	0.549516	-3.73174	0.00019	0.001327	FALSE	DOWN
PEX10	922.6616	-2.04995	0.35815	-5.72371	1.04E-08	2.73E-07	FALSE	DOWN
MAD2L2	1884.289	-2.04837	0.472728	-4.33309	1.47E-05	0.000152	FALSE	DOWN
PHACTR2	2020.296	-2.03853	0.656079	-3.10714	0.001889	0.009001	FALSE	DOWN
DDX24	7770.955	-2.03141	0.293723	-6.91608	4.64E-12	2.43E-10	FALSE	DOWN
CHD1L	1812.879	-2.03117	0.364122	-5.57828	2.43E-08	5.67E-07	FALSE	DOWN

NEK11	318.2696	-2.03062	0.462664	-4.38897	1.14E-05	0.000123	FALSE	DOWN
GBA2	2281.722	-2.03014	0.347403	-5.84374	5.10E-09	1.43E-07	FALSE	DOWN
MKKS	1897.225	-2.02964	0.303993	-6.67659	2.45E-11	1.11E-09	FALSE	DOWN
ITGB1BP1	2620.865	-2.02891	0.283997	-7.14414	9.06E-13	5.42E-11	FALSE	DOWN
PRICKLE2	2011.72	-2.02656	0.451654	-4.48697	7.22E-06	8.24E-05	FALSE	DOWN
10-Sep	3818.199	-2.02645	0.461626	-4.38981	1.13E-05	0.000123	FALSE	DOWN
BTBD2	5659.104	-2.026	0.337237	-6.00764	1.88E-09	5.83E-08	FALSE	DOWN
MORN2	412.2983	-2.02598	0.500569	-4.04734	5.18E-05	0.000444	FALSE	DOWN
DPH1	1290.895	-2.02565	0.3499	-5.78922	7.07E-09	1.90E-07	FALSE	DOWN
LYSMD4	952.1939	-2.02526	0.468953	-4.31868	1.57E-05	0.000161	FALSE	DOWN
RAB4A	1265.54	-2.02336	0.306625	-6.59883	4.14E-11	1.82E-09	FALSE	DOWN
MPPE1	1288.613	-2.01904	0.317636	-6.35645	2.06E-10	7.96E-09	FALSE	DOWN
CCDC106	704.7478	-2.01856	0.439206	-4.59592	4.31E-06	5.30E-05	FALSE	DOWN
TCFL5	719.0308	-2.01804	0.519231	-3.88659	0.000102	0.000781	FALSE	DOWN
ECHDC1	2562.151	-2.01729	0.437538	-4.61056	4.02E-06	4.99E-05	FALSE	DOWN
TNIP1	5913.533	-2.01364	0.267054	-7.54018	4.69E-14	3.36E-12	FALSE	DOWN
PACS1	6143.977	-2.01301	0.321681	-6.25779	3.90E-10	1.42E-08	FALSE	DOWN
SLC39A8	1702.688	-2.0113	0.638067	-3.15218	0.001621	0.007898	FALSE	DOWN
TRMT61A	1844.415	-2.00657	0.461674	-4.34628	1.38E-05	0.000144	FALSE	DOWN
ZBED5	1805.301	-2.00225	0.332751	-6.01727	1.77E-09	5.55E-08	FALSE	DOWN
TLE4	1817.804	-1.99451	0.458216	-4.35278	1.34E-05	0.000141	FALSE	DOWN
PRDX6	15048.32	-1.99357	0.502881	-3.9643	7.36E-05	0.000597	FALSE	DOWN
SLC5A6	978.1993	-1.98934	0.451746	-4.40366	1.06E-05	0.000116	FALSE	DOWN
TMEM129	2833.559	-1.98574	0.369081	-5.38023	7.44E-08	1.51E-06	FALSE	DOWN
DTD1	921.2383	-1.98329	0.488312	-4.06151	4.88E-05	0.000422	FALSE	DOWN
SLC43A3	4206.679	-1.98309	0.489229	-4.05349	5.05E-05	0.000435	FALSE	DOWN
CASP7	1757.812	-1.98235	0.39776	-4.98379	6.24E-07	9.80E-06	FALSE	DOWN
NUDT16	1643.809	-1.97758	0.341423	-5.79218	6.95E-09	1.88E-07	FALSE	DOWN
FOXRED2	1853.987	-1.97709	0.358231	-5.51905	3.41E-08	7.64E-07	FALSE	DOWN
ZBTB8A	997.4446	-1.9715	0.362913	-5.43244	5.56E-08	1.18E-06	FALSE	DOWN
RPL10A	17014.98	-1.97022	0.378893	-5.19994	1.99E-07	3.61E-06	FALSE	DOWN
SNX22	539.5932	-1.96739	0.481425	-4.0866	4.38E-05	0.000384	FALSE	DOWN
EXOC7	7494.076	-1.96595	0.242805	-8.0968	5.64E-16	5.83E-14	FALSE	DOWN
PML	5498.356	-1.96557	0.305819	-6.42724	1.30E-10	5.32E-09	FALSE	DOWN
PLXNA2	2624.378	-1.96491	0.546826	-3.5933	0.000327	0.002077	FALSE	DOWN
ZYX	22926.55	-1.9641	0.498879	-3.93702	8.25E-05	0.000655	FALSE	DOWN
TMEM203	2208.968	-1.96045	0.316392	-6.19625	5.78E-10	2.03E-08	FALSE	DOWN
H2AFV	9310.924	-1.95958	0.348049	-5.6302	1.80E-08	4.38E-07	FALSE	DOWN
ACACB	1524.8	-1.95336	0.593457	-3.2915	0.000997	0.005329	FALSE	DOWN
ICMT	5970.25	-1.95084	0.316666	-6.16055	7.25E-10	2.51E-08	FALSE	DOWN
ACVR2B	411.3661	-1.94433	0.546808	-3.55579	0.000377	0.002339	FALSE	DOWN
ANKAR	67.82181	-1.9434	0.600287	-3.23745	0.001206	0.00621	FALSE	DOWN
NCOA7	2325.16	-1.94061	0.521866	-3.71859	0.0002	0.001385	FALSE	DOWN
PMP22	11612.36	-1.93639	0.557448	-3.47367	0.000513	0.003049	FALSE	DOWN
NME3	1702.23	-1.93607	0.431705	-4.48472	7.30E-06	8.30E-05	FALSE	DOWN
NAPEPLD	785.3413	-1.93566	0.38547	-5.02157	5.13E-07	8.26E-06	FALSE	DOWN
NT5DC1	804.4744	-1.93376	0.469199	-4.1214	3.77E-05	0.000338	FALSE	DOWN
VWA1	3985.928	-1.93355	0.59848	-3.23077	0.001235	0.006331	FALSE	DOWN

TMEM116	381.0713	-1.93296	0.536332	-3.60405	0.000313	0.00201	FALSE	DOWN
CDC25B	5008.69	-1.93262	0.49466	-3.90697	9.35E-05	0.000728	FALSE	DOWN
LPCAT1	5808.151	-1.932	0.605771	-3.18933	0.001426	0.007104	FALSE	DOWN
CDK5RAP1	898.3252	-1.93174	0.256174	-7.54072	4.67E-14	3.36E-12	FALSE	DOWN
DCHS1	7461.046	-1.92709	0.566344	-3.40268	0.000667	0.003806	FALSE	DOWN
CYYR1	995.629	-1.92566	0.603966	-3.18836	0.001431	0.007113	FALSE	DOWN
AGRN	5871.377	-1.92223	0.601512	-3.19566	0.001395	0.006994	FALSE	DOWN
TECPR2	2528.762	-1.91787	0.452012	-4.24297	2.21E-05	0.000217	FALSE	DOWN
SDR39U1	1564.156	-1.91578	0.351627	-5.44832	5.08E-08	1.09E-06	FALSE	DOWN
CIB1	3578.735	-1.91074	0.403719	-4.73285	2.21E-06	2.95E-05	FALSE	DOWN
CASC4	6291.006	-1.90779	0.354341	-5.38405	7.28E-08	1.49E-06	FALSE	DOWN
LPIN1	1973.277	-1.90612	0.369916	-5.15283	2.57E-07	4.47E-06	FALSE	DOWN
WDR6	7045.196	-1.90552	0.400383	-4.75924	1.94E-06	2.65E-05	FALSE	DOWN
FAM53B	2122.079	-1.90417	0.497588	-3.8268	0.00013	0.000958	FALSE	DOWN
REPIN1	2708.287	-1.9018	0.30677	-6.19942	5.67E-10	2.00E-08	FALSE	DOWN
TBC1D1	6177.897	-1.89872	0.506755	-3.74682	0.000179	0.001259	FALSE	DOWN
PER2	1149.005	-1.8936	0.56461	-3.35382	0.000797	0.004415	FALSE	DOWN
MXRA7	14572.04	-1.89306	0.569066	-3.3266	0.000879	0.004807	FALSE	DOWN
RPGR	289.5421	-1.8921	0.467403	-4.04811	5.16E-05	0.000443	FALSE	DOWN
TRIM62	485.0148	-1.88965	0.393958	-4.79657	1.61E-06	2.25E-05	FALSE	DOWN
TUFM	6930.661	-1.8803	0.256277	-7.33697	2.18E-13	1.45E-11	FALSE	DOWN
JRK	870.58	-1.88008	0.54418	-3.45488	0.000551	0.003248	FALSE	DOWN
PQLC1	2618.957	-1.87915	0.337697	-5.56458	2.63E-08	6.08E-07	FALSE	DOWN
HINT3	1461.984	-1.87899	0.389782	-4.82061	1.43E-06	2.04E-05	FALSE	DOWN
MEGF9	1632.688	-1.8737	0.446906	-4.19261	2.76E-05	0.000262	FALSE	DOWN
DCTD	3325.448	-1.87164	0.367258	-5.09626	3.46E-07	5.83E-06	FALSE	DOWN
TM2D3	1931.07	-1.87145	0.239014	-7.82985	4.88E-15	4.07E-13	FALSE	DOWN
ZSWIM1	583.9408	-1.87009	0.288276	-6.48716	8.75E-11	3.66E-09	FALSE	DOWN
PDK2	2368.072	-1.86832	0.446233	-4.18687	2.83E-05	0.000267	FALSE	DOWN
FAM120C	741.2093	-1.86569	0.45969	-4.05858	4.94E-05	0.000426	FALSE	DOWN
SEPHS2	2667.169	-1.86391	0.34403	-5.41787	6.03E-08	1.26E-06	FALSE	DOWN
PCDHGC3	12425.31	-1.86112	0.534835	-3.4798	0.000502	0.002986	FALSE	DOWN
SIX5	2162.89	-1.85777	0.333982	-5.56247	2.66E-08	6.13E-07	FALSE	DOWN
RSAD1	1723.707	-1.85596	0.291648	-6.36372	1.97E-10	7.63E-09	FALSE	DOWN
AGBL5	1226.824	-1.85337	0.486304	-3.81113	0.000138	0.001009	FALSE	DOWN
FAM71D	48.84865	-1.85267	0.498169	-3.71896	0.0002	0.001383	FALSE	DOWN
GALNT11	2568.961	-1.85074	0.41208	-4.49121	7.08E-06	8.12E-05	FALSE	DOWN
TMEM120E	1047.649	-1.84872	0.574225	-3.2195	0.001284	0.00653	FALSE	DOWN
SUSD1	572.9196	-1.84658	0.599565	-3.07987	0.002071	0.009721	FALSE	DOWN
LRRC8A	6124.734	-1.84327	0.391213	-4.71167	2.46E-06	3.24E-05	FALSE	DOWN
SLC12A7	4061.589	-1.84083	0.57983	-3.17478	0.001499	0.007383	FALSE	DOWN
ALG3	1635.628	-1.83646	0.329171	-5.57905	2.42E-08	5.65E-07	FALSE	DOWN
RBM12	2854.681	-1.8303	0.275221	-6.6503	2.93E-11	1.31E-09	FALSE	DOWN
MBD4	1419.476	-1.82594	0.230645	-7.91665	2.44E-15	2.21E-13	FALSE	DOWN
SNAI2	3405.846	-1.81753	0.576462	-3.15291	0.001617	0.007881	FALSE	DOWN
LRRC27	358.2292	-1.81529	0.438213	-4.14249	3.44E-05	0.000313	FALSE	DOWN
SGTB	925.3198	-1.81378	0.37514	-4.83494	1.33E-06	1.92E-05	FALSE	DOWN
NUCKS1	16838.26	-1.81278	0.295154	-6.14182	8.16E-10	2.78E-08	FALSE	DOWN

PJA2	8710.279	-1.80967	0.390883	-4.6297	3.66E-06	4.60E-05	FALSE	DOWN
TMEM106C	3995.677	-1.80785	0.566854	-3.18926	0.001426	0.007104	FALSE	DOWN
CRYL1	1127.068	-1.80761	0.507503	-3.56176	0.000368	0.002294	FALSE	DOWN
KCTD7	1091.102	-1.8075	0.440574	-4.10259	4.09E-05	0.000362	FALSE	DOWN
TMEM44	826.0972	-1.80584	0.432229	-4.17798	2.94E-05	0.000275	FALSE	DOWN
TMEM69	975.254	-1.80255	0.252413	-7.14126	9.25E-13	5.52E-11	FALSE	DOWN
ADCK2	1754.399	-1.80031	0.343524	-5.24072	1.60E-07	2.98E-06	FALSE	DOWN
IDS	5852.881	-1.79498	0.419234	-4.28157	1.86E-05	0.000187	FALSE	DOWN
DDB2	1080.144	-1.79233	0.396854	-4.51635	6.29E-06	7.36E-05	FALSE	DOWN
PMVK	2307.55	-1.79212	0.360216	-4.97513	6.52E-07	1.01E-05	FALSE	DOWN
RDH11	3069.781	-1.79174	0.323198	-5.54378	2.96E-08	6.74E-07	FALSE	DOWN
PPIF	2118.848	-1.78962	0.478807	-3.73766	0.000186	0.001301	FALSE	DOWN
NRP1	10647.49	-1.78639	0.511031	-3.49567	0.000473	0.002841	FALSE	DOWN
TNFRSF14	2617.178	-1.78473	0.551677	-3.2351	0.001216	0.006245	FALSE	DOWN
SIVA1	3581.171	-1.78448	0.395101	-4.51651	6.29E-06	7.36E-05	FALSE	DOWN
GDE1	3527.425	-1.78279	0.269098	-6.62505	3.47E-11	1.53E-09	FALSE	DOWN
LAMB2	24314.1	-1.77989	0.433315	-4.10761	4.00E-05	0.000355	FALSE	DOWN
PLXND1	11636.51	-1.77639	0.493911	-3.59658	0.000322	0.002055	FALSE	DOWN
MAGEH1	2110.017	-1.77485	0.426457	-4.16185	3.16E-05	0.000293	FALSE	DOWN
CCDC28A	993.3663	-1.77419	0.543204	-3.26615	0.00109	0.005722	FALSE	DOWN
ANKRD42	432.1794	-1.77369	0.387101	-4.58199	4.61E-06	5.60E-05	FALSE	DOWN
LRPAP1	7467.418	-1.77001	0.318995	-5.54871	2.88E-08	6.56E-07	FALSE	DOWN
ECE1	9694.159	-1.76447	0.456893	-3.86189	0.000113	0.000853	FALSE	DOWN
VEGFB	5403.088	-1.76053	0.44422	-3.96319	7.40E-05	0.000598	FALSE	DOWN
APH1B	1008.988	-1.75919	0.392861	-4.47789	7.54E-06	8.54E-05	FALSE	DOWN
POLR2L	3516.99	-1.75775	0.526737	-3.33705	0.000847	0.004649	FALSE	DOWN
FAM160B2	2769.504	-1.75683	0.288338	-6.09295	1.11E-09	3.62E-08	FALSE	DOWN
ACTB	318126.5	-1.75554	0.379818	-4.62205	3.80E-06	4.76E-05	FALSE	DOWN
CD81	33597.75	-1.75385	0.402996	-4.35202	1.35E-05	0.000141	FALSE	DOWN
FAM50A	4836.427	-1.75329	0.488917	-3.58606	0.000336	0.002126	FALSE	DOWN
CLN6	2206.148	-1.75269	0.412303	-4.25098	2.13E-05	0.000211	FALSE	DOWN
CLIC4	27000.87	-1.75178	0.448229	-3.90823	9.30E-05	0.000725	FALSE	DOWN
TBC1D5	2967.142	-1.74531	0.30107	-5.79703	6.75E-09	1.83E-07	FALSE	DOWN
KLHDC8B	1756.535	-1.74462	0.484283	-3.60249	0.000315	0.002019	FALSE	DOWN
UBXN11	789.2364	-1.74383	0.550813	-3.16592	0.001546	0.00758	FALSE	DOWN
AHI1	1023.194	-1.74249	0.539866	-3.22764	0.001248	0.006391	FALSE	DOWN
CD99L2	4854.932	-1.73555	0.318916	-5.44203	5.27E-08	1.12E-06	FALSE	DOWN
PLOD1	18960.39	-1.73416	0.534676	-3.24338	0.001181	0.006103	FALSE	DOWN
LBR	4126.021	-1.73097	0.550549	-3.14408	0.001666	0.008081	FALSE	DOWN
PLD3	17903.45	-1.72743	0.501525	-3.44435	0.000572	0.003354	FALSE	DOWN
SNRNP200	10770.83	-1.72624	0.323803	-5.33113	9.76E-08	1.93E-06	FALSE	DOWN
EID1	11480.32	-1.72318	0.29941	-5.75526	8.65E-09	2.28E-07	FALSE	DOWN
PCM1	5544.617	-1.72238	0.355834	-4.84042	1.30E-06	1.87E-05	FALSE	DOWN
DTX3L	2917.446	-1.7203	0.377191	-4.56083	5.10E-06	6.12E-05	FALSE	DOWN
UBA52	24929.54	-1.71935	0.389673	-4.41228	1.02E-05	0.000112	FALSE	DOWN
HSDL2	1780.157	-1.71892	0.362397	-4.74321	2.10E-06	2.82E-05	FALSE	DOWN
ARL6IP4	6915.019	-1.71861	0.409344	-4.19846	2.69E-05	0.000257	FALSE	DOWN
DMXL2	1329.276	-1.71696	0.394689	-4.35016	1.36E-05	0.000142	FALSE	DOWN

ARRDC2	2169.599	-1.71234	0.496737	-3.44718	0.000566	0.003325	FALSE	DOWN
TAB3	1249.492	-1.71217	0.468694	-3.65307	0.000259	0.001726	FALSE	DOWN
ATP6V1C2	211.1552	-1.70992	0.53982	-3.16757	0.001537	0.007545	FALSE	DOWN
TCTN3	1972.735	-1.70977	0.45448	-3.76204	0.000169	0.0012	FALSE	DOWN
VPS13C	2655.103	-1.70971	0.37066	-4.6126	3.98E-06	4.95E-05	FALSE	DOWN
RNF135	1228.25	-1.70923	0.3367	-5.07642	3.85E-07	6.42E-06	FALSE	DOWN
KLHL8	695.3164	-1.70699	0.407002	-4.19405	2.74E-05	0.000261	FALSE	DOWN
EIF2AK1	5780.893	-1.7069	0.334339	-5.1053	3.30E-07	5.59E-06	FALSE	DOWN
BNC2	2317.531	-1.70585	0.443565	-3.84578	0.00012	0.000896	FALSE	DOWN
EHBP1	2777.791	-1.70453	0.380012	-4.48545	7.28E-06	8.29E-05	FALSE	DOWN
ATMIN	2346.905	-1.70412	0.300726	-5.66668	1.46E-08	3.64E-07	FALSE	DOWN
HDAC11	1166.387	-1.70313	0.522168	-3.26165	0.001108	0.005798	FALSE	DOWN
KDELR1	9094.896	-1.6986	0.353325	-4.80748	1.53E-06	2.14E-05	FALSE	DOWN
PGM1	4908.067	-1.69578	0.407466	-4.16177	3.16E-05	0.000293	FALSE	DOWN
MKRN1	4930.064	-1.69369	0.271023	-6.24927	4.12E-10	1.49E-08	FALSE	DOWN
DTWD1	1218.897	-1.69366	0.401885	-4.21428	2.51E-05	0.000243	FALSE	DOWN
NBAS	2286.287	-1.69335	0.277216	-6.10843	1.01E-09	3.35E-08	FALSE	DOWN
SNUPN	1319.908	-1.69311	0.288303	-5.87267	4.29E-09	1.22E-07	FALSE	DOWN
ERC1	3467.399	-1.69262	0.376651	-4.49388	6.99E-06	8.04E-05	FALSE	DOWN
ARV1	557.8502	-1.6915	0.426694	-3.96419	7.36E-05	0.000597	FALSE	DOWN
MFN2	5709.173	-1.68868	0.326462	-5.17268	2.31E-07	4.08E-06	FALSE	DOWN
CCDC25	1880.393	-1.68778	0.323542	-5.21658	1.82E-07	3.33E-06	FALSE	DOWN
MANBAL	3904.31	-1.68677	0.461329	-3.65633	0.000256	0.001708	FALSE	DOWN
DYSF	3282.789	-1.68662	0.523718	-3.22047	0.00128	0.006512	FALSE	DOWN
PIGT	9370.638	-1.68213	0.368205	-4.56847	4.91E-06	5.93E-05	FALSE	DOWN
ACADVL	8840.539	-1.68015	0.392383	-4.2819	1.85E-05	0.000187	FALSE	DOWN
TMEM205	2018.656	-1.67972	0.456244	-3.68163	0.000232	0.001571	FALSE	DOWN
PRMT2	4743.897	-1.67226	0.380799	-4.39145	1.13E-05	0.000122	FALSE	DOWN
NKIRAS2	3361.39	-1.67098	0.254925	-6.55479	5.57E-11	2.41E-09	FALSE	DOWN
KDM4D	101.6091	-1.66853	0.514898	-3.24051	0.001193	0.00616	FALSE	DOWN
PDXK	4550.499	-1.66431	0.400829	-4.15217	3.29E-05	0.000302	FALSE	DOWN
ANKRD13D	1508.067	-1.66068	0.36995	-4.48892	7.16E-06	8.17E-05	FALSE	DOWN
RABL3	927.2771	-1.66018	0.323941	-5.12494	2.98E-07	5.10E-06	FALSE	DOWN
OSBPL1A	2394.015	-1.65947	0.447537	-3.70801	0.000209	0.001436	FALSE	DOWN
KIF3B	2906.005	-1.65771	0.42947	-3.85989	0.000113	0.000858	FALSE	DOWN
KCTD21	791.9654	-1.65471	0.444459	-3.72298	0.000197	0.001365	FALSE	DOWN
SLC25A35	298.4577	-1.65443	0.501729	-3.29746	0.000976	0.005248	FALSE	DOWN
NAP1L1	18995.17	-1.65349	0.472435	-3.49994	0.000465	0.002802	FALSE	DOWN
GNE	1677.63	-1.65329	0.292636	-5.64965	1.61E-08	3.99E-07	FALSE	DOWN
CD151	12873.88	-1.65104	0.515198	-3.20466	0.001352	0.006813	FALSE	DOWN
TRIM13	978.9514	-1.65035	0.432166	-3.8188	0.000134	0.000984	FALSE	DOWN
CUTC	828.0773	-1.64915	0.476976	-3.4575	0.000545	0.00322	FALSE	DOWN
EEPD1	1039.921	-1.6491	0.531845	-3.10072	0.00193	0.009173	FALSE	DOWN
YEATS2	2448.556	-1.64672	0.322262	-5.10988	3.22E-07	5.47E-06	FALSE	DOWN
LRRC41	4770.472	-1.64516	0.228442	-7.20165	5.95E-13	3.63E-11	FALSE	DOWN
COMMD6	3503.727	-1.64194	0.441401	-3.71984	0.000199	0.001379	FALSE	DOWN
SLC25A25	2184.725	-1.64072	0.415699	-3.94689	7.92E-05	0.000634	FALSE	DOWN
STAT5A	2191.528	-1.63379	0.352047	-4.64084	3.47E-06	4.40E-05	FALSE	DOWN

THADA	1554.51	-1.63362	0.298601	-5.47089	4.48E-08	9.73E-07	FALSE	DOWN
VGLL4	3931.883	-1.63357	0.41427	-3.94325	8.04E-05	0.000641	FALSE	DOWN
ZHX3	3507.294	-1.63199	0.430985	-3.78664	0.000153	0.001104	FALSE	DOWN
ZSWIM7	780.6598	-1.63046	0.451943	-3.60767	0.000309	0.001988	FALSE	DOWN
H6PD	5903.259	-1.62757	0.449884	-3.61775	0.000297	0.001931	FALSE	DOWN
STARD7	5374.721	-1.62309	0.276214	-5.87622	4.20E-09	1.20E-07	FALSE	DOWN
COX11	1702.14	-1.62277	0.303842	-5.34083	9.25E-08	1.83E-06	FALSE	DOWN
RAB11FIP2	1204.496	-1.62031	0.403026	-4.02037	5.81E-05	0.00049	FALSE	DOWN
ORMDL1	1522.755	-1.61866	0.311309	-5.19953	2.00E-07	3.61E-06	FALSE	DOWN
NEIL2	833.8087	-1.61793	0.389129	-4.15782	3.21E-05	0.000297	FALSE	DOWN
ZXDC	2071.911	-1.61749	0.291386	-5.55103	2.84E-08	6.49E-07	FALSE	DOWN
KLF11	1762.901	-1.61649	0.325542	-4.96554	6.85E-07	1.06E-05	FALSE	DOWN
WASF1	1053.55	-1.61259	0.520546	-3.09789	0.001949	0.00924	FALSE	DOWN
MED16	3248.972	-1.61119	0.332225	-4.84969	1.24E-06	1.80E-05	FALSE	DOWN
TBC1D9B	8150.717	-1.60991	0.317356	-5.07287	3.92E-07	6.52E-06	FALSE	DOWN
RECQL	2549.752	-1.60741	0.388513	-4.13734	3.51E-05	0.000319	FALSE	DOWN
TRAM2	4572.113	-1.60644	0.48044	-3.34369	0.000827	0.004561	FALSE	DOWN
PMS1	703.8177	-1.60574	0.274311	-5.85373	4.81E-09	1.35E-07	FALSE	DOWN
TMEM62	576.3706	-1.60503	0.460522	-3.48524	0.000492	0.002932	FALSE	DOWN
ELK1	3115.932	-1.60111	0.513281	-3.11936	0.001812	0.008671	FALSE	DOWN
PPT1	7501.983	-1.60108	0.295209	-5.42353	5.84E-08	1.23E-06	FALSE	DOWN
RNF185	2509.153	-1.59908	0.344115	-4.64693	3.37E-06	4.30E-05	FALSE	DOWN
CRTAP	17812.57	-1.59832	0.32868	-4.86286	1.16E-06	1.70E-05	FALSE	DOWN
NADSYN1	1571.335	-1.59636	0.322612	-4.94825	7.49E-07	1.14E-05	FALSE	DOWN
PARP4	2345.562	-1.59568	0.416995	-3.82662	0.00013	0.000959	FALSE	DOWN
RARS2	1575.32	-1.59339	0.319733	-4.98351	6.24E-07	9.80E-06	FALSE	DOWN
ARMC6	1509.695	-1.58985	0.355302	-4.47465	7.65E-06	8.64E-05	FALSE	DOWN
ST6GALNA(	5009.447	-1.58983	0.415248	-3.82862	0.000129	0.000952	FALSE	DOWN
MCCC2	1594.044	-1.58866	0.342164	-4.64298	3.43E-06	4.36E-05	FALSE	DOWN
SLC35D2	1004.363	-1.58154	0.317653	-4.97882	6.40E-07	1.00E-05	FALSE	DOWN
SP100	3348.72	-1.58007	0.373798	-4.22705	2.37E-05	0.000231	FALSE	DOWN
SMPD1	3136.908	-1.58004	0.459495	-3.43866	0.000585	0.003407	FALSE	DOWN
SLC25A13	916.1466	-1.57307	0.397382	-3.9586	7.54E-05	0.000608	FALSE	DOWN
DDT	1454.919	-1.5701	0.456635	-3.43841	0.000585	0.003409	FALSE	DOWN
TADA2B	1591.896	-1.56869	0.280744	-5.5876	2.30E-08	5.40E-07	FALSE	DOWN
ABHD10	1255.295	-1.56258	0.301557	-5.1817	2.20E-07	3.92E-06	FALSE	DOWN
CTDSPL	2548.029	-1.56128	0.397684	-3.92594	8.64E-05	0.000682	FALSE	DOWN
RRM2B	1797.103	-1.55993	0.39112	-3.98835	6.65E-05	0.00055	FALSE	DOWN
POLDIP2	4800.136	-1.55823	0.279927	-5.56656	2.60E-08	6.03E-07	FALSE	DOWN
SMAD2	3412.58	-1.55543	0.258396	-6.01958	1.75E-09	5.49E-08	FALSE	DOWN
GTF3C5	2557.28	-1.5545	0.305002	-5.09668	3.46E-07	5.83E-06	FALSE	DOWN
SH3YL1	648.5464	-1.55346	0.390721	-3.97586	7.01E-05	0.000574	FALSE	DOWN
MGAT1	14764.21	-1.55131	0.422289	-3.67358	0.000239	0.001615	FALSE	DOWN
ECH1	7195.926	-1.5505	0.356127	-4.35379	1.34E-05	0.000141	FALSE	DOWN
COASY	2427.783	-1.54791	0.266386	-5.81077	6.22E-09	1.70E-07	FALSE	DOWN
ITPKC	1616.653	-1.54765	0.400152	-3.86765	0.00011	0.000837	FALSE	DOWN
ZER1	4291.167	-1.54505	0.3293	-4.69193	2.71E-06	3.53E-05	FALSE	DOWN
GLOD4	2269.976	-1.54431	0.27859	-5.5433	2.97E-08	6.74E-07	FALSE	DOWN

SSU72	5068.284	-1.54215	0.467599	-3.29802	0.000974	0.00524	FALSE	DOWN
BAG5	2588.55	-1.54142	0.373263	-4.12958	3.63E-05	0.000327	FALSE	DOWN
ACSL3	3517.22	-1.53959	0.394368	-3.90394	9.46E-05	0.000735	FALSE	DOWN
PPP1CB	15065.8	-1.53762	0.391807	-3.92442	8.69E-05	0.000684	FALSE	DOWN
NUP43	1595.471	-1.5329	0.38531	-3.97835	6.94E-05	0.000569	FALSE	DOWN
RPA2	2707.214	-1.53286	0.285025	-5.37798	7.53E-08	1.52E-06	FALSE	DOWN
KIF22	2623.52	-1.53178	0.420307	-3.64444	0.000268	0.001773	FALSE	DOWN
ALDH9A1	4265.319	-1.52805	0.365701	-4.17842	2.94E-05	0.000274	FALSE	DOWN
TBC1D25	1351.067	-1.52307	0.39496	-3.85625	0.000115	0.000867	FALSE	DOWN
VAMP2	4205.766	-1.52192	0.394447	-3.85837	0.000114	0.000861	FALSE	DOWN
ADCY3	3239.962	-1.52164	0.472664	-3.21928	0.001285	0.006532	FALSE	DOWN
TADA3	4764.364	-1.5211	0.367331	-4.14094	3.46E-05	0.000315	FALSE	DOWN
STK40	3184.782	-1.51878	0.337526	-4.49976	6.80E-06	7.86E-05	FALSE	DOWN
YTHDC2	1142.006	-1.5127	0.282261	-5.35921	8.36E-08	1.67E-06	FALSE	DOWN
HMGXB3	2550.121	-1.51214	0.251094	-6.02222	1.72E-09	5.41E-08	FALSE	DOWN
MRPL16	1481.2	-1.50771	0.300526	-5.01691	5.25E-07	8.44E-06	FALSE	DOWN
TMEM109	7498.523	-1.50639	0.248693	-6.05721	1.39E-09	4.43E-08	FALSE	DOWN
MRPS27	2999.049	-1.50466	0.279088	-5.39133	6.99E-08	1.43E-06	FALSE	DOWN
DPYSL2	9074.853	-1.50182	0.358349	-4.19095	2.78E-05	0.000263	FALSE	DOWN
NUCB1	18622.54	-1.50179	0.333138	-4.50801	6.54E-06	7.60E-05	FALSE	DOWN
RWDD2A	230.9105	-1.50126	0.339846	-4.41747	9.99E-06	0.00011	FALSE	DOWN
RBL2	2826.311	-1.50106	0.331266	-4.5313	5.86E-06	6.92E-05	FALSE	DOWN
NPHP3	1000.002	-1.50098	0.379244	-3.95783	7.56E-05	0.000609	FALSE	DOWN
MKS1	535.72	-1.50061	0.331963	-4.52042	6.17E-06	7.24E-05	FALSE	DOWN
TCEA2	1691.054	-1.50014	0.449409	-3.33804	0.000844	0.004636	FALSE	DOWN
PARP1	5790.836	-1.49508	0.329765	-4.53379	5.79E-06	6.87E-05	FALSE	DOWN
QRSL1	757.1321	-1.49398	0.282153	-5.29493	1.19E-07	2.29E-06	FALSE	DOWN
HAUS4	1745.96	-1.49225	0.335918	-4.44231	8.90E-06	9.85E-05	FALSE	DOWN
WDR25	545.3305	-1.48996	0.405744	-3.67217	0.00024	0.001622	FALSE	DOWN
DHRS7	2217.764	-1.48953	0.396314	-3.75847	0.000171	0.001211	FALSE	DOWN
AKAP13	8254.332	-1.4891	0.366548	-4.06251	4.85E-05	0.000421	FALSE	DOWN
ABI2	3019.572	-1.48787	0.357699	-4.15957	3.19E-05	0.000295	FALSE	DOWN
YIF1A	2933.893	-1.48729	0.453323	-3.28086	0.001035	0.005483	FALSE	DOWN
PTTG1IP	14221.57	-1.48529	0.373006	-3.98194	6.84E-05	0.000562	FALSE	DOWN
BSDC1	4076.206	-1.4847	0.245775	-6.04092	1.53E-09	4.84E-08	FALSE	DOWN
DOCK1	3817.109	-1.48313	0.407387	-3.64058	0.000272	0.001795	FALSE	DOWN
L3MBTL2	2019.972	-1.47781	0.269614	-5.48122	4.22E-08	9.20E-07	FALSE	DOWN
RAB5B	8784.618	-1.47463	0.337285	-4.37206	1.23E-05	0.000132	FALSE	DOWN
KCTD3	2655.246	-1.47436	0.310475	-4.74872	2.05E-06	2.76E-05	FALSE	DOWN
PARN	2446.907	-1.47137	0.2716	-5.4174	6.05E-08	1.26E-06	FALSE	DOWN
RUSC1	3133.17	-1.4711	0.391478	-3.75781	0.000171	0.001213	FALSE	DOWN
CRY1	1218.694	-1.46996	0.362525	-4.05479	5.02E-05	0.000433	FALSE	DOWN
CPNE1	5684.584	-1.46983	0.441243	-3.33111	0.000865	0.004738	FALSE	DOWN
MRPL40	1379.649	-1.46915	0.373194	-3.93671	8.26E-05	0.000655	FALSE	DOWN
OSTM1	2593.595	-1.46575	0.413979	-3.54063	0.000399	0.002457	FALSE	DOWN
MRPL37	3453.897	-1.46441	0.307279	-4.76573	1.88E-06	2.58E-05	FALSE	DOWN
ASAP2	2179.681	-1.46428	0.427171	-3.42785	0.000608	0.003522	FALSE	DOWN
TTLL3	1764.745	-1.46349	0.452719	-3.23266	0.001226	0.006292	FALSE	DOWN

NDUFS5	5321.797	-1.46337	0.369042	-3.96531	7.33E-05	0.000595	FALSE	DOWN
GOPC	2644.816	-1.46335	0.447305	-3.27148	0.00107	0.005643	FALSE	DOWN
ECHS1	3871.308	-1.46318	0.452617	-3.2327	0.001226	0.006292	FALSE	DOWN
PLCG1	5879.358	-1.46296	0.389199	-3.7589	0.000171	0.00121	FALSE	DOWN
PLSCR1	2416.561	-1.46259	0.408696	-3.57867	0.000345	0.002175	FALSE	DOWN
CTNNAL1	2115.828	-1.45857	0.447305	-3.26079	0.001111	0.005812	FALSE	DOWN
RIN3	2709.605	-1.45273	0.409468	-3.54785	0.000388	0.0024	FALSE	DOWN
NUPL2	878.9059	-1.45082	0.265946	-5.45532	4.89E-08	1.06E-06	FALSE	DOWN
ATN1	11475.79	-1.45073	0.356386	-4.07068	4.69E-05	0.000407	FALSE	DOWN
CMIP	3045.784	-1.45012	0.454555	-3.19021	0.001422	0.007091	FALSE	DOWN
PNPO	1240.653	-1.44961	0.435107	-3.33161	0.000863	0.004731	FALSE	DOWN
TBCD	4059.076	-1.44644	0.354357	-4.08187	4.47E-05	0.000391	FALSE	DOWN
SF3A1	5735.897	-1.44642	0.234918	-6.15715	7.41E-10	2.56E-08	FALSE	DOWN
NUMB	2272.396	-1.44577	0.290632	-4.97457	6.54E-07	1.02E-05	FALSE	DOWN
GAB2	2442.508	-1.44418	0.460239	-3.13789	0.001702	0.008215	FALSE	DOWN
CNTROB	2082.748	-1.4429	0.373672	-3.8614	0.000113	0.000854	FALSE	DOWN
PRKCSH	15856.77	-1.4427	0.320071	-4.50745	6.56E-06	7.62E-05	FALSE	DOWN
RAB2B	1194.669	-1.4399	0.32068	-4.49014	7.12E-06	8.14E-05	FALSE	DOWN
DUSP22	2025.701	-1.43753	0.352995	-4.07238	4.65E-05	0.000405	FALSE	DOWN
DHTKD1	892.4571	-1.43709	0.461757	-3.11223	0.001857	0.008859	FALSE	DOWN
GPAA1	5431.238	-1.43536	0.341009	-4.20914	2.56E-05	0.000247	FALSE	DOWN
FAHD1	1158.554	-1.43424	0.354238	-4.04881	5.15E-05	0.000442	FALSE	DOWN
CRAT	4073.659	-1.43167	0.376841	-3.79914	0.000145	0.001054	FALSE	DOWN
WDTC1	3648.025	-1.4296	0.268799	-5.31848	1.05E-07	2.06E-06	FALSE	DOWN
TPP1	11423.88	-1.42786	0.405224	-3.52363	0.000426	0.002591	FALSE	DOWN
STX17	1563.825	-1.42781	0.34539	-4.1339	3.57E-05	0.000323	FALSE	DOWN
PEF1	4024.971	-1.42417	0.250421	-5.68712	1.29E-08	3.27E-07	FALSE	DOWN
IPP	374.0804	-1.42274	0.455868	-3.12096	0.001803	0.008639	FALSE	DOWN
SLC25A38	1761.803	-1.42169	0.274213	-5.18462	2.16E-07	3.86E-06	FALSE	DOWN
CD320	2339.215	-1.42146	0.41937	-3.38952	0.0007	0.003957	FALSE	DOWN
TSR2	2449.059	-1.4208	0.346998	-4.09453	4.23E-05	0.000373	FALSE	DOWN
FAM122A	1177.49	-1.41992	0.338021	-4.20067	2.66E-05	0.000255	FALSE	DOWN
FAM3A	2348.423	-1.41677	0.452485	-3.1311	0.001742	0.008378	FALSE	DOWN
ZC3H6	623.249	-1.41496	0.389931	-3.62875	0.000285	0.001862	FALSE	DOWN
EEFSEC	1228.624	-1.41372	0.361862	-3.90679	9.35E-05	0.000728	FALSE	DOWN
PHTF2	1821.199	-1.41019	0.387397	-3.64017	0.000272	0.001797	FALSE	DOWN
KDSR	3837.978	-1.40881	0.364703	-3.86289	0.000112	0.00085	FALSE	DOWN
PTGES2	1712.4	-1.40703	0.40466	-3.47708	0.000507	0.003014	FALSE	DOWN
UBE2A	3243.076	-1.40565	0.23681	-5.93575	2.93E-09	8.74E-08	FALSE	DOWN
CREBBP	5462.103	-1.40165	0.334364	-4.19197	2.77E-05	0.000262	FALSE	DOWN
ANXA11	8166.493	-1.40119	0.454992	-3.0796	0.002073	0.009725	FALSE	DOWN
MPST	2147.587	-1.40095	0.431826	-3.24424	0.001178	0.006089	FALSE	DOWN
SNX19	3602.373	-1.39829	0.336274	-4.15817	3.21E-05	0.000297	FALSE	DOWN
PEPD	3535.108	-1.39546	0.453898	-3.0744	0.002109	0.009866	FALSE	DOWN
TESK1	3069.897	-1.39523	0.327865	-4.25551	2.09E-05	0.000207	FALSE	DOWN
MADD	2402.45	-1.39318	0.213505	-6.52529	6.79E-11	2.89E-09	FALSE	DOWN
PCCB	2450.058	-1.39286	0.293467	-4.74623	2.07E-06	2.78E-05	FALSE	DOWN
RNF216	4364.632	-1.38976	0.386767	-3.59326	0.000327	0.002077	FALSE	DOWN

TMEM115	3449.831	-1.38836	0.302404	-4.59107	4.41E-06	5.42E-05	FALSE	DOWN
MLH1	1715.211	-1.38809	0.214333	-6.47629	9.40E-11	3.91E-09	FALSE	DOWN
DAXX	3509.894	-1.38734	0.295662	-4.69231	2.70E-06	3.53E-05	FALSE	DOWN
PRKACA	8144.197	-1.38145	0.338601	-4.07987	4.51E-05	0.000394	FALSE	DOWN
TMEM42	579.2559	-1.37825	0.377461	-3.65137	0.000261	0.001734	FALSE	DOWN
NCOA4	7181.142	-1.37746	0.348468	-3.95289	7.72E-05	0.00062	FALSE	DOWN
HADHA	9056.662	-1.37595	0.304139	-4.52408	6.07E-06	7.12E-05	FALSE	DOWN
MEAF6	2801.293	-1.37361	0.257235	-5.33991	9.30E-08	1.84E-06	FALSE	DOWN
ADD1	15896.39	-1.37274	0.271903	-5.04866	4.45E-07	7.29E-06	FALSE	DOWN
SPEN	3705.128	-1.37203	0.329304	-4.16645	3.09E-05	0.000287	FALSE	DOWN
WDR13	4293.372	-1.36988	0.356038	-3.84758	0.000119	0.000892	FALSE	DOWN
UBIAD1	962.4914	-1.36904	0.285092	-4.80212	1.57E-06	2.19E-05	FALSE	DOWN
PRKD2	2123.861	-1.36415	0.373011	-3.65714	0.000255	0.001704	FALSE	DOWN
LRP10	9815.347	-1.36375	0.373864	-3.64772	0.000265	0.001754	FALSE	DOWN
HSPB11	993.22	-1.36256	0.426855	-3.1921	0.001412	0.007063	FALSE	DOWN
UBR7	2796.396	-1.36226	0.380655	-3.57873	0.000345	0.002175	FALSE	DOWN
HTATSF1	4838.776	-1.36152	0.273121	-4.98505	6.19E-07	9.76E-06	FALSE	DOWN
MORC4	1639.937	-1.36034	0.431887	-3.14976	0.001634	0.00795	FALSE	DOWN
SETDB2	557.0794	-1.3582	0.412805	-3.29016	0.001001	0.005341	FALSE	DOWN
MRPS2	1880.243	-1.35346	0.340104	-3.97953	6.91E-05	0.000567	FALSE	DOWN
ZYG11B	2539.213	-1.35228	0.296203	-4.56537	4.99E-06	6.01E-05	FALSE	DOWN
ZFYVE21	2921.081	-1.35136	0.403202	-3.35158	0.000804	0.004445	FALSE	DOWN
SGSM3	2791.499	-1.34878	0.314742	-4.28534	1.82E-05	0.000184	FALSE	DOWN
AP3S2	3208.568	-1.34749	0.301665	-4.46686	7.94E-06	8.90E-05	FALSE	DOWN
RIC8A	4488.658	-1.34701	0.252057	-5.34408	9.09E-08	1.81E-06	FALSE	DOWN
CPNE3	4127.22	-1.34685	0.403553	-3.33749	0.000845	0.004643	FALSE	DOWN
MUL1	2159.565	-1.34537	0.245588	-5.47815	4.30E-08	9.35E-07	FALSE	DOWN
EHD2	14670.69	-1.34331	0.42591	-3.15399	0.001611	0.007857	FALSE	DOWN
GTF3C1	6016.331	-1.34281	0.286598	-4.68533	2.80E-06	3.63E-05	FALSE	DOWN
TRAF3IP2	1256.308	-1.34236	0.418518	-3.2074	0.001339	0.006757	FALSE	DOWN
PCTP	793.5438	-1.34139	0.408895	-3.28053	0.001036	0.005484	FALSE	DOWN
UBAP1	3180.856	-1.33851	0.278388	-4.80807	1.52E-06	2.14E-05	FALSE	DOWN
UROS	1199.394	-1.33722	0.41546	-3.21865	0.001288	0.006544	FALSE	DOWN
POP7	1073.709	-1.33709	0.289916	-4.61201	3.99E-06	4.96E-05	FALSE	DOWN
ST3GAL3	1260.888	-1.33686	0.37283	-3.58572	0.000336	0.002128	FALSE	DOWN
HDDC3	567.5788	-1.33534	0.364558	-3.6629	0.000249	0.001671	FALSE	DOWN
ERCC1	2496.371	-1.33446	0.357684	-3.73083	0.000191	0.001331	FALSE	DOWN
PI4KA	4864.802	-1.33171	0.396547	-3.35826	0.000784	0.004353	FALSE	DOWN
EIF2AK4	2547.328	-1.33011	0.317372	-4.19102	2.78E-05	0.000263	FALSE	DOWN
TMEM43	9281.81	-1.32952	0.373401	-3.56056	0.00037	0.002302	FALSE	DOWN
WDR89	499.5264	-1.32917	0.335172	-3.96564	7.32E-05	0.000595	FALSE	DOWN
DHPS	2350.074	-1.32635	0.334021	-3.97087	7.16E-05	0.000585	FALSE	DOWN
MAML1	3006.804	-1.32032	0.318312	-4.14789	3.36E-05	0.000307	FALSE	DOWN
DGCR2	6047.326	-1.3196	0.280515	-4.70421	2.55E-06	3.36E-05	FALSE	DOWN
IGHMBP2	913.6618	-1.31726	0.366163	-3.59746	0.000321	0.00205	FALSE	DOWN
PRDX3	4218.435	-1.31389	0.291212	-4.51181	6.43E-06	7.50E-05	FALSE	DOWN
MAPKAP1	4070.376	-1.31381	0.301489	-4.35774	1.31E-05	0.000139	FALSE	DOWN
RFXANK	2049.296	-1.31221	0.325566	-4.03054	5.56E-05	0.000471	FALSE	DOWN

RAF1	5614.766	-1.30933	0.264406	-4.95195	7.35E-07	1.13E-05	FALSE	DOWN
ACSF3	916.7295	-1.30804	0.326529	-4.0059	6.18E-05	0.000516	FALSE	DOWN
PATZ1	1846.214	-1.30339	0.320912	-4.06151	4.88E-05	0.000422	FALSE	DOWN
PHF8	1498.003	-1.30318	0.333974	-3.90205	9.54E-05	0.000739	FALSE	DOWN
PLEKHM2	4954.364	-1.30246	0.328413	-3.96592	7.31E-05	0.000594	FALSE	DOWN
METTL4	680.875	-1.30202	0.384803	-3.3836	0.000715	0.00403	FALSE	DOWN
MPG	2449.973	-1.30098	0.340621	-3.81944	0.000134	0.000982	FALSE	DOWN
UBE2Q2	2463.507	-1.30045	0.321873	-4.04027	5.34E-05	0.000455	FALSE	DOWN
SLC39A9	3316.417	-1.29632	0.286338	-4.52724	5.98E-06	7.03E-05	FALSE	DOWN
CBLL1	1108.746	-1.29612	0.294882	-4.39538	1.11E-05	0.00012	FALSE	DOWN
BBS2	1299.776	-1.29482	0.324729	-3.98737	6.68E-05	0.000552	FALSE	DOWN
DDX19B	1477.063	-1.29477	0.344026	-3.76357	0.000168	0.001194	FALSE	DOWN
SERTAD3	1388.926	-1.29258	0.357957	-3.61099	0.000305	0.00197	FALSE	DOWN
ACTR6	974.6744	-1.28897	0.402675	-3.20103	0.001369	0.006882	FALSE	DOWN
AP1M1	4313.465	-1.28563	0.265906	-4.83491	1.33E-06	1.92E-05	FALSE	DOWN
APLP2	27606.35	-1.28519	0.338524	-3.79644	0.000147	0.001064	FALSE	DOWN
ALAD	2418.38	-1.28479	0.323233	-3.97481	7.04E-05	0.000576	FALSE	DOWN
TTC23	1278.558	-1.28426	0.320977	-4.00109	6.31E-05	0.000526	FALSE	DOWN
YPEL2	1433.219	-1.2825	0.404695	-3.16905	0.001529	0.007512	FALSE	DOWN
PKN1	11471.1	-1.28142	0.359039	-3.56902	0.000358	0.002242	FALSE	DOWN
PREPL	3064.314	-1.28062	0.408004	-3.13874	0.001697	0.008197	FALSE	DOWN
VPS45	2372.166	-1.28049	0.312872	-4.09271	4.26E-05	0.000376	FALSE	DOWN
MTMR12	1677.859	-1.28047	0.380892	-3.36177	0.000774	0.004305	FALSE	DOWN
LRPPRC	4315.963	-1.27891	0.333697	-3.83253	0.000127	0.000938	FALSE	DOWN
CBR1	2911.718	-1.2786	0.406801	-3.14305	0.001672	0.0081	FALSE	DOWN
AK3	3558.465	-1.27736	0.365415	-3.49563	0.000473	0.002841	FALSE	DOWN
BBS5	494.7322	-1.27427	0.330839	-3.85164	0.000117	0.00088	FALSE	DOWN
GANAB	21312.5	-1.27087	0.223756	-5.67972	1.35E-08	3.40E-07	FALSE	DOWN
MGRN1	4384.069	-1.27026	0.257049	-4.9417	7.74E-07	1.18E-05	FALSE	DOWN
RNF40	5402.545	-1.26893	0.217685	-5.82921	5.57E-09	1.54E-07	FALSE	DOWN
PPP1R3D	478.0208	-1.2679	0.38988	-3.25203	0.001146	0.005958	FALSE	DOWN
CHID1	3723.617	-1.26788	0.400566	-3.16522	0.00155	0.007592	FALSE	DOWN
PRPS1	2113.677	-1.26286	0.337262	-3.74444	0.000181	0.00127	FALSE	DOWN
INTS10	2436.872	-1.26227	0.31835	-3.96504	7.34E-05	0.000596	FALSE	DOWN
VPS4A	4251.643	-1.26179	0.2537	-4.97352	6.57E-07	1.02E-05	FALSE	DOWN
GLG1	13733.84	-1.26174	0.351504	-3.58954	0.000331	0.002102	FALSE	DOWN
ULK3	1557.034	-1.25827	0.372808	-3.37512	0.000738	0.004135	FALSE	DOWN
DDX56	2645.456	-1.25811	0.279504	-4.50122	6.76E-06	7.82E-05	FALSE	DOWN
CHMP1B	3141.305	-1.25668	0.325191	-3.86444	0.000111	0.000845	FALSE	DOWN
FANCG	853.8773	-1.25513	0.386744	-3.24539	0.001173	0.006073	FALSE	DOWN
SYDE1	3330.063	-1.2513	0.342526	-3.65315	0.000259	0.001726	FALSE	DOWN
CUL4B	3833.418	-1.25127	0.325567	-3.84336	0.000121	0.000904	FALSE	DOWN
ARHGAP17	2824.274	-1.24855	0.26311	-4.74534	2.08E-06	2.79E-05	FALSE	DOWN
RFX5	2379.8	-1.24677	0.361046	-3.45321	0.000554	0.003263	FALSE	DOWN
CIAO1	3462.078	-1.24524	0.184289	-6.75701	1.41E-11	6.74E-10	FALSE	DOWN
CHURC1	2885.766	-1.24435	0.362463	-3.43306	0.000597	0.00347	FALSE	DOWN
MED8	1847.772	-1.24109	0.260703	-4.76053	1.93E-06	2.63E-05	FALSE	DOWN
AP2B1	8758.621	-1.23907	0.2734	-4.53209	5.84E-06	6.91E-05	FALSE	DOWN

PRKRA	1799.4	-1.23767	0.29623	-4.17807	2.94E-05	0.000275	FALSE	DOWN
CLEC16A	1446.752	-1.23515	0.311226	-3.96866	7.23E-05	0.000589	FALSE	DOWN
WBP2	6471.711	-1.23477	0.338231	-3.65066	0.000262	0.001738	FALSE	DOWN
HMGCL	1937.6	-1.23458	0.370121	-3.33561	0.000851	0.004671	FALSE	DOWN
WIZ	4375.741	-1.2338	0.322131	-3.83012	0.000128	0.000947	FALSE	DOWN
DCTN1	8602.713	-1.23338	0.266151	-4.63413	3.58E-06	4.52E-05	FALSE	DOWN
SLC35E1	3337.137	-1.23297	0.311074	-3.96361	7.38E-05	0.000598	FALSE	DOWN
CD164	15413.11	-1.23158	0.374582	-3.28787	0.001009	0.005376	FALSE	DOWN
SIN3A	3944.088	-1.22982	0.371427	-3.31106	0.000929	0.005033	FALSE	DOWN
NDUFB4	3331.838	-1.22928	0.345468	-3.55831	0.000373	0.002319	FALSE	DOWN
HMGN2	14507.06	-1.22665	0.386223	-3.17603	0.001493	0.007359	FALSE	DOWN
TIGD5	422.2367	-1.22387	0.356219	-3.43573	0.000591	0.00344	FALSE	DOWN
ZFP36L1	18084.76	-1.22234	0.378882	-3.22619	0.001255	0.006419	FALSE	DOWN
RBMX2	837.9955	-1.21707	0.287173	-4.23812	2.25E-05	0.000221	FALSE	DOWN
SCAMP4	3311.463	-1.21525	0.314756	-3.86092	0.000113	0.000855	FALSE	DOWN
TXLNA	5692.609	-1.21503	0.232176	-5.23322	1.67E-07	3.07E-06	FALSE	DOWN
AP2A1	6245.344	-1.21285	0.295099	-4.10997	3.96E-05	0.000352	FALSE	DOWN
ERCC5	2075.382	-1.21159	0.318096	-3.8089	0.00014	0.001018	FALSE	DOWN
SORBS3	7783.458	-1.21114	0.339347	-3.56903	0.000358	0.002242	FALSE	DOWN
ZNFX1	4218.537	-1.21027	0.35743	-3.38603	0.000709	0.003998	FALSE	DOWN
JMJD8	3532.547	-1.20646	0.345958	-3.48731	0.000488	0.002917	FALSE	DOWN
KDELC2	5318.392	-1.20608	0.37685	-3.20042	0.001372	0.006894	FALSE	DOWN
TMEM59	11492.73	-1.20532	0.28825	-4.1815	2.90E-05	0.000272	FALSE	DOWN
MLST8	1944.742	-1.2037	0.280567	-4.29022	1.78E-05	0.000181	FALSE	DOWN
TMEM101	1649.74	-1.1954	0.330563	-3.61626	0.000299	0.00194	FALSE	DOWN
TMEM138	1002.131	-1.19455	0.319196	-3.74237	0.000182	0.001279	FALSE	DOWN
RRAGA	4372.202	-1.19307	0.319688	-3.73198	0.00019	0.001327	FALSE	DOWN
BAP1	4557.761	-1.19213	0.224765	-5.3039	1.13E-07	2.19E-06	FALSE	DOWN
RAD1	1711.763	-1.18878	0.35946	-3.30713	0.000943	0.005091	FALSE	DOWN
IARS2	3951.07	-1.18651	0.272948	-4.34703	1.38E-05	0.000144	FALSE	DOWN
RSL24D1	3899.431	-1.18494	0.285454	-4.15109	3.31E-05	0.000304	FALSE	DOWN
COIL	825.1865	-1.18438	0.267841	-4.42194	9.78E-06	0.000108	FALSE	DOWN
KLHL12	1475.927	-1.18351	0.264182	-4.47989	7.47E-06	8.46E-05	FALSE	DOWN
SUPT7L	1998.452	-1.1825	0.219564	-5.38564	7.22E-08	1.48E-06	FALSE	DOWN
ZBTB4	5364.916	-1.18	0.307226	-3.8408	0.000123	0.000912	FALSE	DOWN
WASL	3688.412	-1.17848	0.357698	-3.29463	0.000986	0.005293	FALSE	DOWN
HADHB	4561.72	-1.17794	0.301698	-3.90437	9.45E-05	0.000734	FALSE	DOWN
MTR	2443.061	-1.17756	0.347269	-3.39092	0.000697	0.003939	FALSE	DOWN
ANAPC16	4154.175	-1.17737	0.325475	-3.61737	0.000298	0.001933	FALSE	DOWN
EXOC4	2945.505	-1.17559	0.255286	-4.60499	4.12E-06	5.11E-05	FALSE	DOWN
USP20	2136.897	-1.17327	0.374026	-3.13687	0.001708	0.008241	FALSE	DOWN
STK24	4814.043	-1.17315	0.334994	-3.502	0.000462	0.002784	FALSE	DOWN
NBN	2061.261	-1.17216	0.324758	-3.60932	0.000307	0.00198	FALSE	DOWN
STAT3	10042.8	-1.17141	0.290537	-4.03187	5.53E-05	0.00047	FALSE	DOWN
TAF1C	1632.859	-1.17015	0.35908	-3.25874	0.001119	0.005845	FALSE	DOWN
TRIM68	543.984	-1.16951	0.348903	-3.35198	0.000802	0.004441	FALSE	DOWN
SCAMP1	2615.272	-1.16936	0.284543	-4.10961	3.96E-05	0.000353	FALSE	DOWN
POMT1	1686.865	-1.16705	0.296767	-3.93256	8.40E-05	0.000666	FALSE	DOWN

HNRNPUL1	12189.86	-1.16521	0.296798	-3.92593	8.64E-05	0.000682	FALSE	DOWN
SEC16A	5247.172	-1.16387	0.283028	-4.11222	3.92E-05	0.000349	FALSE	DOWN
OXA1L	3962.757	-1.16278	0.268888	-4.3244	1.53E-05	0.000158	FALSE	DOWN
CCDC130	1502.882	-1.16273	0.329373	-3.53012	0.000415	0.002537	FALSE	DOWN
TUBGCP2	2889.174	-1.16209	0.257319	-4.51614	6.30E-06	7.36E-05	FALSE	DOWN
OGDH	7781.741	-1.16174	0.331553	-3.50394	0.000458	0.002769	FALSE	DOWN
PDE8A	1559.869	-1.16146	0.365082	-3.18135	0.001466	0.007247	FALSE	DOWN
UBAC2	3122.545	-1.16032	0.37125	-3.12544	0.001775	0.008521	FALSE	DOWN
SEC22C	1381.6	-1.15752	0.325224	-3.55915	0.000372	0.002314	FALSE	DOWN
ERAL1	2117.478	-1.15573	0.212703	-5.43352	5.53E-08	1.17E-06	FALSE	DOWN
SYNRG	1788.592	-1.15562	0.289418	-3.99292	6.53E-05	0.000541	FALSE	DOWN
OAZ1	26361.97	-1.15519	0.372832	-3.09842	0.001946	0.009228	FALSE	DOWN
NOMO1	9493.108	-1.15334	0.350518	-3.2904	0.001	0.005338	FALSE	DOWN
MAPK1	6901.815	-1.15263	0.297264	-3.87745	0.000106	0.000809	FALSE	DOWN
NPLOC4	5041.198	-1.14951	0.219281	-5.24218	1.59E-07	2.97E-06	FALSE	DOWN
PSMD10	1970.619	-1.14831	0.246561	-4.65729	3.20E-06	4.11E-05	FALSE	DOWN
VAMP3	5344.188	-1.14652	0.35364	-3.24205	0.001187	0.006129	FALSE	DOWN
MLX	2194.096	-1.14513	0.214	-5.35109	8.74E-08	1.75E-06	FALSE	DOWN
HGSNAT	4868.88	-1.14513	0.310013	-3.69381	0.000221	0.001508	FALSE	DOWN
SRP14	9185.431	-1.14352	0.203303	-5.62469	1.86E-08	4.50E-07	FALSE	DOWN
UBOX5	797.5254	-1.1435	0.351848	-3.24997	0.001154	0.005985	FALSE	DOWN
ASCC1	1103.739	-1.1418	0.358304	-3.18669	0.001439	0.007139	FALSE	DOWN
GPBP1L1	3552.797	-1.14125	0.207599	-5.49737	3.86E-08	8.49E-07	FALSE	DOWN
EIF3L	14113.4	-1.13741	0.325266	-3.49687	0.000471	0.002831	FALSE	DOWN
SCAP	4287.382	-1.1366	0.236605	-4.80379	1.56E-06	2.18E-05	FALSE	DOWN
SCCPDH	2143.201	-1.13604	0.361833	-3.13967	0.001691	0.008183	FALSE	DOWN
IMPAD1	6127.093	-1.13343	0.33111	-3.42312	0.000619	0.003573	FALSE	DOWN
WASF2	9871.126	-1.13293	0.316187	-3.58309	0.00034	0.002145	FALSE	DOWN
BRWD1	2001.452	-1.1329	0.348809	-3.24792	0.001163	0.006024	FALSE	DOWN
CASC3	5046.461	-1.1328	0.329211	-3.44096	0.00058	0.00339	FALSE	DOWN
METTL5	983.3447	-1.13191	0.320606	-3.53055	0.000415	0.002537	FALSE	DOWN
TINF2	2757.319	-1.13106	0.251719	-4.49337	7.01E-06	8.05E-05	FALSE	DOWN
SF3A2	3940.818	-1.12905	0.346955	-3.25416	0.001137	0.005922	FALSE	DOWN
WDR82	7320.929	-1.12883	0.270601	-4.17157	3.03E-05	0.000281	FALSE	DOWN
POLR1E	1364.724	-1.12779	0.366184	-3.07984	0.002071	0.009721	FALSE	DOWN
STAU2	1247.043	-1.12338	0.343342	-3.27191	0.001068	0.005638	FALSE	DOWN
MAP3K3	3675.734	-1.12003	0.304987	-3.6724	0.00024	0.001621	FALSE	DOWN
MCM3AP	3586.258	-1.11996	0.292663	-3.8268	0.00013	0.000958	FALSE	DOWN
VPS39	4771.456	-1.11949	0.252033	-4.44184	8.92E-06	9.86E-05	FALSE	DOWN
ATG9A	3900.118	-1.11914	0.255715	-4.37651	1.21E-05	0.000129	FALSE	DOWN
RPUSD2	451.1162	-1.11705	0.287639	-3.88352	0.000103	0.000791	FALSE	DOWN
ERLEC1	3423.481	-1.11653	0.313844	-3.55759	0.000374	0.002324	FALSE	DOWN
RPA1	4919.221	-1.1141	0.299772	-3.7165	0.000202	0.001393	FALSE	DOWN
TMEM9	4317.487	-1.11315	0.336392	-3.30909	0.000936	0.005065	FALSE	DOWN
GBF1	4137.337	-1.11196	0.276237	-4.02539	5.69E-05	0.00048	FALSE	DOWN
NUP188	4019.21	-1.11137	0.318874	-3.48529	0.000492	0.002932	FALSE	DOWN
ENOPH1	1608.114	-1.11055	0.228025	-4.87032	1.11E-06	1.65E-05	FALSE	DOWN
ASCC3	1807.797	-1.1075	0.347577	-3.18635	0.001441	0.007145	FALSE	DOWN

CTNND1	8665.735	-1.10519	0.301228	-3.66894	0.000244	0.001638	FALSE	DOWN
SPTLC1	3269.994	-1.10376	0.333582	-3.3088	0.000937	0.005066	FALSE	DOWN
NUDCD3	5006.728	-1.10361	0.263339	-4.19082	2.78E-05	0.000263	FALSE	DOWN
CCDC117	1795.608	-1.10235	0.26922	-4.09461	4.23E-05	0.000373	FALSE	DOWN
GTDC1	943.5795	-1.10173	0.321807	-3.42357	0.000618	0.003572	FALSE	DOWN
TAF6	3527.5	-1.0999	0.321527	-3.42088	0.000624	0.003595	FALSE	DOWN
POLR2B	4052.889	-1.09904	0.25631	-4.28794	1.80E-05	0.000182	FALSE	DOWN
SOS1	2634.675	-1.09893	0.341868	-3.21448	0.001307	0.006618	FALSE	DOWN
SAE1	5891.655	-1.09814	0.302612	-3.62886	0.000285	0.001862	FALSE	DOWN
UROD	3606.386	-1.09595	0.326161	-3.36014	0.000779	0.004329	FALSE	DOWN
ATP6V1E1	5779.577	-1.09506	0.336015	-3.25897	0.001118	0.005843	FALSE	DOWN
ANKLE2	3010.853	-1.09263	0.264783	-4.12651	3.68E-05	0.000331	FALSE	DOWN
SETX	3926.532	-1.09154	0.275465	-3.96254	7.42E-05	0.000599	FALSE	DOWN
PLEKHB2	3632.911	-1.09114	0.346518	-3.14887	0.001639	0.00797	FALSE	DOWN
TEX2	2037.054	-1.0898	0.323056	-3.37342	0.000742	0.004159	FALSE	DOWN
NARS2	552.1447	-1.08951	0.320779	-3.39644	0.000683	0.003879	FALSE	DOWN
MAPK14	3288.87	-1.08831	0.291914	-3.7282	0.000193	0.001342	FALSE	DOWN
POM121	4297.747	-1.08783	0.297619	-3.6551	0.000257	0.001716	FALSE	DOWN
SUN1	5504.359	-1.08439	0.313444	-3.45958	0.000541	0.003196	FALSE	DOWN
OGFOD1	1649.823	-1.08343	0.287219	-3.77214	0.000162	0.001159	FALSE	DOWN
MATR3	12838.84	-1.08026	0.20141	-5.3635	8.16E-08	1.64E-06	FALSE	DOWN
GOLGA3	5705.045	-1.07765	0.336925	-3.1985	0.001381	0.006935	FALSE	DOWN
BRF2	937.3522	-1.07716	0.256034	-4.20709	2.59E-05	0.000249	FALSE	DOWN
JAK1	9508.319	-1.07473	0.300938	-3.57125	0.000355	0.002227	FALSE	DOWN
PINK1	5112.773	-1.07402	0.335136	-3.20473	0.001352	0.006813	FALSE	DOWN
ARMCX5	475.6978	-1.07239	0.336165	-3.19006	0.001422	0.007092	FALSE	DOWN
RFT1	924.0585	-1.0714	0.265173	-4.04039	5.34E-05	0.000455	FALSE	DOWN
RPRD1B	1726.956	-1.06967	0.280067	-3.81934	0.000134	0.000982	FALSE	DOWN
MSH6	2804.567	-1.06697	0.327215	-3.26076	0.001111	0.005812	FALSE	DOWN
TPD52L2	8741.571	-1.0664	0.265851	-4.01127	6.04E-05	0.000506	FALSE	DOWN
TFG	4123.78	-1.06438	0.237818	-4.47562	7.62E-06	8.62E-05	FALSE	DOWN
SMARCA2	6327.261	-1.06282	0.342759	-3.10078	0.00193	0.009173	FALSE	DOWN
APEX1	5482.828	-1.06264	0.275377	-3.85884	0.000114	0.00086	FALSE	DOWN
NFE2L1	21679.84	-1.06217	0.324636	-3.27187	0.001068	0.005638	FALSE	DOWN
COX15	1509.157	-1.06178	0.25712	-4.12952	3.64E-05	0.000327	FALSE	DOWN
TRMT5	835.1173	-1.05946	0.267756	-3.9568	7.60E-05	0.000611	FALSE	DOWN
FBXO18	3816.444	-1.05803	0.299826	-3.5288	0.000417	0.002547	FALSE	DOWN
EARS2	882.5641	-1.0565	0.328306	-3.21802	0.001291	0.006554	FALSE	DOWN
DDX18	3065.711	-1.05635	0.298928	-3.5338	0.00041	0.002513	FALSE	DOWN
MAP4	23459.57	-1.05635	0.301689	-3.50146	0.000463	0.002788	FALSE	DOWN
PDCD7	1243.237	-1.05542	0.281965	-3.74309	0.000182	0.001276	FALSE	DOWN
PPP2R1A	13302.4	-1.05453	0.315185	-3.34573	0.000821	0.004531	FALSE	DOWN
IPO8	2579.843	-1.05327	0.310816	-3.38871	0.000702	0.003966	FALSE	DOWN
AATF	2602.77	-1.04868	0.271784	-3.8585	0.000114	0.000861	FALSE	DOWN
NCOA6	2963.974	-1.04867	0.273569	-3.83329	0.000126	0.000936	FALSE	DOWN
ZMPSTE24	3115.683	-1.04803	0.267541	-3.91728	8.96E-05	0.0007	FALSE	DOWN
IFT52	1961.422	-1.04798	0.296734	-3.5317	0.000413	0.002528	FALSE	DOWN
PPP1R10	4566.323	-1.04641	0.296188	-3.53293	0.000411	0.002519	FALSE	DOWN

UQCRC2	5961.472	-1.04637	0.305134	-3.42922	0.000605	0.003509	FALSE	DOWN
SPATA7	436.4261	-1.04588	0.323962	-3.22839	0.001245	0.006379	FALSE	DOWN
BLOC1S3	779.2341	-1.04414	0.316841	-3.29547	0.000983	0.00528	FALSE	DOWN
EHMT1	2686.54	-1.04247	0.295229	-3.53108	0.000414	0.002533	FALSE	DOWN
PRPF4	1592.92	-1.04241	0.279524	-3.72923	0.000192	0.001339	FALSE	DOWN
ATP6AP2	9377.347	-1.0418	0.299486	-3.47863	0.000504	0.002998	FALSE	DOWN
LRRC47	2840.346	-1.0408	0.278203	-3.74117	0.000183	0.001284	FALSE	DOWN
NUFIP2	3575.033	-1.0399	0.273571	-3.80122	0.000144	0.001047	FALSE	DOWN
NSL1	1144.855	-1.03867	0.254464	-4.08181	4.47E-05	0.000391	FALSE	DOWN
KPNA1	3083.129	-1.03819	0.232554	-4.46431	8.03E-06	8.98E-05	FALSE	DOWN
GPATCH8	2322.146	-1.03812	0.307511	-3.37589	0.000736	0.004125	FALSE	DOWN
TM9SF4	5222.178	-1.03683	0.315925	-3.28188	0.001031	0.005468	FALSE	DOWN
RABGEF1	2127.117	-1.03561	0.242145	-4.27682	1.90E-05	0.00019	FALSE	DOWN
GLO1	4534.106	-1.03455	0.325971	-3.17376	0.001505	0.007404	FALSE	DOWN
THUMPD1	2303.64	-1.03131	0.330768	-3.11792	0.001821	0.008708	FALSE	DOWN
CANX	29173.3	-1.03121	0.314312	-3.28083	0.001035	0.005483	FALSE	DOWN
SNX3	8725.228	-1.02905	0.287546	-3.57873	0.000345	0.002175	FALSE	DOWN
SPSB3	2087.214	-1.0286	0.310064	-3.31739	0.000909	0.00494	FALSE	DOWN
CNNM3	1574.343	-1.028	0.316071	-3.25243	0.001144	0.005952	FALSE	DOWN
RRN3	2421.502	-1.02555	0.287114	-3.57194	0.000354	0.002224	FALSE	DOWN
SPG11	2384.556	-1.0223	0.270233	-3.78305	0.000155	0.001116	FALSE	DOWN
SECISBP2	1397.056	-1.02188	0.310732	-3.28863	0.001007	0.005364	FALSE	DOWN
DHX29	1807.806	-1.02132	0.273898	-3.72885	0.000192	0.00134	FALSE	DOWN
GEMIN4	1202.506	-1.02026	0.32676	-3.12236	0.001794	0.008604	FALSE	DOWN
DPH2	837.0236	-1.01982	0.324933	-3.13855	0.001698	0.0082	FALSE	DOWN
RNF167	4814.294	-1.01719	0.320181	-3.17692	0.001488	0.00734	FALSE	DOWN
MRPS34	2041.073	-1.01429	0.306751	-3.30654	0.000945	0.005099	FALSE	DOWN
HNRNPH3	5312.311	-1.01324	0.226507	-4.47333	7.70E-06	8.68E-05	FALSE	DOWN
ST7	1334.909	-1.01179	0.301733	-3.35327	0.000799	0.004422	FALSE	DOWN
CDIPT	5397.786	-1.00982	0.246074	-4.10373	4.07E-05	0.00036	FALSE	DOWN
RHOA	28545.78	-1.00736	0.277893	-3.62498	0.000289	0.001884	FALSE	DOWN
RING1	2284.872	-1.00429	0.317018	-3.16792	0.001535	0.007538	FALSE	DOWN
SIRT3	1234.334	-1.00405	0.308527	-3.25433	0.001137	0.005921	FALSE	DOWN
ZDHHC7	2829.389	-1.00253	0.304672	-3.29053	0.001	0.005338	FALSE	DOWN

gene_symb	delabel
USP9Y	USP9Y
NKX2.5	NKX2.5
SPRR2F	SPRR2F
CLEC3A	CLEC3A
MAGEB2	MAGEB2
MAGEA3	MAGEA3
MAGEA6	MAGEA6
CYP2W1	CYP2W1
RNASE1	RNASE1
CALCOCO2	CALCOCO2
PLA2G2A	PLA2G2A
A2M	A2M
MYOM1	MYOM1
CPVL	CPVL
NIPSNAP3A	NIPSNAP3A
FPR3	FPR3
H3F3C	H3F3C
SCUBE3	SCUBE3
WFDC2	WFDC2
UBE2D4	UBE2D4
MYH11	MYH11
SCRG1	SCRG1
METTL7B	METTL7B
RGN	RGN
IGSF21	IGSF21
LNP1	LNP1
LRRC37A	LRRC37A
AMY2B	AMY2B
GSTM3	GSTM3
HOXB13	HOXB13
C7	C7
CCDC113	CCDC113
SPESP1	SPESP1
PCDHA6	PCDHA6
LDLRAD2	LDLRAD2
SCG5	SCG5
CCDC121	CCDC121
LBH	LBH
LRRC25	LRRC25
NMUR1	NMUR1
RPE65	RPE65
FMO3	FMO3
GPBAR1	GPBAR1
ADRA2C	ADRA2C
CYTL1	CYTL1
LMOD1	LMOD1
ENAM	ENAM

PNMAL1	PNMAL1
SLC2A5	SLC2A5
CPZ	CPZ
ALDOB	ALDOB
RAB17	RAB17
TMSB15A	TMSB15A
CTSG	CTSG
IL22RA1	IL22RA1
DUOXA2	DUOXA2
NRSN2	NRSN2
CDRT4	CDRT4
SPTLC3	SPTLC3
IFI44L	IFI44L
BEX4	BEX4
SERPINA5	SERPINA5
PCDHA3	PCDHA3
KCNK1	KCNK1
TRIM38	TRIM38
TMEM59L	TMEM59L
PTGDS	PTGDS
STMN3	STMN3
ATM	ATM
DEFB1	DEFB1
NAT1	NAT1
CD70	CD70
SPINK5	SPINK5
SCG2	SCG2
CLDN4	CLDN4
LYPD3	LYPD3
TFF3	TFF3
GPAT2	GPAT2
LDOC1	LDOC1
C2CD4B	C2CD4B
CCL26	CCL26
PM20D2	PM20D2
NOXA1	NOXA1
PCDHA1	PCDHA1
PTP4A1	PTP4A1
FAM162B	FAM162B
MYL12B	MYL12B
FFAR3	FFAR3
CFD	CFD
PABPC5	PABPC5
PERP	PERP
IQCG	IQCG
NUPL1	NUPL1
TMEM217	TMEM217
MYLK	MYLK

PRIMA1	PRIMA1
IDO1	IDO1
ST6GAL2	ST6GAL2
ALDH1B1	ALDH1B1
KCNG1	KCNG1
HAPLN3	HAPLN3
CCR6	CCR6
SPATA20	SPATA20
ANKRD22	ANKRD22
KLRB1	KLRB1
09-Mar	09-Mar
CPM	CPM
CYBRD1	CYBRD1
MUC20	MUC20
NAP1L3	NAP1L3
CLDN14	CLDN14
CRTAC1	CRTAC1
IGF2	IGF2
ACBD7	ACBD7
FBP1	FBP1
RERGL	RERGL
ALPL	ALPL
FATE1	FATE1
CFI	CFI
SPOCK1	SPOCK1
FCGBP	FCGBP
XRRA1	XRRA1
MFRP	MFRP
LYG1	LYG1
PNMA1	PNMA1
TCF21	TCF21
APOC1	APOC1
CRISPLD1	CRISPLD1
KLHL6	KLHL6
HSD11B2	HSD11B2
SLC17A7	SLC17A7
SLC27A2	SLC27A2
FHL5	FHL5
PARM1	PARM1
DLX5	DLX5
ACOX2	ACOX2
TNFAIP8L3	TNFAIP8L3
TMEM54	TMEM54
AOC3	AOC3
TMEM30B	TMEM30B
BTN2A2	BTN2A2
BEX2	BEX2
KEL	KEL

S100A2	S100A2
GRIN2C	GRIN2C
TAF7L	TAF7L
FAM198A	FAM198A
PGM5	PGM5
MSMP	MSMP
OLFML1	OLFML1
SPOCD1	SPOCD1
TDRD6	TDRD6
CD163L1	CD163L1
UBE2NL	UBE2NL
PLA2G5	PLA2G5
CNTNAP3	CNTNAP3
FAM107A	FAM107A
RARRES1	RARRES1
CCDC81	CCDC81
NKX3.1	NKX3.1
DNALI1	DNALI1
GPR1	GPR1
KCNJ12	KCNJ12
FAM122C	FAM122C
ALDH1A3	ALDH1A3
CIDEB	CIDEB
SPATA18	SPATA18
LCN10	LCN10
CYP1B1	CYP1B1
NTRK1	NTRK1
SYNPO2	SYNPO2
CLSTN3	CLSTN3
EMILIN3	EMILIN3
FST	FST
KCTD20	KCTD20
VSIG4	VSIG4
SLC25A1	SLC25A1
DYRK4	DYRK4
TNFSF4	TNFSF4
PATL2	PATL2
UHRF1BP1	UHRF1BP1
KCNMB1	KCNMB1
EYA2	EYA2
MDH1B	MDH1B
TREX2	TREX2
TPPP	TPPP
HRH1	HRH1
TBX19	TBX19
FKBP5	FKBP5
PPIL6	PPIL6
HS3ST2	HS3ST2

SYNM	SYNM
KRT86	KRT86
RSPO3	RSPO3
MST1	MST1
TFAP2E	TFAP2E
PTPRU	PTPRU
LIMS2	LIMS2
FAM24B	FAM24B
OLFM2	OLFM2
RIBC2	RIBC2
ACTG2	ACTG2
CATSPER1	CATSPER1
GPC3	GPC3
CPS1	CPS1
SLC24A1	SLC24A1
CCDC146	CCDC146
SFRP1	SFRP1
MAOB	MAOB
HSD17B6	HSD17B6
SNURF	SNURF
TPSAB1	TPSAB1
TNXB	TNXB
RBP7	RBP7
CLEC4F	CLEC4F
CELF6	CELF6
LY6K	LY6K
SYTL3	SYTL3
PTGER3	PTGER3
ELF3	ELF3
MAP7D3	MAP7D3
SMOC1	SMOC1
QPRT	QPRT
PCDHB2	PCDHB2
ARHGEF37	ARHGEF37
TCEAL3	TCEAL3
HSPB6	HSPB6
LMO3	LMO3
HNMT	HNMT
SNCG	SNCG
HRASLS5	HRASLS5
RASD2	RASD2
SAMD11	SAMD11
CITED4	CITED4
CD300C	CD300C
SDSL	SDSL
PIPOX	PIPOX
CBS	CBS
DVL3	DVL3

FAM50B	FAM50B
PNMA2	PNMA2
LAMA3	LAMA3
NINJ2	NINJ2
PCDHB10	PCDHB10
TEKT4	TEKT4
TOX2	TOX2
HIST1H2BK	HIST1H2BK
SATL1	SATL1
P2RX1	P2RX1
CLEC3B	CLEC3B
ROBO3	ROBO3
PCDHB4	PCDHB4
PPARD	PPARD
ZSCAN18	ZSCAN18
PCDHB12	PCDHB12
STYXL1	STYXL1
TMEM178	TMEM178
C1QL1	C1QL1
WFDC1	WFDC1
AASS	AASS
HRNR	HRNR
MYL9	MYL9
CD163	CD163
PPP1R14A	PPP1R14A
TCEAL7	TCEAL7
VMO1	VMO1
EFHC2	EFHC2
IDI2	IDI2
NAGS	NAGS
MAPK13	MAPK13
PPP2R2B	PPP2R2B
CMTM1	CMTM1
GPNMB	GPNMB
SUSD5	SUSD5
LRRC66	LRRC66
FUT1	FUT1
CCDC69	CCDC69
ATF7IP2	ATF7IP2
RIMKLB	RIMKLB
RIMS4	RIMS4
EPHX2	EPHX2
SLC40A1	SLC40A1
NHLRC4	NHLRC4
PLIN4	PLIN4
TAF7	TAF7
RIMKLA	RIMKLA
SLC47A1	SLC47A1

FBXL7	FBXL7
HEY2	HEY2
TSSK3	TSSK3
ADCY9	ADCY9
RPP25	RPP25
BGLAP	BGLAP
CCDC80	CCDC80
KLHDC9	KLHDC9
HFM1	HFM1
SNRPN	SNRPN
VWF	VWF
HEPH	HEPH
JAZF1	JAZF1
LONRF2	LONRF2
GNA14	GNA14
CERCAM	CERCAM
PLB1	PLB1
PRSS23	PRSS23
RARB	RARB
CCDC110	CCDC110
SORBS1	SORBS1
NTF3	NTF3
RRAGB	RRAGB
SLC35B1	SLC35B1
SORT1	SORT1
HOXB7	HOXB7
CNTFR	CNTFR
PCDHB8	PCDHB8
CD4	CD4
ALDH1A1	ALDH1A1
FCER1A	FCER1A
CYB5R2	CYB5R2
MRVI1	MRVI1
CACNA1H	CACNA1H
GIPC3	GIPC3
VSTM2L	VSTM2L
CYFIP2	CYFIP2
CHRDL1	CHRDL1
USP11	USP11
AKAP12	AKAP12
ANKDD1A	ANKDD1A
CD36	CD36
LRRC3	LRRC3
SLC7A9	SLC7A9
PPM1N	PPM1N
LRP3	LRP3
SYNC	SYNC
HRCT1	HRCT1

HIF3A	HIF3A
MMP24	MMP24
ENPEP	ENPEP
SLC35F2	SLC35F2
CCDC89	CCDC89
TCEAL6	TCEAL6
ETV2	ETV2
ST6GALNAC	ST6GALNAC5
A1BG	A1BG
COLEC11	COLEC11
RNF112	RNF112
TRIM29	TRIM29
CLU	CLU
RASL11A	RASL11A
CSRP1	CSRP1
PLA2G4C	PLA2G4C
PPARA	PPARA
ANKRD36	ANKRD36
ATP6AP1L	ATP6AP1L
IFITM1	IFITM1
FN3K	FN3K
SCUBE2	SCUBE2
SENP5	SENP5
STOX1	STOX1
LRRN2	LRRN2
CYP26B1	CYP26B1
NAP1L5	NAP1L5
PRKDC	PRKDC
TEAD3	TEAD3
GDF5	GDF5
TMEM102	TMEM102
EPHA4	EPHA4
CD46	CD46
PCDHB3	PCDHB3
SPON2	SPON2
BCAM	BCAM
TAF9B	TAF9B
FRAS1	FRAS1
EFCAB1	EFCAB1
MDM2	MDM2
RASL12	RASL12
GIMAP5	GIMAP5
EID3	EID3
INMT	INMT
PCDHB11	PCDHB11
FRZB	FRZB
ZP3	ZP3
OPRL1	OPRL1

IGFBP5	IGFBP5
EPAS1	EPAS1
TSPAN1	TSPAN1
NXPH3	NXPH3
SEZ6L2	SEZ6L2
CNTN4	CNTN4
SUSD4	SUSD4
RSC1A1	RSC1A1
RASD1	RASD1
HSBP1L1	HSBP1L1
TMTC1	TMTC1
CHRNE	CHRNE
FAM13A	FAM13A
OS9	OS9
GPC6	GPC6
MYOZ3	MYOZ3
ELN	ELN
NRIP3	NRIP3
SLC19A3	SLC19A3
TYMP	TYMP
ACSM3	ACSM3
PHYHIP	PHYHIP
CEL	CEL
GLT8D2	GLT8D2
CCDC13	CCDC13
NGFR	NGFR
CD55	CD55
GJD3	GJD3
TNFRSF19	TNFRSF19
SETMAR	SETMAR
EPOR	EPOR
LNX1	LNX1
LDHB	LDHB
SLC8A1	SLC8A1
ITGB5	ITGB5
OPTN	OPTN
SDS	SDS
FOXD1	FOXD1
CCIN	CCIN
SVEP1	SVEP1
FABP3	FABP3
ARNT2	ARNT2
LRRC24	LRRC24
TRPV1	TRPV1
LIMCH1	LIMCH1
ANKRD53	ANKRD53
EIF2B5	EIF2B5
ACOT1	ACOT1

BAALC	BAALC
CADPS2	CADPS2
SPARCL1	SPARCL1
SLC2A12	SLC2A12
GGT1	GGT1
PCDHB6	PCDHB6
NOTCH3	NOTCH3
PCDH12	PCDH12
GSTK1	GSTK1
TFPI	TFPI
RTN4R	RTN4R
SLC22A18	SLC22A18
TUB	TUB
PTPRF	PTPRF
TMEM25	TMEM25
CDCP1	CDCP1
CLEC14A	CLEC14A
CELSR3	CELSR3
QRICH2	QRICH2
COL12A1	COL12A1
GPR3	GPR3
MAPK10	MAPK10
ASB9	ASB9
C1RL	C1RL
ABHD3	ABHD3
CCDC92	CCDC92
MESP1	MESP1
MEGF6	MEGF6
BTBD6	BTBD6
UPK3BL	UPK3BL
PLD6	PLD6
THSD4	THSD4
SLC25A29	SLC25A29
PELI3	PELI3
GCA	GCA
MCAM	MCAM
SNPH	SNPH
FAM89A	FAM89A
PTAR1	PTAR1
ANKRD12	ANKRD12
MAMDC2	MAMDC2
MX2	MX2
HDHD3	HDHD3
SOX13	SOX13
F8	F8
NPAS2	NPAS2
WASF3	WASF3
C1QTNF1	C1QTNF1

PXMP2	PXMP2
TAF9	TAF9
RARRES2	RARRES2
FAM184A	FAM184A
BBS10	BBS10
POPDC2	POPDC2
SLC4A5	SLC4A5
RPL27	RPL27
KRT222	KRT222
RAVER2	RAVER2
H1FX	H1FX
STK38	STK38
AGMAT	AGMAT
NECAB3	NECAB3
HSPA13	HSPA13
PRH1	PRH1
SEMA3G	SEMA3G
YWHAQ	YWHAQ
LARP6	LARP6
DPYD	DPYD
TMEM108	TMEM108
RAI2	RAI2
ZCCHC3	ZCCHC3
NGRN	NGRN
PCDHB9	PCDHB9
RBPMS	RBPMS
ZDHHC23	ZDHHC23
ITIH5	ITIH5
CNTNAP1	CNTNAP1
FBXO7	FBXO7
CLDN23	CLDN23
LZTS1	LZTS1
PTPRN2	PTPRN2
SPATS2L	SPATS2L
ATP8B3	ATP8B3
SNCA	SNCA
GJA4	GJA4
SCRN1	SCRN1
CMBL	CMBL
ADAMTS13	ADAMTS13
TSPAN18	TSPAN18
FXYD6	FXYD6
PPARG	PPARG
BDH1	BDH1
KCNS3	KCNS3
FGF13	FGF13
CDK18	CDK18
AMPH	AMPH

GALNT12	GALNT12
COL9A2	COL9A2
KLF15	KLF15
RPUSD3	RPUSD3
HIST1H2AC	HIST1H2AC
PDK4	PDK4
NPR1	NPR1
CHST3	CHST3
PLCXD1	PLCXD1
F2RL3	F2RL3
KLF12	KLF12
MOAP1	MOAP1
EGFLAM	EGFLAM
PAPLN	PAPLN
FHL1	FHL1
MANSC1	MANSC1
TMEM8B	TMEM8B
ETNK2	ETNK2
FAHD2A	FAHD2A
DLG4	DLG4
FLYWCH2	FLYWCH2
PCBD1	PCBD1
PCDHB7	PCDHB7
KBTBD6	KBTBD6
TNFSF10	TNFSF10
MALL	MALL
MYO5C	MYO5C
APLP1	APLP1
LRRC34	LRRC34
ATP6V1E2	ATP6V1E2
RAB33A	RAB33A
LGALS3BP	LGALS3BP
NAALADL1	NAALADL1
APOLD1	APOLD1
CIRBP	CIRBP
COL8A2	COL8A2
NECAB1	NECAB1
RNF208	RNF208
PRDM6	PRDM6
PDE5A	PDE5A
RPS12	RPS12
PLXNB1	PLXNB1
FILIP1	FILIP1
FGD5	FGD5
TMEM56	TMEM56
SPOCK2	SPOCK2
ABLIM3	ABLIM3
MGLL	MGLL

COL6A2	COL6A2
LAMA5	LAMA5
KANK1	KANK1
SYTL2	SYTL2
NNMT	NNMT
PSMF1	PSMF1
IL17RB	IL17RB
EFHD1	EFHD1
RECK	RECK
CCDC103	CCDC103
MFAP4	MFAP4
RNF125	RNF125
L3MBTL4	L3MBTL4
FADS2	FADS2
TCEAL1	TCEAL1
P4HTM	P4HTM
EFS	EFS
MYL6B	MYL6B
RPL5	RPL5
PPP1R3B	PPP1R3B
LYRM7	LYRM7
NIPSNAP1	NIPSNAP1
PRRT3	PRRT3
OPN3	OPN3
BRPF3	BRPF3
KCNE4	KCNE4
CCDC14	CCDC14
REC8	REC8
RBM4	RBM4
PLEKHG4	PLEKHG4
KLHL4	KLHL4
FBLN1	FBLN1
WFS1	WFS1
PYGL	PYGL
ARG2	ARG2
SH3BP5	SH3BP5
LPAR5	LPAR5
SCN4B	SCN4B
DAAM2	DAAM2
GAL3ST4	GAL3ST4
LRP11	LRP11
SMPD4	SMPD4
S1PR3	S1PR3
NFE2L3	NFE2L3
ARHGAP20	ARHGAP20
CD276	CD276
SLC45A1	SLC45A1
FAM20C	FAM20C

MRGPRF	MRGPRF
ALKBH7	ALKBH7
STOM	STOM
FBXO41	FBXO41
AKAP3	AKAP3
PPCS	PPCS
BBS1	BBS1
PRKG1	PRKG1
THRB	THRB
TST	TST
KCNIP3	KCNIP3
KANK2	KANK2
ADAR	ADAR
FAU	FAU
GSTA4	GSTA4
NCALD	NCALD
PIK3R3	PIK3R3
TSPYL5	TSPYL5
GRHPR	GRHPR
FHIT	FHIT
GUK1	GUK1
APOL6	APOL6
NME4	NME4
GGT7	GGT7
RNF144A	RNF144A
TSPAN15	TSPAN15
SPAG8	SPAG8
B4GALT4	B4GALT4
OSBPL10	OSBPL10
THAP8	THAP8
PEMT	PEMT
PLLP	PLLP
XPC	XPC
SVIP	SVIP
TSPYL4	TSPYL4
ASPRV1	ASPRV1
LDB2	LDB2
LRFN3	LRFN3
SLC41A3	SLC41A3
ASTN2	ASTN2
BOC	BOC
AADAT	AADAT
MGST3	MGST3
SLC16A14	SLC16A14
SPRN	SPRN
FBXO44	FBXO44
ACSS2	ACSS2
HSD17B1	HSD17B1

HEY1	HEY1
RAD23A	RAD23A
ACTR3B	ACTR3B
CCDC96	CCDC96
HEBP2	HEBP2
ACSL1	ACSL1
IL4I1	IL4I1
SMARCA1	SMARCA1
SLC46A1	SLC46A1
SPINT2	SPINT2
FZD4	FZD4
SPIN4	SPIN4
ARL4D	ARL4D
RCAN2	RCAN2
KLHL29	KLHL29
NUDT15	NUDT15
ATP9A	ATP9A
IFNGR2	IFNGR2
OAS3	OAS3
CLDN15	CLDN15
LEPR	LEPR
CREG1	CREG1
CABLES1	CABLES1
MX1	MX1
ITPK1	ITPK1
CTSF	CTSF
FAM122B	FAM122B
KRT10	KRT10
MSC	MSC
HSD17B14	HSD17B14
CHST10	CHST10
GBE1	GBE1
RPH3AL	RPH3AL
ROR1	ROR1
TBXA2R	TBXA2R
MAMLD1	MAMLD1
PPFIBP2	PPFIBP2
COX7A1	COX7A1
NUDT3	NUDT3
PGBD1	PGBD1
STIM1	STIM1
CREB3L4	CREB3L4
TRIM14	TRIM14
VCL	VCL
LYPLAL1	LYPLAL1
SARM1	SARM1
ATP5G2	ATP5G2
EPB41L4A	EPB41L4A

SLC37A1	SLC37A1
SRGAP1	SRGAP1
EFCAB7	EFCAB7
TIMP3	TIMP3
LRRC23	LRRC23
UBTD2	UBTD2
VASH1	VASH1
STK32C	STK32C
SHPK	SHPK
SLC10A3	SLC10A3
COQ4	COQ4
HAAO	HAAO
XRCC5	XRCC5
CLSTN1	CLSTN1
NQO1	NQO1
ESRP2	ESRP2
ABHD15	ABHD15
HSPB1	HSPB1
ST5	ST5
SELENBP1	SELENBP1
RAB40B	RAB40B
MAVS	MAVS
NFATC2IP	NFATC2IP
RBMX	RBMX
HOXB2	HOXB2
NUMA1	NUMA1
WDR34	WDR34
TERF2IP	TERF2IP
PLEKHA4	PLEKHA4
IKZF4	IKZF4
ATPAF1	ATPAF1
CCDC149	CCDC149
ANGEL1	ANGEL1
ARHGAP1	ARHGAP1
EFNB1	EFNB1
GLUL	GLUL
SLC2A10	SLC2A10
GPR4	GPR4
ARVCF	ARVCF
EPHX1	EPHX1
GPRIN1	GPRIN1
ODF3B	ODF3B
DIXDC1	DIXDC1
GNAI1	GNAI1
NEURL1B	NEURL1B
FAM198B	FAM198B
PRPS2	PRPS2
SUMF2	SUMF2

SPRY3	SPRY3
KIF7	KIF7
NID2	NID2
ECM2	ECM2
ZKSCAN4	ZKSCAN4
ICAM2	ICAM2
EXOSC6	EXOSC6
RAB23	RAB23
ARHGEF9	ARHGEF9
LYRM2	LYRM2
TMEM14A	TMEM14A
NDUFAF3	NDUFAF3
ADNP	ADNP
SCARB2	SCARB2
MAGED2	MAGED2
NAT14	NAT14
CHSY3	CHSY3
HIPK2	HIPK2
DDX60	DDX60
CALML4	CALML4
TNFAIP3	TNFAIP3
LMCD1	LMCD1
TMEM220	TMEM220
FAM131A	FAM131A
KLF9	KLF9
CALHM2	CALHM2
PDGFRB	PDGFRB
LMNB2	LMNB2
TUBG2	TUBG2
CABYR	CABYR
TMEM79	TMEM79
ICA1L	ICA1L
MMRN2	MMRN2
SLC43A1	SLC43A1
CCDC142	CCDC142
HESX1	HESX1
LARP1B	LARP1B
ENTPD6	ENTPD6
ABHD14B	ABHD14B
NPHP4	NPHP4
MED29	MED29
STAT5B	STAT5B
TXNIP	TXNIP
URB1	URB1
JAM3	JAM3
TIMP1	TIMP1
MID2	MID2
NCOA1	NCOA1

ACSS1	ACSS1
UNC13B	UNC13B
SLC25A43	SLC25A43
KCNC4	KCNC4
SGCD	SGCD
MSRB2	MSRB2
ILVBL	ILVBL
PGAP3	PGAP3
RWDD2B	RWDD2B
CRY2	CRY2
FAM102A	FAM102A
PVRIG	PVRIG
FBXW5	FBXW5
FUCA2	FUCA2
GIMAP7	GIMAP7
B3GALT6	B3GALT6
HOXB3	HOXB3
TMUB1	TMUB1
SLC27A3	SLC27A3
ADI1	ADI1
ANKS6	ANKS6
LMBR1	LMBR1
FAS	FAS
COL4A2	COL4A2
LIN7B	LIN7B
PHF19	PHF19
BET1L	BET1L
PLSCR4	PLSCR4
USP22	USP22
DCUN1D4	DCUN1D4
PRKD1	PRKD1
GAS6	GAS6
BTG2	BTG2
TACO1	TACO1
SNX29	SNX29
RAB27A	RAB27A
XRCC6	XRCC6
TYW3	TYW3
HDDC2	HDDC2
BCAP29	BCAP29
SPAG16	SPAG16
RPL10	RPL10
ERBB2	ERBB2
TTC3	TTC3
ZGLP1	ZGLP1
PER1	PER1
GYPC	GYPC
SAT2	SAT2

PCDHB14	PCDHB14
ESAM	ESAM
SGCB	SGCB
CCDC51	CCDC51
PDE2A	PDE2A
TMEM219	TMEM219
DENND3	DENND3
ZFP36	ZFP36
CNP	CNP
HOXA4	HOXA4
GSPT2	GSPT2
MPZL1	MPZL1
SEC61G	SEC61G
ANTXR1	ANTXR1
SAP18	SAP18
RAB42	RAB42
KCTD1	KCTD1
JAG1	JAG1
SGSH	SGSH
GALM	GALM
RCAN1	RCAN1
EMILIN1	EMILIN1
AHR	AHR
CYP2R1	CYP2R1
WARS	WARS
SLC22A17	SLC22A17
RHBDD2	RHBDD2
ILF3	ILF3
CRTC3	CRTC3
PYGB	PYGB
UXT	UXT
SYTL4	SYTL4
RETSAT	RETSAT
PPDPF	PPDPF
SCO2	SCO2
PDPR	PDPR
ADAMTS1	ADAMTS1
ELAC2	ELAC2
HS1BP3	HS1BP3
LAMA4	LAMA4
WDR1	WDR1
SUOX	SUOX
KCNMB3	KCNMB3
PEX10	PEX10
MAD2L2	MAD2L2
PHACTR2	PHACTR2
DDX24	DDX24
CHD1L	CHD1L

NEK11	NEK11
GBA2	GBA2
MKKS	MKKS
ITGB1BP1	ITGB1BP1
PRICKLE2	PRICKLE2
10-Sep	10-Sep
BTBD2	BTBD2
MORN2	MORN2
DPH1	DPH1
LYSMD4	LYSMD4
RAB4A	RAB4A
MPPE1	MPPE1
CCDC106	CCDC106
TCFL5	TCFL5
ECHDC1	ECHDC1
TNIP1	TNIP1
PACS1	PACS1
SLC39A8	SLC39A8
TRMT61A	TRMT61A
ZBED5	ZBED5
TLE4	TLE4
PRDX6	PRDX6
SLC5A6	SLC5A6
TMEM129	TMEM129
DTD1	DTD1
SLC43A3	SLC43A3
CASP7	CASP7
NUDT16	NUDT16
FOXRED2	FOXRED2
ZBTB8A	ZBTB8A
RPL10A	RPL10A
SNX22	SNX22
EXOC7	EXOC7
PML	PML
PLXNA2	PLXNA2
ZYX	ZYX
TMEM203	TMEM203
H2AFV	H2AFV
ACACB	ACACB
ICMT	ICMT
ACVR2B	ACVR2B
ANKAR	ANKAR
NCOA7	NCOA7
PMP22	PMP22
NME3	NME3
NAPEPLD	NAPEPLD
NT5DC1	NT5DC1
VWA1	VWA1

TMEM116	TMEM116
CDC25B	CDC25B
LPCAT1	LPCAT1
CDK5RAP1	CDK5RAP1
DCHS1	DCHS1
CYYR1	CYYR1
AGRN	AGRN
TECPR2	TECPR2
SDR39U1	SDR39U1
CIB1	CIB1
CASC4	CASC4
LPIN1	LPIN1
WDR6	WDR6
FAM53B	FAM53B
REPIN1	REPIN1
TBC1D1	TBC1D1
PER2	PER2
MXRA7	MXRA7
RPGR	RPGR
TRIM62	TRIM62
TUFM	TUFM
JRK	JRK
PQLC1	PQLC1
HINT3	HINT3
MEGF9	MEGF9
DCTD	DCTD
TM2D3	TM2D3
ZSWIM1	ZSWIM1
PDK2	PDK2
FAM120C	FAM120C
SEPHS2	SEPHS2
PCDHGC3	PCDHGC3
SIX5	SIX5
RSAD1	RSAD1
AGBL5	AGBL5
FAM71D	FAM71D
GALNT11	GALNT11
TMEM120E	TMEM120B
SUSD1	SUSD1
LRRC8A	LRRC8A
SLC12A7	SLC12A7
ALG3	ALG3
RBM12	RBM12
MBD4	MBD4
SNAI2	SNAI2
LRRC27	LRRC27
SGTB	SGTB
NUCKS1	NUCKS1

PJA2	PJA2
TMEM106C	TMEM106C
CRYL1	CRYL1
KCTD7	KCTD7
TMEM44	TMEM44
TMEM69	TMEM69
ADCK2	ADCK2
IDS	IDS
DDB2	DDB2
PMVK	PMVK
RDH11	RDH11
PPIF	PPIF
NRP1	NRP1
TNFRSF14	TNFRSF14
SIVA1	SIVA1
GDE1	GDE1
LAMB2	LAMB2
PLXND1	PLXND1
MAGEH1	MAGEH1
CCDC28A	CCDC28A
ANKRD42	ANKRD42
LRPAP1	LRPAP1
ECE1	ECE1
VEGFB	VEGFB
APH1B	APH1B
POLR2L	POLR2L
FAM160B2	FAM160B2
ACTB	ACTB
CD81	CD81
FAM50A	FAM50A
CLN6	CLN6
CLIC4	CLIC4
TBC1D5	TBC1D5
KLHDC8B	KLHDC8B
UBXN11	UBXN11
AHI1	AHI1
CD99L2	CD99L2
PLOD1	PLOD1
LBR	LBR
PLD3	PLD3
SNRNP200	SNRNP200
EID1	EID1
PCM1	PCM1
DTX3L	DTX3L
UBA52	UBA52
HSDL2	HSDL2
ARL6IP4	ARL6IP4
DMXL2	DMXL2

ARRDC2	ARRDC2
TAB3	TAB3
ATP6V1C2	ATP6V1C2
TCTN3	TCTN3
VPS13C	VPS13C
RNF135	RNF135
KLHL8	KLHL8
EIF2AK1	EIF2AK1
BNC2	BNC2
EHBP1	EHBP1
ATMIN	ATMIN
HDAC11	HDAC11
KDELR1	KDELR1
PGM1	PGM1
MKRN1	MKRN1
DTWD1	DTWD1
NBAS	NBAS
SNUPN	SNUPN
ERC1	ERC1
ARV1	ARV1
MFN2	MFN2
CCDC25	CCDC25
MANBAL	MANBAL
DYSF	DYSF
PIGT	PIGT
ACADVL	ACADVL
TMEM205	TMEM205
PRMT2	PRMT2
NKIRAS2	NKIRAS2
KDM4D	KDM4D
PDXK	PDXK
ANKRD13D	ANKRD13D
RABL3	RABL3
OSBPL1A	OSBPL1A
KIF3B	KIF3B
KCTD21	KCTD21
SLC25A35	SLC25A35
NAP1L1	NAP1L1
GNE	GNE
CD151	CD151
TRIM13	TRIM13
CUTC	CUTC
EEPD1	EEPD1
YEATS2	YEATS2
LRRC41	LRRC41
COMMD6	COMMD6
SLC25A25	SLC25A25
STAT5A	STAT5A

THADA	THADA
VGLL4	VGLL4
ZHX3	ZHX3
ZSWIM7	ZSWIM7
H6PD	H6PD
STARD7	STARD7
COX11	COX11
RAB11FIP2	RAB11FIP2
ORMDL1	ORMDL1
NEIL2	NEIL2
ZXDC	ZXDC
KLF11	KLF11
WASF1	WASF1
MED16	MED16
TBC1D9B	TBC1D9B
RECQL	RECQL
TRAM2	TRAM2
PMS1	PMS1
TMEM62	TMEM62
ELK1	ELK1
PPT1	PPT1
RNF185	RNF185
CRTAP	CRTAP
NADSYN1	NADSYN1
PARP4	PARP4
RARS2	RARS2
ARMC6	ARMC6
ST6GALNAC	ST6GALNAC6
MCCC2	MCCC2
SLC35D2	SLC35D2
SP100	SP100
SMPD1	SMPD1
SLC25A13	SLC25A13
DDT	DDT
TADA2B	TADA2B
ABHD10	ABHD10
CTDSPL	CTDSPL
RRM2B	RRM2B
POLDIP2	POLDIP2
SMAD2	SMAD2
GTF3C5	GTF3C5
SH3YL1	SH3YL1
MGAT1	MGAT1
ECH1	ECH1
COASY	COASY
ITPKC	ITPKC
ZER1	ZER1
GLOD4	GLOD4

SSU72	SSU72
BAG5	BAG5
ACSL3	ACSL3
PPP1CB	PPP1CB
NUP43	NUP43
RPA2	RPA2
KIF22	KIF22
ALDH9A1	ALDH9A1
TBC1D25	TBC1D25
VAMP2	VAMP2
ADCY3	ADCY3
TADA3	TADA3
STK40	STK40
YTHDC2	YTHDC2
HMGXB3	HMGXB3
MRPL16	MRPL16
TMEM109	TMEM109
MRPS27	MRPS27
DPYSL2	DPYSL2
NUCB1	NUCB1
RWDD2A	RWDD2A
RBL2	RBL2
NPHP3	NPHP3
MKS1	MKS1
TCEA2	TCEA2
PARP1	PARP1
QRSL1	QRSL1
HAUS4	HAUS4
WDR25	WDR25
DHRS7	DHRS7
AKAP13	AKAP13
ABI2	ABI2
YIF1A	YIF1A
PTTG1IP	PTTG1IP
BSDC1	BSDC1
DOCK1	DOCK1
L3MBTL2	L3MBTL2
RAB5B	RAB5B
KCTD3	KCTD3
PARN	PARN
RUSC1	RUSC1
CRY1	CRY1
CPNE1	CPNE1
MRPL40	MRPL40
OSTM1	OSTM1
MRPL37	MRPL37
ASAP2	ASAP2
TTLL3	TTLL3

NDUFS5	NDUFS5
GOPC	GOPC
ECHS1	ECHS1
PLCG1	PLCG1
PLSCR1	PLSCR1
CTNNAL1	CTNNAL1
RIN3	RIN3
NUPL2	NUPL2
ATN1	ATN1
CMIP	CMIP
PNPO	PNPO
TBCD	TBCD
SF3A1	SF3A1
NUMB	NUMB
GAB2	GAB2
CNTROB	CNTROB
PRKCSH	PRKCSH
RAB2B	RAB2B
DUSP22	DUSP22
DHTKD1	DHTKD1
GPAA1	GPAA1
FAHD1	FAHD1
CRAT	CRAT
WDTC1	WDTC1
TPP1	TPP1
STX17	STX17
PEF1	PEF1
IPP	IPP
SLC25A38	SLC25A38
CD320	CD320
TSR2	TSR2
FAM122A	FAM122A
FAM3A	FAM3A
ZC3H6	ZC3H6
EEFSEC	EEFSEC
PHTF2	PHTF2
KDSR	KDSR
PTGES2	PTGES2
UBE2A	UBE2A
CREBBP	CREBBP
ANXA11	ANXA11
MPST	MPST
SNX19	SNX19
PEPD	PEPD
TESK1	TESK1
MADD	MADD
PCCB	PCCB
RNF216	RNF216

TMEM115	TMEM115
MLH1	MLH1
DAXX	DAXX
PRKACA	PRKACA
TMEM42	TMEM42
NCOA4	NCOA4
HADHA	HADHA
MEAF6	MEAF6
ADD1	ADD1
SPEN	SPEN
WDR13	WDR13
UBIAD1	UBIAD1
PRKD2	PRKD2
LRP10	LRP10
HSPB11	HSPB11
UBR7	UBR7
HTATSF1	HTATSF1
MORC4	MORC4
SETDB2	SETDB2
MRPS2	MRPS2
ZYG11B	ZYG11B
ZFYVE21	ZFYVE21
SGSM3	SGSM3
AP3S2	AP3S2
RIC8A	RIC8A
CPNE3	CPNE3
MUL1	MUL1
EHD2	EHD2
GTF3C1	GTF3C1
TRAF3IP2	TRAF3IP2
PCTP	PCTP
UBAP1	UBAP1
UROS	UROS
POP7	POP7
ST3GAL3	ST3GAL3
HDDC3	HDDC3
ERCC1	ERCC1
PI4KA	PI4KA
EIF2AK4	EIF2AK4
TMEM43	TMEM43
WDR89	WDR89
DHPS	DHPS
MAML1	MAML1
DGCR2	DGCR2
IGHMBP2	IGHMBP2
PRDX3	PRDX3
MAPKAP1	MAPKAP1
RFXANK	RFXANK

RAF1	RAF1
ACSF3	ACSF3
PATZ1	PATZ1
PHF8	PHF8
PLEKHM2	PLEKHM2
METTL4	METTL4
MPG	MPG
UBE2Q2	UBE2Q2
SLC39A9	SLC39A9
CBLL1	CBLL1
BBS2	BBS2
DDX19B	DDX19B
SERTAD3	SERTAD3
ACTR6	ACTR6
AP1M1	AP1M1
APLP2	APLP2
ALAD	ALAD
TTC23	TTC23
YPEL2	YPEL2
PKN1	PKN1
PREPL	PREPL
VPS45	VPS45
MTMR12	MTMR12
LRPPRC	LRPPRC
CBR1	CBR1
AK3	AK3
BBS5	BBS5
GANAB	GANAB
MGRN1	MGRN1
RNF40	RNF40
PPP1R3D	PPP1R3D
CHID1	CHID1
PRPS1	PRPS1
INTS10	INTS10
VPS4A	VPS4A
GLG1	GLG1
ULK3	ULK3
DDX56	DDX56
CHMP1B	CHMP1B
FANCG	FANCG
SYDE1	SYDE1
CUL4B	CUL4B
ARHGAP17	ARHGAP17
RFX5	RFX5
CIAO1	CIAO1
CHURC1	CHURC1
MED8	MED8
AP2B1	AP2B1

PRKRA	PRKRA
CLEC16A	CLEC16A
WBP2	WBP2
HMGCL	HMGCL
WIZ	WIZ
DCTN1	DCTN1
SLC35E1	SLC35E1
CD164	CD164
SIN3A	SIN3A
NDUFB4	NDUFB4
HMGN2	HMGN2
TIGD5	TIGD5
ZFP36L1	ZFP36L1
RBMX2	RBMX2
SCAMP4	SCAMP4
TXLNA	TXLNA
AP2A1	AP2A1
ERCC5	ERCC5
SORBS3	SORBS3
ZNFX1	ZNFX1
JMJD8	JMJD8
KDELC2	KDELC2
TMEM59	TMEM59
MLST8	MLST8
TMEM101	TMEM101
TMEM138	TMEM138
RRAGA	RRAGA
BAP1	BAP1
RAD1	RAD1
IARS2	IARS2
RSL24D1	RSL24D1
COIL	COIL
KLHL12	KLHL12
SUPT7L	SUPT7L
ZBTB4	ZBTB4
WASL	WASL
HADHB	HADHB
MTR	MTR
ANAPC16	ANAPC16
EXOC4	EXOC4
USP20	USP20
STK24	STK24
NBN	NBN
STAT3	STAT3
TAF1C	TAF1C
TRIM68	TRIM68
SCAMP1	SCAMP1
POMT1	POMT1

HNRNPUL1	HNRNPUL1
SEC16A	SEC16A
OXA1L	OXA1L
CCDC130	CCDC130
TUBGCP2	TUBGCP2
OGDH	OGDH
PDE8A	PDE8A
UBAC2	UBAC2
SEC22C	SEC22C
ERAL1	ERAL1
SYNRG	SYNRG
OAZ1	OAZ1
NOMO1	NOMO1
MAPK1	MAPK1
NPLOC4	NPLOC4
PSMD10	PSMD10
VAMP3	VAMP3
MLX	MLX
HGSNAT	HGSNAT
SRP14	SRP14
UBOX5	UBOX5
ASCC1	ASCC1
GPBP1L1	GPBP1L1
EIF3L	EIF3L
SCAP	SCAP
SCCPDH	SCCPDH
IMPAD1	IMPAD1
WASF2	WASF2
BRWD1	BRWD1
CASC3	CASC3
METTL5	METTL5
TINF2	TINF2
SF3A2	SF3A2
WDR82	WDR82
POLR1E	POLR1E
STAU2	STAU2
MAP3K3	MAP3K3
MCM3AP	MCM3AP
VPS39	VPS39
ATG9A	ATG9A
RPUSD2	RPUSD2
ERLEC1	ERLEC1
RPA1	RPA1
TMEM9	TMEM9
GBF1	GBF1
NUP188	NUP188
ENOPH1	ENOPH1
ASCC3	ASCC3

CTNND1	CTNND1
SPTLC1	SPTLC1
NUDCD3	NUDCD3
CCDC117	CCDC117
GTDC1	GTDC1
TAF6	TAF6
POLR2B	POLR2B
SOS1	SOS1
SAE1	SAE1
UROD	UROD
ATP6V1E1	ATP6V1E1
ANKLE2	ANKLE2
SETX	SETX
PLEKHB2	PLEKHB2
TEX2	TEX2
NARS2	NARS2
MAPK14	MAPK14
POM121	POM121
SUN1	SUN1
OGFOD1	OGFOD1
MATR3	MATR3
GOLGA3	GOLGA3
BRF2	BRF2
JAK1	JAK1
PINK1	PINK1
ARMCX5	ARMCX5
RFT1	RFT1
RPRD1B	RPRD1B
MSH6	MSH6
TPD52L2	TPD52L2
TFG	TFG
SMARCA2	SMARCA2
APEX1	APEX1
NFE2L1	NFE2L1
COX15	COX15
TRMT5	TRMT5
FBXO18	FBXO18
EARS2	EARS2
DDX18	DDX18
MAP4	MAP4
PDCD7	PDCD7
PPP2R1A	PPP2R1A
IPO8	IPO8
AATF	AATF
NCOA6	NCOA6
ZMPSTE24	ZMPSTE24
IFT52	IFT52
PPP1R10	PPP1R10

UQCRC2	UQCRC2
SPATA7	SPATA7
BLOC1S3	BLOC1S3
EHMT1	EHMT1
PRPF4	PRPF4
ATP6AP2	ATP6AP2
LRRC47	LRRC47
NUFIP2	NUFIP2
NSL1	NSL1
KPNA1	KPNA1
GPATCH8	GPATCH8
TM9SF4	TM9SF4
RABGEF1	RABGEF1
GLO1	GLO1
THUMPD1	THUMPD1
CANX	CANX
SNX3	SNX3
SPSB3	SPSB3
CNNM3	CNNM3
RRN3	RRN3
SPG11	SPG11
SECISBP2	SECISBP2
DHX29	DHX29
GEMIN4	GEMIN4
DPH2	DPH2
RNF167	RNF167
MRPS34	MRPS34
HNRNPH3	HNRNPH3
ST7	ST7
CDIPT	CDIPT
RHOA	RHOA
RING1	RING1
SIRT3	SIRT3
ZDHHC7	ZDHHC7
