# Supplementary material for: The KrasG12D;Trp53fl/fl murine model of undifferentiated pleomorphic sarcoma is macrophage dense, lymphocyte poor, and resistant to immune checkpoint blockade
Source: PLoS One. 2021 Jul 9;16(7):e0253864. doi: 10.1371/journal.pone.0253864 (PMC8270133; doi:10.1371/journal.pone.0253864)
Supplement: S2 File — Complete list of differentially upregulated murine genes relative to human UPS. (PDF) [file pone.0253864.s002.pdf]

	baseMean	log2FoldCh	lfcSE	stat	pvalue	padj	threshold	diffexpresse
MYH4	517.1944	13.00078	1.843487	7.052279	1.76E-12	1.02E-10	TRUE	UP
RPL21	1258.383	11.95812	0.761919	15.69474	1.64E-55	7.58E-52	TRUE	UP
CD300LD	26.55845	11.94945	3.211301	3.721061	0.000198	0.001375	TRUE	UP
S100A7A	23.56248	11.80596	3.295141	3.582839	0.00034	0.002146	TRUE	UP
GGNBP1	16.04271	11.24989	3.026917	3.716618	0.000202	0.001393	TRUE	UP
GPR149	26.28002	11.00762	2.547177	4.321497	1.55E-05	0.00016	TRUE	UP
GFRA4	15.82366	10.79958	3.416139	3.161342	0.00157	0.007681	TRUE	UP
CELA1	21.02614	10.3368	1.07773	9.591267	8.70E-22	1.70E-19	TRUE	UP
GNG8	116.6243	10.1912	1.216618	8.376661	5.45E-17	5.89E-15	TRUE	UP
CACNG2	9.050486	10.13655	2.221518	4.562895	5.05E-06	6.07E-05	TRUE	UP
ZAN	8.519595	10.10631	2.295659	4.402358	1.07E-05	0.000117	TRUE	UP
TRIML1	11.07692	9.619109	2.040506	4.71408	2.43E-06	3.21E-05	TRUE	UP
CEACAM20	9.158946	9.562811	2.646133	3.613881	0.000302	0.001953	TRUE	UP
RAG2	5.505354	9.46203	2.376786	3.981018	6.86E-05	0.000564	TRUE	UP
GBP7	54.06665	9.434874	1.109408	8.504424	1.82E-17	2.09E-15	TRUE	UP
RTP3	14.89388	9.393099	2.64695	3.54865	0.000387	0.002395	TRUE	UP
BRDT	7.558001	9.345251	2.529382	3.694677	0.00022	0.001504	TRUE	UP
PRAMEF8	16.10381	9.336302	2.909358	3.209059	0.001332	0.006725	TRUE	UP
EXD1	17.98281	9.298585	1.719923	5.406396	6.43E-08	1.33E-06	TRUE	UP
MYL1	181.0843	9.230241	2.978405	3.099055	0.001941	0.009212	TRUE	UP
GRK1	45.92838	9.139126	2.233327	4.092159	4.27E-05	0.000377	TRUE	UP
PF4	50.28801	9.10754	1.534564	5.934935	2.94E-09	8.77E-08	TRUE	UP
PDE6H	9.798556	9.026157	1.299087	6.948076	3.70E-12	2.03E-10	TRUE	UP
ART1	9.889506	9.019029	2.539874	3.550975	0.000384	0.002375	TRUE	UP
ACTN3	133.3976	8.980145	0.8561	10.4896	9.64E-26	2.78E-23	TRUE	UP
MYLPF	289.3247	8.950395	1.709949	5.234304	1.66E-07	3.06E-06	TRUE	UP
MIA2	97.31491	8.894685	2.725032	3.264066	0.001098	0.005757	TRUE	UP
LARS2	12156.76	8.642727	0.365501	23.64623	1.29E-123	1.79E-119	TRUE	UP
LRTM1	5.240036	8.550874	1.711896	4.994973	5.88E-07	9.33E-06	TRUE	UP
STARD6	8.855078	8.55014	1.23546	6.920614	4.50E-12	2.37E-10	TRUE	UP
FAM58B	25.04615	8.548617	0.582502	14.67569	9.23E-49	1.60E-45	TRUE	UP
CKM	351.7161	8.547943	1.353085	6.317374	2.66E-10	1.01E-08	TRUE	UP
CST6	65.58163	8.533362	1.214621	7.025534	2.13E-12	1.22E-10	TRUE	UP
CR1L	59.21581	8.532224	1.264003	6.750162	1.48E-11	7.05E-10	TRUE	UP
TMEM88B	4.260662	8.41592	2.245041	3.748672	0.000178	0.001252	TRUE	UP
BOLL	3.68241	8.352311	2.561719	3.260433	0.001112	0.005817	TRUE	UP
KLHL33	94.04731	8.298749	1.38918	5.973846	2.32E-09	7.03E-08	TRUE	UP
PKD2L2	19.02948	8.246322	0.777178	10.6106	2.66E-26	8.00E-24	TRUE	UP
EPB42	6.711683	8.156858	2.243044	3.636513	0.000276	0.001816	TRUE	UP
GPR45	16.99056	8.152782	1.008916	8.080734	6.44E-16	6.55E-14	TRUE	UP
ZBTB20	318.9832	8.08104	0.584134	13.83422	1.58E-43	1.57E-40	TRUE	UP
LOR	12.14141	8.079422	2.044476	3.951831	7.76E-05	0.000622	TRUE	UP
CRX	2.70855	8.052372	1.969628	4.088271	4.35E-05	0.000382	TRUE	UP
HIST1H4B	6.657262	7.897893	1.397809	5.650196	1.60E-08	3.99E-07	TRUE	UP
TSHB	4.708188	7.777193	1.963492	3.960898	7.47E-05	0.000603	TRUE	UP
PSMB11	4.674465	7.759071	1.261505	6.150647	7.72E-10	2.66E-08	TRUE	UP
AWAT2	9.326814	7.753228	1.56213	4.963241	6.93E-07	1.07E-05	TRUE	UP

PLEKHN1	137.9672	7.744394	1.133426	6.832732	8.33E-12	4.19E-10	TRUE	UP
HIST1H4F	1.967959	7.742567	2.330844	3.321787	0.000894	0.004878	TRUE	UP
RPL3L	8.507571	7.721866	2.264413	3.410096	0.000649	0.003722	TRUE	UP
PGLYRP1	7.700599	7.670698	1.175505	6.525447	6.78E-11	2.89E-09	TRUE	UP
DUPD1	3.280805	7.617073	2.328595	3.271103	0.001071	0.005649	TRUE	UP
MYH8	23.10434	7.598033	1.657454	4.584159	4.56E-06	5.55E-05	TRUE	UP
CPSF4L	2.835988	7.53923	1.199897	6.283229	3.32E-10	1.23E-08	TRUE	UP
GNAT1	7.282756	7.526855	2.018707	3.728553	0.000193	0.001341	TRUE	UP
COX6A2	27.11928	7.503641	2.181391	3.439843	0.000582	0.003399	TRUE	UP
GCSH	54.63147	7.428307	0.563885	13.17344	1.25E-39	8.63E-37	TRUE	UP
PROX2	11.40252	7.302759	1.012815	7.210358	5.58E-13	3.42E-11	TRUE	UP
LGALS7	20.5204	7.299712	1.482935	4.922477	8.55E-07	1.29E-05	TRUE	UP
CCR9	10.90618	7.184573	1.383277	5.19388	2.06E-07	3.71E-06	TRUE	UP
IL4	2.714914	7.173687	1.500446	4.781037	1.74E-06	2.42E-05	TRUE	UP
PCDHGC4	210.1306	7.099765	1.267279	5.602369	2.11E-08	5.04E-07	TRUE	UP
PRSS42	1.955254	7.09307	1.676546	4.230763	2.33E-05	0.000228	TRUE	UP
CCL7	114.8497	7.044135	1.555357	4.528952	5.93E-06	6.98E-05	TRUE	UP
CRYBB3	11.0706	7.017425	1.15915	6.053942	1.41E-09	4.50E-08	TRUE	UP
CRLF2	69.23461	7.00741	1.722379	4.068448	4.73E-05	0.000411	TRUE	UP
CHRNA2	4.394983	6.974811	1.730401	4.030749	5.56E-05	0.000471	TRUE	UP
ACPP	290.4607	6.963909	1.128025	6.173538	6.68E-10	2.32E-08	TRUE	UP
HIST1H3I	3.740717	6.942823	1.545572	4.492072	7.05E-06	8.09E-05	TRUE	UP
NEUROD2	4.273477	6.926902	1.745143	3.969246	7.21E-05	0.000588	TRUE	UP
RPL36A	614.4771	6.921534	0.454304	15.23545	2.06E-52	5.69E-49	TRUE	UP
ARR3	1.998964	6.920481	1.717401	4.029625	5.59E-05	0.000473	TRUE	UP
PLSCR2	4.780045	6.919165	1.644724	4.206885	2.59E-05	0.000249	TRUE	UP
BTNL2	2.386466	6.917204	2.209399	3.130808	0.001743	0.008384	TRUE	UP
UPP2	16.90377	6.877144	1.494939	4.600283	4.22E-06	5.21E-05	TRUE	UP
SPRED3	55.82543	6.875387	1.47448	4.662925	3.12E-06	4.01E-05	TRUE	UP
ARL5C	11.30654	6.849256	0.784547	8.730203	2.54E-18	3.26E-16	TRUE	UP
SPEF1	57.43797	6.842617	0.901716	7.588436	3.24E-14	2.40E-12	TRUE	UP
GBP6	64.34225	6.842028	1.230966	5.558259	2.72E-08	6.26E-07	TRUE	UP
RGS17	238.7407	6.837334	0.985322	6.93919	3.94E-12	2.13E-10	TRUE	UP
ATP2A1	346.1406	6.823829	1.134869	6.012879	1.82E-09	5.67E-08	TRUE	UP
TNNC2	377.6791	6.801414	1.000544	6.797719	1.06E-11	5.20E-10	TRUE	UP
AMPD1	29.75473	6.799108	2.057906	3.303896	0.000954	0.005139	TRUE	UP
CACNA1S	24.64153	6.776655	1.771703	3.824937	0.000131	0.000964	TRUE	UP
TMOD4	26.66759	6.767143	0.730251	9.266878	1.92E-20	3.27E-18	TRUE	UP
SMN1	33.29644	6.764191	1.029551	6.570037	5.03E-11	2.20E-09	TRUE	UP
HIST1H2BB	1.262382	6.745268	1.884003	3.580286	0.000343	0.002164	TRUE	UP
RGS8	3.256548	6.727857	1.586831	4.239808	2.24E-05	0.00022	TRUE	UP
MYH1	154.0787	6.706532	2.176128	3.081864	0.002057	0.009668	TRUE	UP
HAL	19.25208	6.679748	1.042995	6.404391	1.51E-10	5.95E-09	TRUE	UP
HIST1H1D	29.44156	6.667398	1.145401	5.821015	5.85E-09	1.61E-07	TRUE	UP
NUP210L	17.48073	6.663037	1.368955	4.867244	1.13E-06	1.67E-05	TRUE	UP
CALR3	3.210016	6.654639	1.093969	6.083023	1.18E-09	3.82E-08	TRUE	UP
TIFAB	78.70168	6.622536	1.266484	5.22907	1.70E-07	3.13E-06	TRUE	UP
FGF21	8.090475	6.616245	2.080174	3.180621	0.00147	0.007257	TRUE	UP

KCNA7	6.096616	6.60774	2.112375	3.12811	0.001759	0.008452	TRUE	UP
CNGB1	10.79799	6.605475	1.924482	3.43234	0.000598	0.003476	TRUE	UP
DCST1	5.328879	6.599253	1.305034	5.056769	4.26E-07	7.04E-06	TRUE	UP
APOC4	10.06409	6.552211	1.633219	4.011839	6.02E-05	0.000506	TRUE	UP
RPS27	1060.369	6.515796	0.488294	13.34401	1.28E-40	1.04E-37	TRUE	UP
DPPA3	1.008525	6.500474	2.102815	3.091321	0.001993	0.00941	TRUE	UP
GCNT7	2.868332	6.493365	0.967301	6.712872	1.91E-11	8.89E-10	TRUE	UP
NEURL3	74.67463	6.476063	0.93963	6.892141	5.50E-12	2.84E-10	TRUE	UP
PVALB	364.6049	6.474295	1.763156	3.671993	0.000241	0.001622	TRUE	UP
P2RY4	5.565533	6.465743	0.914434	7.07076	1.54E-12	9.07E-11	TRUE	UP
DKK2	1426.998	6.436955	1.267874	5.076966	3.84E-07	6.41E-06	TRUE	UP
KCNH7	8.921276	6.410726	1.483888	4.320223	1.56E-05	0.00016	TRUE	UP
IL1RL1	421.5789	6.397923	1.21868	5.249881	1.52E-07	2.86E-06	TRUE	UP
SLC39A2	34.0829	6.393994	1.769747	3.612941	0.000303	0.001957	TRUE	UP
GJB3	29.39013	6.370617	1.043172	6.106964	1.02E-09	3.38E-08	TRUE	UP
PRKAG3	17.73769	6.367983	1.905364	3.342135	0.000831	0.004583	TRUE	UP
GAPDHS	15.2317	6.360575	0.949049	6.70205	2.06E-11	9.45E-10	TRUE	UP
XIRP2	36.12663	6.345375	1.683007	3.770261	0.000163	0.001165	TRUE	UP
DCDC2B	2.85277	6.334602	1.039296	6.095091	1.09E-09	3.58E-08	TRUE	UP
PIRT	13.41995	6.318218	1.936354	3.262945	0.001103	0.005774	TRUE	UP
HIST1H2AD	5.964988	6.317006	1.629909	3.87568	0.000106	0.000814	TRUE	UP
TCAP	83.23559	6.29999	0.630964	9.984701	1.78E-23	4.24E-21	TRUE	UP
TNNI2	258.426	6.294968	1.21765	5.16977	2.34E-07	4.13E-06	TRUE	UP
CCDC27	2.130659	6.287437	1.678002	3.746977	0.000179	0.001259	TRUE	UP
ACTL6B	2.155243	6.276156	2.001863	3.135157	0.001718	0.008286	TRUE	UP
SH2D5	57.74397	6.255557	1.267927	4.933691	8.07E-07	1.22E-05	TRUE	UP
TEKT2	29.62866	6.207827	1.10827	5.60137	2.13E-08	5.06E-07	TRUE	UP
MYBPC2	99.62408	6.204275	1.925851	3.221576	0.001275	0.006495	TRUE	UP
DQX1	15.91908	6.186132	1.43598	4.307951	1.65E-05	0.000168	TRUE	UP
DMP1	10.79616	6.166071	1.492033	4.132665	3.59E-05	0.000324	TRUE	UP
OPTC	5.201105	6.150825	1.638687	3.753508	0.000174	0.00123	TRUE	UP
HFE2	23.40227	6.149377	1.578819	3.894923	9.82E-05	0.000758	TRUE	UP
FGF22	5.738537	6.126704	1.704155	3.595157	0.000324	0.002064	TRUE	UP
KLHL32	32.58648	6.089812	1.251642	4.865459	1.14E-06	1.68E-05	TRUE	UP
CRYAA	4.370913	6.074437	1.351255	4.495405	6.94E-06	8.00E-05	TRUE	UP
FBXO47	1.649662	6.059407	1.817137	3.33459	0.000854	0.004685	TRUE	UP
PGLYRP2	3.451188	6.050507	1.86459	3.244954	0.001175	0.006078	TRUE	UP
SPATA1	12.37113	6.04082	0.712648	8.476583	2.32E-17	2.63E-15	TRUE	UP
NIPAL1	16.68831	6.011127	1.461157	4.113949	3.89E-05	0.000347	TRUE	UP
RNF17	4.542783	6.000216	1.691948	3.546335	0.000391	0.002411	TRUE	UP
DCT	19.72731	5.992556	1.26107	4.751962	2.01E-06	2.73E-05	TRUE	UP
WNT8B	3.688309	5.986809	1.918838	3.120017	0.001808	0.008661	TRUE	UP
LMOD2	16.03307	5.937126	1.377804	4.309122	1.64E-05	0.000167	TRUE	UP
CACNG8	8.731765	5.934717	1.610392	3.685262	0.000228	0.00155	TRUE	UP
ASAH2	21.25554	5.930533	0.770651	7.695483	1.41E-14	1.09E-12	TRUE	UP
HIST1H4I	11.37032	5.856692	0.812197	7.210927	5.56E-13	3.42E-11	TRUE	UP
CTXN3	2.559898	5.855107	1.899003	3.083253	0.002048	0.009626	TRUE	UP
GPR152	1.946225	5.85358	1.290447	4.536088	5.73E-06	6.81E-05	TRUE	UP

ALOXE3	8.525097	5.843929	1.224555	4.772287	1.82E-06	2.52E-05	TRUE	UP
IL1F9	10.30823	5.835081	1.276927	4.569626	4.89E-06	5.91E-05	TRUE	UP
KLHL31	139.6334	5.828573	1.531494	3.805808	0.000141	0.001029	TRUE	UP
DDI2	173.5333	5.802595	0.70741	8.202596	2.35E-16	2.47E-14	TRUE	UP
CCL25	23.29179	5.793453	1.640075	3.532432	0.000412	0.002523	TRUE	UP
RPGRIP1	56.27412	5.775902	0.951043	6.073227	1.25E-09	4.03E-08	TRUE	UP
ALG11	76.52416	5.774464	0.559776	10.31567	5.99E-25	1.66E-22	TRUE	UP
GJB4	2.876096	5.769548	1.197443	4.818222	1.45E-06	2.06E-05	TRUE	UP
EREG	61.19038	5.759577	1.554157	3.705917	0.000211	0.001446	TRUE	UP
KCNA2	26.19736	5.752967	1.410528	4.07859	4.53E-05	0.000396	TRUE	UP
MYT1L	4.311269	5.688696	1.677418	3.391341	0.000696	0.003937	TRUE	UP
DDX4	2.650155	5.684201	1.298864	4.376286	1.21E-05	0.000129	TRUE	UP
PPEF2	6.885401	5.676046	1.086982	5.22184	1.77E-07	3.25E-06	TRUE	UP
COL20A1	7.448414	5.660278	1.635968	3.459894	0.00054	0.003194	TRUE	UP
TMEM212	1.605687	5.657015	1.556034	3.635534	0.000277	0.001822	TRUE	UP
GRIP2	108.2505	5.653742	1.090748	5.183365	2.18E-07	3.89E-06	TRUE	UP
LCT	4.422073	5.640214	0.975174	5.783803	7.30E-09	1.95E-07	TRUE	UP
CACNG1	15.99597	5.6182	1.689938	3.324501	0.000886	0.004838	TRUE	UP
UOX	9.827829	5.575461	1.453535	3.835795	0.000125	0.000928	TRUE	UP
CLEC4D	29.15204	5.573528	1.193783	4.668794	3.03E-06	3.91E-05	TRUE	UP
MYOT	28.78545	5.569129	1.217919	4.572658	4.82E-06	5.83E-05	TRUE	UP
HIST1H4A	2.10891	5.567759	1.158118	4.80759	1.53E-06	2.14E-05	TRUE	UP
CCR5	810.8417	5.544945	0.939323	5.903131	3.57E-09	1.03E-07	TRUE	UP
HRK	23.13644	5.532687	1.723242	3.210628	0.001324	0.006691	TRUE	UP
GLTPD2	1.357949	5.470283	1.703568	3.211075	0.001322	0.00669	TRUE	UP
CNR2	8.899566	5.450586	1.285071	4.241466	2.22E-05	0.000219	TRUE	UP
CREG2	15.72893	5.439493	1.374294	3.958028	7.56E-05	0.000609	TRUE	UP
SERPINC1	54.24926	5.438356	1.384271	3.928679	8.54E-05	0.000676	TRUE	UP
ZDHHC20	474.844	5.424267	0.727112	7.460013	8.65E-14	6.02E-12	TRUE	UP
CCDC114	66.09247	5.423018	0.756285	7.170604	7.47E-13	4.51E-11	TRUE	UP
TREML4	2.291948	5.418962	1.632097	3.320244	0.000899	0.004901	TRUE	UP
CASP12	90.25373	5.409064	0.984198	5.495912	3.89E-08	8.55E-07	TRUE	UP
ANKRD23	145.4174	5.408344	0.477563	11.32488	9.88E-30	3.91E-27	TRUE	UP
SVOPL	8.106452	5.368194	1.413136	3.79878	0.000145	0.001055	TRUE	UP
HIST1H1B	8.946984	5.367354	1.039518	5.163313	2.43E-07	4.26E-06	TRUE	UP
ACOT6	9.290735	5.351727	0.97249	5.503119	3.73E-08	8.26E-07	TRUE	UP
FNDC7	6.48442	5.34087	1.300499	4.106785	4.01E-05	0.000356	TRUE	UP
12-Sep	2.310307	5.32417	1.666821	3.194206	0.001402	0.007024	TRUE	UP
TRIML2	8.986657	5.302273	1.668249	3.178347	0.001481	0.007306	TRUE	UP
UPB1	27.7761	5.282351	0.817294	6.46322	1.02E-10	4.25E-09	TRUE	UP
GAN	124.4756	5.279998	0.423206	12.4762	1.01E-35	5.57E-33	TRUE	UP
ZBTB37	97.29616	5.279856	0.600255	8.79602	1.42E-18	1.87E-16	TRUE	UP
PHACTR1	171.7629	5.256768	0.728678	7.214116	5.43E-13	3.37E-11	TRUE	UP
PATE2	5.485092	5.247779	1.35467	3.873842	0.000107	0.00082	TRUE	UP
CDKL4	2.6372	5.215863	1.359261	3.83728	0.000124	0.000923	TRUE	UP
ACADL	148.7353	5.20473	1.678532	3.100763	0.00193	0.009173	TRUE	UP
DENND1B	128.7688	5.194155	0.52271	9.936975	2.87E-23	6.63E-21	TRUE	UP
DHDH	78.81646	5.189603	1.077065	4.818284	1.45E-06	2.06E-05	TRUE	UP

IDO2	22.9791	5.18687	1.016263	5.103864	3.33E-07	5.63E-06	TRUE	UP
RASAL1	7.622321	5.178251	1.181167	4.384013	1.17E-05	0.000125	TRUE	UP
TNFRSF9	94.99066	5.177615	1.224871	4.227069	2.37E-05	0.000231	TRUE	UP
GRM5	13.87969	5.173469	1.584999	3.264021	0.001098	0.005757	TRUE	UP
LY6G6C	3.532576	5.127987	1.151067	4.454986	8.39E-06	9.35E-05	TRUE	UP
NANOG	11.81466	5.124526	1.607249	3.188384	0.001431	0.007113	TRUE	UP
CAPNS2	5.134444	5.096461	1.10084	4.629612	3.66E-06	4.60E-05	TRUE	UP
RFX3	149.9826	5.09539	0.577953	8.816265	1.18E-18	1.59E-16	TRUE	UP
SLC4A9	1.481807	5.091586	1.174025	4.336862	1.45E-05	0.00015	TRUE	UP
PLD4	309.9384	5.088813	1.001308	5.082164	3.73E-07	6.25E-06	TRUE	UP
HIST1H3C	5.537308	5.063217	1.261917	4.012323	6.01E-05	0.000505	TRUE	UP
OMP	1.239523	5.047632	1.216922	4.147869	3.36E-05	0.000307	TRUE	UP
NR6A1	22.19988	5.038293	1.060679	4.750064	2.03E-06	2.75E-05	TRUE	UP
EML5	157.5311	5.038131	0.583704	8.631315	6.07E-18	7.23E-16	TRUE	UP
ZSCAN10	3.372895	5.027854	1.419228	3.542667	0.000396	0.002439	TRUE	UP
PRDM11	117.1226	5.027261	0.670447	7.498377	6.46E-14	4.54E-12	TRUE	UP
GJC3	8.07215	5.012576	0.765637	6.546933	5.87E-11	2.53E-09	TRUE	UP
SOAT2	30.16377	5.003901	1.208684	4.139957	3.47E-05	0.000316	TRUE	UP
PRKCG	7.487163	4.993706	0.818155	6.103615	1.04E-09	3.44E-08	TRUE	UP
SLC26A8	6.121286	4.983293	0.926348	5.379506	7.47E-08	1.52E-06	TRUE	UP
GRHL2	24.28805	4.980191	1.36149	3.657896	0.000254	0.0017	TRUE	UP
GNAT2	5.018215	4.971584	0.817212	6.083589	1.18E-09	3.82E-08	TRUE	UP
FRRS1	183.7713	4.969107	0.819018	6.067155	1.30E-09	4.17E-08	TRUE	UP
CCDC62	16.15527	4.965646	0.465281	10.67235	1.37E-26	4.22E-24	TRUE	UP
C1QL3	27.93558	4.959399	1.074901	4.613819	3.95E-06	4.93E-05	TRUE	UP
MYOZ1	71.23683	4.940179	1.167691	4.230725	2.33E-05	0.000228	TRUE	UP
CD274	109.7092	4.931222	0.843491	5.846206	5.03E-09	1.41E-07	TRUE	UP
MYO1H	17.26156	4.915704	1.041634	4.719224	2.37E-06	3.14E-05	TRUE	UP
LENEP	1.105463	4.91471	1.364137	3.602797	0.000315	0.002018	TRUE	UP
XKR6	31.95451	4.901407	0.947099	5.175181	2.28E-07	4.04E-06	TRUE	UP
HIST1H2BC	159.843	4.898603	0.862429	5.680006	1.35E-08	3.40E-07	TRUE	UP
PDZD9	3.638404	4.891139	0.969288	5.046117	4.51E-07	7.37E-06	TRUE	UP
IPMK	100.4706	4.881632	0.842648	5.793202	6.91E-09	1.87E-07	TRUE	UP
HIST1H4C	3.051331	4.86276	1.006035	4.83359	1.34E-06	1.93E-05	TRUE	UP
TRPM6	68.14907	4.857345	0.97568	4.97842	6.41E-07	1.00E-05	TRUE	UP
TTC9B	1.522345	4.854126	1.138847	4.262317	2.02E-05	0.000202	TRUE	UP
CELF4	105.2873	4.846792	1.110248	4.365503	1.27E-05	0.000135	TRUE	UP
CHST8	37.76376	4.808797	1.506128	3.19282	0.001409	0.007049	TRUE	UP
MEGF11	43.05888	4.805422	1.259019	3.816799	0.000135	0.00099	TRUE	UP
TPTE	5.597652	4.799593	1.427185	3.36298	0.000771	0.00429	TRUE	UP
NEB	166.7956	4.790403	0.913782	5.242392	1.59E-07	2.97E-06	TRUE	UP
STMN4	22.56463	4.786543	1.281358	3.735524	0.000187	0.00131	TRUE	UP
POU2F2	403.4386	4.78051	0.702874	6.801371	1.04E-11	5.08E-10	TRUE	UP
VWA3A	6.5399	4.774395	1.06709	4.47422	7.67E-06	8.65E-05	TRUE	UP
GDF3	21.20274	4.766953	1.483601	3.213097	0.001313	0.006648	TRUE	UP
A4GNT	4.076023	4.73324	1.117747	4.234624	2.29E-05	0.000224	TRUE	UP
YIPF7	3.766043	4.73176	0.829578	5.703817	1.17E-08	3.02E-07	TRUE	UP
ARHGDIG	13.37804	4.716469	1.482545	3.181333	0.001466	0.007247	TRUE	UP

RPL7	2070.018	4.684563	0.41092	11.40019	4.17E-30	1.70E-27	TRUE	UP
PRRG2	57.9046	4.680614	0.833258	5.617244	1.94E-08	4.64E-07	TRUE	UP
GRIN1	8.492218	4.666114	1.514161	3.081649	0.002059	0.009671	TRUE	UP
CFP	359.0705	4.658559	0.824939	5.647155	1.63E-08	4.03E-07	TRUE	UP
HIST1H2AB	1.702807	4.658085	1.399814	3.327646	0.000876	0.004795	TRUE	UP
MPP6	278.5901	4.651166	0.693618	6.70566	2.00E-11	9.25E-10	TRUE	UP
NCR1	5.646783	4.640048	1.109415	4.182427	2.88E-05	0.000271	TRUE	UP
STYK1	33.62911	4.636678	1.040265	4.457207	8.30E-06	9.27E-05	TRUE	UP
AGBL3	28.51551	4.636472	0.598631	7.745127	9.55E-15	7.73E-13	TRUE	UP
MB	196.9749	4.614445	1.39947	3.297281	0.000976	0.00525	TRUE	UP
SLC16A6	57.83519	4.610509	0.749469	6.151702	7.67E-10	2.64E-08	TRUE	UP
BRI3BP	170.0245	4.601332	0.395559	11.63248	2.82E-31	1.30E-28	TRUE	UP
IL9R	6.036266	4.596503	1.191091	3.859071	0.000114	0.00086	TRUE	UP
HIST1H2AH	2.430593	4.596169	0.954921	4.813142	1.49E-06	2.10E-05	TRUE	UP
FAM19A1	46.8041	4.581185	1.429863	3.203934	0.001356	0.006826	TRUE	UP
GNGT2	114.4799	4.580487	0.842734	5.435273	5.47E-08	1.16E-06	TRUE	UP
PLA2G7	546.7875	4.576432	0.923045	4.957973	7.12E-07	1.10E-05	TRUE	UP
ACSL6	74.42126	4.566393	1.128001	4.048216	5.16E-05	0.000443	TRUE	UP
SLC30A4	374.9898	4.563877	0.597485	7.638479	2.20E-14	1.65E-12	TRUE	UP
DNAJB13	5.472112	4.550941	0.746054	6.100018	1.06E-09	3.49E-08	TRUE	UP
BTBD18	3.714973	4.545081	0.860037	5.284752	1.26E-07	2.41E-06	TRUE	UP
KCNK10	17.35373	4.54365	1.371111	3.313846	0.00092	0.004993	TRUE	UP
MYLK4	161.6338	4.536021	1.037689	4.371274	1.24E-05	0.000132	TRUE	UP
DGKH	112.2951	4.534821	0.607006	7.470806	7.97E-14	5.57E-12	TRUE	UP
NRG4	7.121332	4.49774	0.985467	4.56407	5.02E-06	6.04E-05	TRUE	UP
NCCRP1	18.91632	4.496981	1.079452	4.165986	3.10E-05	0.000288	TRUE	UP
MEP1A	1.581227	4.491718	1.334818	3.365042	0.000765	0.004265	TRUE	UP
CCR2	272.4695	4.477799	0.952563	4.700789	2.59E-06	3.41E-05	TRUE	UP
TPH1	4.425108	4.474955	1.1434	3.913727	9.09E-05	0.00071	TRUE	UP
ALOX12	20.17009	4.473571	0.778044	5.749765	8.94E-09	2.36E-07	TRUE	UP
AGBL1	2.247388	4.469084	1.433341	3.117948	0.001821	0.008708	TRUE	UP
GALR2	7.091745	4.436305	1.138971	3.895011	9.82E-05	0.000758	TRUE	UP
UNC45B	33.26197	4.433292	1.139256	3.891392	9.97E-05	0.000768	TRUE	UP
RNASE10	4.081245	4.413622	0.887211	4.974717	6.53E-07	1.02E-05	TRUE	UP
BTBD16	8.851618	4.405656	1.190893	3.699455	0.000216	0.001481	TRUE	UP
MBOAT4	6.922503	4.40546	0.847049	5.200949	1.98E-07	3.59E-06	TRUE	UP
EME2	56.31046	4.395497	0.770636	5.703725	1.17E-08	3.02E-07	TRUE	UP
ZBP1	105.0038	4.390305	1.068965	4.107061	4.01E-05	0.000356	TRUE	UP
SNX32	66.40588	4.384867	0.921645	4.757651	1.96E-06	2.67E-05	TRUE	UP
RPL18A	3724.154	4.376892	0.457428	9.568477	1.08E-21	2.08E-19	TRUE	UP
SLC25A2	1.99978	4.359	0.874722	4.983298	6.25E-07	9.80E-06	TRUE	UP
NEDD4	2033.239	4.357043	0.442814	9.839452	7.61E-23	1.67E-20	TRUE	UP
MED12L	35.67519	4.356947	1.036914	4.201839	2.65E-05	0.000254	TRUE	UP
MSH4	14.86055	4.328457	1.018127	4.25139	2.12E-05	0.000211	TRUE	UP
GRIK4	55.8949	4.314226	1.17559	3.669837	0.000243	0.001633	TRUE	UP
RPL39	1334.834	4.309161	0.557802	7.725256	1.12E-14	8.88E-13	TRUE	UP
CX3CR1	468.435	4.304647	1.096717	3.925029	8.67E-05	0.000684	TRUE	UP
ATG9B	71.51609	4.285507	0.958607	4.470559	7.80E-06	8.77E-05	TRUE	UP

CUX2	49.37442	4.279832	1.14205	3.747498	0.000179	0.001257	TRUE	UP
NAPSA	20.33756	4.271504	0.679832	6.283176	3.32E-10	1.23E-08	TRUE	UP
NAT2	21.38636	4.266302	1.246724	3.422011	0.000622	0.003585	TRUE	UP
RRH	5.137657	4.2647	0.790803	5.392875	6.93E-08	1.43E-06	TRUE	UP
MS4A14	208.9761	4.263156	0.850553	5.012216	5.38E-07	8.62E-06	TRUE	UP
TXK	36.73207	4.256624	0.801022	5.313992	1.07E-07	2.10E-06	TRUE	UP
HSF5	4.525562	4.246576	0.970463	4.375825	1.21E-05	0.00013	TRUE	UP
HAVCR1	1.319079	4.243362	1.101861	3.851085	0.000118	0.000882	TRUE	UP
ENO3	417.2164	4.242986	0.901964	4.704165	2.55E-06	3.36E-05	TRUE	UP
IGSF10	330.2097	4.241235	1.091161	3.8869	0.000102	0.000781	TRUE	UP
WNT9A	60.77603	4.237147	0.960904	4.409542	1.04E-05	0.000113	TRUE	UP
MBNL3	105.5722	4.226426	0.706358	5.983403	2.19E-09	6.67E-08	TRUE	UP
PFKFB1	12.58799	4.21834	0.908389	4.643758	3.42E-06	4.34E-05	TRUE	UP
SASS6	392.3208	4.207747	0.454619	9.255554	2.13E-20	3.55E-18	TRUE	UP
RPS28	1209.364	4.19511	1.070568	3.918582	8.91E-05	0.000698	TRUE	UP
IL18RAP	55.09589	4.187628	0.841468	4.976576	6.47E-07	1.01E-05	TRUE	UP
MYADML2	4.549373	4.167379	0.938866	4.438737	9.05E-06	9.98E-05	TRUE	UP
RHO	3.248089	4.166207	1.318436	3.159962	0.001578	0.007714	TRUE	UP
SOCS7	110.8475	4.161874	0.677838	6.139923	8.26E-10	2.81E-08	TRUE	UP
SYNGR4	4.627666	4.158642	1.067092	3.897172	9.73E-05	0.000752	TRUE	UP
TTN	466.4983	4.15065	0.597376	6.948141	3.70E-12	2.03E-10	TRUE	UP
ART3	22.17571	4.133884	1.312305	3.150093	0.001632	0.007946	TRUE	UP
SLC12A5	36.01163	4.124571	0.87731	4.701384	2.58E-06	3.40E-05	TRUE	UP
FAM71F1	4.415161	4.124509	1.074223	3.839528	0.000123	0.000916	TRUE	UP
SLC35E2.1	132.187	4.089959	0.535075	7.643712	2.11E-14	1.60E-12	TRUE	UP
ENKUR	15.68716	4.084346	1.083148	3.770811	0.000163	0.001164	TRUE	UP
CD3G	62.00589	4.065978	1.245254	3.26518	0.001094	0.005738	TRUE	UP
SLC38A6	565.7601	4.064209	0.476132	8.535885	1.39E-17	1.60E-15	TRUE	UP
LMTK3	81.3913	4.041798	1.069688	3.778484	0.000158	0.001135	TRUE	UP
CRHR2	4.307469	4.03938	1.147244	3.520944	0.00043	0.002613	TRUE	UP
ITGA2B	73.74562	4.036447	0.892007	4.525132	6.04E-06	7.10E-05	TRUE	UP
ELK4	233.2882	4.033013	0.408462	9.87365	5.42E-23	1.21E-20	TRUE	UP
CLEC12A	120.8795	4.030208	1.042104	3.867375	0.00011	0.000837	TRUE	UP
DOC2B	45.6972	4.027449	0.98023	4.108676	3.98E-05	0.000354	TRUE	UP
PTX4	5.372743	4.026135	1.114294	3.613171	0.000302	0.001957	TRUE	UP
LPAR4	97.46434	4.024976	1.079956	3.726981	0.000194	0.001347	TRUE	UP
PROZ	5.298818	4.004441	1.263595	3.169086	0.001529	0.007512	TRUE	UP
WDR66	72.89212	3.984119	0.838401	4.752043	2.01E-06	2.73E-05	TRUE	UP
GPT	32.03706	3.976622	0.894211	4.447075	8.70E-06	9.65E-05	TRUE	UP
RCAN3	71.39482	3.964169	0.889532	4.456466	8.33E-06	9.30E-05	TRUE	UP
RAB27B	38.11734	3.961265	1.284542	3.083795	0.002044	0.009612	TRUE	UP
RNF183	16.57097	3.959987	1.251292	3.164717	0.001552	0.007603	TRUE	UP
BTBD8	16.03426	3.953309	0.795302	4.970829	6.67E-07	1.03E-05	TRUE	UP
ACRBP	145.7852	3.91669	0.703885	5.564389	2.63E-08	6.08E-07	TRUE	UP
TTYH1	39.78371	3.902202	0.932221	4.185918	2.84E-05	0.000268	TRUE	UP
IL1RL2	31.58112	3.900432	1.081217	3.607447	0.000309	0.001989	TRUE	UP
SLC23A1	9.519422	3.892334	0.853522	4.560319	5.11E-06	6.13E-05	TRUE	UP
CXCR2	33.07631	3.890689	1.115746	3.487074	0.000488	0.002919	TRUE	UP

CACNA2D1	311.0665	3.880186	0.980014	3.959315	7.52E-05	0.000606	TRUE	UP
SFT2D2	189.087	3.877413	0.447793	8.658949	4.76E-18	5.78E-16	TRUE	UP
ANXA3	364.408	3.865223	1.089369	3.548129	0.000388	0.002399	TRUE	UP
CCNT1	220.9543	3.859336	0.715152	5.396524	6.79E-08	1.40E-06	TRUE	UP
CDHR4	15.71439	3.856207	1.173677	3.285578	0.001018	0.005412	TRUE	UP
ANAPC4	1217.634	3.84177	0.336153	11.42865	3.01E-30	1.27E-27	TRUE	UP
UCK2	874.487	3.833037	0.414942	9.237519	2.52E-20	4.11E-18	TRUE	UP
PRDM12	4.19913	3.830751	1.129662	3.391058	0.000696	0.003939	TRUE	UP
FAM53A	127.4784	3.816878	0.549046	6.951834	3.61E-12	2.00E-10	TRUE	UP
PKD1L3	6.157904	3.808003	0.840412	4.531114	5.87E-06	6.92E-05	TRUE	UP
GPR182	12.49047	3.807495	1.188689	3.203105	0.00136	0.006843	TRUE	UP
CACNA1F	9.538314	3.791886	0.869152	4.362744	1.28E-05	0.000136	TRUE	UP
STOX2	156.7666	3.778668	0.93276	4.051063	5.10E-05	0.000438	TRUE	UP
EFCAB3	2.48991	3.77424	1.090677	3.460457	0.000539	0.003189	TRUE	UP
ZKSCAN3	190.4034	3.769635	0.407275	9.255757	2.13E-20	3.55E-18	TRUE	UP
INSL3	25.56099	3.757817	1.195485	3.14334	0.00167	0.008095	TRUE	UP
RASL10A	32.11428	3.749421	1.076936	3.481562	0.000498	0.002969	TRUE	UP
HIST4H4	24.184	3.734567	0.791853	4.716241	2.40E-06	3.18E-05	TRUE	UP
RPL9	2393.133	3.724739	0.772304	4.82289	1.41E-06	2.02E-05	TRUE	UP
PSORS1C2	10.461	3.719279	1.15318	3.225237	0.001259	0.00643	TRUE	UP
RPS26	2077.408	3.716437	0.641247	5.795644	6.81E-09	1.85E-07	TRUE	UP
SLC5A11	6.555261	3.694762	0.968687	3.814195	0.000137	0.000999	TRUE	UP
HES7	14.68056	3.693753	1.11186	3.32214	0.000893	0.004874	TRUE	UP
PHOSPHO2	102.5004	3.693198	0.584896	6.314279	2.71E-10	1.03E-08	TRUE	UP
RABGAP1L	887.7104	3.684566	0.48977	7.523057	5.35E-14	3.80E-12	TRUE	UP
KCNRG	15.11035	3.683279	0.582575	6.322412	2.58E-10	9.81E-09	TRUE	UP
SYCP3	1.247276	3.672969	1.123647	3.268792	0.00108	0.005686	TRUE	UP
NMBR	2.763429	3.66657	1.137142	3.224373	0.001262	0.006446	TRUE	UP
IMMP1L	132.0816	3.65072	0.375805	9.714412	2.62E-22	5.57E-20	TRUE	UP
KCNH4	15.86818	3.645821	0.914499	3.986687	6.70E-05	0.000553	TRUE	UP
KCNN4	420.0513	3.641905	0.993018	3.667512	0.000245	0.001646	TRUE	UP
KLC3	31.1982	3.641033	1.035989	3.514549	0.000441	0.00267	TRUE	UP
RBMS3	327.9164	3.634941	0.627854	5.78947	7.06E-09	1.90E-07	TRUE	UP
STAP1	38.98757	3.623912	1.172583	3.090537	0.001998	0.009428	TRUE	UP
PYGO1	70.52215	3.622397	0.844494	4.289431	1.79E-05	0.000181	TRUE	UP
ERN1	200.5712	3.621035	0.681822	5.31082	1.09E-07	2.12E-06	TRUE	UP
CBX6	2951.028	3.617898	0.616717	5.86638	4.45E-09	1.26E-07	TRUE	UP
GPR157	37.73798	3.617818	0.689594	5.246305	1.55E-07	2.91E-06	TRUE	UP
GABPB2	276.1278	3.616326	0.401681	9.002986	2.20E-19	3.17E-17	TRUE	UP
GPRC5D	3.395181	3.603681	1.078683	3.340814	0.000835	0.004601	TRUE	UP
NUDT14	812.8412	3.600017	0.634699	5.672007	1.41E-08	3.54E-07	TRUE	UP
FBXO15	24.18125	3.592961	0.957127	3.753902	0.000174	0.001229	TRUE	UP
P2RY2	33.89294	3.590482	0.855573	4.196583	2.71E-05	0.000259	TRUE	UP
NFATC2	115.2134	3.58488	0.913763	3.923205	8.74E-05	0.000686	TRUE	UP
MYO1A	12.62926	3.583708	0.998111	3.590492	0.00033	0.002096	TRUE	UP
SLC9A5	148.4047	3.5765	0.705887	5.066675	4.05E-07	6.72E-06	TRUE	UP
HAUS8	647.6853	3.571994	0.495097	7.214737	5.40E-13	3.37E-11	TRUE	UP
RGS20	37.1231	3.565967	1.128581	3.159691	0.001579	0.007719	TRUE	UP

GXYLT2	919.5901	3.543248	0.747915	4.737497	2.16E-06	2.89E-05	TRUE	UP
PPP1R14B	2366.838	3.535901	0.538645	6.564437	5.22E-11	2.27E-09	TRUE	UP
NBEAL1	240.2488	3.53083	0.708541	4.98324	6.25E-07	9.80E-06	TRUE	UP
FCGR2B	1090.831	3.527405	1.021217	3.454121	0.000552	0.003255	TRUE	UP
NPFF	73.47937	3.519945	0.893495	3.939524	8.16E-05	0.000649	TRUE	UP
CSF2RA	569.7138	3.51982	0.757501	4.646622	3.37E-06	4.30E-05	TRUE	UP
IMPG2	14.06122	3.491991	0.828883	4.21289	2.52E-05	0.000244	TRUE	UP
CARD9	248.2037	3.47384	0.805117	4.3147	1.60E-05	0.000164	TRUE	UP
RDM1	47.78248	3.471845	0.979653	3.543953	0.000394	0.002428	TRUE	UP
USP49	57.31457	3.47005	0.814519	4.260245	2.04E-05	0.000203	TRUE	UP
PIK3CA	1114.667	3.469975	0.40208	8.630055	6.13E-18	7.25E-16	TRUE	UP
PPM1L	241.6418	3.469339	0.838086	4.139596	3.48E-05	0.000316	TRUE	UP
ABCA5	528.1473	3.454543	0.852456	4.052458	5.07E-05	0.000436	TRUE	UP
CCDC65	21.11339	3.452905	0.80889	4.268695	1.97E-05	0.000197	TRUE	UP
ZC3H12D	36.93411	3.450723	0.833459	4.140242	3.47E-05	0.000316	TRUE	UP
PRIM1	548.5871	3.449168	0.537923	6.412009	1.44E-10	5.76E-09	TRUE	UP
ANKRD44	236.0574	3.440486	0.664642	5.176449	2.26E-07	4.02E-06	TRUE	UP
CCDC157	92.2895	3.428307	0.434426	7.891582	2.98E-15	2.58E-13	TRUE	UP
P2RY12	69.4254	3.420144	0.927807	3.686266	0.000228	0.001545	TRUE	UP
REL	88.42977	3.389789	0.587214	5.772663	7.80E-09	2.08E-07	TRUE	UP
FMN1	124.9542	3.385694	1.049707	3.22537	0.001258	0.00643	TRUE	UP
ABHD12B	6.304572	3.38231	1.035075	3.267696	0.001084	0.005702	TRUE	UP
IL27	13.24995	3.381821	0.965496	3.502678	0.000461	0.002778	TRUE	UP
GVIN1	208.1821	3.371368	0.753298	4.47548	7.62E-06	8.62E-05	TRUE	UP
CDC26	175.4206	3.355572	0.467253	7.181494	6.90E-13	4.18E-11	TRUE	UP
CD300LB	59.22793	3.354655	0.83544	4.015436	5.93E-05	0.000499	TRUE	UP
GRAMD1B	154.9319	3.344719	0.839001	3.986548	6.70E-05	0.000553	TRUE	UP
MSI2	310.6365	3.34282	0.628648	5.317475	1.05E-07	2.06E-06	TRUE	UP
SESN3	353.3057	3.335895	0.692735	4.815546	1.47E-06	2.08E-05	TRUE	UP
GTF2A1	310.5003	3.332893	0.696147	4.787626	1.69E-06	2.35E-05	TRUE	UP
HOMER1	205.5413	3.316331	0.798108	4.155243	3.25E-05	0.0003	TRUE	UP
TLE6	127.8614	3.316039	0.970461	3.416974	0.000633	0.003643	TRUE	UP
IRAK1BP1	97.93683	3.312234	0.379764	8.721829	2.74E-18	3.47E-16	TRUE	UP
CD38	155.5607	3.308819	1.036932	3.190968	0.001418	0.007076	TRUE	UP
PDGFA	1146.678	3.303487	0.757517	4.360941	1.30E-05	0.000137	TRUE	UP
FITM1	32.09956	3.301885	0.83029	3.976787	6.99E-05	0.000572	TRUE	UP
MPZL3	9.672721	3.301795	0.883756	3.736093	0.000187	0.001308	TRUE	UP
CD84	222.0077	3.294924	0.795407	4.142437	3.44E-05	0.000313	TRUE	UP
PTPN14	315.5903	3.283219	0.602461	5.449679	5.05E-08	1.09E-06	TRUE	UP
DLEC1	26.74477	3.271066	0.775361	4.218766	2.46E-05	0.000239	TRUE	UP
HSPA4L	538.9998	3.261844	0.866006	3.766536	0.000166	0.001182	TRUE	UP
BRAF	246.5592	3.259979	0.416006	7.836372	4.64E-15	3.91E-13	TRUE	UP
GATC	81.7073	3.257569	0.461123	7.064433	1.61E-12	9.46E-11	TRUE	UP
RPL17	5588.792	3.256414	0.399274	8.155841	3.47E-16	3.61E-14	TRUE	UP
TNFRSF11A	127.867	3.254702	0.841543	3.867541	0.00011	0.000837	TRUE	UP
PCDHGC5	100.1458	3.247729	1.049192	3.095458	0.001965	0.009295	TRUE	UP
PAFAH1B2	585.097	3.247081	0.501232	6.478205	9.28E-11	3.87E-09	TRUE	UP
GNG3	9.894224	3.237448	0.816163	3.966669	7.29E-05	0.000593	TRUE	UP

LRRC39	36.57656	3.227858	0.77794	4.149239	3.34E-05	0.000305	TRUE	UP
GLRX2	347.5568	3.22761	0.440755	7.322908	2.43E-13	1.58E-11	TRUE	UP
FABP5	316.4286	3.224825	0.790447	4.079749	4.51E-05	0.000394	TRUE	UP
FAM71F2	16.9533	3.222766	0.797068	4.043278	5.27E-05	0.000449	TRUE	UP
SLC9A7	66.62429	3.221408	0.932989	3.452783	0.000555	0.003267	TRUE	UP
GZMB	139.1678	3.221032	0.935363	3.443616	0.000574	0.00336	TRUE	UP
SMTNL1	12.92076	3.218936	0.86842	3.706659	0.00021	0.001442	TRUE	UP
SIRT4	86.54991	3.214393	0.54707	5.875649	4.21E-09	1.20E-07	TRUE	UP
SMYD2	1003.996	3.212575	0.362272	8.867858	7.46E-19	1.03E-16	TRUE	UP
ZSCAN5B	5.040637	3.209768	0.83447	3.846476	0.00012	0.000894	TRUE	UP
TBX15	894.804	3.207953	1.018005	3.151217	0.001626	0.007921	TRUE	UP
ARHGAP22	757.3937	3.205209	0.901884	3.553906	0.00038	0.002354	TRUE	UP
DNASE1L2	31.37411	3.193506	0.763855	4.180772	2.91E-05	0.000272	TRUE	UP
UGCG	552.3741	3.191182	0.613443	5.202087	1.97E-07	3.57E-06	TRUE	UP
CENPI	147.221	3.187513	0.696702	4.575147	4.76E-06	5.78E-05	TRUE	UP
CHD7	628.3099	3.184889	0.759563	4.193057	2.75E-05	0.000262	TRUE	UP
AGBL2	29.95256	3.184136	0.724329	4.395983	1.10E-05	0.00012	TRUE	UP
RPL23A	5957.278	3.182821	0.368505	8.637129	5.76E-18	6.94E-16	TRUE	UP
VWA5B2	36.14327	3.17982	0.922262	3.447848	0.000565	0.003318	TRUE	UP
TMEM38A	158.6063	3.174414	0.783236	4.052945	5.06E-05	0.000436	TRUE	UP
GJB5	8.734125	3.170994	0.690825	4.590154	4.43E-06	5.43E-05	TRUE	UP
HELB	74.98882	3.163262	0.839898	3.766246	0.000166	0.001183	TRUE	UP
SNX20	141.8249	3.155621	0.723167	4.363612	1.28E-05	0.000136	TRUE	UP
TNF	20.30065	3.155209	1.017158	3.101986	0.001922	0.009146	TRUE	UP
NCF1	488.2914	3.153087	0.974512	3.235553	0.001214	0.006238	TRUE	UP
LOXL3	1519.972	3.148724	0.734023	4.289681	1.79E-05	0.000181	TRUE	UP
TSPAN5	289.8011	3.147377	0.795723	3.955365	7.64E-05	0.000614	TRUE	UP
LEFTY1	82.73461	3.138162	0.982897	3.192768	0.001409	0.007049	TRUE	UP
B4GALT6	395.3265	3.135879	1.005295	3.11936	0.001812	0.008671	TRUE	UP
PPCDC	523.9828	3.134866	0.396853	7.89932	2.80E-15	2.46E-13	TRUE	UP
HIST3H2A	94.04317	3.133736	1.014138	3.09005	0.002001	0.00944	TRUE	UP
ENPP3	36.40072	3.130107	0.866186	3.613667	0.000302	0.001954	TRUE	UP
GNRHR	5.438186	3.122036	0.918955	3.397377	0.00068	0.003871	TRUE	UP
INHA	23.97051	3.118763	0.78415	3.977254	6.97E-05	0.000571	TRUE	UP
FLVCR1	142.6101	3.113534	0.517718	6.013956	1.81E-09	5.64E-08	TRUE	UP
CHD9	2519.391	3.112432	0.506492	6.145072	7.99E-10	2.74E-08	TRUE	UP
NOXO1	11.56406	3.111995	0.607925	5.119044	3.07E-07	5.25E-06	TRUE	UP
FAM129C	18.6757	3.109233	0.781776	3.977141	6.97E-05	0.000571	TRUE	UP
PPP4R2	698.6616	3.108238	0.485116	6.407205	1.48E-10	5.88E-09	TRUE	UP
GPR19	72.00966	3.102807	0.806401	3.847721	0.000119	0.000892	TRUE	UP
GFOD1	184.031	3.102032	0.667781	4.645284	3.40E-06	4.31E-05	TRUE	UP
CCDC148	17.8894	3.094336	0.878683	3.521561	0.000429	0.002608	TRUE	UP
RPSA	11850.04	3.091508	0.404545	7.64193	2.14E-14	1.62E-12	TRUE	UP
UHMK1	345.051	3.089555	0.77094	4.007515	6.14E-05	0.000513	TRUE	UP
TOR1AIP2	632.3469	3.08681	0.444884	6.938469	3.96E-12	2.13E-10	TRUE	UP
HAGHL	117.7568	3.086652	0.723669	4.265282	2.00E-05	0.000199	TRUE	UP
CCDC134	126.0245	3.078134	0.389757	7.897575	2.84E-15	2.47E-13	TRUE	UP
TEX9	86.92261	3.070741	0.682499	4.499262	6.82E-06	7.87E-05	TRUE	UP

CDNF	46.79458	3.069625	0.586097	5.237402	1.63E-07	3.02E-06	TRUE	UP
ZMYND15	242.3058	3.062881	0.694228	4.411926	1.02E-05	0.000112	TRUE	UP
SAMD8	189.1244	3.061537	0.479831	6.380446	1.77E-10	6.94E-09	TRUE	UP
HMBOX1	187.3432	3.061455	0.518662	5.902605	3.58E-09	1.03E-07	TRUE	UP
ALS2CR12	10.90892	3.040142	0.723888	4.19974	2.67E-05	0.000256	TRUE	UP
TTC39B	276.9329	3.029383	0.591925	5.117846	3.09E-07	5.27E-06	TRUE	UP
CASC1	17.46095	3.025485	0.919181	3.2915	0.000997	0.005329	TRUE	UP
EIF3C	1066.912	3.022925	0.647571	4.668102	3.04E-06	3.92E-05	TRUE	UP
HJURP	800.7815	3.015051	0.691559	4.359786	1.30E-05	0.000138	TRUE	UP
RAPGEF4	436.2498	3.007546	0.807498	3.724523	0.000196	0.001359	TRUE	UP
NEK10	39.91055	3.006694	0.865761	3.472892	0.000515	0.003056	TRUE	UP
CCDC36	132.6453	3.006098	0.954677	3.14881	0.001639	0.00797	TRUE	UP
LY86	668.8309	3.003848	0.877508	3.423156	0.000619	0.003573	TRUE	UP
RNF217	211.5172	3.002489	0.532962	5.633587	1.76E-08	4.31E-07	TRUE	UP
PSTK	102.9223	2.999044	0.503712	5.953888	2.62E-09	7.89E-08	TRUE	UP
RHBDL2	47.86888	2.998055	0.770809	3.889494	0.0001	0.000774	TRUE	UP
UBE2S	1155.835	2.995189	0.5642	5.308734	1.10E-07	2.14E-06	TRUE	UP
ITPRIPL1	169.7383	2.993282	0.646417	4.630575	3.65E-06	4.59E-05	TRUE	UP
CENPA	350.9973	2.988146	0.668725	4.468426	7.88E-06	8.85E-05	TRUE	UP
XPO4	1137.238	2.987243	0.375802	7.948984	1.88E-15	1.76E-13	TRUE	UP
TAOK1	497.2972	2.979878	0.748217	3.982638	6.82E-05	0.000561	TRUE	UP
PTPN22	166.3312	2.977957	0.847972	3.511857	0.000445	0.002694	TRUE	UP
ADAM32	48.6617	2.966152	0.893959	3.317996	0.000907	0.004931	TRUE	UP
KCNK13	49.43878	2.962858	0.860166	3.444518	0.000572	0.003354	TRUE	UP
LEKR1	27.89109	2.961542	0.647728	4.572202	4.83E-06	5.84E-05	TRUE	UP
NUDT13	86.86345	2.961504	0.523406	5.658136	1.53E-08	3.81E-07	TRUE	UP
GPR35	106.061	2.961144	0.640687	4.621826	3.80E-06	4.76E-05	TRUE	UP
NDRG4	276.0064	2.944972	0.931258	3.16236	0.001565	0.007657	TRUE	UP
BICD1	357.0425	2.934856	0.528898	5.549002	2.87E-08	6.56E-07	TRUE	UP
FBF1	590.0937	2.928462	0.513851	5.699051	1.20E-08	3.09E-07	TRUE	UP
DOK3	693.1709	2.922853	0.669377	4.366528	1.26E-05	0.000134	TRUE	UP
PIGN	982.6846	2.919612	0.422641	6.908014	4.91E-12	2.56E-10	TRUE	UP
PIAS2	551.5898	2.91646	0.317751	9.178451	4.37E-20	6.88E-18	TRUE	UP
SLC25A4	2627.24	2.91459	0.796446	3.659492	0.000253	0.00169	TRUE	UP
NHLRC2	172.6664	2.914512	0.553603	5.264626	1.40E-07	2.66E-06	TRUE	UP
PPAN	1887.841	2.914107	0.594296	4.903462	9.42E-07	1.41E-05	TRUE	UP
SERHL	218.6338	2.900163	0.415975	6.971965	3.13E-12	1.77E-10	TRUE	UP
CAMK1D	333.4148	2.882995	0.542581	5.313482	1.08E-07	2.10E-06	TRUE	UP
BMP2	554.3199	2.878974	0.807599	3.564856	0.000364	0.002269	TRUE	UP
GINS4	536.7199	2.865335	0.513212	5.583146	2.36E-08	5.53E-07	TRUE	UP
PHOSPHO1	11.80991	2.860853	0.875903	3.266175	0.00109	0.005722	TRUE	UP
MYSM1	221.1378	2.855059	0.677953	4.211292	2.54E-05	0.000245	TRUE	UP
TGM1	48.85746	2.854224	0.751061	3.800254	0.000145	0.00105	TRUE	UP
P2RX7	253.4382	2.851502	0.737815	3.864792	0.000111	0.000845	TRUE	UP
NIPSNAP3E	180.0873	2.851236	0.649078	4.392746	1.12E-05	0.000121	TRUE	UP
RSPH9	47.85294	2.842528	0.924643	3.074188	0.002111	0.00987	TRUE	UP
CDYL2	65.01676	2.840027	0.742609	3.82439	0.000131	0.000965	TRUE	UP
FAM133B	194.9445	2.839277	0.243812	11.64534	2.42E-31	1.16E-28	TRUE	UP

TIGD4	11.63455	2.829564	0.830819	3.405755	0.00066	0.003771	TRUE	UP
RIPK3	233.0692	2.827695	0.717571	3.94065	8.13E-05	0.000647	TRUE	UP
FAM173A	299.2928	2.82714	0.476833	5.928991	3.05E-09	9.05E-08	TRUE	UP
GDPD1	41.46871	2.8239	0.525127	5.37756	7.55E-08	1.53E-06	TRUE	UP
OSCP1	284.9171	2.822924	0.476619	5.922812	3.16E-09	9.32E-08	TRUE	UP
MAPK6	1931.853	2.822726	0.374344	7.540469	4.68E-14	3.36E-12	TRUE	UP
HFE	269.9309	2.819413	0.582051	4.843928	1.27E-06	1.84E-05	TRUE	UP
FBXL20	340.8858	2.805019	0.307556	9.120346	7.49E-20	1.14E-17	TRUE	UP
CD300LF	109.2394	2.799879	0.864715	3.237921	0.001204	0.006202	TRUE	UP
KLF7	326.913	2.798892	0.531097	5.270024	1.36E-07	2.59E-06	TRUE	UP
NOL12	707.3718	2.798054	0.353397	7.9176	2.42E-15	2.20E-13	TRUE	UP
ETV3	296.0015	2.790966	0.722324	3.863871	0.000112	0.000847	TRUE	UP
ACSBG1	61.83845	2.788566	0.758439	3.676715	0.000236	0.001598	TRUE	UP
CHKB	677.1529	2.786539	0.481703	5.784767	7.26E-09	1.95E-07	TRUE	UP
SAMD1	1027.788	2.780875	0.337691	8.234969	1.80E-16	1.90E-14	TRUE	UP
ATXN7L2	292.4555	2.773518	0.591105	4.692089	2.70E-06	3.53E-05	TRUE	UP
RHD	9.031293	2.771763	0.800576	3.46221	0.000536	0.003169	TRUE	UP
UBN2	1060.541	2.759676	0.40745	6.773047	1.26E-11	6.06E-10	TRUE	UP
08-Mar	387.0874	2.757565	0.515551	5.348772	8.86E-08	1.77E-06	TRUE	UP
MORN1	125.4059	2.75244	0.622113	4.424342	9.67E-06	0.000106	TRUE	UP
EIF4E	1105.728	2.748705	0.24746	11.10769	1.15E-28	4.30E-26	TRUE	UP
LARP4B	2129.841	2.74259	0.298866	9.176667	4.45E-20	6.91E-18	TRUE	UP
LRRIQ3	15.25995	2.740439	0.801656	3.418473	0.00063	0.003624	TRUE	UP
IL3RA	713.8723	2.728348	0.587926	4.640633	3.47E-06	4.40E-05	TRUE	UP
ADAM8	423.2406	2.719758	0.812775	3.34626	0.000819	0.004524	TRUE	UP
E2F5	128.2425	2.71906	0.737533	3.686696	0.000227	0.001543	TRUE	UP
P2RY6	391.8707	2.713223	0.851169	3.187644	0.001434	0.007126	TRUE	UP
RRP1	1700.769	2.712613	0.455807	5.951232	2.66E-09	8.00E-08	TRUE	UP
RAD52	310.8218	2.711341	0.463363	5.851438	4.87E-09	1.37E-07	TRUE	UP
E2F8	225.3356	2.704735	0.828248	3.265611	0.001092	0.005731	TRUE	UP
ZDHHC21	185.3892	2.703265	0.526327	5.136098	2.81E-07	4.84E-06	TRUE	UP
PAIP2B	219.2932	2.701097	0.829136	3.257726	0.001123	0.005858	TRUE	UP
CAPN11	44.91991	2.69941	0.850225	3.174936	0.001499	0.007382	TRUE	UP
LCOR	83.06958	2.699053	0.730548	3.694558	0.00022	0.001504	TRUE	UP
NACC2	275.6499	2.685629	0.75177	3.572407	0.000354	0.002221	TRUE	UP
ADK	510.9799	2.684268	0.471246	5.696105	1.23E-08	3.13E-07	TRUE	UP
CCHCR1	2029.828	2.677981	0.582206	4.599711	4.23E-06	5.21E-05	TRUE	UP
SAMD14	344.9487	2.676312	0.854642	3.131502	0.001739	0.008373	TRUE	UP
LIPT2	36.4225	2.673873	0.430542	6.210487	5.28E-10	1.89E-08	TRUE	UP
ZBED3	125.3131	2.663035	0.660878	4.029543	5.59E-05	0.000473	TRUE	UP
TAF13	338.88	2.660103	0.449397	5.919278	3.23E-09	9.46E-08	TRUE	UP
CALHM1	7.721932	2.658074	0.833774	3.188002	0.001433	0.00712	TRUE	UP
DAPP1	168.8093	2.657188	0.811205	3.275607	0.001054	0.005572	TRUE	UP
WWTR1	1706.532	2.656388	0.576987	4.603899	4.15E-06	5.13E-05	TRUE	UP
DNAJB14	569.797	2.638181	0.395853	6.664556	2.65E-11	1.20E-09	TRUE	UP
RNFT2	93.37899	2.632792	0.754024	3.491656	0.00048	0.002875	TRUE	UP
LMBRD2	273.4801	2.632032	0.597482	4.405209	1.06E-05	0.000115	TRUE	UP
SPC24	200.6342	2.62927	0.835178	3.148156	0.001643	0.007982	TRUE	UP

EXOSC8	789.0123	2.629066	0.327606	8.025085	1.01E-15	9.89E-14	TRUE	UP
TIGD3	23.42333	2.627997	0.702827	3.739178	0.000185	0.001294	TRUE	UP
RPS7	5988.343	2.624092	0.403299	6.506559	7.69E-11	3.24E-09	TRUE	UP
SBNO1	839.3582	2.621689	0.733175	3.575802	0.000349	0.002195	TRUE	UP
BAIAP2	2139.315	2.621023	0.659888	3.971921	7.13E-05	0.000583	TRUE	UP
LETM2	152.5782	2.618472	0.538292	4.864405	1.15E-06	1.69E-05	TRUE	UP
CENPC1	627.5405	2.606694	0.31094	8.38326	5.15E-17	5.61E-15	TRUE	UP
COMTD1	218.51	2.603067	0.516225	5.042502	4.59E-07	7.50E-06	TRUE	UP
TMPRSS5	14.67819	2.600695	0.810855	3.207348	0.00134	0.006757	TRUE	UP
SYCE2	80.90774	2.600355	0.721955	3.601824	0.000316	0.002021	TRUE	UP
TIPIN	263.3605	2.595815	0.416732	6.228974	4.69E-10	1.69E-08	TRUE	UP
ULK4	178.4818	2.587478	0.515369	5.020636	5.15E-07	8.30E-06	TRUE	UP
RPL22L1	1106.337	2.57874	0.512851	5.028248	4.95E-07	8.01E-06	TRUE	UP
LRRC45	1043.338	2.57737	0.476009	5.414544	6.14E-08	1.28E-06	TRUE	UP
EYA3	213.4866	2.571915	0.521127	4.935291	8.00E-07	1.21E-05	TRUE	UP
CDK8	306.1651	2.567414	0.500554	5.129146	2.91E-07	5.00E-06	TRUE	UP
RASA2	168.3787	2.55921	0.606954	4.216478	2.48E-05	0.000241	TRUE	UP
CCDC138	94.34776	2.537975	0.49615	5.115341	3.13E-07	5.34E-06	TRUE	UP
TMEM160	241.233	2.532918	0.534054	4.742813	2.11E-06	2.82E-05	TRUE	UP
DUSP11	1050.073	2.531491	0.256377	9.874107	5.39E-23	1.21E-20	TRUE	UP
FBXW8	1067.449	2.527968	0.372041	6.794865	1.08E-11	5.28E-10	TRUE	UP
SKIL	670.2786	2.512127	0.6113	4.109485	3.97E-05	0.000353	TRUE	UP
ACE	1731.363	2.510691	0.783974	3.20252	0.001362	0.006854	TRUE	UP
GHR	959.472	2.509008	0.628154	3.994256	6.49E-05	0.000538	TRUE	UP
SLC7A6OS	392.965	2.508382	0.277107	9.052033	1.40E-19	2.07E-17	TRUE	UP
SPATA24	77.13009	2.505213	0.430499	5.819326	5.91E-09	1.62E-07	TRUE	UP
ACER2	97.99212	2.504697	0.674926	3.711072	0.000206	0.001421	TRUE	UP
UNC93B1	1876.841	2.504615	0.542186	4.619477	3.85E-06	4.81E-05	TRUE	UP
CARS	2519.754	2.500887	0.358966	6.966912	3.24E-12	1.81E-10	TRUE	UP
REST	421.177	2.487426	0.67871	3.664931	0.000247	0.001659	TRUE	UP
SMPD3	184.9037	2.487202	0.65144	3.818008	0.000135	0.000986	TRUE	UP
ARNTL2	182.5106	2.483105	0.756879	3.280716	0.001035	0.005483	TRUE	UP
FSD1L	111.3225	2.478797	0.496341	4.99414	5.91E-07	9.36E-06	TRUE	UP
SNRNP48	648.0283	2.477773	0.34186	7.247917	4.23E-13	2.69E-11	TRUE	UP
AHSA2	860.2604	2.477365	0.556005	4.455649	8.36E-06	9.33E-05	TRUE	UP
NETO2	871.7245	2.476887	0.675649	3.665936	0.000246	0.001654	TRUE	UP
PPIA	8630.29	2.476341	0.319446	7.751978	9.05E-15	7.36E-13	TRUE	UP
MTBP	229.065	2.474002	0.48435	5.107881	3.26E-07	5.52E-06	TRUE	UP
RAD9B	138.0335	2.468693	0.72	3.428742	0.000606	0.003513	TRUE	UP
IL34	625.2755	2.468685	0.784573	3.146533	0.001652	0.008024	TRUE	UP
DENND2C	99.29276	2.468579	0.632163	3.904972	9.42E-05	0.000733	TRUE	UP
AOC2	46.33461	2.466359	0.509058	4.844947	1.27E-06	1.84E-05	TRUE	UP
PARP8	521.6908	2.465318	0.688486	3.580781	0.000343	0.002161	TRUE	UP
USE1	1043.226	2.464559	0.354854	6.945267	3.78E-12	2.07E-10	TRUE	UP
ANKLE1	63.54306	2.451335	0.597627	4.101779	4.10E-05	0.000363	TRUE	UP
IER5L	1325.686	2.443392	0.76298	3.202431	0.001363	0.006854	TRUE	UP
CTH	128.8653	2.442695	0.7372	3.313478	0.000921	0.004996	TRUE	UP
FITM2	122.1076	2.442585	0.485724	5.028746	4.94E-07	8.00E-06	TRUE	UP

ALG10B	636.6422	2.442071	0.529367	4.613187	3.97E-06	4.94E-05	TRUE	UP
ADCK5	417.4877	2.437787	0.393057	6.202128	5.57E-10	1.98E-08	TRUE	UP
POU2F1	145.8799	2.437281	0.528997	4.60736	4.08E-06	5.06E-05	TRUE	UP
CCDC153	32.94035	2.428555	0.662035	3.668321	0.000244	0.001641	TRUE	UP
ASB14	19.48861	2.426698	0.581011	4.17668	2.96E-05	0.000276	TRUE	UP
RBM15	315.3614	2.424048	0.314093	7.717606	1.19E-14	9.32E-13	TRUE	UP
CLOCK	519.374	2.42118	0.623327	3.884283	0.000103	0.000788	TRUE	UP
EXOC6B	356.6561	2.416202	0.710034	3.402937	0.000667	0.003804	TRUE	UP
RAB43	519.6835	2.396929	0.347787	6.891945	5.50E-12	2.84E-10	TRUE	UP
RGP1	246.2416	2.396329	0.571739	4.191299	2.77E-05	0.000263	TRUE	UP
PDZD7	31.09117	2.394422	0.623224	3.841994	0.000122	0.000908	TRUE	UP
CORO2A	137.3564	2.391646	0.753694	3.173234	0.001508	0.007414	TRUE	UP
TM6SF1	377.8524	2.381938	0.483565	4.925791	8.40E-07	1.27E-05	TRUE	UP
CTSS	4967.242	2.377731	0.760312	3.127311	0.001764	0.008472	TRUE	UP
AFG3L1	464.1111	2.372299	0.400533	5.92285	3.16E-09	9.32E-08	TRUE	UP
ADCY7	1535.532	2.372287	0.556637	4.261823	2.03E-05	0.000202	TRUE	UP
ZC3HAV1L	102.1712	2.370656	0.714052	3.320004	0.0009	0.004903	TRUE	UP
SPATA6	182.8657	2.367002	0.415981	5.690168	1.27E-08	3.22E-07	TRUE	UP
CNOT10	1329.392	2.365469	0.300185	7.880031	3.27E-15	2.80E-13	TRUE	UP
ANKRD37	317.3219	2.363866	0.611091	3.86827	0.00011	0.000837	TRUE	UP
RARG	2341.097	2.360903	0.549621	4.295508	1.74E-05	0.000177	TRUE	UP
RAD54L2	295.4324	2.354947	0.63856	3.687903	0.000226	0.001537	TRUE	UP
CHIC2	638.0928	2.354403	0.346771	6.789503	1.13E-11	5.45E-10	TRUE	UP
REEP3	840.772	2.347083	0.486158	4.827824	1.38E-06	1.98E-05	TRUE	UP
RUNX1T1	386.0995	2.34596	0.73757	3.180662	0.001469	0.007257	TRUE	UP
ANKRD16	319.5383	2.343369	0.373079	6.28116	3.36E-10	1.24E-08	TRUE	UP
ECSCR	331.2872	2.342166	0.696144	3.364488	0.000767	0.00427	TRUE	UP
SNHG11	513.7558	2.341317	0.396015	5.912187	3.38E-09	9.83E-08	TRUE	UP
RPS2	16957.49	2.333474	0.632557	3.688955	0.000225	0.001532	TRUE	UP
STRN	394.1672	2.332417	0.594466	3.923546	8.73E-05	0.000686	TRUE	UP
RPL13A	22963.46	2.332276	0.452749	5.151363	2.59E-07	4.49E-06	TRUE	UP
AGAP1	863.3973	2.328307	0.438193	5.313427	1.08E-07	2.10E-06	TRUE	UP
PCSK4	100.2512	2.327055	0.651639	3.571081	0.000356	0.002228	TRUE	UP
ABHD11	669.5967	2.323624	0.379225	6.127297	8.94E-10	3.02E-08	TRUE	UP
USP12	451.4046	2.31491	0.633844	3.652175	0.00026	0.00173	TRUE	UP
MCM9	92.97771	2.314131	0.500182	4.626581	3.72E-06	4.66E-05	TRUE	UP
NAA16	573.865	2.312902	0.406083	5.695633	1.23E-08	3.14E-07	TRUE	UP
RAB30	192.6514	2.310214	0.578076	3.996387	6.43E-05	0.000534	TRUE	UP
IQCD	103.9775	2.305042	0.658027	3.502962	0.00046	0.002776	TRUE	UP
UEVLD	379.6328	2.30187	0.45499	5.059167	4.21E-07	6.97E-06	TRUE	UP
SEC62	6727.084	2.300589	0.304076	7.565848	3.85E-14	2.84E-12	TRUE	UP
NUFIP1	332.9722	2.297704	0.331131	6.938959	3.95E-12	2.13E-10	TRUE	UP
OGFRL1	703.649	2.297504	0.506736	4.533927	5.79E-06	6.87E-05	TRUE	UP
TCP11L2	176.4441	2.291793	0.548201	4.180571	2.91E-05	0.000272	TRUE	UP
RHEBL1	104.8808	2.290808	0.638942	3.585315	0.000337	0.00213	TRUE	UP
LRRC46	31.81938	2.287914	0.607615	3.7654	0.000166	0.001186	TRUE	UP
ZMAT3	409.3637	2.268131	0.684168	3.315168	0.000916	0.004977	TRUE	UP
CLCF1	375.9626	2.266966	0.598431	3.788186	0.000152	0.001098	TRUE	UP

DPP8	655.2834	2.25942	0.4835	4.673052	2.97E-06	3.84E-05	TRUE	UP
DPEP2	163.7767	2.257126	0.734884	3.071406	0.002131	0.009958	TRUE	UP
DNAJC28	45.30897	2.250847	0.558382	4.031017	5.55E-05	0.000471	TRUE	UP
ZRSR2	772.9831	2.249454	0.406243	5.53721	3.07E-08	6.95E-07	TRUE	UP
GRK4	254.4594	2.248927	0.407929	5.513034	3.53E-08	7.88E-07	TRUE	UP
BMP2K	631.732	2.24503	0.507516	4.423561	9.71E-06	0.000107	TRUE	UP
PIWIL4	196.4898	2.243259	0.593114	3.782169	0.000155	0.00112	TRUE	UP
TPK1	309.5813	2.240928	0.529463	4.232456	2.31E-05	0.000227	TRUE	UP
TNRC6C	1654.037	2.239855	0.441331	5.075228	3.87E-07	6.45E-06	TRUE	UP
CETN3	706.3255	2.239215	0.298379	7.504606	6.16E-14	4.35E-12	TRUE	UP
PYROXD1	611.4778	2.236534	0.424213	5.27219	1.35E-07	2.57E-06	TRUE	UP
HOXC9	606.3735	2.234417	0.654702	3.412879	0.000643	0.003689	TRUE	UP
RC3H2	640.2126	2.232349	0.434713	5.13522	2.82E-07	4.86E-06	TRUE	UP
RP9	428.8326	2.232133	0.350153	6.374731	1.83E-10	7.16E-09	TRUE	UP
SNX12	445.9358	2.23067	0.365491	6.103212	1.04E-09	3.44E-08	TRUE	UP
PIK3CG	178.3464	2.227984	0.700275	3.181584	0.001465	0.007246	TRUE	UP
SLC35A3	330.9019	2.21972	0.354858	6.255241	3.97E-10	1.44E-08	TRUE	UP
PDP2	133.3999	2.217533	0.520423	4.261019	2.03E-05	0.000203	TRUE	UP
FNDC8	28.55283	2.215724	0.570057	3.886843	0.000102	0.000781	TRUE	UP
ZWINT	1332.933	2.20951	0.537263	4.112532	3.91E-05	0.000349	TRUE	UP
CCDC84	355.5416	2.193839	0.469614	4.671578	2.99E-06	3.86E-05	TRUE	UP
GUCA1B	111.4185	2.190983	0.676458	3.238905	0.0012	0.006185	TRUE	UP
PLEKHA8	128.8445	2.190815	0.535022	4.094815	4.23E-05	0.000373	TRUE	UP
FAM151B	46.39108	2.188797	0.558717	3.917544	8.95E-05	0.0007	TRUE	UP
CHIC1	833.3395	2.18156	0.404617	5.391669	6.98E-08	1.43E-06	TRUE	UP
PRDM5	235.9968	2.180963	0.535603	4.071977	4.66E-05	0.000406	TRUE	UP
SFT2D1	741.1633	2.17614	0.318609	6.830137	8.48E-12	4.25E-10	TRUE	UP
ADCY4	659.6503	2.175378	0.651638	3.338323	0.000843	0.004633	TRUE	UP
TATDN1	544.3896	2.174818	0.344939	6.304932	2.88E-10	1.08E-08	TRUE	UP
LIMS1	827.772	2.159467	0.540039	3.998723	6.37E-05	0.000529	TRUE	UP
PFDN6	1137.625	2.159098	0.388613	5.555913	2.76E-08	6.34E-07	TRUE	UP
THOC1	918.2584	2.158313	0.311065	6.938468	3.96E-12	2.13E-10	TRUE	UP
PRELID1	3612.007	2.158018	0.316709	6.813884	9.50E-12	4.75E-10	TRUE	UP
PSMC1	1264.734	2.157854	0.328088	6.577057	4.80E-11	2.10E-09	TRUE	UP
IQCB1	961.8149	2.155754	0.369175	5.839386	5.24E-09	1.46E-07	TRUE	UP
MAST4	845.5821	2.152049	0.597128	3.603998	0.000313	0.00201	TRUE	UP
BTBD19	113.1818	2.15054	0.576825	3.728236	0.000193	0.001342	TRUE	UP
SPCS2	1145.959	2.145692	0.377051	5.690722	1.27E-08	3.22E-07	TRUE	UP
NUDT4	2616.305	2.138312	0.496087	4.310353	1.63E-05	0.000167	TRUE	UP
GCLM	451.8928	2.137707	0.465836	4.588971	4.45E-06	5.45E-05	TRUE	UP
ZRANB1	2429.181	2.136612	0.332999	6.416277	1.40E-10	5.65E-09	TRUE	UP
TAF1D	1095.927	2.136272	0.362812	5.888102	3.91E-09	1.12E-07	TRUE	UP
ZNRF1	1340.727	2.128385	0.380268	5.597064	2.18E-08	5.15E-07	TRUE	UP
METTL3	1278.239	2.127027	0.348674	6.100337	1.06E-09	3.49E-08	TRUE	UP
PHKG2	1144.611	2.126934	0.32623	6.519737	7.04E-11	2.99E-09	TRUE	UP
TLR6	60.32198	2.12474	0.659848	3.220044	0.001282	0.00652	TRUE	UP
MAN2A1	1694.855	2.123933	0.581413	3.653052	0.000259	0.001726	TRUE	UP
SFI1	892.519	2.118855	0.492789	4.299718	1.71E-05	0.000174	TRUE	UP

KIF18A	297.6688	2.117104	0.651302	3.250573	0.001152	0.005975	TRUE	UP
TLCD1	141.8024	2.114243	0.553023	3.823066	0.000132	0.000969	TRUE	UP
HELLS	267.8038	2.11208	0.500575	4.219309	2.45E-05	0.000238	TRUE	UP
SH2B2	120.0929	2.096	0.632576	3.313434	0.000922	0.004996	TRUE	UP
FAM168A	438.7847	2.09278	0.573732	3.647659	0.000265	0.001754	TRUE	UP
CENPE	892.5842	2.091373	0.633801	3.299731	0.000968	0.005212	TRUE	UP
ANO8	861.7829	2.09097	0.495612	4.218969	2.45E-05	0.000238	TRUE	UP
ANAPC10	294.2058	2.089043	0.222891	9.372477	7.08E-21	1.26E-18	TRUE	UP
TIGD2	208.8313	2.076412	0.398491	5.210689	1.88E-07	3.42E-06	TRUE	UP
PRIM2	490.6259	2.075424	0.374736	5.538359	3.05E-08	6.91E-07	TRUE	UP
TMPPE	58.69628	2.073598	0.417471	4.967049	6.80E-07	1.05E-05	TRUE	UP
MMAA	415.7178	2.073276	0.362234	5.72358	1.04E-08	2.73E-07	TRUE	UP
SSR3	6009.029	2.069807	0.455499	4.54404	5.52E-06	6.59E-05	TRUE	UP
ADAM17	974.505	2.068898	0.433567	4.771804	1.83E-06	2.52E-05	TRUE	UP
NOP16	559.9395	2.06735	0.422455	4.893651	9.90E-07	1.48E-05	TRUE	UP
ERMP1	1052.285	2.06688	0.582956	3.545513	0.000392	0.002416	TRUE	UP
NEK8	168.1395	2.061934	0.427501	4.823225	1.41E-06	2.02E-05	TRUE	UP
FDXACB1	140.0946	2.057006	0.433056	4.749974	2.03E-06	2.75E-05	TRUE	UP
NEIL1	178.0282	2.0537	0.619499	3.315097	0.000916	0.004977	TRUE	UP
ERO1L	1506.688	2.047346	0.472606	4.332035	1.48E-05	0.000153	TRUE	UP
RSRC1	502.3602	2.045927	0.372448	5.493182	3.95E-08	8.67E-07	TRUE	UP
SLC25A45	269.335	2.045525	0.505097	4.049765	5.13E-05	0.00044	TRUE	UP
GUF1	1104.401	2.037253	0.313697	6.494324	8.34E-11	3.51E-09	TRUE	UP
GTF2F1	5141.196	2.034901	0.292171	6.96477	3.29E-12	1.83E-10	TRUE	UP
SLC25A37	949.9599	2.031639	0.540114	3.761499	0.000169	0.001201	TRUE	UP
ING1	822.8455	2.030053	0.309428	6.560664	5.36E-11	2.32E-09	TRUE	UP
EEF1E1	439.9008	2.023862	0.415162	4.874875	1.09E-06	1.61E-05	TRUE	UP
KIN	444.4478	2.023214	0.252163	8.023437	1.03E-15	9.95E-14	TRUE	UP
DOK1	1281.199	2.017493	0.495432	4.072188	4.66E-05	0.000406	TRUE	UP
TMEM106A	548.1664	2.012143	0.575374	3.497106	0.00047	0.002829	TRUE	UP
RBPJ	2244.914	2.012033	0.458048	4.392623	1.12E-05	0.000121	TRUE	UP
USMG5	1705.373	2.011205	0.33469	6.009165	1.86E-09	5.79E-08	TRUE	UP
EMG1	1041.899	2.0024	0.338164	5.921386	3.19E-09	9.37E-08	TRUE	UP
PANK3	725.298	2.000723	0.434973	4.599648	4.23E-06	5.21E-05	TRUE	UP
CCDC34	748.8995	1.999706	0.51301	3.89799	9.70E-05	0.00075	TRUE	UP
NAA35	701.1434	1.997768	0.326273	6.122997	9.18E-10	3.09E-08	TRUE	UP
PAOX	231.2061	1.994178	0.383018	5.20648	1.92E-07	3.49E-06	TRUE	UP
NFXL1	427.0911	1.991545	0.331982	5.998945	1.99E-09	6.11E-08	TRUE	UP
ZBTB7A	1872.441	1.985322	0.286611	6.926877	4.30E-12	2.28E-10	TRUE	UP
TECPR1	682.4773	1.982978	0.374937	5.288827	1.23E-07	2.37E-06	TRUE	UP
SNAPC5	283.6932	1.980975	0.383734	5.162367	2.44E-07	4.28E-06	TRUE	UP
ATL3	966.7198	1.979146	0.476295	4.155298	3.25E-05	0.0003	TRUE	UP
SLC16A13	97.93005	1.978261	0.580745	3.406422	0.000658	0.003765	TRUE	UP
CUEDC2	2733.144	1.978181	0.319161	6.198073	5.72E-10	2.01E-08	TRUE	UP
TULP4	1469.312	1.976787	0.269622	7.33171	2.27E-13	1.49E-11	TRUE	UP
IFNAR2	886.7785	1.975996	0.331751	5.956261	2.58E-09	7.81E-08	TRUE	UP
ZFHX3	2885.529	1.966078	0.43143	4.557118	5.19E-06	6.22E-05	TRUE	UP
ALKBH1	591.8105	1.962749	0.285489	6.875032	6.20E-12	3.18E-10	TRUE	UP

EIF2AK2	644.6844	1.952728	0.362935	5.38038	7.43E-08	1.51E-06	TRUE	UP
ZBTB26	160.6438	1.950149	0.560255	3.480821	0.0005	0.002976	TRUE	UP
PRKAR2A	516.1689	1.950067	0.584013	3.339082	0.000841	0.004622	TRUE	UP
NAA15	1669.72	1.946768	0.303231	6.420074	1.36E-10	5.53E-09	TRUE	UP
NRL	55.85112	1.945258	0.425748	4.569036	4.90E-06	5.92E-05	TRUE	UP
CHKA	913.2792	1.945067	0.447425	4.347247	1.38E-05	0.000144	TRUE	UP
WDFY2	428.8811	1.942382	0.450105	4.315401	1.59E-05	0.000164	TRUE	UP
GMEB1	360.4361	1.940509	0.275544	7.042459	1.89E-12	1.09E-10	TRUE	UP
RFXAP	273.9241	1.939972	0.358177	5.416246	6.09E-08	1.27E-06	TRUE	UP
MAPK8	352.6705	1.934213	0.455111	4.249983	2.14E-05	0.000212	TRUE	UP
ATE1	280.2354	1.931806	0.53071	3.64004	0.000273	0.001797	TRUE	UP
SKP2	318.4529	1.925983	0.535409	3.597219	0.000322	0.002051	TRUE	UP
PIGB	378.9227	1.921255	0.327116	5.873313	4.27E-09	1.21E-07	TRUE	UP
PTGR2	358.281	1.919852	0.369293	5.198727	2.01E-07	3.62E-06	TRUE	UP
PSMD12	1271.12	1.919816	0.239305	8.022469	1.04E-15	9.96E-14	TRUE	UP
SENP7	1014.942	1.917974	0.42345	4.529401	5.92E-06	6.97E-05	TRUE	UP
TTC5	374.419	1.916104	0.274969	6.968444	3.20E-12	1.80E-10	TRUE	UP
LEPROT	1225.747	1.914758	0.501259	3.819902	0.000134	0.000981	TRUE	UP
GLRX3	1503.291	1.908167	0.334279	5.708314	1.14E-08	2.96E-07	TRUE	UP
RSBN1	1063.953	1.907479	0.33125	5.758421	8.49E-09	2.25E-07	TRUE	UP
ASPSCR1	1393.848	1.906793	0.376967	5.058247	4.23E-07	6.99E-06	TRUE	UP
RNF19B	1481.629	1.906743	0.345094	5.525289	3.29E-08	7.39E-07	TRUE	UP
PCYT2	1128.56	1.900451	0.417014	4.55728	5.18E-06	6.22E-05	TRUE	UP
AGER	148.35	1.899522	0.550068	3.453251	0.000554	0.003263	TRUE	UP
APLF	138.6325	1.897125	0.371015	5.113342	3.17E-07	5.38E-06	TRUE	UP
LIAS	427.32	1.894773	0.351567	5.389506	7.07E-08	1.45E-06	TRUE	UP
IKBKE	406.5193	1.893175	0.547622	3.45708	0.000546	0.003223	TRUE	UP
SNRPA1	538.1279	1.890845	0.336325	5.622078	1.89E-08	4.53E-07	TRUE	UP
ORAOV1	631.3895	1.889451	0.489659	3.85871	0.000114	0.00086	TRUE	UP
TSPO	4070.161	1.887575	0.558011	3.382682	0.000718	0.004037	TRUE	UP
C1GALT1	679.1942	1.886722	0.553843	3.406599	0.000658	0.003765	TRUE	UP
MRPL33	1273.406	1.885887	0.335366	5.623367	1.87E-08	4.51E-07	TRUE	UP
WDR4	392.3229	1.8848	0.370541	5.086616	3.65E-07	6.11E-06	TRUE	UP
DCUN1D5	845.2195	1.883197	0.379984	4.955992	7.20E-07	1.11E-05	TRUE	UP
NVL	922.9871	1.87771	0.273578	6.863526	6.72E-12	3.42E-10	TRUE	UP
MAD2L1	841.7222	1.875612	0.498869	3.759726	0.00017	0.001208	TRUE	UP
FIP1L1	1531.267	1.873241	0.247883	7.556959	4.13E-14	3.02E-12	TRUE	UP
ENTPD5	282.1187	1.860924	0.521799	3.566362	0.000362	0.002259	TRUE	UP
AMMECR1	583.5159	1.859364	0.559681	3.322185	0.000893	0.004874	TRUE	UP
TMED5	3153.436	1.855415	0.42351	4.38104	1.18E-05	0.000127	TRUE	UP
TNRC6A	2795.149	1.854542	0.360548	5.143671	2.69E-07	4.67E-06	TRUE	UP
AUH	482.4255	1.848543	0.568154	3.253594	0.00114	0.005932	TRUE	UP
SERINC3	4821.459	1.845485	0.304906	6.052634	1.42E-09	4.52E-08	TRUE	UP
BAK1	836.8301	1.845071	0.362378	5.091568	3.55E-07	5.97E-06	TRUE	UP
SMPD2	355.521	1.844974	0.461372	3.998886	6.36E-05	0.000529	TRUE	UP
ANAPC1	1059.056	1.8444	0.316736	5.823141	5.78E-09	1.59E-07	TRUE	UP
ABCA7	618.4549	1.844061	0.576291	3.199881	0.001375	0.006905	TRUE	UP
NPM3	954.4551	1.842249	0.472474	3.899151	9.65E-05	0.000747	TRUE	UP

RPS27A	9751.639	1.830797	0.431087	4.246934	2.17E-05	0.000214	TRUE	UP
XYLB	199.9926	1.830229	0.474642	3.85602	0.000115	0.000868	TRUE	UP
DOCK5	563.9886	1.829551	0.587459	3.114345	0.001844	0.008805	TRUE	UP
LCORL	236.6462	1.827704	0.443981	4.116622	3.84E-05	0.000345	TRUE	UP
FZD5	269.5878	1.826739	0.537766	3.396906	0.000682	0.003874	TRUE	UP
CASP8	791.3495	1.825748	0.420626	4.340547	1.42E-05	0.000148	TRUE	UP
MRPL13	1205.222	1.818713	0.29009	6.269482	3.62E-10	1.33E-08	TRUE	UP
TRIM3	752.5438	1.817059	0.323542	5.616147	1.95E-08	4.66E-07	TRUE	UP
PLK4	510.1646	1.816445	0.545967	3.327022	0.000878	0.004804	TRUE	UP
DNLZ	543.454	1.81615	0.480986	3.775885	0.000159	0.001144	TRUE	UP
TRAF3	550.4981	1.812641	0.468915	3.865607	0.000111	0.000843	TRUE	UP
MPND	1090.726	1.803022	0.284214	6.343895	2.24E-10	8.59E-09	TRUE	UP
MAGI3	748.6416	1.802927	0.48759	3.697626	0.000218	0.00149	TRUE	UP
P2RX4	1408.688	1.80111	0.449595	4.006071	6.17E-05	0.000516	TRUE	UP
CDK7	564.9231	1.799018	0.320184	5.618693	1.92E-08	4.61E-07	TRUE	UP
ZBTB25	236.5079	1.798566	0.495253	3.63161	0.000282	0.001846	TRUE	UP
TBC1D19	336.9924	1.797675	0.426744	4.212542	2.53E-05	0.000244	TRUE	UP
TRIM59	537.2191	1.795966	0.563931	3.184727	0.001449	0.007183	TRUE	UP
UBA5	1350.273	1.795639	0.229903	7.810411	5.70E-15	4.67E-13	TRUE	UP
FBXL6	632.1485	1.795176	0.370472	4.845644	1.26E-06	1.83E-05	TRUE	UP
AKAP8L	2741.255	1.790645	0.357002	5.01579	5.28E-07	8.48E-06	TRUE	UP
XRCC4	287.8274	1.790562	0.416121	4.302986	1.69E-05	0.000172	TRUE	UP
NUDT9	1381.459	1.789531	0.233691	7.657682	1.89E-14	1.46E-12	TRUE	UP
RBM45	319.5803	1.786177	0.178675	9.99677	1.57E-23	3.82E-21	TRUE	UP
LGALS1	39983.51	1.786038	0.50056	3.568081	0.00036	0.002249	TRUE	UP
TRMT2B	477.0407	1.78488	0.362636	4.92196	8.57E-07	1.29E-05	TRUE	UP
PAK1IP1	715.7972	1.784746	0.34544	5.166581	2.38E-07	4.20E-06	TRUE	UP
CLPB	590.9656	1.784289	0.354386	5.034878	4.78E-07	7.77E-06	TRUE	UP
RNF168	309.7004	1.784234	0.499755	3.570215	0.000357	0.002234	TRUE	UP
INO80D	959.7198	1.781772	0.292447	6.092625	1.11E-09	3.62E-08	TRUE	UP
PPID	1499.961	1.779381	0.277694	6.407706	1.48E-10	5.87E-09	TRUE	UP
TET3	829.8807	1.779089	0.452475	3.931907	8.43E-05	0.000667	TRUE	UP
NIP7	843.9708	1.778682	0.346482	5.133549	2.84E-07	4.89E-06	TRUE	UP
AP1S1	1683.771	1.776354	0.374131	4.74795	2.05E-06	2.77E-05	TRUE	UP
MSTO1	1350.145	1.774843	0.428916	4.13797	3.50E-05	0.000318	TRUE	UP
SLC35E4	410.6181	1.773878	0.528649	3.355491	0.000792	0.00439	TRUE	UP
FUT11	503.024	1.771919	0.536011	3.305749	0.000947	0.005111	TRUE	UP
TRMT1	1932.271	1.769067	0.381315	4.639386	3.49E-06	4.42E-05	TRUE	UP
SLC41A2	390.1436	1.765935	0.565761	3.121346	0.0018	0.008631	TRUE	UP
ANAPC2	1995.262	1.765526	0.284789	6.199425	5.67E-10	2.00E-08	TRUE	UP
CHRNB1	393.4701	1.765108	0.547634	3.223152	0.001268	0.006468	TRUE	UP
HSPE1	2433.344	1.762797	0.364206	4.840111	1.30E-06	1.87E-05	TRUE	UP
TIRAP	298.4694	1.75315	0.388697	4.510329	6.47E-06	7.54E-05	TRUE	UP
C1D	520.4039	1.750662	0.325951	5.370932	7.83E-08	1.58E-06	TRUE	UP
ANKRD39	320.2813	1.747678	0.321731	5.432109	5.57E-08	1.18E-06	TRUE	UP
PDCL3	917.6276	1.746517	0.296889	5.882728	4.04E-09	1.15E-07	TRUE	UP
FAM193B	1986.436	1.744712	0.417376	4.180192	2.91E-05	0.000272	TRUE	UP
CRK	1737.439	1.743912	0.333173	5.234246	1.66E-07	3.06E-06	TRUE	UP

PHTF1	682.5627	1.743243	0.297614	5.857386	4.70E-09	1.33E-07	TRUE	UP
GAR1	739.6579	1.739075	0.308233	5.642075	1.68E-08	4.14E-07	TRUE	UP
POLB	541.7548	1.736246	0.301155	5.765298	8.15E-09	2.17E-07	TRUE	UP
DTYMK	1491.28	1.732279	0.441366	3.924816	8.68E-05	0.000684	TRUE	UP
POLR2F	2128.581	1.73013	0.348793	4.960332	7.04E-07	1.08E-05	TRUE	UP
MFN1	1178.559	1.730092	0.330511	5.234602	1.65E-07	3.06E-06	TRUE	UP
HEATR3	541.6366	1.729321	0.412457	4.192732	2.76E-05	0.000262	TRUE	UP
COMMD8	665.085	1.729104	0.358494	4.823248	1.41E-06	2.02E-05	TRUE	UP
RNF126	1326.643	1.72052	0.366445	4.695163	2.66E-06	3.49E-05	TRUE	UP
SPAST	992.9974	1.720288	0.385192	4.466058	7.97E-06	8.93E-05	TRUE	UP
PSMC6	1553.851	1.719436	0.263347	6.529156	6.61E-11	2.83E-09	TRUE	UP
GRAMD1C	110.9801	1.716753	0.556186	3.08665	0.002024	0.009533	TRUE	UP
SSBP3	1205.448	1.713324	0.472462	3.626374	0.000287	0.001875	TRUE	UP
PRELID2	123.6327	1.712471	0.537262	3.187404	0.001436	0.007129	TRUE	UP
TARBP2	1131.741	1.709544	0.537279	3.181854	0.001463	0.007241	TRUE	UP
RNPC3	294.3902	1.708854	0.504306	3.388528	0.000703	0.003967	TRUE	UP
EHMT2	3656.169	1.707776	0.327252	5.218537	1.80E-07	3.30E-06	TRUE	UP
AMDHD2	859.4511	1.706857	0.460667	3.705183	0.000211	0.001449	TRUE	UP
PDCD2L	271.8423	1.698732	0.41325	4.110659	3.95E-05	0.000351	TRUE	UP
SPSB2	412.4301	1.695249	0.396509	4.275434	1.91E-05	0.000191	TRUE	UP
RPS6KB2	1861.115	1.694377	0.34283	4.942324	7.72E-07	1.18E-05	TRUE	UP
PRPF39	1033.891	1.691428	0.304182	5.560586	2.69E-08	6.19E-07	TRUE	UP
DGKA	1039.156	1.684883	0.425236	3.962231	7.43E-05	0.0006	TRUE	UP
TMCC1	1305.674	1.684142	0.281502	5.982691	2.19E-09	6.69E-08	TRUE	UP
PSMA3	2351.934	1.683406	0.311427	5.405459	6.46E-08	1.34E-06	TRUE	UP
SPDYA	31.63567	1.682883	0.478599	3.516266	0.000438	0.002654	TRUE	UP
ENY2	1356.431	1.678929	0.302285	5.554122	2.79E-08	6.39E-07	TRUE	UP
MRPL48	656.2147	1.67862	0.369433	4.543772	5.53E-06	6.60E-05	TRUE	UP
TAF1A	188.2642	1.677239	0.400527	4.18758	2.82E-05	0.000266	TRUE	UP
TARS2	1100	1.675594	0.306501	5.466848	4.58E-08	9.93E-07	TRUE	UP
RAB2A	2176.985	1.674001	0.276969	6.044004	1.50E-09	4.76E-08	TRUE	UP
CYHR1	1942.7	1.673828	0.358289	4.671724	2.99E-06	3.86E-05	TRUE	UP
PARP3	1335.699	1.667641	0.452672	3.683995	0.00023	0.001557	TRUE	UP
PLA2G6	572.3791	1.667487	0.533228	3.127153	0.001765	0.008474	TRUE	UP
RBM28	750.9219	1.665412	0.297906	5.590392	2.27E-08	5.33E-07	TRUE	UP
CFLAR	1539.159	1.664705	0.343003	4.853331	1.21E-06	1.77E-05	TRUE	UP
MCOLN1	2174.27	1.664042	0.409971	4.05893	4.93E-05	0.000426	TRUE	UP
RBM7	1008.61	1.660581	0.320196	5.186142	2.15E-07	3.85E-06	TRUE	UP
PPIC	3590.774	1.659528	0.511003	3.247591	0.001164	0.006029	TRUE	UP
FGFR1OP	386.8193	1.659057	0.387928	4.276718	1.90E-05	0.00019	TRUE	UP
SIRT7	583.1182	1.658471	0.390567	4.246312	2.17E-05	0.000214	TRUE	UP
CHEK2	656.3952	1.654724	0.487768	3.39244	0.000693	0.003927	TRUE	UP
SLC36A4	436.9195	1.653831	0.465711	3.551196	0.000383	0.002374	TRUE	UP
PHF7	175.0981	1.6523	0.530704	3.113411	0.001849	0.008827	TRUE	UP
SSH2	1191.297	1.651045	0.417266	3.956821	7.60E-05	0.000611	TRUE	UP
CHORDC1	698.5311	1.649197	0.342059	4.821375	1.43E-06	2.03E-05	TRUE	UP
COMMD1	685.6576	1.648518	0.406763	4.052771	5.06E-05	0.000436	TRUE	UP
PIAS1	815.7704	1.648034	0.353791	4.658212	3.19E-06	4.09E-05	TRUE	UP

LSM6	661.5275	1.647822	0.322175	5.114677	3.14E-07	5.35E-06	TRUE	UP
CDADC1	389.2551	1.647633	0.375637	4.386234	1.15E-05	0.000124	TRUE	UP
METTL14	586.0362	1.647293	0.355464	4.634201	3.58E-06	4.52E-05	TRUE	UP
ZNHIT2	378.291	1.646016	0.531036	3.09963	0.001938	0.0092	TRUE	UP
FGFR1OP2	1128.506	1.641855	0.291192	5.638386	1.72E-08	4.21E-07	TRUE	UP
IDE	648.5992	1.640229	0.464726	3.529454	0.000416	0.002541	TRUE	UP
RPL6	13287.16	1.640025	0.477712	3.433085	0.000597	0.00347	TRUE	UP
RPL7L1	1414.172	1.634464	0.338825	4.823924	1.41E-06	2.01E-05	TRUE	UP
MRPS17	599.0005	1.631385	0.394102	4.139501	3.48E-05	0.000316	TRUE	UP
MYBBP1A	2551.31	1.630783	0.36658	4.448641	8.64E-06	9.59E-05	TRUE	UP
LSM7	1880.231	1.629047	0.392323	4.152313	3.29E-05	0.000302	TRUE	UP
DUSP18	188.2497	1.62868	0.341725	4.76605	1.88E-06	2.58E-05	TRUE	UP
PDSS1	156.1254	1.627297	0.358253	4.542313	5.56E-06	6.64E-05	TRUE	UP
MDP1	535.355	1.627062	0.323425	5.030723	4.89E-07	7.93E-06	TRUE	UP
UTP15	441.0687	1.626258	0.291588	5.577246	2.44E-08	5.69E-07	TRUE	UP
HAUS3	767.1378	1.621216	0.315385	5.140433	2.74E-07	4.73E-06	TRUE	UP
HSD17B7	275.1806	1.619549	0.358937	4.512071	6.42E-06	7.50E-05	TRUE	UP
ZCRB1	2012.404	1.618274	0.377327	4.288781	1.80E-05	0.000182	TRUE	UP
WDR74	1350.31	1.617548	0.358724	4.509173	6.51E-06	7.57E-05	TRUE	UP
TCIRG1	4145.456	1.615234	0.490289	3.294456	0.000986	0.005295	TRUE	UP
AMN1	382.1628	1.614861	0.408832	3.949941	7.82E-05	0.000626	TRUE	UP
DRAP1	4523.439	1.614103	0.413256	3.905822	9.39E-05	0.00073	TRUE	UP
ANKMY1	360.5433	1.612696	0.463917	3.476263	0.000508	0.003022	TRUE	UP
NOL9	387.4868	1.611634	0.291504	5.528692	3.23E-08	7.26E-07	TRUE	UP
U2AF1L4	431.5096	1.611046	0.477017	3.377336	0.000732	0.004105	TRUE	UP
DTNB	434.688	1.610571	0.472798	3.406464	0.000658	0.003765	TRUE	UP
POLR1C	686.682	1.607666	0.313178	5.13339	2.85E-07	4.89E-06	TRUE	UP
IPO4	1729.403	1.603664	0.349517	4.588231	4.47E-06	5.46E-05	TRUE	UP
FAM185A	173.1332	1.60363	0.335532	4.77936	1.76E-06	2.44E-05	TRUE	UP
DBF4	465.9916	1.602626	0.38951	4.114461	3.88E-05	0.000347	TRUE	UP
CCDC9	1208.512	1.598461	0.268986	5.942538	2.81E-09	8.42E-08	TRUE	UP
RBMS1	4994.115	1.595652	0.406643	3.923964	8.71E-05	0.000685	TRUE	UP
CDKL3	118.3853	1.594948	0.412387	3.8676	0.00011	0.000837	TRUE	UP
PRPF38B	2428.782	1.588963	0.235624	6.743627	1.54E-11	7.32E-10	TRUE	UP
E4F1	1384.228	1.588107	0.301521	5.266993	1.39E-07	2.63E-06	TRUE	UP
SNRPG	1094.364	1.58279	0.292544	5.410424	6.29E-08	1.30E-06	TRUE	UP
TTC13	575.2411	1.579452	0.376393	4.19628	2.71E-05	0.000259	TRUE	UP
AKR1A1	5047.386	1.578525	0.349168	4.520822	6.16E-06	7.23E-05	TRUE	UP
CYB5R4	662.0682	1.578185	0.412447	3.826398	0.00013	0.000959	TRUE	UP
SENP8	92.13087	1.577073	0.444527	3.547752	0.000389	0.0024	TRUE	UP
SLC38A7	458.5779	1.575591	0.464295	3.393515	0.00069	0.003914	TRUE	UP
PTMA	25570.27	1.574764	0.27053	5.821034	5.85E-09	1.61E-07	TRUE	UP
EEF1B2	6073.714	1.573228	0.36106	4.35725	1.32E-05	0.000139	TRUE	UP
RRAGC	906.976	1.570368	0.37629	4.173291	3.00E-05	0.00028	TRUE	UP
NISCH	5890.68	1.569564	0.31544	4.975785	6.50E-07	1.01E-05	TRUE	UP
UBE2R2	5150.658	1.569057	0.278156	5.640925	1.69E-08	4.16E-07	TRUE	UP
FDX1L	831.9796	1.568868	0.404613	3.877454	0.000106	0.000809	TRUE	UP
SNRPE	1113.936	1.566654	0.31421	4.986007	6.16E-07	9.72E-06	TRUE	UP

MAFG	1906.341	1.566474	0.403567	3.881569	0.000104	0.000796	TRUE	UP
FUT10	253.1514	1.564286	0.444068	3.522627	0.000427	0.0026	TRUE	UP
CCAR1	1958.423	1.563426	0.275582	5.673176	1.40E-08	3.53E-07	TRUE	UP
STRBP	635.6482	1.557717	0.499671	3.117485	0.001824	0.008717	TRUE	UP
ELMOD2	254.5421	1.556261	0.345981	4.498105	6.86E-06	7.91E-05	TRUE	UP
IQCC	241.215	1.555961	0.440393	3.533122	0.000411	0.002519	TRUE	UP
MRPL24	2683.506	1.551804	0.386047	4.01973	5.83E-05	0.000491	TRUE	UP
FAM98B	515.8954	1.549728	0.395625	3.917161	8.96E-05	0.0007	TRUE	UP
TUBGCP4	539.709	1.549607	0.440036	3.521545	0.000429	0.002608	TRUE	UP
TUT1	1228.821	1.548696	0.281647	5.498715	3.83E-08	8.46E-07	TRUE	UP
CUEDC1	1276.771	1.548021	0.394751	3.921515	8.80E-05	0.00069	TRUE	UP
ANKZF1	1063.614	1.543026	0.353393	4.366319	1.26E-05	0.000134	TRUE	UP
MRPL52	1605.296	1.542952	0.370146	4.16849	3.07E-05	0.000285	TRUE	UP
SUGT1	1081.216	1.541622	0.343291	4.490717	7.10E-06	8.13E-05	TRUE	UP
FBXO4	571.1199	1.540736	0.355693	4.33165	1.48E-05	0.000153	TRUE	UP
GTPBP4	1740.139	1.53822	0.323529	4.754508	1.99E-06	2.70E-05	TRUE	UP
TNKS2	2721.582	1.538165	0.362096	4.247947	2.16E-05	0.000213	TRUE	UP
SAAL1	379.645	1.537168	0.352446	4.361426	1.29E-05	0.000137	TRUE	UP
ERGIC2	1747.513	1.534138	0.311052	4.9321	8.14E-07	1.23E-05	TRUE	UP
UHRF1BP1I	1241.054	1.533044	0.430408	3.56184	0.000368	0.002294	TRUE	UP
SRP19	1358.407	1.532528	0.229469	6.678576	2.41E-11	1.10E-09	TRUE	UP
CRLF3	599.5846	1.532378	0.328628	4.662949	3.12E-06	4.01E-05	TRUE	UP
NEK3	353.9599	1.532179	0.428712	3.573914	0.000352	0.002209	TRUE	UP
SIPA1L3	1464.91	1.531304	0.473158	3.236346	0.001211	0.006227	TRUE	UP
FUBP1	1963.618	1.527696	0.348771	4.380223	1.19E-05	0.000127	TRUE	UP
RANBP1	2131.693	1.526726	0.320214	4.767837	1.86E-06	2.56E-05	TRUE	UP
CCDC102A	822.9924	1.524947	0.481567	3.166638	0.001542	0.007565	TRUE	UP
UBAP2	1441.92	1.522488	0.301482	5.050009	4.42E-07	7.27E-06	TRUE	UP
COX6A1	3634.966	1.519023	0.385789	3.937445	8.24E-05	0.000654	TRUE	UP
ZC3H7A	2447.216	1.515816	0.279618	5.421021	5.93E-08	1.24E-06	TRUE	UP
BBS12	239.9186	1.507963	0.382212	3.945356	7.97E-05	0.000637	TRUE	UP
SPPL2A	1372.789	1.505886	0.348814	4.317165	1.58E-05	0.000162	TRUE	UP
LDB1	2453.573	1.501669	0.397867	3.774299	0.00016	0.00115	TRUE	UP
SCAI	606.3132	1.494531	0.463672	3.223253	0.001267	0.006468	TRUE	UP
DOHH	925.2014	1.494172	0.280801	5.321103	1.03E-07	2.03E-06	TRUE	UP
WDR43	1482.718	1.493882	0.335785	4.448926	8.63E-06	9.58E-05	TRUE	UP
POLR3K	499.8368	1.492529	0.282326	5.286554	1.25E-07	2.39E-06	TRUE	UP
MAPKAPK5	1126.601	1.49045	0.412343	3.614585	0.000301	0.00195	TRUE	UP
ZCCHC7	937.9106	1.489925	0.283204	5.260968	1.43E-07	2.71E-06	TRUE	UP
PAIP1	1630.911	1.489758	0.240955	6.182715	6.30E-10	2.20E-08	TRUE	UP
SNX24	486.3721	1.488728	0.456987	3.257702	0.001123	0.005858	TRUE	UP
TET2	898.2187	1.488678	0.365312	4.07509	4.60E-05	0.000401	TRUE	UP
BHLHB9	344.4379	1.485415	0.432023	3.438276	0.000585	0.003409	TRUE	UP
TSC22D2	1275.979	1.483576	0.379611	3.908148	9.30E-05	0.000725	TRUE	UP
REXO2	2304.581	1.481408	0.380619	3.892098	9.94E-05	0.000766	TRUE	UP
USPL1	775.8261	1.480861	0.402877	3.675719	0.000237	0.001603	TRUE	UP
ATP5J2	3379.984	1.477765	0.408784	3.615029	0.0003	0.001948	TRUE	UP
LYAR	819.3973	1.477219	0.335286	4.405845	1.05E-05	0.000115	TRUE	UP

INVS	626.8744	1.476655	0.361458	4.085273	4.40E-05	0.000386	TRUE	UP
COX17	816.5987	1.473366	0.409456	3.598355	0.00032	0.002045	TRUE	UP
CTU1	162.0205	1.473173	0.457091	3.222936	0.001269	0.006471	TRUE	UP
MED31	322.8988	1.472819	0.337516	4.363705	1.28E-05	0.000136	TRUE	UP
LCLAT1	699.9374	1.472638	0.35425	4.157059	3.22E-05	0.000298	TRUE	UP
SNRPD1	1617.503	1.472448	0.309504	4.75744	1.96E-06	2.67E-05	TRUE	UP
PRKACB	3101.584	1.469634	0.336592	4.366213	1.26E-05	0.000134	TRUE	UP
PRKCD	1678.289	1.466614	0.40381	3.631943	0.000281	0.001845	TRUE	UP
SNX5	3391.973	1.46327	0.334887	4.369446	1.25E-05	0.000133	TRUE	UP
OTUD6B	495.3378	1.461933	0.363157	4.025623	5.68E-05	0.00048	TRUE	UP
NFKB1	2727.553	1.460829	0.342519	4.264956	2.00E-05	0.0002	TRUE	UP
AVL9	376.2331	1.460043	0.403896	3.614895	0.0003	0.001948	TRUE	UP
ZBTB8OS	429.6988	1.459359	0.362551	4.025246	5.69E-05	0.00048	TRUE	UP
KIF3A	648.3841	1.457605	0.356304	4.090909	4.30E-05	0.000378	TRUE	UP
PAN3	1196.26	1.457481	0.316722	4.601773	4.19E-06	5.18E-05	TRUE	UP
UBR2	1411.889	1.456784	0.469384	3.103607	0.001912	0.009102	TRUE	UP
PDE7A	1045.981	1.455567	0.431736	3.371429	0.000748	0.00418	TRUE	UP
TRIM35	1093.186	1.454733	0.301985	4.817237	1.46E-06	2.06E-05	TRUE	UP
MARS2	168.2433	1.45186	0.398728	3.641232	0.000271	0.001792	TRUE	UP
SETD6	386.6154	1.451773	0.382462	3.795857	0.000147	0.001066	TRUE	UP
USP45	339.0789	1.45131	0.340784	4.25874	2.06E-05	0.000204	TRUE	UP
CDK5	763.5541	1.442665	0.360786	3.998678	6.37E-05	0.000529	TRUE	UP
PPOX	594.6424	1.441959	0.401592	3.590611	0.00033	0.002096	TRUE	UP
ZCWPW1	321.6984	1.439743	0.439884	3.273003	0.001064	0.005622	TRUE	UP
NOP14	1922.973	1.437262	0.303624	4.733696	2.20E-06	2.94E-05	TRUE	UP
TXNDC17	1057.989	1.437069	0.448848	3.201682	0.001366	0.006869	TRUE	UP
SLC25A17	981.6442	1.435744	0.264727	5.423487	5.84E-08	1.23E-06	TRUE	UP
NUDCD1	805.132	1.435595	0.358459	4.004911	6.20E-05	0.000518	TRUE	UP
DCAF13	1509.928	1.434958	0.363242	3.950416	7.80E-05	0.000625	TRUE	UP
APBA3	1004.169	1.429165	0.311688	4.585241	4.53E-06	5.53E-05	TRUE	UP
CDC16	2491.407	1.429	0.297146	4.809083	1.52E-06	2.14E-05	TRUE	UP
ZMYM5	567.9568	1.428979	0.307605	4.645505	3.39E-06	4.31E-05	TRUE	UP
CSTF2	522.2507	1.428358	0.356424	4.007462	6.14E-05	0.000513	TRUE	UP
TBP	640.6152	1.426223	0.227757	6.262046	3.80E-10	1.38E-08	TRUE	UP
ACBD5	969.0857	1.425224	0.358136	3.97956	6.90E-05	0.000567	TRUE	UP
AEBP2	1463.704	1.42489	0.357758	3.982829	6.81E-05	0.000561	TRUE	UP
BCL10	893.7346	1.42327	0.35172	4.0466	5.20E-05	0.000444	TRUE	UP
CDK11B	2094.034	1.422222	0.285173	4.987232	6.13E-07	9.67E-06	TRUE	UP
WBP4	685.9529	1.420335	0.308524	4.603642	4.15E-06	5.13E-05	TRUE	UP
TDRD3	746.3322	1.418601	0.436073	3.25313	0.001141	0.005939	TRUE	UP
TMEM206	380.2247	1.417239	0.412109	3.438988	0.000584	0.003406	TRUE	UP
INO80C	506.203	1.417044	0.397272	3.566936	0.000361	0.002256	TRUE	UP
PIAS4	788.2709	1.413904	0.348301	4.05943	4.92E-05	0.000425	TRUE	UP
TFB2M	486.7247	1.413273	0.325582	4.340756	1.42E-05	0.000148	TRUE	UP
HSPA8	33364.99	1.412215	0.342014	4.129114	3.64E-05	0.000328	TRUE	UP
METTL6	668.7419	1.411264	0.262143	5.383575	7.30E-08	1.49E-06	TRUE	UP
CASP6	488.4314	1.41062	0.326019	4.326805	1.51E-05	0.000156	TRUE	UP
POLG2	255.642	1.405773	0.319335	4.402197	1.07E-05	0.000117	TRUE	UP

LSM12	1680.692	1.405127	0.256051	5.487676	4.07E-08	8.92E-07	TRUE	UP
KLHDC4	601.269	1.404458	0.390511	3.59646	0.000323	0.002055	TRUE	UP
RCHY1	712.9219	1.402561	0.269886	5.196873	2.03E-07	3.65E-06	TRUE	UP
KCTD17	1102.021	1.400773	0.382005	3.666895	0.000246	0.001649	TRUE	UP
EIF4G3	3757.436	1.398875	0.354509	3.945952	7.95E-05	0.000636	TRUE	UP
DENND4A	822.8012	1.398664	0.420808	3.323761	0.000888	0.004849	TRUE	UP
MAST3	966.4442	1.397491	0.381764	3.660612	0.000252	0.001683	TRUE	UP
POP1	301.9646	1.39424	0.42328	3.293894	0.000988	0.005299	TRUE	UP
DIRC2	481.5195	1.393636	0.331985	4.197884	2.69E-05	0.000257	TRUE	UP
RHBDF1	1633.73	1.392413	0.383907	3.626951	0.000287	0.001872	TRUE	UP
MED7	458.8093	1.391003	0.240373	5.786856	7.17E-09	1.93E-07	TRUE	UP
PIGM	548.6219	1.386793	0.386418	3.588842	0.000332	0.002107	TRUE	UP
SEC61A2	311.7471	1.383162	0.37675	3.671304	0.000241	0.001625	TRUE	UP
PCYT1A	612.4517	1.378314	0.325021	4.240686	2.23E-05	0.000219	TRUE	UP
YOD1	445.559	1.377414	0.418987	3.287487	0.001011	0.005381	TRUE	UP
BOLA3	527.6935	1.377009	0.364253	3.780365	0.000157	0.001127	TRUE	UP
OTUD4	1542.606	1.374852	0.320131	4.294652	1.75E-05	0.000178	TRUE	UP
ATP6V0A2	970.8381	1.373667	0.31229	4.398693	1.09E-05	0.000119	TRUE	UP
G3BP1	4167.072	1.372281	0.274629	4.996849	5.83E-07	9.25E-06	TRUE	UP
TXNDC9	913.4473	1.371141	0.293337	4.674293	2.95E-06	3.82E-05	TRUE	UP
VDAC2	2451.864	1.369909	0.349684	3.917563	8.94E-05	0.0007	TRUE	UP
RSBN1L	752.3855	1.366555	0.391047	3.494605	0.000475	0.00285	TRUE	UP
PTCD2	345.661	1.365901	0.326709	4.180787	2.91E-05	0.000272	TRUE	UP
CCNL1	2033.713	1.365477	0.325456	4.195582	2.72E-05	0.000259	TRUE	UP
NOP58	2276.791	1.364311	0.250569	5.444852	5.18E-08	1.11E-06	TRUE	UP
PUS10	229.8143	1.362307	0.351728	3.873186	0.000107	0.000821	TRUE	UP
LRIG2	391.6421	1.361901	0.388187	3.50836	0.000451	0.002727	TRUE	UP
ARHGEF7	2581.763	1.361835	0.349113	3.900847	9.59E-05	0.000743	TRUE	UP
HEATR5A	1381.075	1.359287	0.415956	3.267862	0.001084	0.005701	TRUE	UP
PGS1	1258.741	1.356758	0.257969	5.259381	1.45E-07	2.73E-06	TRUE	UP
AP4M1	861.7513	1.356695	0.289248	4.690418	2.73E-06	3.55E-05	TRUE	UP
ALG5	1098.087	1.35212	0.334103	4.047011	5.19E-05	0.000444	TRUE	UP
BNIP2	2145.722	1.352004	0.269204	5.022235	5.11E-07	8.25E-06	TRUE	UP
SSBP4	2579.5	1.35021	0.375286	3.597817	0.000321	0.002048	TRUE	UP
WDR12	674.7683	1.347339	0.32934	4.091031	4.29E-05	0.000378	TRUE	UP
GEMIN7	531.7515	1.345182	0.335038	4.015017	5.94E-05	0.0005	TRUE	UP
MRM1	261.0714	1.342289	0.339586	3.952716	7.73E-05	0.00062	TRUE	UP
TNPO3	1870.247	1.338462	0.347622	3.850343	0.000118	0.000884	TRUE	UP
GTPBP10	456.3429	1.338054	0.304846	4.389278	1.14E-05	0.000123	TRUE	UP
GRAMD4	1286.746	1.336948	0.383015	3.490587	0.000482	0.002884	TRUE	UP
SOCS5	1431.833	1.331212	0.395667	3.364475	0.000767	0.00427	TRUE	UP
ATP5S	164.9062	1.329789	0.367501	3.618458	0.000296	0.001928	TRUE	UP
NUP88	1340.998	1.328875	0.278533	4.770984	1.83E-06	2.53E-05	TRUE	UP
TMEM164	889.1361	1.328655	0.368041	3.610073	0.000306	0.001976	TRUE	UP
COX19	680.3845	1.326966	0.364728	3.638235	0.000275	0.001806	TRUE	UP
NSMCE2	665.1935	1.325984	0.320635	4.135498	3.54E-05	0.000321	TRUE	UP
NOP2	1479.394	1.325277	0.368996	3.591571	0.000329	0.00209	TRUE	UP
ATP6V0C	6553.664	1.322956	0.370928	3.566607	0.000362	0.002258	TRUE	UP

CORO1B	4596.938	1.321056	0.326393	4.047442	5.18E-05	0.000444	TRUE	UP
G2E3	776.762	1.317371	0.399984	3.293562	0.000989	0.005301	TRUE	UP
TTC14	1301.09	1.317159	0.417727	3.153156	0.001615	0.007877	TRUE	UP
DGKZ	1494.011	1.316225	0.336475	3.911809	9.16E-05	0.000715	TRUE	UP
BANP	713.4872	1.314575	0.268806	4.890426	1.01E-06	1.50E-05	TRUE	UP
RAB3GAP2	1572.982	1.314379	0.303507	4.330634	1.49E-05	0.000154	TRUE	UP
SDCCAG8	941.9125	1.313342	0.317667	4.134333	3.56E-05	0.000322	TRUE	UP
DHODH	276.5027	1.313097	0.403937	3.250751	0.001151	0.005973	TRUE	UP
BPTF	2965.374	1.311708	0.427082	3.071326	0.002131	0.009958	TRUE	UP
PGAP2	604.1759	1.308362	0.33041	3.959813	7.50E-05	0.000605	TRUE	UP
BRPF1	1548.418	1.308101	0.260441	5.022648	5.10E-07	8.24E-06	TRUE	UP
ALKBH8	382.8093	1.307405	0.31487	4.152209	3.29E-05	0.000302	TRUE	UP
CTU2	725.3407	1.304405	0.362416	3.599194	0.000319	0.00204	TRUE	UP
RNF214	671.0032	1.302873	0.289087	4.506856	6.58E-06	7.63E-05	TRUE	UP
KIF2A	1038.563	1.29906	0.376428	3.451018	0.000558	0.003287	TRUE	UP
ING3	505.4433	1.29859	0.291826	4.449879	8.59E-06	9.55E-05	TRUE	UP
ALG14	351.267	1.297366	0.40407	3.210747	0.001324	0.006691	TRUE	UP
ATP9B	877.4179	1.296954	0.320709	4.044016	5.25E-05	0.000448	TRUE	UP
SON	9349.528	1.295321	0.340243	3.807049	0.000141	0.001025	TRUE	UP
SMC5	1774.074	1.294774	0.406788	3.182922	0.001458	0.007223	TRUE	UP
NAT6	540.049	1.29321	0.395406	3.270587	0.001073	0.005657	TRUE	UP
MOSPD2	527.7454	1.287173	0.401472	3.206137	0.001345	0.006783	TRUE	UP
LRRFIP2	2508.984	1.286869	0.342589	3.7563	0.000172	0.001219	TRUE	UP
GTF2H3	389.8286	1.285013	0.372597	3.448801	0.000563	0.00331	TRUE	UP
RBX1	2407.242	1.284865	0.333147	3.856751	0.000115	0.000866	TRUE	UP
VAPB	2085.102	1.279728	0.302909	4.224789	2.39E-05	0.000233	TRUE	UP
DCUN1D1	811.8543	1.278283	0.313998	4.07099	4.68E-05	0.000407	TRUE	UP
TRNT1	615.6786	1.275593	0.285667	4.465312	8.00E-06	8.95E-05	TRUE	UP
AP1AR	747.5988	1.275004	0.323443	3.941978	8.08E-05	0.000644	TRUE	UP
PPP2R3C	1116.035	1.2746	0.338902	3.760966	0.000169	0.001203	TRUE	UP
SPATA5	424.5067	1.270347	0.302387	4.201061	2.66E-05	0.000255	TRUE	UP
PHAX	995.3246	1.269471	0.239397	5.302793	1.14E-07	2.20E-06	TRUE	UP
SGPP1	1161.041	1.268434	0.368041	3.446444	0.000568	0.003333	TRUE	UP
SART1	4304.391	1.266304	0.313675	4.037001	5.41E-05	0.00046	TRUE	UP
MED30	491.5029	1.263321	0.388617	3.250813	0.001151	0.005973	TRUE	UP
NUDT6	197.7435	1.2628	0.371744	3.396962	0.000681	0.003874	TRUE	UP
DNAJC18	646.6209	1.262697	0.372327	3.391365	0.000695	0.003937	TRUE	UP
PITPNA	3727.167	1.261808	0.315443	4.000117	6.33E-05	0.000527	TRUE	UP
BCAS2	1387.275	1.26082	0.250317	5.03689	4.73E-07	7.70E-06	TRUE	UP
OTUD7B	1767.655	1.260122	0.378791	3.326693	0.000879	0.004807	TRUE	UP
MNAT1	442.1351	1.25799	0.335513	3.74945	0.000177	0.001248	TRUE	UP
CSNK1G2	3329.08	1.256439	0.298185	4.21362	2.51E-05	0.000243	TRUE	UP
RNF25	1281.153	1.253469	0.32768	3.825286	0.000131	0.000963	TRUE	UP
COQ2	646.2806	1.249392	0.356616	3.503466	0.000459	0.002772	TRUE	UP
TMEM110	445.9074	1.248889	0.318217	3.924651	8.69E-05	0.000684	TRUE	UP
PTP4A2	4909.677	1.243198	0.276277	4.499831	6.80E-06	7.86E-05	TRUE	UP
EIF2S2	3336.177	1.240048	0.275408	4.502583	6.71E-06	7.78E-05	TRUE	UP
TBCE	651.2967	1.239744	0.312258	3.970257	7.18E-05	0.000586	TRUE	UP

RBMXL1	1460.291	1.238321	0.286136	4.32774	1.51E-05	0.000156	TRUE	UP
FAM76A	610.5299	1.237911	0.294884	4.197953	2.69E-05	0.000257	TRUE	UP
ABCE1	2264.426	1.236801	0.261307	4.733141	2.21E-06	2.94E-05	TRUE	UP
CTTNBP2NL	1716.049	1.235557	0.278208	4.441128	8.95E-06	9.88E-05	TRUE	UP
VARS	3684.399	1.234161	0.346593	3.560837	0.00037	0.002301	TRUE	UP
RFX7	1015.585	1.233915	0.367498	3.357609	0.000786	0.004361	TRUE	UP
GAS8	604.2546	1.233095	0.39643	3.110495	0.001868	0.008908	TRUE	UP
TRAF6	334.682	1.231826	0.321252	3.834457	0.000126	0.000932	TRUE	UP
RBM4B	682.9879	1.229585	0.290881	4.227115	2.37E-05	0.000231	TRUE	UP
NMT2	1218.825	1.227487	0.373423	3.287126	0.001012	0.005386	TRUE	UP
ZMYM6	902.0699	1.22722	0.263056	4.665238	3.08E-06	3.97E-05	TRUE	UP
H3F3A	8064.885	1.225531	0.310976	3.940917	8.12E-05	0.000646	TRUE	UP
ZZZ3	1481.395	1.223844	0.317632	3.853022	0.000117	0.000876	TRUE	UP
ZNRD1	790.9199	1.222873	0.354998	3.444727	0.000572	0.003353	TRUE	UP
TAF3	390.1134	1.221043	0.334814	3.646926	0.000265	0.001759	TRUE	UP
SMARCAL1	1437.543	1.218072	0.317255	3.839412	0.000123	0.000916	TRUE	UP
CD3EAP	875.0233	1.217345	0.393656	3.092411	0.001985	0.009379	TRUE	UP
CLK4	1057.936	1.216784	0.359384	3.385747	0.00071	0.004001	TRUE	UP
NOM1	895.5187	1.216423	0.293343	4.146759	3.37E-05	0.000308	TRUE	UP
PINX1	276.4403	1.214269	0.369011	3.290603	0.001	0.005338	TRUE	UP
PTPN23	2975.235	1.211487	0.239908	5.0498	4.42E-07	7.27E-06	TRUE	UP
MRPS30	761.4035	1.207277	0.284494	4.243599	2.20E-05	0.000217	TRUE	UP
TFAP4	575.0371	1.205425	0.354337	3.401914	0.000669	0.003813	TRUE	UP
TMEM126A	557.4969	1.203105	0.356748	3.372425	0.000745	0.00417	TRUE	UP
SAR1B	1041.739	1.197675	0.376543	3.180708	0.001469	0.007257	TRUE	UP
MPHOSPH6	517.1929	1.197109	0.34756	3.444323	0.000572	0.003354	TRUE	UP
CWC15	1762.602	1.19654	0.274659	4.356464	1.32E-05	0.000139	TRUE	UP
PRMT3	506.6724	1.193476	0.36848	3.238917	0.0012	0.006185	TRUE	UP
FAM114A2	1078.881	1.19131	0.231058	5.155882	2.52E-07	4.41E-06	TRUE	UP
ZADH2	1301.434	1.180631	0.371022	3.182108	0.001462	0.007238	TRUE	UP
GSTCD	348.8756	1.179854	0.358274	3.293162	0.000991	0.005307	TRUE	UP
PNPLA8	1684.368	1.178714	0.334908	3.519519	0.000432	0.002625	TRUE	UP
KRIT1	1368.256	1.177431	0.325921	3.612627	0.000303	0.001959	TRUE	UP
ELP2	2040.649	1.172735	0.26403	4.44167	8.93E-06	9.86E-05	TRUE	UP
ZCCHC4	352.5411	1.169933	0.286	4.090671	4.30E-05	0.000378	TRUE	UP
CLK2	1728.562	1.168635	0.324167	3.605037	0.000312	0.002004	TRUE	UP
MAGOHB	597.72	1.168374	0.36757	3.178643	0.00148	0.007301	TRUE	UP
USP4	2903.685	1.165587	0.233824	4.984886	6.20E-07	9.76E-06	TRUE	UP
ZC3H15	2195.74	1.164106	0.231388	5.030962	4.88E-07	7.93E-06	TRUE	UP
IFT80	1247.868	1.162516	0.344875	3.370837	0.000749	0.004186	TRUE	UP
NAF1	311.6446	1.161788	0.329361	3.527406	0.00042	0.002558	TRUE	UP
SEH1L	1422.41	1.156909	0.276455	4.184797	2.85E-05	0.000269	TRUE	UP
NTAN1	1837.061	1.152285	0.317085	3.633994	0.000279	0.001832	TRUE	UP
FAM76B	552.4504	1.151882	0.348565	3.304641	0.000951	0.005128	TRUE	UP
FAIM	373.499	1.147206	0.37154	3.087708	0.002017	0.009506	TRUE	UP
SBNO2	3550.038	1.147054	0.336385	3.409941	0.00065	0.003723	TRUE	UP
DAP3	3476.131	1.142751	0.28242	4.046289	5.20E-05	0.000445	TRUE	UP
MRPS14	837.7883	1.140356	0.224508	5.079344	3.79E-07	6.34E-06	TRUE	UP

PDSS2	791.0087	1.139437	0.345701	3.296021	0.000981	0.005271	TRUE	UP
ARL6	124.1648	1.138148	0.323379	3.519544	0.000432	0.002625	TRUE	UP
MINPP1	724.8032	1.135115	0.352387	3.221217	0.001276	0.0065	TRUE	UP
TRA2A	2511.253	1.134996	0.229226	4.951437	7.37E-07	1.13E-05	TRUE	UP
MON1B	888.9318	1.134342	0.271243	4.182015	2.89E-05	0.000271	TRUE	UP
MTX2	1023.251	1.133027	0.323649	3.50079	0.000464	0.002794	TRUE	UP
SAMM50	2149.069	1.132527	0.262744	4.310375	1.63E-05	0.000167	TRUE	UP
TLK2	1093.377	1.131322	0.263371	4.295539	1.74E-05	0.000177	TRUE	UP
ATP5H	4649.567	1.129906	0.340938	3.31411	0.000919	0.00499	TRUE	UP
MTFR1	952.7764	1.128742	0.317689	3.552982	0.000381	0.002361	TRUE	UP
PAPD5	706.0095	1.126365	0.31481	3.577926	0.000346	0.00218	TRUE	UP
LRWD1	935.4082	1.12298	0.31754	3.536501	0.000405	0.00249	TRUE	UP
MFSD11	937.7299	1.120605	0.335512	3.339989	0.000838	0.004613	TRUE	UP
PPFIA1	1956.767	1.118022	0.313607	3.565036	0.000364	0.002268	TRUE	UP
TXNL1	2483.684	1.117646	0.279665	3.996374	6.43E-05	0.000534	TRUE	UP
PFDN2	2163.151	1.116656	0.331148	3.372072	0.000746	0.004172	TRUE	UP
DYNLL2	2620.607	1.116444	0.346889	3.218453	0.001289	0.006546	TRUE	UP
ALKBH3	862.7469	1.114801	0.32575	3.422263	0.000621	0.003583	TRUE	UP
LEMD2	2460.824	1.110038	0.327792	3.386407	0.000708	0.003994	TRUE	UP
MBD2	2440.818	1.108709	0.307199	3.609095	0.000307	0.00198	TRUE	UP
SLC10A7	349.2792	1.108282	0.355147	3.12063	0.001805	0.008646	TRUE	UP
COG8	857.1467	1.106615	0.308825	3.583307	0.000339	0.002144	TRUE	UP
PHF5A	1055.871	1.105344	0.264061	4.185944	2.84E-05	0.000268	TRUE	UP
GNL2	2843.28	1.103443	0.300115	3.676736	0.000236	0.001598	TRUE	UP
MRPS18C	647.7841	1.103209	0.315717	3.4943	0.000475	0.002851	TRUE	UP
NLE1	607.7628	1.101722	0.290784	3.788792	0.000151	0.001096	TRUE	UP
SSB	3948.996	1.100285	0.227784	4.830387	1.36E-06	1.96E-05	TRUE	UP
TMF1	2260.997	1.100151	0.319853	3.439558	0.000583	0.0034	TRUE	UP
RASA1	1729.964	1.098088	0.339247	3.236837	0.001209	0.006219	TRUE	UP
PDCD10	1022.977	1.098078	0.256587	4.279557	1.87E-05	0.000188	TRUE	UP
STK11	3166.707	1.097554	0.285312	3.846854	0.00012	0.000894	TRUE	UP
CMC1	331.721	1.096955	0.344209	3.186886	0.001438	0.007139	TRUE	UP
PHRF1	2358.411	1.096831	0.293844	3.732696	0.000189	0.001324	TRUE	UP
TAF6L	695.4056	1.095075	0.298284	3.671249	0.000241	0.001625	TRUE	UP
CSNK2A2	1142.947	1.093863	0.311634	3.510092	0.000448	0.00271	TRUE	UP
LARP4	1615.218	1.091351	0.32644	3.343196	0.000828	0.004567	TRUE	UP
SARS	6830.781	1.088761	0.294767	3.693639	0.000221	0.001508	TRUE	UP
KCMF1	2404.674	1.086433	0.224554	4.838174	1.31E-06	1.89E-05	TRUE	UP
NDUFV3	1706.009	1.086357	0.271621	3.999533	6.35E-05	0.000528	TRUE	UP
GIN1	272.7304	1.085312	0.337823	3.212666	0.001315	0.006655	TRUE	UP
HSPA4	3913.013	1.085149	0.287201	3.778356	0.000158	0.001135	TRUE	UP
MRPS33	1083.801	1.081448	0.31361	3.44838	0.000564	0.003313	TRUE	UP
RIOK3	2749.109	1.080666	0.310033	3.485648	0.000491	0.002932	TRUE	UP
TRIM39	830.8157	1.080132	0.287301	3.759587	0.00017	0.001208	TRUE	UP
CLP1	483.288	1.077008	0.304507	3.536895	0.000405	0.002487	TRUE	UP
HARBI1	136.5041	1.076467	0.338147	3.183434	0.001455	0.007212	TRUE	UP
CGRRF1	472.2541	1.075964	0.29245	3.679138	0.000234	0.001585	TRUE	UP
SEC22B	2718.331	1.074436	0.278021	3.864589	0.000111	0.000845	TRUE	UP

TAF5	246.4049	1.07378	0.28408	3.779848	0.000157	0.001129	TRUE	UP
DYNC1I2	2250.072	1.069242	0.259479	4.120732	3.78E-05	0.000339	TRUE	UP
FXR2	2044.842	1.067013	0.337088	3.165385	0.001549	0.007591	TRUE	UP
TCF25	4868.159	1.065572	0.283532	3.758207	0.000171	0.001212	TRUE	UP
CEPT1	1248.932	1.064938	0.271348	3.924618	8.69E-05	0.000684	TRUE	UP
RBM26	1116.457	1.063099	0.316409	3.359895	0.00078	0.004331	TRUE	UP
CSNK2A1	2082.823	1.061541	0.286521	3.704927	0.000211	0.00145	TRUE	UP
MMS19	2095.58	1.059045	0.31453	3.367072	0.00076	0.004237	TRUE	UP
RIOK1	965.7192	1.058166	0.338008	3.130597	0.001745	0.008387	TRUE	UP
VCPIP1	1201.479	1.058046	0.310844	3.40379	0.000665	0.003795	TRUE	UP
TSEN2	337.7863	1.056396	0.3278	3.222686	0.00127	0.006474	TRUE	UP
PDRG1	1203.519	1.053455	0.333688	3.157008	0.001594	0.007782	TRUE	UP
DTNBP1	933.2519	1.051879	0.338573	3.106802	0.001891	0.009008	TRUE	UP
SUMO2	6063.366	1.051713	0.255621	4.114347	3.88E-05	0.000347	TRUE	UP
MTHFSD	714.6617	1.049381	0.282966	3.708504	0.000208	0.001434	TRUE	UP
COPG2	707.1297	1.048076	0.289985	3.614238	0.000301	0.001951	TRUE	UP
ESD	3815.612	1.047092	0.316023	3.313342	0.000922	0.004996	TRUE	UP
DDX52	851.3269	1.045325	0.259269	4.031814	5.53E-05	0.00047	TRUE	UP
RBM25	2855.877	1.039129	0.287983	3.608299	0.000308	0.001984	TRUE	UP
EED	696.4857	1.030944	0.266175	3.873178	0.000107	0.000821	TRUE	UP
ZBTB6	642.3937	1.029448	0.280815	3.665928	0.000246	0.001654	TRUE	UP
SRA1	1835.279	1.028552	0.331829	3.099646	0.001938	0.0092	TRUE	UP
ACOX3	773.7026	1.026365	0.332584	3.086034	0.002028	0.009549	TRUE	UP
TIAL1	2470.206	1.025823	0.262771	3.90387	9.47E-05	0.000735	TRUE	UP
ZC3H14	1954.841	1.025347	0.296131	3.462481	0.000535	0.003167	TRUE	UP
ILKAP	1254.266	1.024001	0.319162	3.208407	0.001335	0.006737	TRUE	UP
HNRNPF	7648.703	1.021988	0.272021	3.757015	0.000172	0.001216	TRUE	UP
MYPOP	424.2835	1.020755	0.300925	3.392054	0.000694	0.00393	TRUE	UP
ZCCHC8	980.7755	1.019309	0.294155	3.465204	0.00053	0.003139	TRUE	UP
CLK3	2076.495	1.018749	0.269047	3.786511	0.000153	0.001104	TRUE	UP
RUFY2	620.2598	1.017803	0.306752	3.317997	0.000907	0.004931	TRUE	UP
RABIF	598.0996	1.016698	0.295666	3.438673	0.000585	0.003407	TRUE	UP
DNAJC19	721.6138	1.015137	0.318231	3.189942	0.001423	0.007093	TRUE	UP
UBE2I	4164.337	1.014338	0.222615	4.556475	5.20E-06	6.23E-05	TRUE	UP
INTS12	767.4226	1.01349	0.21372	4.742145	2.11E-06	2.83E-05	TRUE	UP
DHX16	1864.906	1.012806	0.236955	4.274252	1.92E-05	0.000192	TRUE	UP
HMGB1	9963.739	1.010926	0.258414	3.912035	9.15E-05	0.000715	TRUE	UP
CC2D1B	1747.598	1.010885	0.271983	3.716726	0.000202	0.001393	TRUE	UP
WDR70	1279.971	1.009818	0.281873	3.582525	0.00034	0.002148	TRUE	UP
CWC25	740.5465	1.001774	0.238588	4.19876	2.68E-05	0.000257	TRUE	UP

gene_symb	delabel
MYH4	MYH4
RPL21	RPL21
CD300LD	CD300LD
S100A7A	S100A7A
GGNBP1	GGNBP1
GPR149	GPR149
GFRA4	GFRA4
CELA1	CELA1
GNG8	GNG8
CACNG2	CACNG2
ZAN	ZAN
TRIML1	TRIML1
CEACAM20	CEACAM20
RAG2	RAG2
GBP7	GBP7
RTP3	RTP3
BRDT	BRDT
PRAMEF8	PRAMEF8
EXD1	EXD1
MYL1	MYL1
GRK1	GRK1
PF4	PF4
PDE6H	PDE6H
ART1	ART1
ACTN3	ACTN3
MYLPF	MYLPF
MIA2	MIA2
LARS2	LARS2
LRTM1	LRTM1
STARD6	STARD6
FAM58B	FAM58B
CKM	CKM
CST6	CST6
CR1L	CR1L
TMEM88B	TMEM88B
BOLL	BOLL
KLHL33	KLHL33
PKD2L2	PKD2L2
EPB42	EPB42
GPR45	GPR45
ZBTB20	ZBTB20
LOR	LOR
CRX	CRX
HIST1H4B	HIST1H4B
TSHB	TSHB
PSMB11	PSMB11
AWAT2	AWAT2

PLEKHN1	PLEKHN1
HIST1H4F	HIST1H4F
RPL3L	RPL3L
PGLYRP1	PGLYRP1
DUPD1	DUPD1
MYH8	MYH8
CPSF4L	CPSF4L
GNAT1	GNAT1
COX6A2	COX6A2
GCSH	GCSH
PROX2	PROX2
LGALS7	LGALS7
CCR9	CCR9
IL4	IL4
PCDHGC4	PCDHGC4
PRSS42	PRSS42
CCL7	CCL7
CRYBB3	CRYBB3
CRLF2	CRLF2
CHRNA2	CHRNA2
ACPP	ACPP
HIST1H3I	HIST1H3I
NEUROD2	NEUROD2
RPL36A	RPL36A
ARR3	ARR3
PLSCR2	PLSCR2
BTNL2	BTNL2
UPP2	UPP2
SPRED3	SPRED3
ARL5C	ARL5C
SPEF1	SPEF1
GBP6	GBP6
RGS17	RGS17
ATP2A1	ATP2A1
TNNC2	TNNC2
AMPD1	AMPD1
CACNA1S	CACNA1S
TMOD4	TMOD4
SMN1	SMN1
HIST1H2BB	HIST1H2BB
RGS8	RGS8
MYH1	MYH1
HAL	HAL
HIST1H1D	HIST1H1D
NUP210L	NUP210L
CALR3	CALR3
TIFAB	TIFAB
FGF21	FGF21

KCNA7	KCNA7
CNGB1	CNGB1
DCST1	DCST1
APOC4	APOC4
RPS27	RPS27
DPPA3	DPPA3
GCNT7	GCNT7
NEURL3	NEURL3
PVALB	PVALB
P2RY4	P2RY4
DKK2	DKK2
KCNH7	KCNH7
IL1RL1	IL1RL1
SLC39A2	SLC39A2
GJB3	GJB3
PRKAG3	PRKAG3
GAPDHS	GAPDHS
XIRP2	XIRP2
DCDC2B	DCDC2B
PIRT	PIRT
HIST1H2AC	HIST1H2AD
TCAP	TCAP
TNNI2	TNNI2
CCDC27	CCDC27
ACTL6B	ACTL6B
SH2D5	SH2D5
TEKT2	TEKT2
MYBPC2	MYBPC2
DQX1	DQX1
DMP1	DMP1
OPTC	OPTC
HFE2	HFE2
FGF22	FGF22
KLHL32	KLHL32
CRYAA	CRYAA
FBXO47	FBXO47
PGLYRP2	PGLYRP2
SPATA1	SPATA1
NIPAL1	NIPAL1
RNF17	RNF17
DCT	DCT
WNT8B	WNT8B
LMOD2	LMOD2
CACNG8	CACNG8
ASAH2	ASAH2
HIST1H4I	HIST1H4I
CTXN3	CTXN3
GPR152	GPR152

ALOXE3	ALOXE3
IL1F9	IL1F9
KLHL31	KLHL31
DDI2	DDI2
CCL25	CCL25
RPGRIP1	RPGRIP1
ALG11	ALG11
GJB4	GJB4
EREG	EREG
KCNA2	KCNA2
MYT1L	MYT1L
DDX4	DDX4
PPEF2	PPEF2
COL20A1	COL20A1
TMEM212	TMEM212
GRIP2	GRIP2
LCT	LCT
CACNG1	CACNG1
UOX	UOX
CLEC4D	CLEC4D
MYOT	MYOT
HIST1H4A	HIST1H4A
CCR5	CCR5
HRK	HRK
GLTPD2	GLTPD2
CNR2	CNR2
CREG2	CREG2
SERPINC1	SERPINC1
ZDHHC20	ZDHHC20
CCDC114	CCDC114
TREML4	TREML4
CASP12	CASP12
ANKRD23	ANKRD23
SVOPL	SVOPL
HIST1H1B	HIST1H1B
ACOT6	ACOT6
FNDC7	FNDC7
12-Sep	12-Sep
TRIML2	TRIML2
UPB1	UPB1
GAN	GAN
ZBTB37	ZBTB37
PHACTR1	PHACTR1
PATE2	PATE2
CDKL4	CDKL4
ACADL	ACADL
DENND1B	DENND1B
DHDH	DHDH

IDO2	IDO2
RASAL1	RASAL1
TNFRSF9	TNFRSF9
GRM5	GRM5
LY6G6C	LY6G6C
NANOG	NANOG
CAPNS2	CAPNS2
RFX3	RFX3
SLC4A9	SLC4A9
PLD4	PLD4
HIST1H3C	HIST1H3C
OMP	OMP
NR6A1	NR6A1
EML5	EML5
ZSCAN10	ZSCAN10
PRDM11	PRDM11
GJC3	GJC3
SOAT2	SOAT2
PRKCG	PRKCG
SLC26A8	SLC26A8
GRHL2	GRHL2
GNAT2	GNAT2
FRRS1	FRRS1
CCDC62	CCDC62
C1QL3	C1QL3
MYOZ1	MYOZ1
CD274	CD274
MYO1H	MYO1H
LENEP	LENEP
XKR6	XKR6
HIST1H2BC	HIST1H2BC
PDZD9	PDZD9
IPMK	IPMK
HIST1H4C	HIST1H4C
TRPM6	TRPM6
TTC9B	TTC9B
CELF4	CELF4
CHST8	CHST8
MEGF11	MEGF11
TPTE	TPTE
NEB	NEB
STMN4	STMN4
POU2F2	POU2F2
VWA3A	VWA3A
GDF3	GDF3
A4GNT	A4GNT
YIPF7	YIPF7
ARHGDIG	ARHGDIG

RPL7	RPL7
PRRG2	PRRG2
GRIN1	GRIN1
CFP	CFP
HIST1H2AB	HIST1H2AB
MPP6	MPP6
NCR1	NCR1
STYK1	STYK1
AGBL3	AGBL3
MB	MB
SLC16A6	SLC16A6
BRI3BP	BRI3BP
IL9R	IL9R
HIST1H2AH	HIST1H2AH
FAM19A1	FAM19A1
GNGT2	GNGT2
PLA2G7	PLA2G7
ACSL6	ACSL6
SLC30A4	SLC30A4
DNAJB13	DNAJB13
BTBD18	BTBD18
KCNK10	KCNK10
MYLK4	MYLK4
DGKH	DGKH
NRG4	NRG4
NCCRP1	NCCRP1
MEP1A	MEP1A
CCR2	CCR2
TPH1	TPH1
ALOX12	ALOX12
AGBL1	AGBL1
GALR2	GALR2
UNC45B	UNC45B
RNASE10	RNASE10
BTBD16	BTBD16
MBOAT4	MBOAT4
EME2	EME2
ZBP1	ZBP1
SNX32	SNX32
RPL18A	RPL18A
SLC25A2	SLC25A2
NEDD4	NEDD4
MED12L	MED12L
MSH4	MSH4
GRIK4	GRIK4
RPL39	RPL39
CX3CR1	CX3CR1
ATG9B	ATG9B

CUX2	CUX2
NAPSA	NAPSA
NAT2	NAT2
RRH	RRH
MS4A14	MS4A14
TXK	TXK
HSF5	HSF5
HAVCR1	HAVCR1
ENO3	ENO3
IGSF10	IGSF10
WNT9A	WNT9A
MBNL3	MBNL3
PFKFB1	PFKFB1
SASS6	SASS6
RPS28	RPS28
IL18RAP	IL18RAP
MYADML2	MYADML2
RHO	RHO
SOCS7	SOCS7
SYNGR4	SYNGR4
TTN	TTN
ART3	ART3
SLC12A5	SLC12A5
FAM71F1	FAM71F1
SLC35E2.1	SLC35E2.1
ENKUR	ENKUR
CD3G	CD3G
SLC38A6	SLC38A6
LMTK3	LMTK3
CRHR2	CRHR2
ITGA2B	ITGA2B
ELK4	ELK4
CLEC12A	CLEC12A
DOC2B	DOC2B
PTX4	PTX4
LPAR4	LPAR4
PROZ	PROZ
WDR66	WDR66
GPT	GPT
RCAN3	RCAN3
RAB27B	RAB27B
RNF183	RNF183
BTBD8	BTBD8
ACRBP	ACRBP
TTYH1	TTYH1
IL1RL2	IL1RL2
SLC23A1	SLC23A1
CXCR2	CXCR2

CACNA2D1	CACNA2D1
SFT2D2	SFT2D2
ANXA3	ANXA3
CCNT1	CCNT1
CDHR4	CDHR4
ANAPC4	ANAPC4
UCK2	UCK2
PRDM12	PRDM12
FAM53A	FAM53A
PKD1L3	PKD1L3
GPR182	GPR182
CACNA1F	CACNA1F
STOX2	STOX2
EFCAB3	EFCAB3
ZKSCAN3	ZKSCAN3
INSL3	INSL3
RASL10A	RASL10A
HIST4H4	HIST4H4
RPL9	RPL9
PSORS1C2	PSORS1C2
RPS26	RPS26
SLC5A11	SLC5A11
HES7	HES7
PHOSPHO2	PHOSPHO2
RABGAP1L	RABGAP1L
KCNRG	KCNRG
SYCP3	SYCP3
NMBR	NMBR
IMMP1L	IMMP1L
KCNH4	KCNH4
KCNN4	KCNN4
KLC3	KLC3
RBMS3	RBMS3
STAP1	STAP1
PYGO1	PYGO1
ERN1	ERN1
CBX6	CBX6
GPR157	GPR157
GABPB2	GABPB2
GPRC5D	GPRC5D
NUDT14	NUDT14
FBXO15	FBXO15
P2RY2	P2RY2
NFATC2	NFATC2
MYO1A	MYO1A
SLC9A5	SLC9A5
HAUS8	HAUS8
RGS20	RGS20

GXYLT2	GXYLT2
PPP1R14B	PPP1R14B
NBEAL1	NBEAL1
FCGR2B	FCGR2B
NPFF	NPFF
CSF2RA	CSF2RA
IMPG2	IMPG2
CARD9	CARD9
RDM1	RDM1
USP49	USP49
PIK3CA	PIK3CA
PPM1L	PPM1L
ABCA5	ABCA5
CCDC65	CCDC65
ZC3H12D	ZC3H12D
PRIM1	PRIM1
ANKRD44	ANKRD44
CCDC157	CCDC157
P2RY12	P2RY12
REL	REL
FMN1	FMN1
ABHD12B	ABHD12B
IL27	IL27
GVIN1	GVIN1
CDC26	CDC26
CD300LB	CD300LB
GRAMD1B	GRAMD1B
MSI2	MSI2
SESN3	SESN3
GTF2A1	GTF2A1
HOMER1	HOMER1
TLE6	TLE6
IRAK1BP1	IRAK1BP1
CD38	CD38
PDGFA	PDGFA
FITM1	FITM1
MPZL3	MPZL3
CD84	CD84
PTPN14	PTPN14
DLEC1	DLEC1
HSPA4L	HSPA4L
BRAF	BRAF
GATC	GATC
RPL17	RPL17
TNFRSF11A	TNFRSF11A
PCDHGC5	PCDHGC5
PAFAH1B2	PAFAH1B2
GNG3	GNG3

LRRC39	LRRC39
GLRX2	GLRX2
FABP5	FABP5
FAM71F2	FAM71F2
SLC9A7	SLC9A7
GZMB	GZMB
SMTNL1	SMTNL1
SIRT4	SIRT4
SMYD2	SMYD2
ZSCAN5B	ZSCAN5B
TBX15	TBX15
ARHGAP22	ARHGAP22
DNASE1L2	DNASE1L2
UGCG	UGCG
CENPI	CENPI
CHD7	CHD7
AGBL2	AGBL2
RPL23A	RPL23A
VWA5B2	VWA5B2
TMEM38A	TMEM38A
GJB5	GJB5
HELB	HELB
SNX20	SNX20
TNF	TNF
NCF1	NCF1
LOXL3	LOXL3
TSPAN5	TSPAN5
LEFTY1	LEFTY1
B4GALT6	B4GALT6
PPCDC	PPCDC
HIST3H2A	HIST3H2A
ENPP3	ENPP3
GNRHR	GNRHR
INHA	INHA
FLVCR1	FLVCR1
CHD9	CHD9
NOXO1	NOXO1
FAM129C	FAM129C
PPP4R2	PPP4R2
GPR19	GPR19
GFOD1	GFOD1
CCDC148	CCDC148
RPSA	RPSA
UHMK1	UHMK1
TOR1AIP2	TOR1AIP2
HAGHL	HAGHL
CCDC134	CCDC134
TEX9	TEX9

CDNF	CDNF
ZMYND15	ZMYND15
SAMD8	SAMD8
HMBOX1	HMBOX1
ALS2CR12	ALS2CR12
TTC39B	TTC39B
CASC1	CASC1
EIF3C	EIF3C
HJURP	HJURP
RAPGEF4	RAPGEF4
NEK10	NEK10
CCDC36	CCDC36
LY86	LY86
RNF217	RNF217
PSTK	PSTK
RHBDL2	RHBDL2
UBE2S	UBE2S
ITPRIPL1	ITPRIPL1
CENPA	CENPA
XPO4	XPO4
TAOK1	TAOK1
PTPN22	PTPN22
ADAM32	ADAM32
KCNK13	KCNK13
LEKR1	LEKR1
NUDT13	NUDT13
GPR35	GPR35
NDRG4	NDRG4
BICD1	BICD1
FBF1	FBF1
DOK3	DOK3
PIGN	PIGN
PIAS2	PIAS2
SLC25A4	SLC25A4
NHLRC2	NHLRC2
PPAN	PPAN
SERHL	SERHL
CAMK1D	CAMK1D
BMP2	BMP2
GINS4	GINS4
PHOSPHO1	PHOSPHO1
MYSM1	MYSM1
TGM1	TGM1
P2RX7	P2RX7
NIPSNAP3E	NIPSNAP3B
RSPH9	RSPH9
CDYL2	CDYL2
FAM133B	FAM133B

TIGD4	TIGD4
RIPK3	RIPK3
FAM173A	FAM173A
GDPD1	GDPD1
OSCP1	OSCP1
MAPK6	MAPK6
HFE	HFE
FBXL20	FBXL20
CD300LF	CD300LF
KLF7	KLF7
NOL12	NOL12
ETV3	ETV3
ACSBG1	ACSBG1
CHKB	CHKB
SAMD1	SAMD1
ATXN7L2	ATXN7L2
RHD	RHD
UBN2	UBN2
08-Mar	08-Mar
MORN1	MORN1
EIF4E	EIF4E
LARP4B	LARP4B
LRRIQ3	LRRIQ3
IL3RA	IL3RA
ADAM8	ADAM8
E2F5	E2F5
P2RY6	P2RY6
RRP1	RRP1
RAD52	RAD52
E2F8	E2F8
ZDHHC21	ZDHHC21
PAIP2B	PAIP2B
CAPN11	CAPN11
LCOR	LCOR
NACC2	NACC2
ADK	ADK
CCHCR1	CCHCR1
SAMD14	SAMD14
LIPT2	LIPT2
ZBED3	ZBED3
TAF13	TAF13
CALHM1	CALHM1
DAPP1	DAPP1
WWTR1	WWTR1
DNAJB14	DNAJB14
RNFT2	RNFT2
LMBRD2	LMBRD2
SPC24	SPC24

EXOSC8	EXOSC8
TIGD3	TIGD3
RPS7	RPS7
SBNO1	SBNO1
BAIAP2	BAIAP2
LETM2	LETM2
CENPC1	CENPC1
COMTD1	COMTD1
TMPRSS5	TMPRSS5
SYCE2	SYCE2
TIPIN	TIPIN
ULK4	ULK4
RPL22L1	RPL22L1
LRRC45	LRRC45
EYA3	EYA3
CDK8	CDK8
RASA2	RASA2
CCDC138	CCDC138
TMEM160	TMEM160
DUSP11	DUSP11
FBXW8	FBXW8
SKIL	SKIL
ACE	ACE
GHR	GHR
SLC7A6OS	SLC7A6OS
SPATA24	SPATA24
ACER2	ACER2
UNC93B1	UNC93B1
CARS	CARS
REST	REST
SMPD3	SMPD3
ARNTL2	ARNTL2
FSD1L	FSD1L
SNRNP48	SNRNP48
AHSA2	AHSA2
NETO2	NETO2
PPIA	PPIA
MTBP	MTBP
RAD9B	RAD9B
IL34	IL34
DENND2C	DENND2C
AOC2	AOC2
PARP8	PARP8
USE1	USE1
ANKLE1	ANKLE1
IER5L	IER5L
CTH	CTH
FITM2	FITM2

ALG10B	ALG10B
ADCK5	ADCK5
POU2F1	POU2F1
CCDC153	CCDC153
ASB14	ASB14
RBM15	RBM15
CLOCK	CLOCK
EXOC6B	EXOC6B
RAB43	RAB43
RGP1	RGP1
PDZD7	PDZD7
CORO2A	CORO2A
TM6SF1	TM6SF1
CTSS	CTSS
AFG3L1	AFG3L1
ADCY7	ADCY7
ZC3HAV1L	ZC3HAV1L
SPATA6	SPATA6
CNOT10	CNOT10
ANKRD37	ANKRD37
RARG	RARG
RAD54L2	RAD54L2
CHIC2	CHIC2
REEP3	REEP3
RUNX1T1	RUNX1T1
ANKRD16	ANKRD16
ECSCR	ECSCR
SNHG11	SNHG11
RPS2	RPS2
STRN	STRN
RPL13A	RPL13A
AGAP1	AGAP1
PCSK4	PCSK4
ABHD11	ABHD11
USP12	USP12
MCM9	MCM9
NAA16	NAA16
RAB30	RAB30
IQCD	IQCD
UEVLD	UEVLD
SEC62	SEC62
NUFIP1	NUFIP1
OGFRL1	OGFRL1
TCP11L2	TCP11L2
RHEBL1	RHEBL1
LRRC46	LRRC46
ZMAT3	ZMAT3
CLCF1	CLCF1

DPP8	DPP8
DPEP2	DPEP2
DNAJC28	DNAJC28
ZRSR2	ZRSR2
GRK4	GRK4
BMP2K	BMP2K
PIWIL4	PIWIL4
TPK1	TPK1
TNRC6C	TNRC6C
CETN3	CETN3
PYROXD1	PYROXD1
HOXC9	HOXC9
RC3H2	RC3H2
RP9	RP9
SNX12	SNX12
PIK3CG	PIK3CG
SLC35A3	SLC35A3
PDP2	PDP2
FNDC8	FNDC8
ZWINT	ZWINT
CCDC84	CCDC84
GUCA1B	GUCA1B
PLEKHA8	PLEKHA8
FAM151B	FAM151B
CHIC1	CHIC1
PRDM5	PRDM5
SFT2D1	SFT2D1
ADCY4	ADCY4
TATDN1	TATDN1
LIMS1	LIMS1
PFDN6	PFDN6
THOC1	THOC1
PRELID1	PRELID1
PSMC1	PSMC1
IQCB1	IQCB1
MAST4	MAST4
BTBD19	BTBD19
SPCS2	SPCS2
NUDT4	NUDT4
GCLM	GCLM
ZRANB1	ZRANB1
TAF1D	TAF1D
ZNRF1	ZNRF1
METTL3	METTL3
PHKG2	PHKG2
TLR6	TLR6
MAN2A1	MAN2A1
SFI1	SFI1

KIF18A	KIF18A
TLCD1	TLCD1
HELLS	HELLS
SH2B2	SH2B2
FAM168A	FAM168A
CENPE	CENPE
ANO8	ANO8
ANAPC10	ANAPC10
TIGD2	TIGD2
PRIM2	PRIM2
TMPPE	TMPPE
MMAA	MMAA
SSR3	SSR3
ADAM17	ADAM17
NOP16	NOP16
ERMP1	ERMP1
NEK8	NEK8
FDXACB1	FDXACB1
NEIL1	NEIL1
ERO1L	ERO1L
RSRC1	RSRC1
SLC25A45	SLC25A45
GUF1	GUF1
GTF2F1	GTF2F1
SLC25A37	SLC25A37
ING1	ING1
EEF1E1	EEF1E1
KIN	KIN
DOK1	DOK1
TMEM106[illegible]	TMEM106A
RBPJ	RBPJ
USMG5	USMG5
EMG1	EMG1
PANK3	PANK3
CCDC34	CCDC34
NAA35	NAA35
PAOX	PAOX
NFXL1	NFXL1
ZBTB7A	ZBTB7A
TECPR1	TECPR1
SNAPC5	SNAPC5
ATL3	ATL3
SLC16A13	SLC16A13
CUEDC2	CUEDC2
TULP4	TULP4
IFNAR2	IFNAR2
ZFHX3	ZFHX3
ALKBH1	ALKBH1

EIF2AK2	EIF2AK2
ZBTB26	ZBTB26
PRKAR2A	PRKAR2A
NAA15	NAA15
NRL	NRL
CHKA	CHKA
WDFY2	WDFY2
GMEB1	GMEB1
RFXAP	RFXAP
MAPK8	MAPK8
ATE1	ATE1
SKP2	SKP2
PIGB	PIGB
PTGR2	PTGR2
PSMD12	PSMD12
SENP7	SENP7
TTC5	TTC5
LEPROT	LEPROT
GLRX3	GLRX3
RSBN1	RSBN1
ASPSCR1	ASPSCR1
RNF19B	RNF19B
PCYT2	PCYT2
AGER	AGER
APLF	APLF
LIAS	LIAS
IKBKE	IKBKE
SNRPA1	SNRPA1
ORAOV1	ORAOV1
TSPO	TSPO
C1GALT1	C1GALT1
MRPL33	MRPL33
WDR4	WDR4
DCUN1D5	DCUN1D5
NVL	NVL
MAD2L1	MAD2L1
FIP1L1	FIP1L1
ENTPD5	ENTPD5
AMMECR1	AMMECR1
TMED5	TMED5
TNRC6A	TNRC6A
AUH	AUH
SERINC3	SERINC3
BAK1	BAK1
SMPD2	SMPD2
ANAPC1	ANAPC1
ABCA7	ABCA7
NPM3	NPM3

RPS27A	RPS27A
XYLB	XYLB
DOCK5	DOCK5
LCORL	LCORL
FZD5	FZD5
CASP8	CASP8
MRPL13	MRPL13
TRIM3	TRIM3
PLK4	PLK4
DNLZ	DNLZ
TRAF3	TRAF3
MPND	MPND
MAGI3	MAGI3
P2RX4	P2RX4
CDK7	CDK7
ZBTB25	ZBTB25
TBC1D19	TBC1D19
TRIM59	TRIM59
UBA5	UBA5
FBXL6	FBXL6
AKAP8L	AKAP8L
XRCC4	XRCC4
NUDT9	NUDT9
RBM45	RBM45
LGALS1	LGALS1
TRMT2B	TRMT2B
PAK1IP1	PAK1IP1
CLPB	CLPB
RNF168	RNF168
INO80D	INO80D
PPID	PPID
TET3	TET3
NIP7	NIP7
AP1S1	AP1S1
MSTO1	MSTO1
SLC35E4	SLC35E4
FUT11	FUT11
TRMT1	TRMT1
SLC41A2	SLC41A2
ANAPC2	ANAPC2
CHRNB1	CHRNB1
HSPE1	HSPE1
TIRAP	TIRAP
C1D	C1D
ANKRD39	ANKRD39
PDCL3	PDCL3
FAM193B	FAM193B
CRK	CRK

PHTF1	PHTF1
GAR1	GAR1
POLB	POLB
DTYMK	DTYMK
POLR2F	POLR2F
MFN1	MFN1
HEATR3	HEATR3
COMMD8	COMMD8
RNF126	RNF126
SPAST	SPAST
PSMC6	PSMC6
GRAMD1C	GRAMD1C
SSBP3	SSBP3
PRELID2	PRELID2
TARBP2	TARBP2
RNPC3	RNPC3
EHMT2	EHMT2
AMDHD2	AMDHD2
PDCD2L	PDCD2L
SPSB2	SPSB2
RPS6KB2	RPS6KB2
PRPF39	PRPF39
DGKA	DGKA
TMCC1	TMCC1
PSMA3	PSMA3
SPDYA	SPDYA
ENY2	ENY2
MRPL48	MRPL48
TAF1A	TAF1A
TARS2	TARS2
RAB2A	RAB2A
CYHR1	CYHR1
PARP3	PARP3
PLA2G6	PLA2G6
RBM28	RBM28
CFLAR	CFLAR
MCOLN1	MCOLN1
RBM7	RBM7
PPIC	PPIC
FGFR1OP	FGFR1OP
SIRT7	SIRT7
CHEK2	CHEK2
SLC36A4	SLC36A4
PHF7	PHF7
SSH2	SSH2
CHORDC1	CHORDC1
COMMD1	COMMD1
PIAS1	PIAS1

LSM6	LSM6
CDADC1	CDADC1
METTL14	METTL14
ZNHIT2	ZNHIT2
FGFR1OP2	FGFR1OP2
IDE	IDE
RPL6	RPL6
RPL7L1	RPL7L1
MRPS17	MRPS17
MYBBP1A	MYBBP1A
LSM7	LSM7
DUSP18	DUSP18
PDSS1	PDSS1
MDP1	MDP1
UTP15	UTP15
HAUS3	HAUS3
HSD17B7	HSD17B7
ZCRB1	ZCRB1
WDR74	WDR74
TCIRG1	TCIRG1
AMN1	AMN1
DRAP1	DRAP1
ANKMY1	ANKMY1
NOL9	NOL9
U2AF1L4	U2AF1L4
DTNB	DTNB
POLR1C	POLR1C
IPO4	IPO4
FAM185A	FAM185A
DBF4	DBF4
CCDC9	CCDC9
RBMS1	RBMS1
CDKL3	CDKL3
PRPF38B	PRPF38B
E4F1	E4F1
SNRPG	SNRPG
TTC13	TTC13
AKR1A1	AKR1A1
CYB5R4	CYB5R4
SENP8	SENP8
SLC38A7	SLC38A7
PTMA	PTMA
EEF1B2	EEF1B2
RRAGC	RRAGC
NISCH	NISCH
UBE2R2	UBE2R2
FDX1L	FDX1L
SNRPE	SNRPE

MAFG	MAFG
FUT10	FUT10
CCAR1	CCAR1
STRBP	STRBP
ELMOD2	ELMOD2
IQCC	IQCC
MRPL24	MRPL24
FAM98B	FAM98B
TUBGCP4	TUBGCP4
TUT1	TUT1
CUEDC1	CUEDC1
ANKZF1	ANKZF1
MRPL52	MRPL52
SUGT1	SUGT1
FBXO4	FBXO4
GTPBP4	GTPBP4
TNKS2	TNKS2
SAAL1	SAAL1
ERGIC2	ERGIC2
UHRF1BP1	UHRF1BP1L
SRP19	SRP19
CRLF3	CRLF3
NEK3	NEK3
SIPA1L3	SIPA1L3
FUBP1	FUBP1
RANBP1	RANBP1
CCDC102A	CCDC102A
UBAP2	UBAP2
COX6A1	COX6A1
ZC3H7A	ZC3H7A
BBS12	BBS12
SPPL2A	SPPL2A
LDB1	LDB1
SCAI	SCAI
DOHH	DOHH
WDR43	WDR43
POLR3K	POLR3K
MAPKAPK5	MAPKAPK5
ZCCHC7	ZCCHC7
PAIP1	PAIP1
SNX24	SNX24
TET2	TET2
BHLHB9	BHLHB9
TSC22D2	TSC22D2
REXO2	REXO2
USPL1	USPL1
ATP5J2	ATP5J2
LYAR	LYAR

INVS	INVS
COX17	COX17
CTU1	CTU1
MED31	MED31
LCLAT1	LCLAT1
SNRPD1	SNRPD1
PRKACB	PRKACB
PRKCD	PRKCD
SNX5	SNX5
OTUD6B	OTUD6B
NFKB1	NFKB1
AVL9	AVL9
ZBTB8OS	ZBTB8OS
KIF3A	KIF3A
PAN3	PAN3
UBR2	UBR2
PDE7A	PDE7A
TRIM35	TRIM35
MARS2	MARS2
SETD6	SETD6
USP45	USP45
CDK5	CDK5
PPOX	PPOX
ZCWPW1	ZCWPW1
NOP14	NOP14
TXNDC17	TXNDC17
SLC25A17	SLC25A17
NUDCD1	NUDCD1
DCAF13	DCAF13
APBA3	APBA3
CDC16	CDC16
ZMYM5	ZMYM5
CSTF2	CSTF2
TBP	TBP
ACBD5	ACBD5
AEBP2	AEBP2
BCL10	BCL10
CDK11B	CDK11B
WBP4	WBP4
TDRD3	TDRD3
TMEM206	TMEM206
INO80C	INO80C
PIAS4	PIAS4
TFB2M	TFB2M
HSPA8	HSPA8
METTL6	METTL6
CASP6	CASP6
POLG2	POLG2

LSM12	LSM12
KLHDC4	KLHDC4
RCHY1	RCHY1
KCTD17	KCTD17
EIF4G3	EIF4G3
DENND4A	DENND4A
MAST3	MAST3
POP1	POP1
DIRC2	DIRC2
RHBDF1	RHBDF1
MED7	MED7
PIGM	PIGM
SEC61A2	SEC61A2
PCYT1A	PCYT1A
YOD1	YOD1
BOLA3	BOLA3
OTUD4	OTUD4
ATP6V0A2	ATP6V0A2
G3BP1	G3BP1
TXNDC9	TXNDC9
VDAC2	VDAC2
RSBN1L	RSBN1L
PTCD2	PTCD2
CCNL1	CCNL1
NOP58	NOP58
PUS10	PUS10
LRIG2	LRIG2
ARHGEF7	ARHGEF7
HEATR5A	HEATR5A
PGS1	PGS1
AP4M1	AP4M1
ALG5	ALG5
BNIP2	BNIP2
SSBP4	SSBP4
WDR12	WDR12
GEMIN7	GEMIN7
MRM1	MRM1
TNPO3	TNPO3
GTPBP10	GTPBP10
GRAMD4	GRAMD4
SOCS5	SOCS5
ATP5S	ATP5S
NUP88	NUP88
TMEM164	TMEM164
COX19	COX19
NSMCE2	NSMCE2
NOP2	NOP2
ATP6V0C	ATP6V0C

CORO1B	CORO1B
G2E3	G2E3
TTC14	TTC14
DGKZ	DGKZ
BANP	BANP
RAB3GAP2	RAB3GAP2
SDCCAG8	SDCCAG8
DHODH	DHODH
BPTF	BPTF
PGAP2	PGAP2
BRPF1	BRPF1
ALKBH8	ALKBH8
CTU2	CTU2
RNF214	RNF214
KIF2A	KIF2A
ING3	ING3
ALG14	ALG14
ATP9B	ATP9B
SON	SON
SMC5	SMC5
NAT6	NAT6
MOSPD2	MOSPD2
LRRFIP2	LRRFIP2
GTF2H3	GTF2H3
RBX1	RBX1
VAPB	VAPB
DCUN1D1	DCUN1D1
TRNT1	TRNT1
AP1AR	AP1AR
PPP2R3C	PPP2R3C
SPATA5	SPATA5
PHAX	PHAX
SGPP1	SGPP1
SART1	SART1
MED30	MED30
NUDT6	NUDT6
DNAJC18	DNAJC18
PITPNA	PITPNA
BCAS2	BCAS2
OTUD7B	OTUD7B
MNAT1	MNAT1
CSNK1G2	CSNK1G2
RNF25	RNF25
COQ2	COQ2
TMEM110	TMEM110
PTP4A2	PTP4A2
EIF2S2	EIF2S2
TBCE	TBCE

RBMXL1	RBMXL1
FAM76A	FAM76A
ABCE1	ABCE1
CTTNBP2NI	CTTNBP2NL
VARS	VARS
RFX7	RFX7
GAS8	GAS8
TRAF6	TRAF6
RBM4B	RBM4B
NMT2	NMT2
ZMYM6	ZMYM6
H3F3A	H3F3A
ZZZ3	ZZZ3
ZNRD1	ZNRD1
TAF3	TAF3
SMARCAL1	SMARCAL1
CD3EAP	CD3EAP
CLK4	CLK4
NOM1	NOM1
PINX1	PINX1
PTPN23	PTPN23
MRPS30	MRPS30
TFAP4	TFAP4
TMEM126A	TMEM126A
SAR1B	SAR1B
MPHOSPH6	MPHOSPH6
CWC15	CWC15
PRMT3	PRMT3
FAM114A2	FAM114A2
ZADH2	ZADH2
GSTCD	GSTCD
PNPLA8	PNPLA8
KRIT1	KRIT1
ELP2	ELP2
ZCCHC4	ZCCHC4
CLK2	CLK2
MAGOHB	MAGOHB
USP4	USP4
ZC3H15	ZC3H15
IFT80	IFT80
NAF1	NAF1
SEH1L	SEH1L
NTAN1	NTAN1
FAM76B	FAM76B
FAIM	FAIM
SBNO2	SBNO2
DAP3	DAP3
MRPS14	MRPS14

PDSS2	PDSS2
ARL6	ARL6
MINPP1	MINPP1
TRA2A	TRA2A
MON1B	MON1B
MTX2	MTX2
SAMM50	SAMM50
TLK2	TLK2
ATP5H	ATP5H
MTFR1	MTFR1
PAPD5	PAPD5
LRWD1	LRWD1
MFSD11	MFSD11
PPFIA1	PPFIA1
TXNL1	TXNL1
PFDN2	PFDN2
DYNLL2	DYNLL2
ALKBH3	ALKBH3
LEMD2	LEMD2
MBD2	MBD2
SLC10A7	SLC10A7
COG8	COG8
PHF5A	PHF5A
GNL2	GNL2
MRPS18C	MRPS18C
NLE1	NLE1
SSB	SSB
TMF1	TMF1
RASA1	RASA1
PDCD10	PDCD10
STK11	STK11
CMC1	CMC1
PHRF1	PHRF1
TAF6L	TAF6L
CSNK2A2	CSNK2A2
LARP4	LARP4
SARS	SARS
KCMF1	KCMF1
NDUFV3	NDUFV3
GIN1	GIN1
HSPA4	HSPA4
MRPS33	MRPS33
RIOK3	RIOK3
TRIM39	TRIM39
CLP1	CLP1
HARBI1	HARBI1
CGRRF1	CGRRF1
SEC22B	SEC22B

TAF5	TAF5
DYNC1I2	DYNC1I2
FXR2	FXR2
TCF25	TCF25
CEPT1	CEPT1
RBM26	RBM26
CSNK2A1	CSNK2A1
MMS19	MMS19
RIOK1	RIOK1
VCPIP1	VCPIP1
TSEN2	TSEN2
PDRG1	PDRG1
DTNBP1	DTNBP1
SUMO2	SUMO2
MTHFSD	MTHFSD
COPG2	COPG2
ESD	ESD
DDX52	DDX52
RBM25	RBM25
EED	EED
ZBTB6	ZBTB6
SRA1	SRA1
ACOX3	ACOX3
TIAL1	TIAL1
ZC3H14	ZC3H14
ILKAP	ILKAP
HNRNPF	HNRNPF
MYPOP	MYPOP
ZCCHC8	ZCCHC8
CLK3	CLK3
RUFY2	RUFY2
RABIF	RABIF
DNAJC19	DNAJC19
UBE2I	UBE2I
INTS12	INTS12
DHX16	DHX16
HMGB1	HMGB1
CC2D1B	CC2D1B
WDR70	WDR70
CWC25	CWC25
