# Supplementary figures and images for: The KrasG12D;Trp53fl/fl murine model of undifferentiated pleomorphic sarcoma is macrophage dense, lymphocyte poor, and resistant to immune checkpoint blockade
Source: PLoS One. 2021 Jul 9;16(7):e0253864. doi: 10.1371/journal.pone.0253864 (PMC8270133; doi:10.1371/journal.pone.0253864)

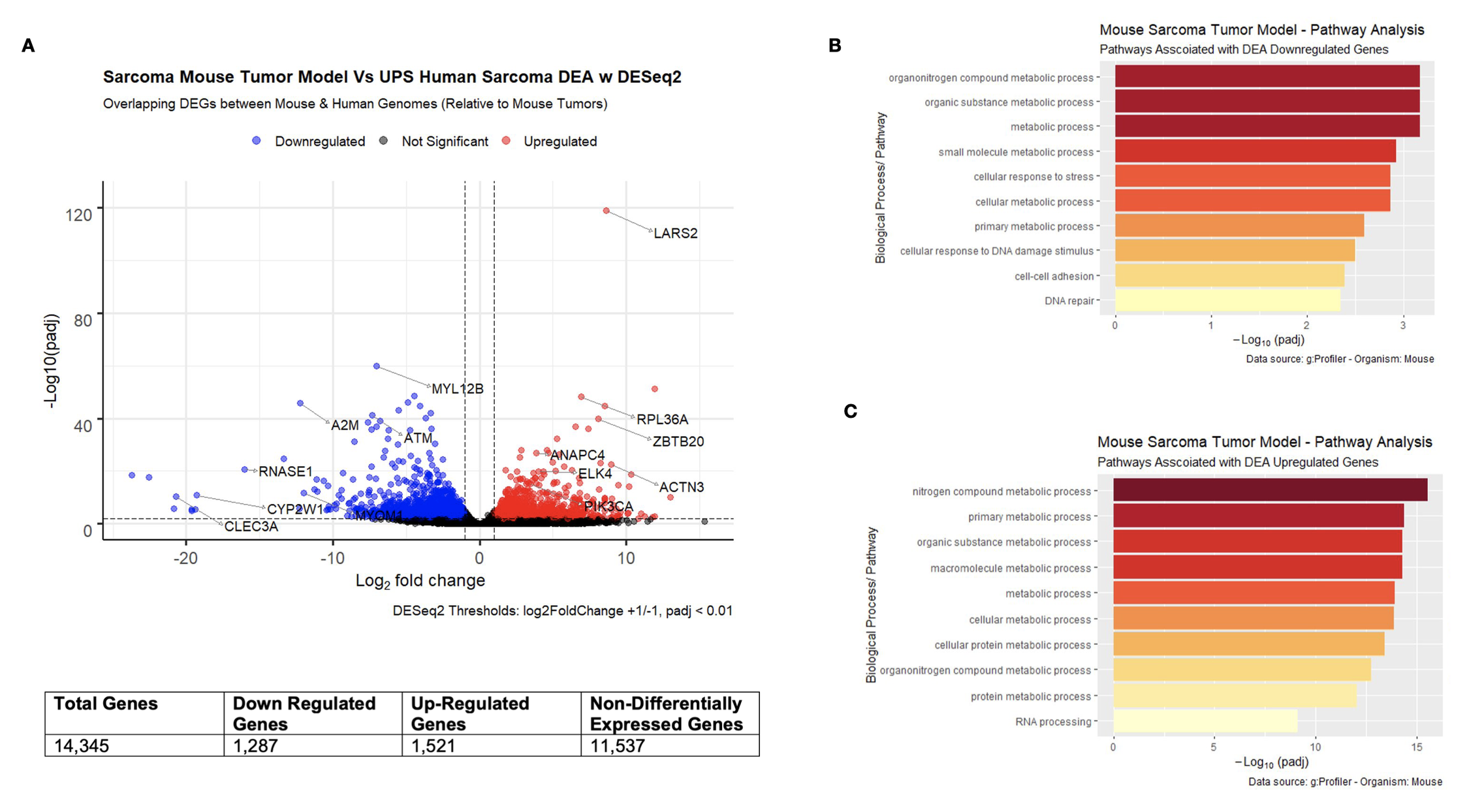

Supplement: S1 Fig — Gene expression signature of murine UPS is similar to human UPS (A) Volcano plot showing the differentially expressed genes in murine UPS relative to human UPS with a log fold change of 2 and a p value set to 0.01. Of the 14,345 genes analyzed, 11,537 genes (80%) were not differentially expressed demonstrating the similarities between the murine and human UPS. (B) Biological pathway analysis of the 1,281 genes (9%) that were downregulated in murine UPS relative to human UPS showing the top ten pathways associated with the downregulation. (C) Biological pathway analysis of the 1,521 upregulated genes (11%) in murine UPS relative to human UPS showing the top ten pathways associated with the upregulation. (TIF) [file pone.0253864.s003.tif]

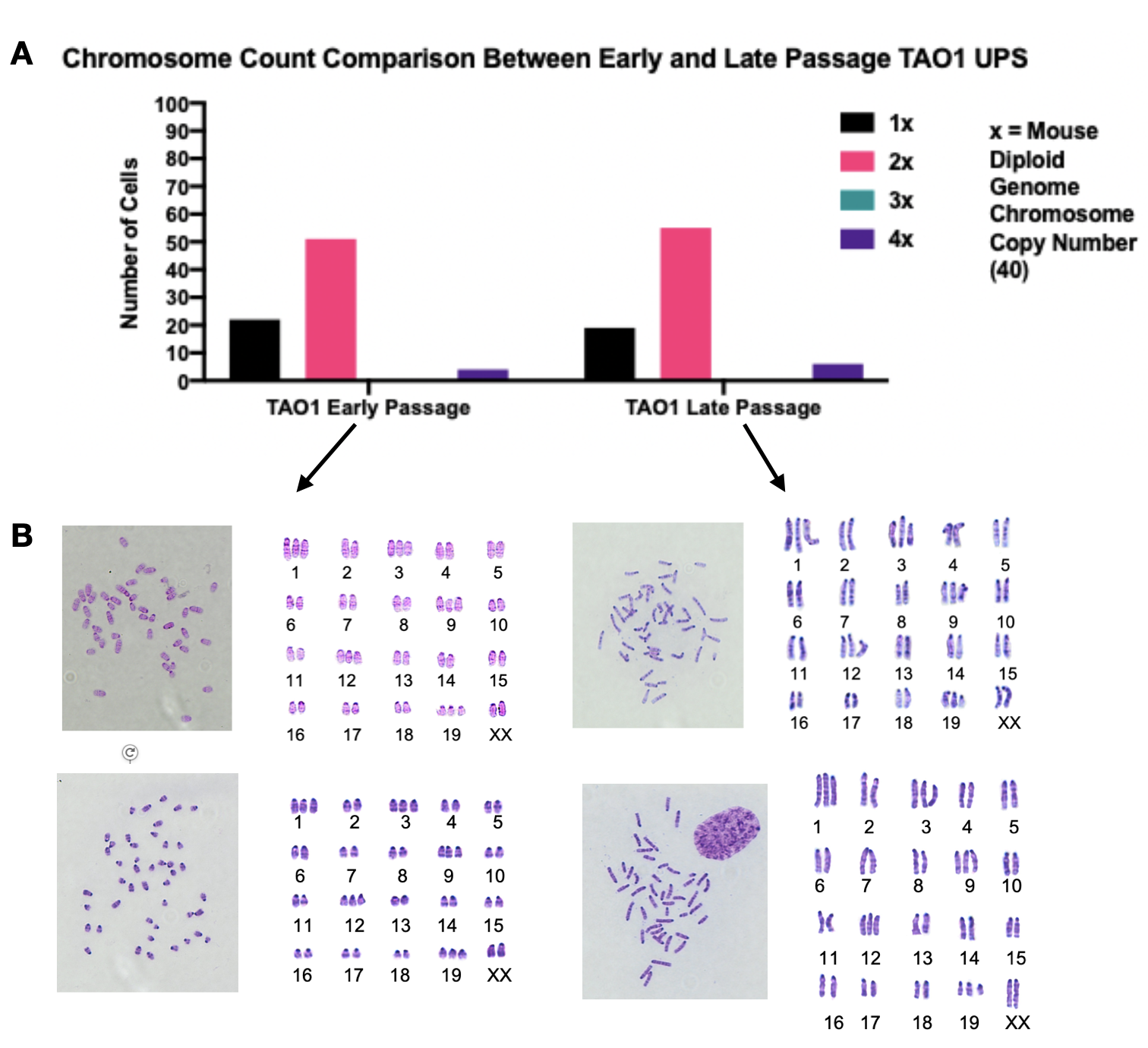

Supplement: S2 Fig — (A) Graphical representation of chromosome copy numbers for early and late passage TAO1 UPS cell lines as a function of normal diploid mouse chromosome count which is 40. 1x is defined as any chromosome count between 40–79, 2x as 80–119, 3x as 120–159, and 4x as 160–200. (B) G-banding of two cells with 45 chromosomes for both the early and late passage TAO1 UPS cell lines. (TIF) [file pone.0253864.s004.tif]
